# Tricoordinate Coinage Metal Complexes with a Redox‐Active Tris‐(Ferrocenyl)triazine Backbone Feature Triazine–Metal Interactions

**DOI:** 10.1002/chem.202000226

**Published:** 2020-04-22

**Authors:** Axel Straube, Peter Coburger, Mark R. Ringenberg, Evamarie Hey‐Hawkins

**Affiliations:** ^1^ Institute of Inorganic Chemistry Universität Leipzig Johannisallee 29 04103 Leipzig Germany; ^2^ Institute of Inorganic Chemistry Universität Stuttgart Pfaffenwaldring 55 70569 Stuttgart Germany; ^3^ Present address: Institute of Inorganic Chemistry Universität Regensburg Universitätsstraße 31 93051 Regensburg Germany

**Keywords:** coinage metal ions, ligand design, phosphane ligands, π interactions, tridentate ligands

## Abstract

2,4,6‐Tris(1‐diphenylphosphanyl‐1’‐ferrocenylene)‐1,3,5‐triazine (**1**) coordinates all three coinage metal(I) ions in a 1:1 tridentate coordination mode. The *C_3_*‐symmetric coordination in both solid state and solution is stabilised by an uncommon cation–π interaction between the triazine core and the metal cation. Intramolecular dynamic behaviour was observed by variable‐temperature NMR spectroscopy. The borane adduct of **1**, **1BH_3_**, displays four accessible oxidation states, suggesting complexes of **1** to be intriguing candidates for redox‐switchable catalysis. Complexes **1Cu**, **1Ag**, and **1Au** display a more complicated electrochemical behaviour, and the electrochemical mechanism was studied by temperature‐resolved UV/Vis spectroelectrochemistry and chemical oxidation.

## Graphical abstract

**One to hold them all**: A redox‐active tris(ferrocenyl)triazine‐based tris‐phosphane was shown to bind all coinage metal(I) ions in the same trigonal‐planar mode, stabilised by rare triazine–metal interactions. The intriguing solution‐state chemistry of these complexes was investigated by using variable‐temperature NMR spectroscopy and spectroelectrochemistry.

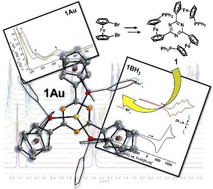

Over the past decades, *C_3_* symmetry has been an intriguing structural feature in ligand design, providing transition‐metal complexes with increased stability and minimising the possible number of transition states especially in the context of asymmetric homogeneous catalysis.^1^ One trend in modern‐day ligand design is to incorporate redox‐switchable units into existing ligand frameworks or to design de novo potential ligands featuring such groups. This method has paved the way for redox‐switchable catalysis (RSC).^2^ Embedded into the greater field of “smart”, that is, stimuli‐responsive catalysts,^3^ RSC is of particular interest in the context of catalyst recycling and orthogonal reactivity for building complex molecular structures. Among the available structural motifs, ferrocenyl groups have found the most widespread use in this field, owing to their highly reversible redox processes, their structural flexibility, and their amenability to many different synthetic procedures.^4^ Combining both approaches and downsizing our recently reported redox‐switchable dendritic ferrocenyl‐based catalysts,^5^ our present work aims to utilise the structurally appealing *C_3_*‐symmetric 1,3,5‐tris(ferrocenyl)arene platform^6^ (some examples are shown in Figure 1) for the design of stimuli‐responsive ligands. Having been extensively studied for their electrochemistry (**IV**, **VI**, **VII**) and, to some degree, suitability for nonlinear optics (**II**, **V**), these molecules—in themselves promising candidates for molecular materials as redox‐active building blocks—have not yet found their way into coordination chemistry with the notable exception reported by the Heck group, who used the central benzene core of **I** as η^6^‐coordinating ligand in **V**.6d

**Figure 1:**
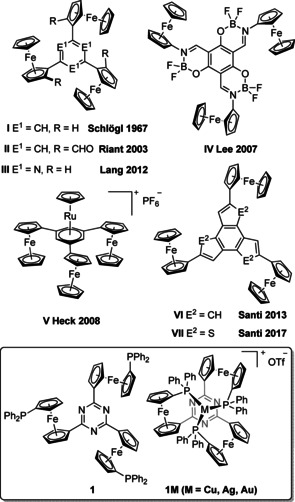
*C_3_*‐symmetric tris(ferrocenyl)arenes documented in the literature and presented in this work (box, bottom).

Therefore, we prepared tris‐phosphane **1** in a modular two‐step procedure from 1,1’‐dibromoferrocene (**2**)^7^ and cyanuric chloride (Scheme 1, left), noting that other arene cores can be utilised at this stage. Adapting a procedure from the Lang group for **III**,6e 2,4,6‐tris(1‐bromo‐1’‐ferrocenylene)‐1,3,5‐triazine (**4**) was obtained in good yield through a Negishi coupling reaction and, after chromatographic purification, was further reacted with chlorodiphenylphosphane. **4** provides an intriguing starting point for the preparation of complex structures based on the tris(ferrocenyl)arene scaffold. The resulting tris‐phosphane **1** and its borane adduct (**1BH_3_**) were fully characterised, including their solid‐state structures by single‐crystal X‐ray crystallography (XRD) (see Supporting Information).

**Scheme 1:**
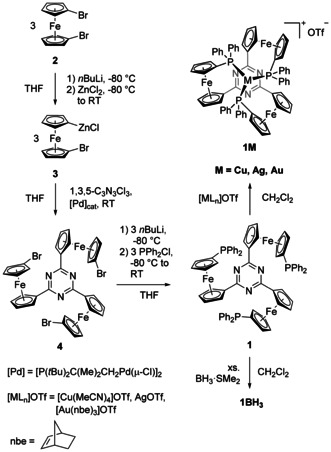
Preparation of tris‐phosphane **1** and its corresponding coinage metal complexes **1Cu**, **1Ag**, and **1Au**. Isolated yields are given in brackets.

In the solid state, **1** exhibits an all‐*syn* conformation, contrasting the *C*_3*v*_ symmetry in solution assessed by NMR spectroscopy and, thus, implying free rotation of the C_3_N_3_–ferrocenylene bonds. This prompted us to investigate the eligibility of this molecule for a tricoordinate binding mode towards coinage metal(I) ions, given their propensity for low coordination numbers and flexibility regarding their coordination geometry due to their closed‐shell d^10^ electronic configurations.^8^

Accordingly, **1** was reacted with suitable metal(I) triflate precursors in dichloromethane (Scheme 1, right). The corresponding 1:1 (metal‐to‐ligand) complexes **1Cu**, **1Ag**, and **1Au** were formed instantaneously, as shown by ^31^P{^1^H} NMR spectroscopy. They can be easily isolated as moderately air‐stable crystals in good yields. Crystals grown from dichloromethane/toluene (**1Cu**) or from 1,2‐dichloroethane/toluene (**1Ag**, **1Au**) proved suitable for XRD, allowing to ascertain the desired tricoordinate binding mode in the solid state (Figure 2).

**Figure 2:**
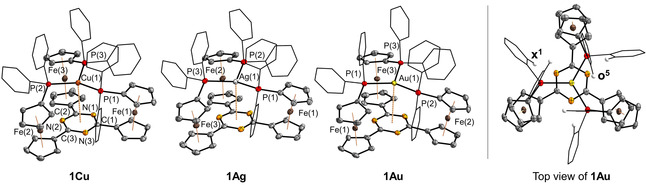
Molecular structures of coinage metal complexes of **1** and top view of **1Au** showcasing the propeller‐like arrangement of the complexes as well as two types of phenyl protons (**x^1^** and **o^5^**) in spectroscopically relevant positions. Thermal ellipsoids are set at the 50 % probability level. For clarity, the phenyl rings are drawn as wireframes, the triflate anions have been omitted, and hydrogen atoms except for **x^1^** and **o^5^** are not depicted.

According to the XRD data, the three complexes form close‐to‐isomorphous crystals (space group *P* $\bar{1}$ ), minor deviations in their cell parameters arising from anions and (disordered) solvent molecules (see Supporting Information). No contacts between the metal ions and their respective triflate anions are present. There are no noteworthy differences between the three ferrocenyl moieties; thus, only average values are listed (Table 1). The coinage metal ions adopt an almost ideal trigonal‐planar coordination environment with a small deflection of the metal from its position in an idealised *P*,*P*,*P* plane towards the triazine core (distance *d* in Table 1), also causing the deviations from the ideal 120° bond angles. The average M−P bond lengths are in line with the differences in the covalent radii of the metal ions.^9^ For **1Ag** and **1Au**, the absolute values are well within the reported range for trigonal‐planar tris‐phosphane complexes of silver(I) and gold(I), a structural motif still found to be quite rare among compounds published in the Cambridge Structural Database (CSD),^10^ particularly when only 1:1 complexes are considered. In the case of **1Cu**, the Cu−P bonds are among the longest that have been reported for this coordination mode (see the Supporting Information for a full list of 1:1 coinage metal‐tris‐phosphane complexes). Ligand **1** thus constitutes only the second example for a tris‐phosphane capable of binding all coinage metal ions in the same trigonal‐planar coordination mode without an additional ligand, as in tris{2‐(diphenylphosphino)ethyl}amine (*NP_3_*) complexes **VIII** (Figure 3).^11^

**Table 1:** Selected average bond lengths [Å], distances [Å], and angles [°] of complexes **1Cu**, **1Ag**, and **1Au**.

	**1Cu**	**1Ag**	**1Au**
P−M(1)	2.3261(7)	2.4910(6)	2.396(2)
C_3_N_3_⋅⋅⋅M(1)^[a]^	3.599	3.430	3.571
*d* ^[b]^	0.297	0.382	0.299
P_a_‐M(1)‐P_b_	118.38(3)	117.69(2)	118.46(6)
*γ* ^[c]^	1.35	1.10	0.287
*α* ^[d]^	172.68	173.22	173.11
*Θ* ^[e]^	7.48	6.91	7.45
*τ* ^[f]^	22.19	24.20	23.38

[a] Distance between a calculated C_3_N_3_ centroid and M(1). [b] Distance between M(1) and a plane defined by P(1), P(2), and P(3). [c] Angle between axis C_3_N_3_⋅⋅⋅M(1) and vector normal to the C_3_N_3_ plane. [d] Average tilt angle about Cp^P^(centroid)⋅⋅⋅Fe⋅⋅⋅Cp^C^(centroid) axes. [e] Average angle between mean planes through cyclopentadienyl (Cp^R^) rings. [f] Average torsion about the Cp^P^(centroid)⋅⋅⋅Fe⋅⋅⋅Cp^C^(centroid) axes. See the Supporting Information for a graphic representation of the geometric parameters.

**Figure 3:**
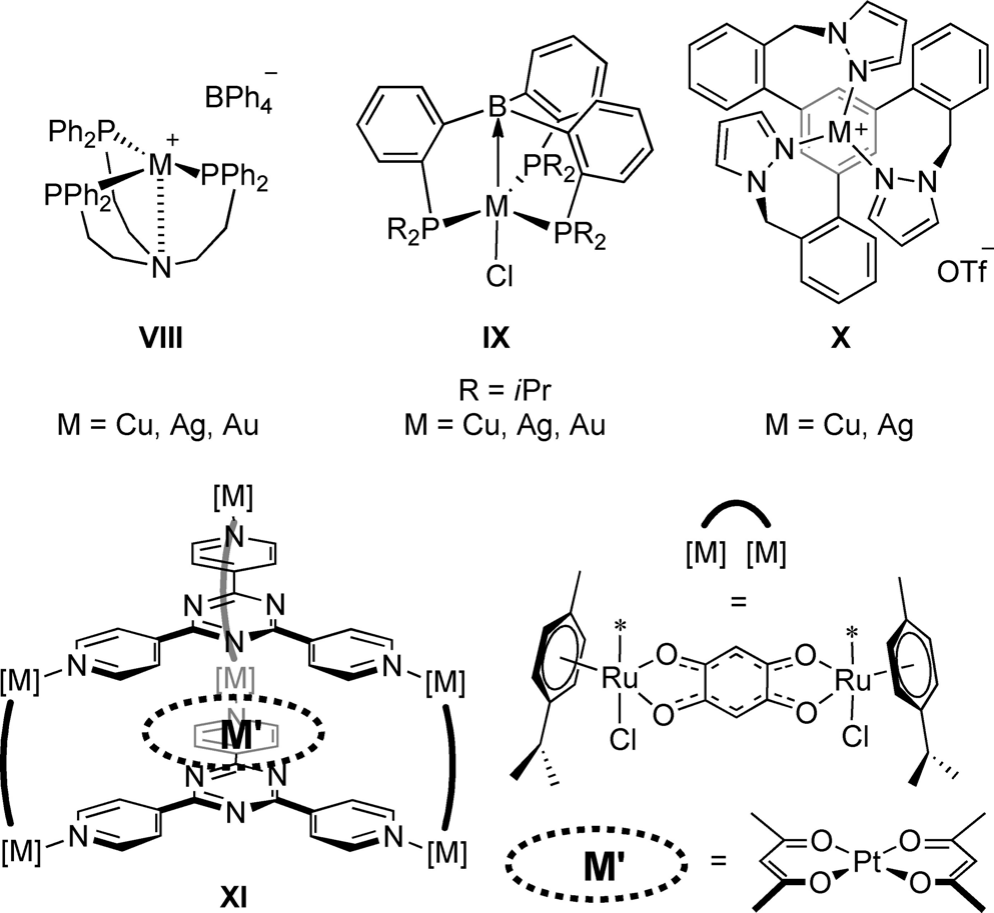
Selected examples of coordination compounds with trigonal‐planar coinage metal ions (**VIII–X**) and arene(**X**)‐/C_3_N_3_(**XI**)⋅⋅⋅M^I^ contacts.

A tris‐phosphino‐borane by Bourissou and co‐workers coordinates the coinage metal chlorides (complexes **IX**),^12^ with the Z‐type boron–metal interactions persisting upon chloride abstraction for gold(I) and copper(I).^13^ In these selected examples, the metal ions are in close contact with an additional donor atom (**VIII**) or a Z‐type acceptor group (**IX**). Broadening the ligand scope, similar complexes of a tris(pyrazolyl) ligand with copper(I) and silver(I), **X**,^14^ feature an interaction with the central arene core. Indeed, most trigonal‐planar 1:1 coinage metal complexes and all of those based on tris‐phosphanes generally feature such contacts, including metallophilic interactions,^15^ which could thus be considered a requirement. In analogy to **VIII**, **IX**, and **X**, complexes **1M** crystallise in a helically twisted, propeller‐like arrangement (Figure 2, right), represented by the synclinal conformation (*τ*=22.19°–24.20°) of the ferrocenylene groups;^16^ both enantiomers (*P* and *M*) are present in the unit cell as evident from the space group (*P* $\bar{1}$ ; see Supporting Information for a packing diagram of **1Au**).

In the case of triazine, the observed close, intramolecular C_3_N_3_⋅⋅⋅M^I^ contacts, combined with the almost perfect centring of the metal (signified by angle *γ*, Table 1) and classified as delocalised M⋅⋅⋅π(arene) interactions for gold by Tiekink,^17^ are still rare. For other, more electron‐rich arenes, close contacts between (coinage) metals and the ring system have been found to be important structural motifs with potential impact on catalytic activities.^17^, ^18^ Even though 1,3,5‐triazine has recently been recognised as a hybrid system capable of binding both cations and anions,^19^ only the latter has been exploited in many examples,^20^ while the former has mainly been investigated in silico for the alkaline metals.^21^ Complexes **1M**, reported here, show the closest C_3_N_3_⋅⋅⋅M^I^ distance for both copper(I) and silver(I). In the case of gold(I), a shorter (3.511 Å), yet intermolecular and less well‐centred contact (*γ*=10.7°) has been reported before.^22^

Among all metals, these distances are only underbid by bis(acetylacetonato)platinum(II) and ‐palladium(II) encapsulated in a *C_3_*‐symmetric molecular clip **XI** (Figure 3, C_3_N_3_⋅⋅⋅Pt=3.397 Å, *γ*=2.9°)^23^ and a less well‐centred nickel(II) analogue.^24^ The authors, however, only stated aromatic π–π interactions as the main reason for the successful encapsulation of both complexes. The reader is referred to the Supporting Information for a survey of such structures in the CSD. Computational methods were employed in order to gain more insight into the nature of the C_3_N_3_⋅⋅⋅M^I^ (M=Cu, Ag, Au) interaction. As expected and previously noted on interactions between triazine and sodium cations,21a the triazine core serves as an additional donor for the metal(I) ions. Studying the interaction of the metal with the parent triazine C_3_H_3_N_3_ (see Supporting Information), EDA‐NOCV analyses furthermore show polarisation of the triazine framework to significantly (20–23 %) contribute to the overall interaction energies. The Wiberg bond indices reveal weak interactions between the metals and the triazine core in the order (Ag>Au>Cu), the order also found for the C_3_N_3_⋅⋅⋅M distances of the complexes in their molecular structures.

Despite their striking similarity in the solid state, the behaviour of the complexes in CH_2_Cl_2_ turned out to be markedly different. While solutions of **1Ag** and **1Au** display only one ^31^P resonance, **1Cu** yields two ^31^P resonances at −7.3 ppm and −20.4 ppm (2:1), suggesting only two phosphanyl groups to be involved in the bonding of the Cu^I^ ion and in contradiction to its solid‐state structure. Similarly, the signals in the room‐temperature ^1^H NMR spectra of the three complexes are broadened (most strongly for **1Ag**), and, in the case of **1Cu**, are neither in line with the solid‐state structure nor the finding from the ^31^P{^1^H} NMR spectrum. We assumed fast interconversion of the two helical isomers of **1Ag** and **1Au**, as reported for **IX** at elevated temperatures, to be responsible for the line broadening and apparent *C*_3*v*_ symmetry in solution at and above room temperature.

Variable‐temperature (VT) NMR experiments were thus conducted in CD_2_Cl_2_ between 40 °C and −70 °C. Both the *C*_3*v*_‐symmetric ^1^H NMR spectrum of **1Au** (Figure 4, bottom left) and the severely broadened signals in the ^1^H NMR spectrum of **1Ag** sharpen in linewidth and break down into apparent *C_3_* symmetry [*T*_coal_(**1Ag**)=248±5 K, *T*_coal_(**1Au**)=268±5 K], rendering all eight ferrocenylene protons distinguishable (Figure 4, left). Most strikingly, the phenyl protons decoalesce and span the region from 5.8 ppm to 9.7 ppm, the most deshielded signal corresponding to the phenyl protons **x^1^** buried between the two ferrocenylene units and the most shielded signal to the protons **o^5^** closest to the gold(I) ion (Figure 2, right; Figure 4, bottom left), the latter in line with findings from the Mingos group.^25^ ^1^H,^1^H COSY NMR experiments conducted at −50 °C for **1Au** as well as NMR shielding parameters obtained from DFT calculations support this assignment, and the ^31^P{^1^H} VT NMR spectra agree with these findings.

**Figure 4:**
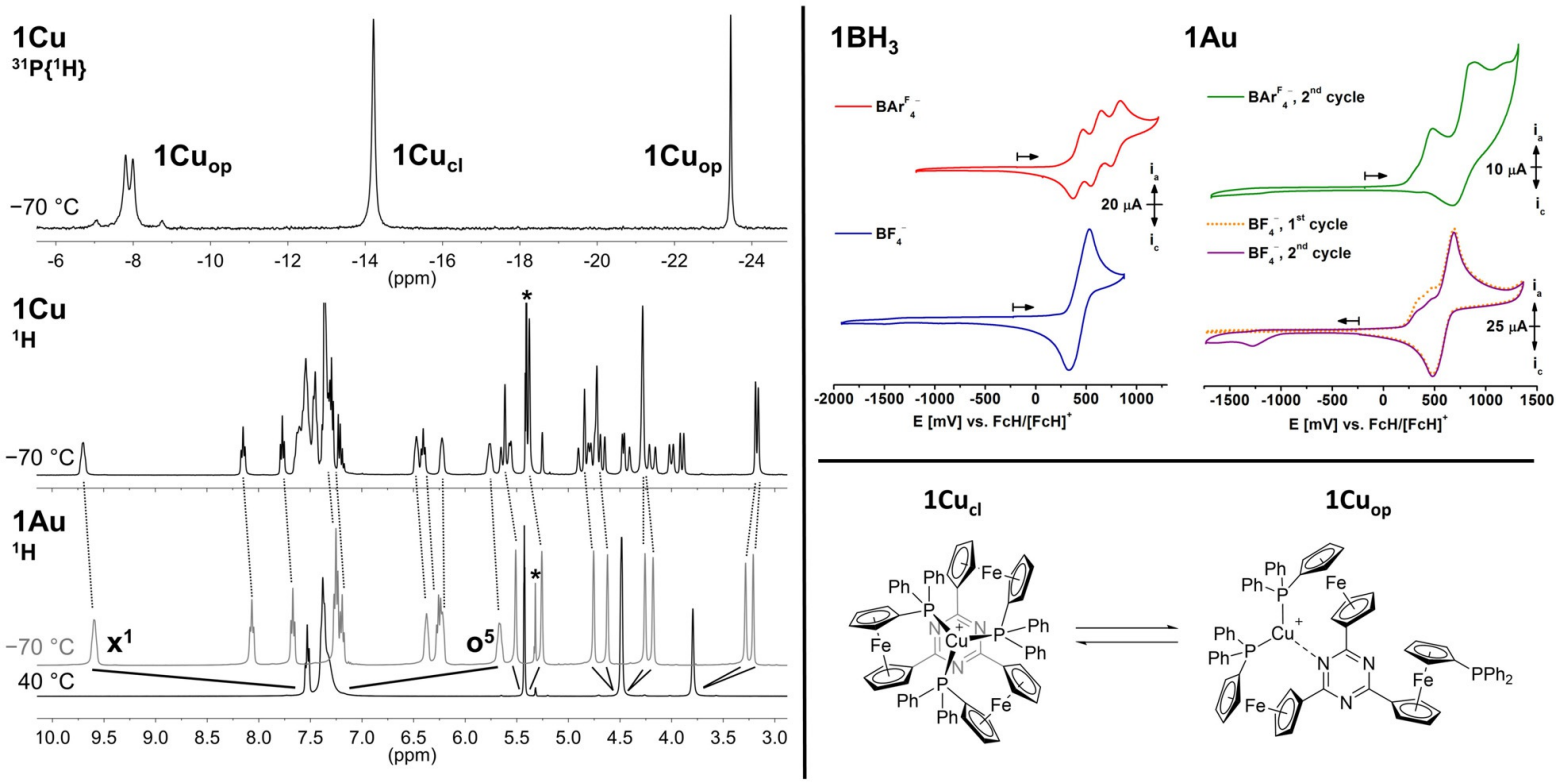
Left: VT ^1^H and ^31^P{^1^H} NMR spectra of **1Cu** and **1Au** at 40 °C and −70 °C; the asterisk denotes the CHDCl_2_ signal, **x^1^** and **o^5^** denote signals of interest (see also Figure 2, right). Solid lines represent the signal splitting, and dashed lines link analogous signals of **1Cu**_cl_ and **1Au**. Top right: Cyclic voltammograms of **1BH_3_** and **1Au** in the BF_4_^−^‐ and BAr^F^_4_^−^‐based SE; arrows denote the starting potential and initial scan direction at 100 mV s^−1^. The second of three consecutively measured cycles is shown if not noted otherwise. Bottom right: Equilibrium between two coordination isomers of **1Cu** in solution.

The low‐temperature ^1^H NMR spectrum of **1Cu** (Figure 4, middle left) exhibits signals of both a *C_3_*‐symmetric form **1Cu_cl_** and an open form **1Cu_op_** with *C_1_* symmetry (Figure 4, bottom right), featuring 24 partly overlapping signals for the ferrocenylene protons. Both forms are in a slow equilibrium, thus observable on the NMR timescale.

Likewise, the ^31^P{^1^H} NMR spectrum of **1Cu** at −70 °C (Figure 4, top left) consists of three signals; one sharp singlet at −14.2 ppm corresponding to the three Cu^I^‐bound phosphanyl moieties of **1Cu_cl_**, a second singlet at −23.2 ppm, and a higher‐order AB multiplet at −7.9 ppm in the intensity ratio of 1:2, thus corresponding to **1Cu_op_**. The respective ^2^ *J*_P,P_ coupling constant of 122 Hz is in line with previously reported values^26^ and constitutes a rare example of a resolved, Cu^I^‐mediated ^2^ *J*_P,P_ coupling in solution,^27^ mostly only being measurable by ^31^P CP‐MAS NMR studies in the solid state.^28^ In order to understand whether this behaviour is connected to the triflate anion—copper(I) is known to bind triflates in the solid state^29^—two different Cu^I^ complexes containing tetrafluoroborate **1CuBF_4_** and tetrakis{3,5‐bis(trifluoromethyl)phenyl}borate (BAr^F^_4_^−^) anions **1CuBAr^F^**_**4**_ were prepared and characterised, including their molecular structures in the solid state; no significant changes in the cation structural parameters were observed (see Supporting Information). The VT NMR spectra of **1CuBF_4_** and **1CuBAr^F^**_**4**_ in CD_2_Cl_2_ match those of **1Cu** very closely, and no peak splitting or broadening is observed for both the ^11^B and ^19^F NMR signals at low temperatures, suggesting no anion involvement in the equilibrium between **1Cu_cl_** and **1Cu_op_** in CD_2_Cl_2_. Most likely and also observable in a hypothetical structure for **1Cu_op_** obtained from a DFT calculation (see Supporting Information), coordination of Cu^I^ by the C_3_N_3_ core^30^ is present in the open form **1Cu_op_**. A bidentate form has also been proposed as the intermediate for the helical interconversion in a dissociative pathway by Bourissou and co‐workers.^12^

UV/Vis spectroscopy supports this notion; since the transition centred at 500 nm is mainly of d(Fe)‐π*(C_3_N_3_) character (cf. Supporting Information), bound Cu^I^ will strongly affect it, explaining the bathochromic shift and peak broadening observed between **1Cu** and **1Au** (Δ*λ*_max=_15 nm) as both compounds **1Cu** and **1Au** are practically identical in the solid state. Among **1M**, only **1Cu** shows a pronounced solvent influence on its NMR and UV/Vis spectra, indicative of the lability of the C_3_N_3_‐Cu contact in coordinating solvents. A UV/Vis‐titration of **1Cu** in CH_2_Cl_2_ with up to 2 equiv of CN^−^ further underpins this hypothesis (see Supporting Information).

Given our aim to utilise these complexes in RSC, their electrochemical characterisation by cyclic voltammetry (CV) was of great interest. Tris(ferrocenyl)triazine **III** itself had already been studied by the group of Lang and showed three separate, resolved oxidation waves in the CV when very weakly coordinating supporting electrolytes (SE), such as (*n*Bu_4_N)[B(C_6_F_5_)_4_], were used.6e Through UV/Vis spectroelectrochemical analyses, Lang and co‐workers could further demonstrate that mono‐ and di‐oxidised **III** have localised charges (Robin–Day class I), in contrast to other oxidised di‐ and tri(ferrocenyl)arenes.

The CV recorded in the BAr^F^_4_^−^‐based SE showed that the precursor **2** shows similar, anodically shifted redox potentials as **III** due to the presence of the bromine substituents, and one singular reversible redox wave in (*n*Bu_4_N)BF_4_ as the SE. The free ligand **1** can only be irreversibly oxidised during cyclic voltammetry in both SEs, in line with previous reports for ferrocenylphosphanes.^31^ In contrast, its borane‐protected analogue **1BH_3_** again allows for threefold oxidation in the BAr^F^_4_^−^‐based SE, proving the principal suitability of **1** for a four oxidation‐state catalyst system for RSC (Figure 4, top right).

The investigation of the electrochemical features of complexes **1M** by CV (exemplarily shown for **1Au** in Figure 4, top right) turned out to be less straightforward. None of **1M** exhibits a single, reversible redox wave in the BF_4_^−^‐ or the BAr^F^_4_^−^‐based SE. In the former, all complexes display an irreversible first oxidation step at 250–350 mV vs. FcH/[FcH]^+^, followed by further oxidation events. Linked to the first oxidation(s), one or two scan speed‐dependent reduction steps at much more cathodic potentials (**1Cu**: −1.4 V, **1Ag**: −450 mV and −1.3 V, **1Au**: −1.3 V vs. FcH/[FcH]^+^) hint at an electron transfer‐induced chemical reaction (EC) mechanism in which step C might involve a geometric rearrangement or an intramolecular electron transfer, producing a species **[1Au]^ox'^** which is more difficult to reduce.^32^ The complexes behave similar in the BAr^F^_4_^−^‐based SE, even though the delayed reductions are less prominent, particularly for **1Au** (Figure 4, top right).

To gain further understanding, spectroelectrochemical measurements were conducted in the BAr^F^_4_^−^‐based SE (Figure 5, left). At 25 °C and at −50 °C, the first oxidation of **1Au** yields a species **[1Au]^ox'^** that is reducible again to form **[1Au]** ^***x***^.

**Figure 5:**
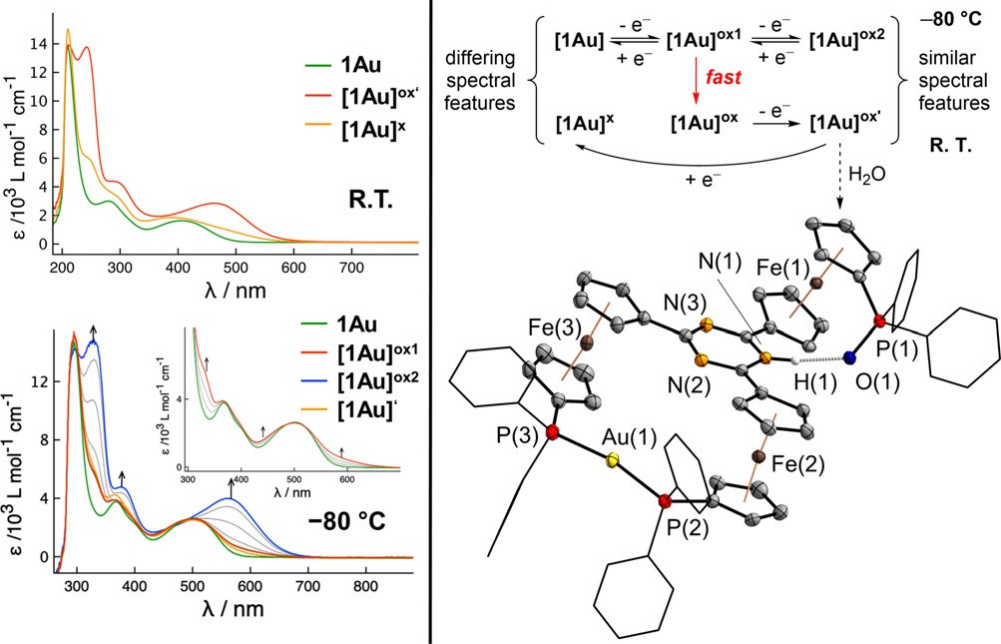
Left: UV/Vis SEC of **1Au** in 0.1 m (*n*Bu_4_N)BAr^F^_4_ in CH_2_Cl_2_, recorded at room temperature (top, intermediate spectra omitted for clarity) and at −80 *°*C (bottom, insert highlighting the first intermediate process). Right: Electrochemistry of **1Au** as assessed by VT SEC (top) and molecular structure of oxidation product **5**. Thermal ellipsoids are set at the 50 *%* probability level. For clarity, the phenyl rings are drawn as wireframes, the two BAr^F^_4_^*−*^ anions have been omitted, and hydrogen atoms except for H(1) are not depicted. For structural parameters, see the Supporting Information.

The UV/Vis spectrum of **[1Au]** ^***x***^ does however not match that of **1Au** (cf. Supporting Information). In contrast, performing the first oxidation at −80 °C results in the appearance of a markedly different UV/Vis spectrum with almost full reversibility upon reduction. The UV/Vis signature relates to an Fe‐centred oxidation **[1Au]^ox1^**,^33^ in line with DFT analyses finding the HOMO of **1Au** to be located at the ferrocenyl moieties (see Supporting Information). A second oxidation at −80 °C and higher potential generates a species **[1Au]^ox2^** with the same spectral features as **[1Au]^ox'^** generated from oxidising **1Au** at room temperature (see Supporting Information). We thus conclude that **1Au** is indeed oxidised following an EC mechanism in which the chemical reaction, fast at temperatures above −80 °C, transforms **[1Au]^ox1^** into an easier‐to‐oxidise species (Figure 5, top right) that is immediately oxidised further to **[1Au]^ox'^**/**[1Au]^ox2^**. Similar observations have been made for **1Cu**, whereas at −80 °C **1Ag** is showing the same characteristics as **1Au** at −50 °C (cf. Supporting Information).

In an attempt to isolate and characterise **[1Au]^ox'^** by chemical oxidation using two equivalents [thianthrenium]BAr^F^_4_ (Scheme 2), a few crystals of a dicationic, dicoordinate gold complex **5**, protonated at the triazine core and bearing a phosphine oxide moiety at the third ferrocenyl group (Figure 5, bottom right), were isolated.

**Scheme 2:**
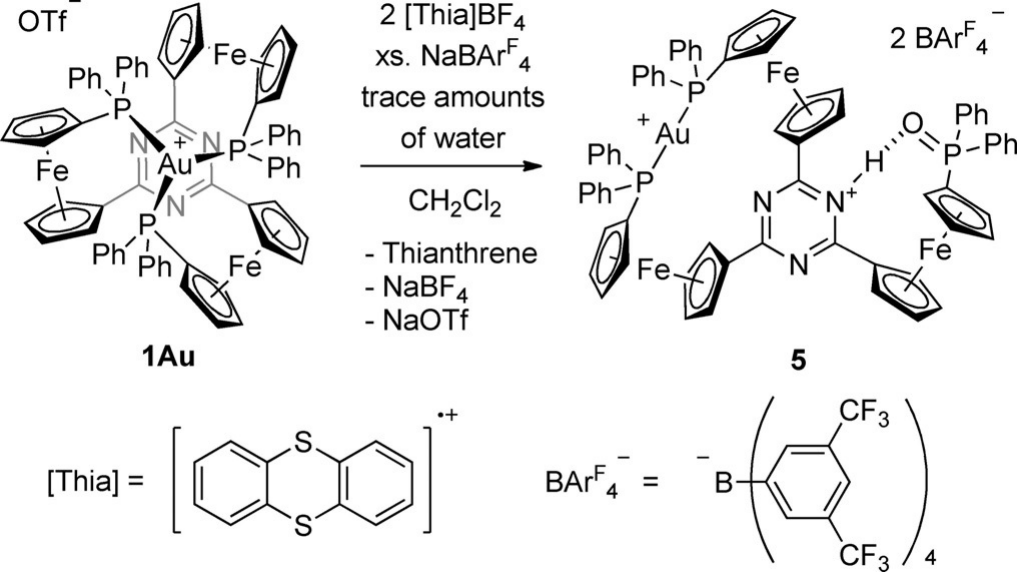
Chemical oxidation of **1Au** using [thianthrenium]BAr^F^_4_ in the presence of an excess of NaBAr^F^_4_ to yield oxidised product **5**.

Adventitious traces of water likely react with the highly reactive species **[1Au]^ox'^—**most likely a P‐centred radical formed after de‐coordination of one phosphanyl group^34^—to form this product in a formal oxidation from P^III^ to P^V^, further underpinning the proposed EC mechanism.

In summary, we have prepared the first tridentate ligand based on a *C_3_*‐symmetric tris(ferrocenyl)arene scaffold, **1**, and the corresponding coinage metal(I) complexes. The rare, C_3_N_3_‐supported and almost perfectly trigonal‐planar tricoordinate binding mode for all three coinage metal ions is tied to helical interconversion at low temperatures. While the borane adduct **1BH_3_** can be triply oxidised in a stepwise fashion, mononuclear complexes **1M** display a temperature‐dependent oxidation behaviour linked to an EC mechanism. Mono‐ and multinuclear complexes of **1** are intriguing candidates for RSC, and corresponding experiments are currently being carried out in our laboratories.

## Experimental Section

**Crystallographic data**: CCDC 1960989 (**1**), 1960985 (**1BH_3_**), 1960986 (**1Cu**), 1960991 (**1CuBF_4_**), 1960987 (**1CuBAr^F^**_**4**_), 1960992 (**1Ag**), 1960988 (**1Au**), and 1960990 (**5**) contains the supplementary crystallographic data for this paper. These data can be obtained free of charge from The Cambridge Crystallographic Data Centre.

## Conflict of interest

The authors declare no conflict of interest.

## Supporting information

As a service to our authors and readers, this journal provides supporting information supplied by the authors. Such materials are peer reviewed and may be re‐organized for online delivery, but are not copy‐edited or typeset. Technical support issues arising from supporting information (other than missing files) should be addressed to the authors.

Supplementary
