# Supplementary material for: Tricoordinate Coinage Metal Complexes with a Redox‐Active Tris‐(Ferrocenyl)triazine Backbone Feature Triazine–Metal Interactions
Source: Chemistry. 2020 Apr 22;26(26):5758–64. doi: 10.1002/chem.202000226 (PMC7317378; doi:10.1002/chem.202000226)
Supplement: Supplementary file 1 — Supplementary [file CHEM-26-5758-s001.pdf]

Chemistry–A European Journal

Supporting Information

Tricoordinate Coinage Metal Complexes with a Redox-Active Tris-(Ferrocenyl)triazine Backbone Feature Triazine–Metal Interactions

Axel Straube,[a] Peter Coburger,[a, c] Mark R. Ringenberg,[b] and Evamarie Hey-Hawkins*[a]

Table of contents

1. Experimental Procedures 2
1.1. General Procedures 2
1.2. Synthesis of 2,4,6-tris(1-bromo-1’-ferrocenylene)-1,3,5-triazine (**4**) 3
1.3. Synthesis of 2,4,6-tris{1-(diphenylphosphanyl)-1’-ferrocenylene}-1,3,5-triazine (**1**) 5
1.4. Synthesis of 2,4,6-tris{1-(diphenylphosphanyl borane)-1’-ferrocenylene}-1,3,5-triazine (**1BH$_3$**) 7
1.5. Synthesis of {2,4,6-tris(1-diphenylphosphanyl-1'-ferrocenylene)-1,3,5-triazine-κP^3} copper(I) triflate (**1Cu**) 10
1.6. Synthesis of {2,4,6-tris(1-diphenylphosphanyl-1'-ferrocenylene)-1,3,5-triazine-κP^3} copper(I) tetrafluoroborate (**1CuBF$_4$**) 16
1.7. Synthesis of {2,4,6-tris(1-diphenylphosphanyl-1'-ferrocenylene)-1,3,5-triazine-κP^3} copper(I) tetrakis{3,5-bis(trifluoromethyl)phenyl}borate (**1CuBAr$^F_4$**) 19
1.8. Synthesis of {2,4,6-tris(1-diphenylphosphanyl-1'-ferrocenylene)-1,3,5-triazine-κP^3} silver(I) triflate (**1Ag**) 21
1.9. Synthesis of {2,4,6-tris(1-diphenylphosphanyl-1'-ferrocenylene)-1,3,5-triazine-κP^3} gold(I) triflate (**1Au**) 25
1.10. Oxidation of **1Au** to yield {2,4-bis(1-diphenylphosphanyl-1'-ferrocenylene)-6-(1-diphenylphosphoryl)-1,3,5-triazin-1-um-κP^2} gold(I) bis[tetrakis{3,5-bis(trifluoromethyl)phenyl}borate] (**5**) 28
2. Results and Discussion 32
2.1. Single Crystal X-Ray Diffraction Analyses 32
2.2. CSD Searches and Results for Comparison 39
2.3. VT NMR Studies 44
2.4. Computational studies 58
2.4.1. Investigations on the metal-triazine interactions 58
2.4.2. Frontier molecular orbitals of complexes **1M** 60
2.4.3. Calculation of UV/Vis spectra 61
2.5. UV/Vis spectroscopy 65
2.5.1. UV/Vis spectra of metal complexes in CH_2Cl_2, THF, and CH_3CN 65
2.5.2. UV/Vis titration of **1Cu** with (*n*Bu$_4$N)CN 67
2.6. Electrochemistry 69
2.7. Spectroelectrochemistry 81
2.7.1. Spectroelectrochemical measurements of **1Au** 81
2.7.2. Spectroelectrochemical measurements of **1Ag** 86
2.7.3. Spectroelectrochemical measurements of **1Cu** 87
References 88
Author Contributions 89
Appendix - Cartesian coordinates of optimised molecular structures 89

1. Experimental Procedures

1.1. General Procedures

Syntheses

All reactions and manipulations were carried out under an atmosphere of either nitrogen or argon using standard Schlenk line techniques unless stated otherwise. Thin-layer chromatography (TLC) with silica gel 60 F_{254} on glass or aluminium sheets available from Merck KGaA was used for monitoring the ligand synthesis reactions. Column chromatography was performed using a Biotage Isolera 1 automatic purification system with SNAP (silica, particle diameter: 0.040 to 0.065 mm) and SNAP Ultra (silica, spherical particle, diameter: 0.025 mm) cartridges using solvents purged with nitrogen prior to use. The fractions were detected by an integrated UV/Vis detector.

Molecular sieves (3 and 4 Å) were activated at 300 °C in vacuo for a minimum of 3 h. Dry, oxygen-free solvents (THF, acetonitrile, CH_2Cl_2, Et_2O, hexanes, and toluene) were obtained from an MBraun Solvent Purification System MB SPS-800 and directly stored over 4 Å molecular sieves, except for THF, which was further distilled from potassium/benzophenone and stored over 4 Å molecular sieves. 1,2-Dichloroethane was degassed using the freeze-pump-thaw method and stored over 4 Å molecular sieves. For the use in NMR measurements, THF-d_8 was distilled from potassium/benzophenone, CD_2Cl_2, $CDCl_3$ and CD_3CN were dried by stirring over P_2O_5 at room temperature for several days, followed by vacuum transfer into a storage flask and degassing by the freeze-pump-thaw method. The solvents were stored over 4 Å (THF-d_8, CD_2Cl_2, $CDCl_3$) or 3 Å (CD_3CN) molecular sieves.

$[P(tBu)_2C(CH_3)_2CH_2Pd(\mu\text{-}Cl)]_2$,[1] 1,1'-dibromoferrocene,[2] $Na[B\{3,5\text{-}C_6H_3(CF_3)_2\}_4]$ ($NaBAr^F{}_4$),[3] $(nBu_4N)[B\{3,5\text{-}C_6H_3(CF_3)_2\}_4]$ ($(nBu_4N)BAr^F{}_4$),[4] and [thianthrenium]tetrafluoroborate[5] were synthesised as described in the literature. $[Cu(CH_3CN)_4]BF_4$ was prepared in a modified version of the procedure for $[Cu(CH_3CN)_4]PF_6$ using aqueous HBF_4.[6] $[Au(\eta^2\text{-nbe})_3]OTf$ was prepared in a modified version of the published synthesis for $[Au(\eta^2\text{-nbe})_3]SbF_6$ using AgOTf.[7] Ph_2PCl was distilled and stored under nitrogen prior to use. Cyanuric chloride was recrystallised from hot heptanes and stored under nitrogen prior to use. All other chemicals were used as purchased.

NMR spectra were recorded with a BRUKER Avance III HD 400 MHz NMR spectrometer at 25 °C (frequencies of ^{1}H: 400.13 MHz; ^{11}B: 128.38 MHz; ^{13}C 100.63 MHz; ^{19}F: 376.53 MHz: ^{31}P: 161.99 MHz). Pseudo-triplets of ferrocenyl protons are abbreviated as *pt* and their observable *H*,*H* coupling constants are given. TMS was used as the internal standard in the ^{1}H and ^{13}C NMR spectra, and spectra of all other nuclei were referenced to TMS using the Ξ scale.[8]

Electrospray ionisation mass spectrometry was performed with an ESI ESQUIRE 3000 PLUS spectrometer with an IonTrap analyser from Bruker Daltonics, or a MicroTOF spectrometer from Bruker Daltonics with a ToF analyser in positive mode. As solvents for the measurements, THF, CH_2Cl_2, CH_3CN, MeOH, or mixtures of these solvents were used. Dry, oxygen-free solvents were used for air-sensitive species. Elemental analyses were performed with a VARIO EL elemental analyser from Heraeus. Melting points were determined with a Gallenkamp MPD350·BM2.5 melting point device and are reported uncorrected. FTIR spectra were obtained with a PerkinElmer FT-IR spectrometer Spectrum 2000 as KBr pellets and with a Thermo Scientific Nicolet iS5 with an ATR unit in the range from 4000 to 400 cm^{-1}. UV/Vis spectra were recorded on a PerkinElmer UVV-IS-NIR Lambda 900 spectrometer in quartz cuvettes (d = 10 mm). Sample concentration was in the range of $3\cdot10^{-5}$ $mol\cdot L^{-1}$.

Electrochemistry

Cyclic voltammetry (CV) measurements on 1.0 $mmol\cdot L^{-1}$ analyte solutions in dry, oxygen-free dichloromethane containing 0.1 $mol\cdot L^{-1}$ $(nBu_4N)BF_4$ or $(nBu_4N)[B\{3,5\text{-}C_6H_3(CF_3)_2\}_4]$ as supporting electrolyte have been conducted in a three-electrode setup (GAMRY Instruments, SP-50 potentiostat by BioLogic Science Instruments) under a blanket of nitrogen at room temperature. The glassy-carbon working electrode (ALS; surface area 0.07 cm^2) and the counter electrode (neoLab; platinum wire, 99.9%) were put in the analyte solution, while the reference electrode (ALS; $Ag/AgNO_3$ (0.01 $mol\cdot L^{-1}$) in 0.1 $mol\cdot L^{-1}$ tetrabutylammonium hexafluorophosphate in dry, oxygen-free CH_3CN) was connected to the cell via a bridge tube (filled with the supporting electrolyte) through Vycor tips. The reference electrode was calibrated against decamethylferrocene as an internal standard at the end of the CV experiment,[9] and the results were converted to the $FcH/[FcH]^+$ scale in accordance with the IUPAC requirements.[10]

Spectroelectrochemistry was performed using an optically transparent thin-layered electrode (OTTLE) cooled using a liquid nitrogen cryostat as described previously.[11]

X-ray crystallography

The data were collected on a Gemini-CCD diffractometer (RIGAKU INC.) using Mo-K_α radiation (λ=0.71073 Å), ω-scan rotation. Data reduction was performed with CrysAlis Pro[12] including the program SCALE3 ABSPACK[13] for empirical absorption correction. The structure solutions for **1**, **1BH**$_3$, **1Cu**, **1CuBF**$_4$, **1CuBAr**^{F}$_4$, **1Ag**, **1Au**, and **5** were performed with SHELXS-97 (direct methods).[14] The anisotropic full-matrix least-squares refinement on F^2 of all non-hydrogen atoms was performed with SHELXL-97.[14] Except for disordered solvent molecules, all non-hydrogen atoms were refined with anisotropic thermal parameters and the HFIX command was used to locate all hydrogen atoms for non-disordered regions of the structure. Structure figures were generated with Mercury (versions 3.8 and 3.10).[15] CCDC 1960989 (**1**), 1960985 (**1BH**$_3$), 1960986 (**1Cu**), 1960991 (**1CuBF**$_4$), 1960987 (**1CuBAr**^{F}$_4$), 1960992 (**1Ag**), 1960988 (**1Au**), and 1960990 (**5**) contain the supplementary crystallographic data for this paper. These data are provided free of charge by The Cambridge Crystallographic Data Centre.

Computational methods

All calculations were carried out with the ORCA program package.[16] All geometry optimisations were performed at the BP86-D3BJ/def2-TZVP[17] level of theory in the gas phase. For calculations involving silver and gold the respective effective core potentials, namely def2-ECP,[18] were used. Frequency calculations were carried out to confirm the nature of stationary points found by geometry optimisations. Density fitting techniques, also called resolution-of-identity approximation (RI),[19] were used for GGA calculations, whereas the RIJCOSX[20] approximation was used for time-dependent DFT (TDDFT) calculations. Wiberg bond indices (WBI) were calculated using the built-in NBO3.1.[21] module of Gaussian09[22] on the geometries obtained with ORCA (using the BP86 functional in combination with the LANL2DZ[23] basis set). ^{1}H NMR shieldings were calculated at the TPSS/def2-TZVP[24,17c] level of theory in the gas phase using the calculated proton shift of tetramethylsilane as reference.

1.2. Synthesis of 2,4,6-tris(1-bromo-1'-ferrocenylene)-1,3,5-triazine (4)

A stirred solution of 3.50 g (10.2 mmol, 3.30 eq.) 1,1'-dibromoferrocene in 30 mL THF was cooled to −80 °C (ethyl acetate/$N_{2(l)}$), and 6.30 mL *n*BuLi in *n*-hexane (1.54 mol·L^{-1}, 9.72 mmol, 3.15 eq.) were added dropwise over the course of 10 min. A dark-orange precipitate formed, and the solution was kept stirring at this temperature for a further 30 min. A freshly prepared solution of 1.45 g (10.6 mmol, 3.45 eq.) zinc chloride in 10 mL THF was added, the clear solution was warmed to room temperature and stirred for a further 2 hours. In a separate flask, 570 mg (3.08 mmol, 1.00 eq.) cyanuric chloride and 32 mg (46 µmol, 1.5 mol%) $[P(tBu)_2C(CH_3)_2CH_2Pd(\mu\text{-}Cl)]_2$ were dissolved in 15 mL THF and transferred to the reaction mixture *via* cannula, immediately followed by a darkening of the red colour. The reaction mixture was left stirring at room temperature overnight. TLC (CH_2Cl_2/hexanes, 3:1) then indicated the complete conversion of the cyanuric chloride; all further work-up was carried out under ambient conditions.
For quenching, 30 mL of brine were added, and the phases were separated. The aqueous phase was extracted with ethyl acetate (2x 20 mL), the combined organic phases were dried over Na_2SO_4, followed by filtration over neutral alumina using ethyl acetate as the eluent. Column chromatography (CH_2Cl_2/hexanes, gradient 25–100%) allowed for isolation of **4** (1.41 g, 53% yield) as a deep red microcrystalline solid.

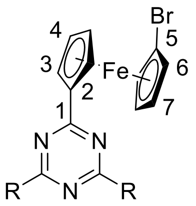

R_f = 0.62 (CH_2Cl_2/hexanes 3:1); m.p. 156–157 °C; ^{1}H NMR (CD_2Cl_2): δ [ppm] = 5.32 (pt, $J_{H,H}$ = 1.9 Hz, 6H, H3), 4.61 (pt, $J_{H,H}$ = 1.9 Hz, 6H, H4), 4.39 (pt, $J_{H,H}$ = 1.9 Hz, 6H, H6), 4.12 (pt, $J_{H,H}$ = 1.9 Hz, 6H, H7); ^{13}C{^{1}H} NMR (CD_2Cl_2): δ [ppm] = 175.0 (s, C1), 82.9 (s, C2), 79.1 (s, C5), 74.3 (s, C3), 72.5 (s, C4), 72.2 (s, C6), 69.4 (s, C7); IR (KBr): ṽ [cm^{-1}] = 2927 (m), 2297 (w), 2254 (m), 1418 (w), 1366 (m), 1084 (m), 1037 (w), 844 (vs, br), 743 (m), 560 (s), 484 (m); UV/Vis (CH_2Cl_2): λ_{max} [nm] (ε [mol^{-1}·L·cm^{-1}]) = 463 (3200), 350 (7200), 288 (34500), 248 (45500); HRMS (ESI): m/z calcd. for $C_{33}H_{24}Br_3Fe_3N_3^+$: 869.7628 [*M*]$^+$, found: 869.7621; elemental analysis calcd. [%] for $C_{33}H_{24}Br_3Fe_3N_3$: C 45.57, H 2.78, N 4.83; found: C 45.36, H 2.54, N 4.84.

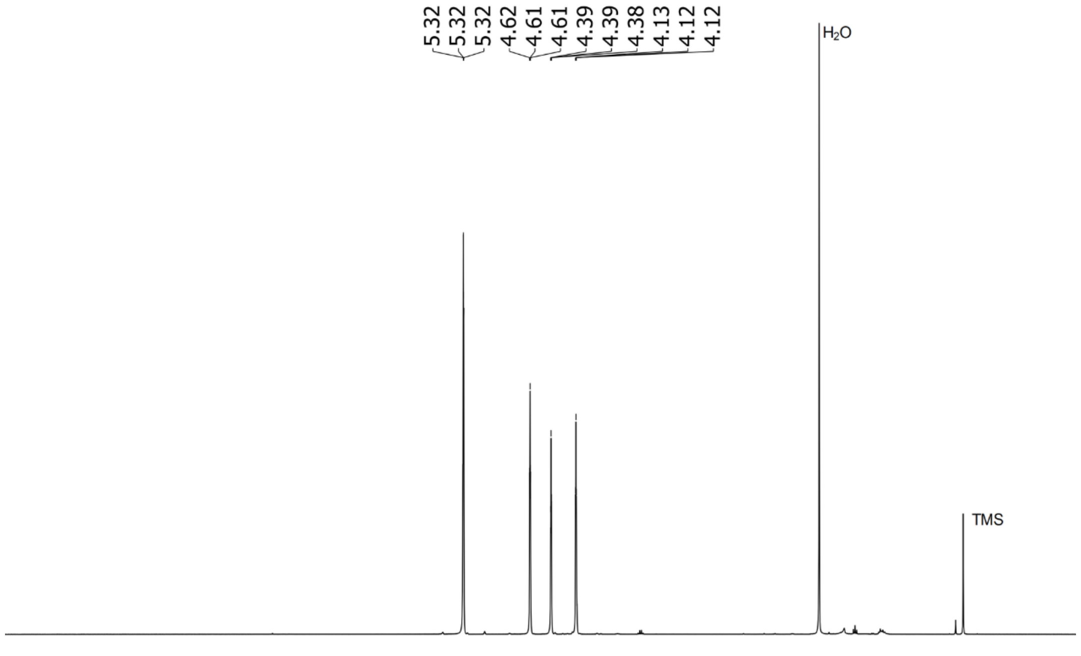

Figure S1. ^{1}H NMR spectrum of **4** in CD_2Cl_2.

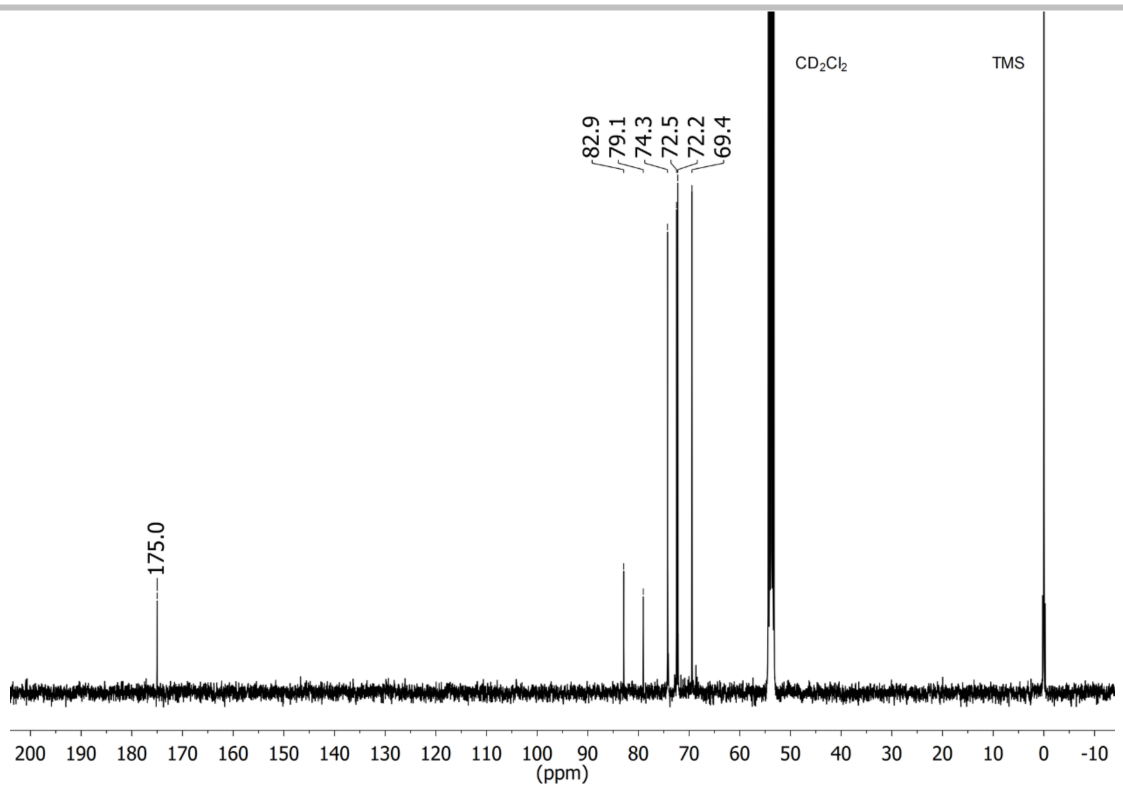

Figure S2. $^{13}C\{^{1}H\}$ NMR spectrum of **4** in CD_2Cl_2.

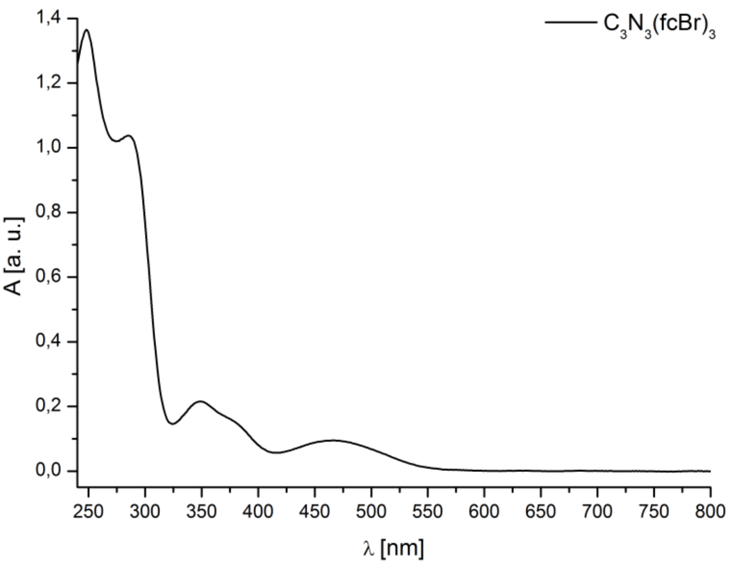

Figure S3. UV/Vis spectrum of **4** in CH_2Cl_2.

1.3. Synthesis of 2,4,6-tris{1-(diphenylphosphanyl)-1'-ferrocenylene}-1,3,5-triazine (1)

A stirred solution of 1.41 g (1.62 mmol, 1.00 eq.) **4** in 50 mL THF was cooled to −80 °C (ethyl acetate/$N_{2(l)}$) and 3.47 mL (1.54 mol·L^{-1}, 5.35 mmol, 3.30 eq.) *n*BuLi in *n*-hexane were added dropwise over the course of 30 min, resulting in a strong intensification of the red colour. The mixture was kept stirring for 2 hours at −80 °C, followed by the dropwise addition of 1.23 g (5.59 mmol, 3.45 eq.) chlorodiphenylphosphane dissolved in 20 mL THF at the same temperature. After slowly warming to room temperature overnight and stirring at 60 °C for 1 hour, TLC (CH_2Cl_2/hexanes, 4:1) indicated full conversion of **4**. For quenching, a degassed saturated aqueous solution of NH_4Cl was added to the fervently stirred mixture *via* cannula. The phases were separated, and the aqueous phase was extracted with diethyl ether (2x 10 mL). The combined organic phases were dried over degassed $MgSO_4$, filtered, and then the product mixture was adsorbed on Celite® and subjected to column chromatography (CH_2Cl_2/hexanes, gradient 20–100%). Crystals suitable for single crystal X-ray diffraction analysis were grown from a so-obtained fraction at 7 °C. Following the unification of the fractions and the removal of solvent under reduced pressure, **1** was filtered over degassed silica using CH_2Cl_2, and the solvent removed *in vacuo*, yielding **1** (1.00 g, 52% yield).

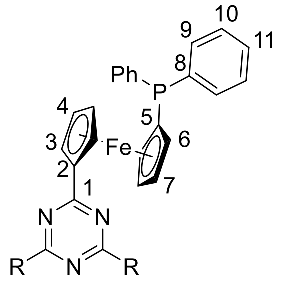

R_f = 0.40 (CH_2Cl_2/hexanes 4:1); m.p. 86–87 °C; ^{1}H NMR (CD_2Cl_2): δ [ppm] = 7.40–7.28 (m, 30H, H9,10,11), 5.17 (pt, $J_{H,H}$ = 2.0 Hz, 6H, H3), 4.41 (pt, $J_{H,H}$ = 2.0 Hz, 6H, H4), 4.27 (pt, $J_{H,H}$ = 1.8 Hz, 6H, H7), 4.04 (m, 6H, H6); ^{13}C{^{1}H} NMR (CD_2Cl_2): δ [ppm] = 175.1 (s, C1), 138.9 (d, $^1J_{C,P}$ = 10.7 Hz, C8), 133.4 (d, $^2J_{C,P}$ = 19.6 Hz, C9), 128.5 (s, C11), 128.1 (d, $^3J_{C,P}$ = 6.8 Hz, C10), 80.9 (s, C2), 77.8 (d, $^1J_{C,P}$ = 8.9 Hz, C5), 74.3 (d, $^2J_{C,P}$ = 14.4 Hz, C6), 73.4 (d, $^3J_{C,P}$ = 3.7 Hz, C7), 72.8 (s, C4), 70.6 (s, C3); ^{31}P{^{1}H} NMR (CD_2Cl_2): δ [ppm] = −18.4 (s); IR (KBr): $\tilde{\nu}$ [cm^{-1}] = 3049 (w), 2963 (w), 2922 (w), 1513 (vs), 1432 (m), 1380 (m), 1359 (m), 1319 (w), 1262 (w), 1159 (w), 1027 (m), 828 (m), 742 (m), 697 (m), 500 (m); UV/Vis (CH_2Cl_2): λ_{max} [nm] (ε [mol^{-1}·L·cm^{-1}]) = 468 (3300), 354 (7600), 250 (63100); HRMS (ESI): m/z calcd. for $C_{69}H_{55}Fe_3N_3P_3^+$: 1186.1659 [M+H]$^+$, found: 1186.1649; elemental analysis calcd. [%] for $C_{69}H_{54}Fe_3N_3P_3$: C 69.90, H 4.59, N 3.54; found: C 69.20, H 4.49, N 3.47.

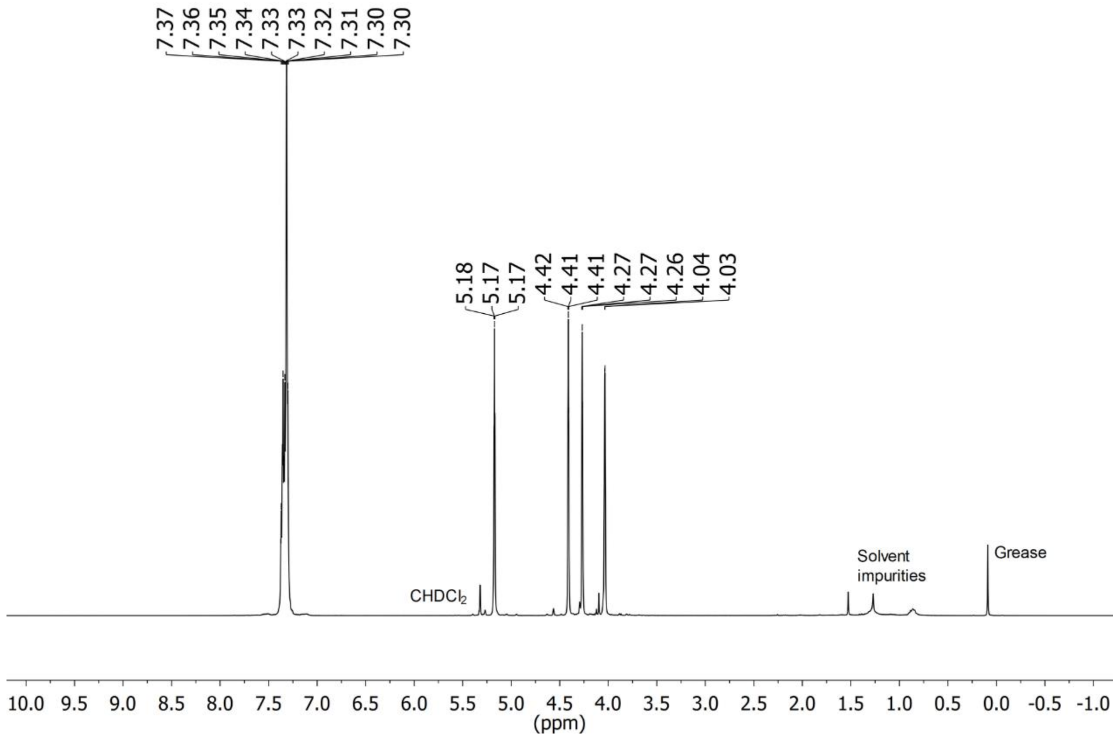

Figure S4. ^{1}H NMR spectrum of **1** in CD_2Cl_2.

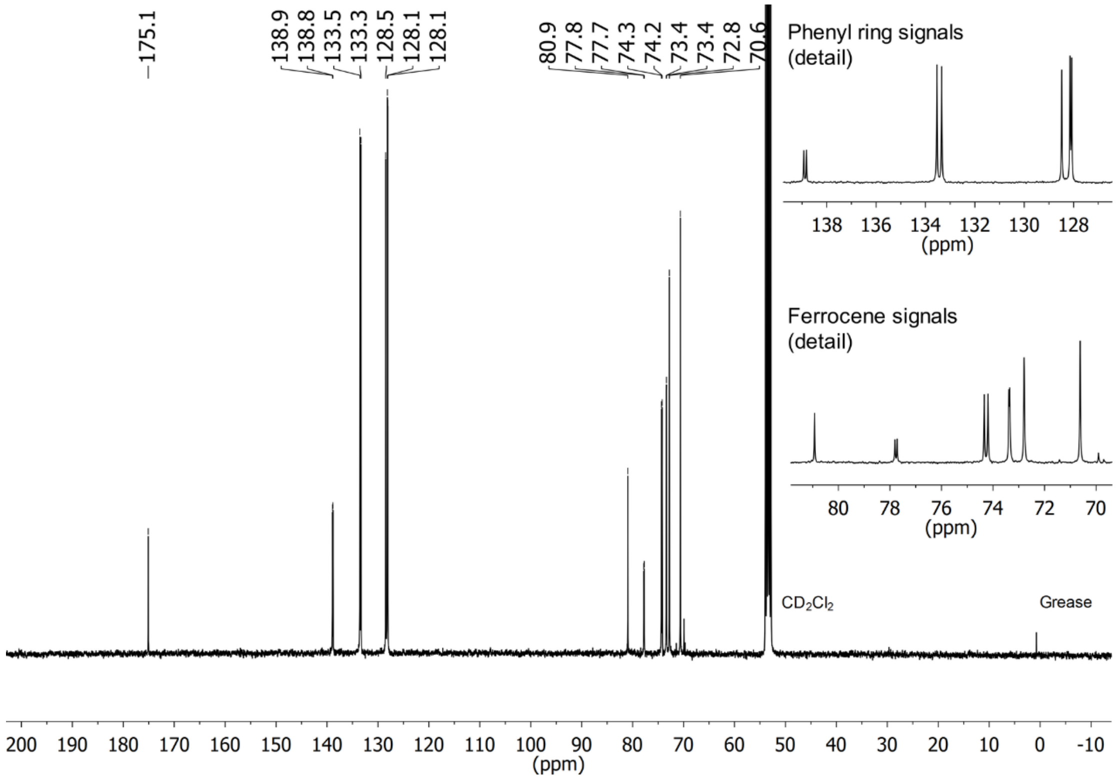

Figure S5. $^{13}C\{^1H\}$ NMR spectrum of **1** in CD_2Cl_2; the inserts show details of the phenyl (top right) and the ferrocenyl region (bottom right) of the spectrum.

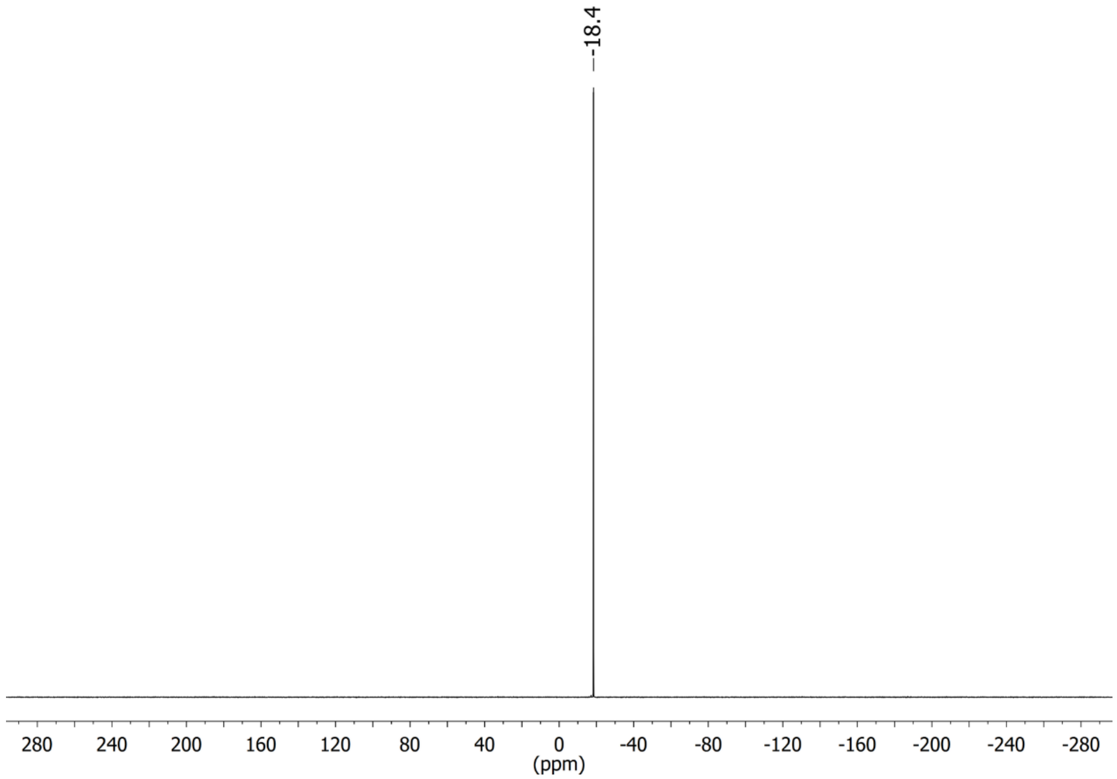

Figure S6. $^{31}P\{^1H\}$ NMR spectrum of **1** in CD_2Cl_2.

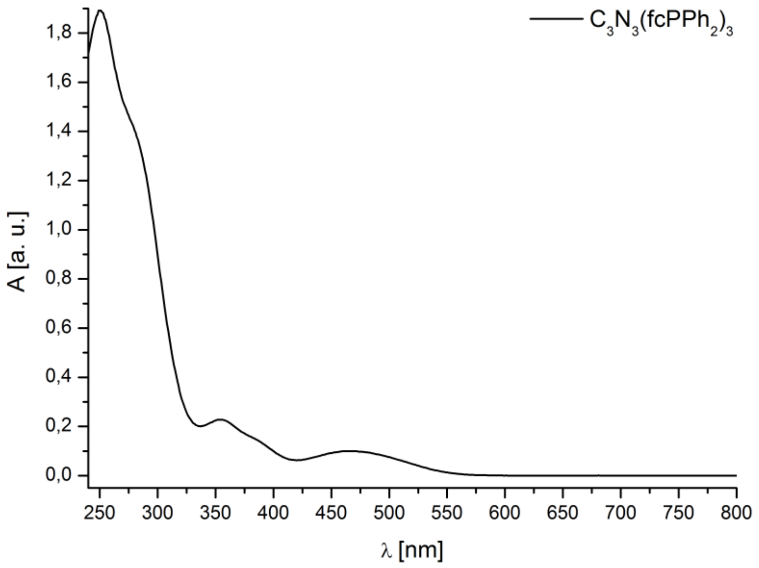

Figure S7. UV/Vis spectrum of **1** in CH_2Cl_2.

1.4. Synthesis of 2,4,6-tris{1-(diphenylphosphanyl borane)-1'-ferrocenylene}-1,3,5-triazine (1BH$_3$)

A stirred solution of 90 mg (76 µmol, 1.0 eq.) **1** in 10 mL CH_2Cl_2 was cooled to 0 °C (ice/water) and 0.13 mL (2.0 mol·L^{-1}, 270 µmol, 3.5 eq.) $BH_3{\cdot}SMe_2$ in THF were carefully added. After the gas evolution had ceased, the reaction mixture was kept stirring at room temperature for 45 min after which TLC (CH_2Cl_2/hexanes 3:1) indicated full conversion of **1**. Further work-up was carried out under ambient conditions. The volatiles were removed under reduced pressure and the residue was filtered over a plug of silica, yielding pure **1BH$_3$** (74 mg, 80% yield) after solvent removal. Crystals suitable for single crystal X-ray diffraction analysis were obtained by slow evaporation of a solution of **1BH$_3$** in CH_2Cl_2/hexanes.

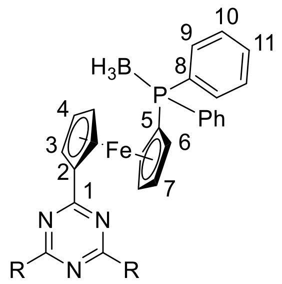

R_f = 0.39 (CH_2Cl_2/hexanes 3:1); m.p. 170 °C (decomp.); ^{1}H NMR (CD_2Cl_2): δ [ppm] = 7.64–7.54 (m, 12H, H9), 7.52–7.46 (m, 6H, H11), 7.45–7.39 (m, 12H, H10), 5.12 (pt, $J_{H,H}$ = 2.0 Hz, 6H, H3), 4.45–4.41 (m, 12H, H4+7), 4.37–4.34 (m, 6H, H6), 1.73–1.00 (br m, 9H, BH); ^{11}B{^{1}H} NMR (CD_2Cl_2): δ [ppm] = −38.2; ^{13}C{^{1}H} NMR (CD_2Cl_2): δ [ppm] = 174.9 (s, C1), 132.5 (d, $^2J_{C,P}$ = 9.7 Hz, C9), 131.05 (d, $^4J_{C,P}$ = 2.5 Hz, C11), 130.98 (d, $^1J_{C,P}$ = 59.2 Hz, C8), 128.5 (d, $^3J_{C,P}$ = 10.1 Hz, C10), 81.3 (s, C2), 74.8 (d, $^2J_{C,P}$ = 7.4 Hz, C6), 74.0 (d, $^3J_{C,P}$ = 9.7 Hz, C7), 73.8 (s, C4), 71.1 (s, C3), 70.3 (d, $^1J_{C,P}$ = 67.1 Hz, C6); ^{31}P{^{1}H} NMR (CD_2Cl_2): δ [ppm] = 15.6 (s, br); IR (KBr): ṽ [cm^{-1}] = 2964 (w), 2924 (w), 2388 (m, B–H), 1515 (vs), 1436 (m), 1383 (m), 1361 (w), 1319 (w), 1262 (w), 1175 (m), 1107 (m), 1061 (w), 1030 (m), 832 (m), 740 (m), 698 (m), 638 (w), 609 (w), 502 (m); UV/Vis (CH_2Cl_2): λ_{max} [nm] (ε [mol^{-1}·L·cm^{-1}]) = 463 (3100), 350 (7500), 288 (43500), 248 (46800); HRMS (ESI): m/z calcd. for $C_{69}H_{64}B_3Fe_3N_3P_3^+$: 1228.2669 [*M*+H$^+$], found: 1228.2683; elemental analysis calcd. [%] for $C_{69}H_{63}B_3Fe_3N_3P_3$: C 67.53, H 5.17, N 3.42; found: C 67.46, H 5.23, N 3.41.

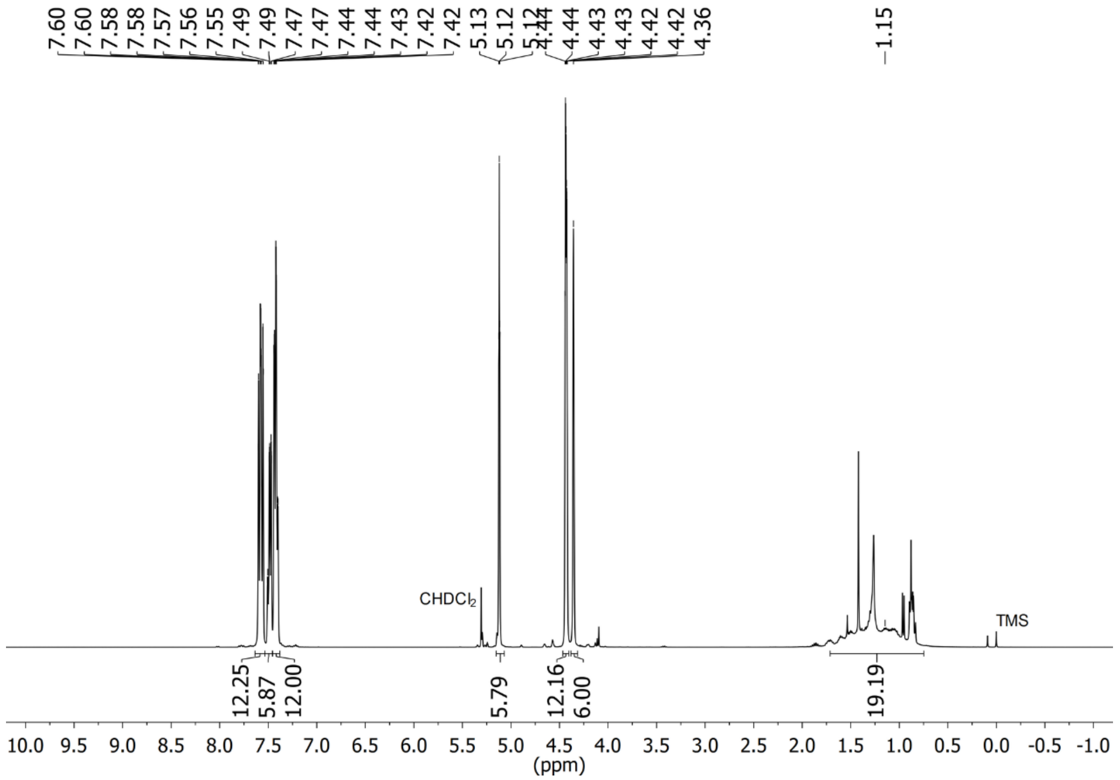

Figure S8. ^{1}H NMR spectrum of **$1BH_3$** in CD_2Cl_2. The integral of the borane region centred around 1.15 pm is overestimated due to NMR solvent impurities.

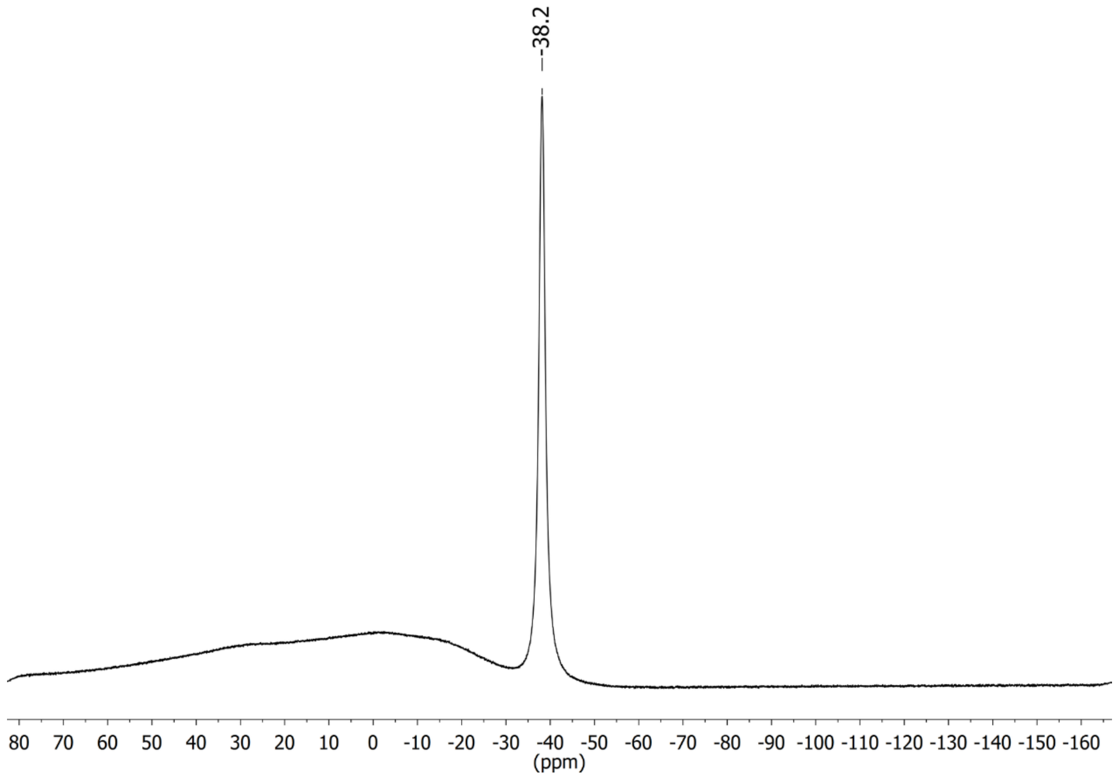

Figure S9. $^{11}B\{^{1}H\}$ NMR spectrum of **$1BH_3$** in CD_2Cl_2.

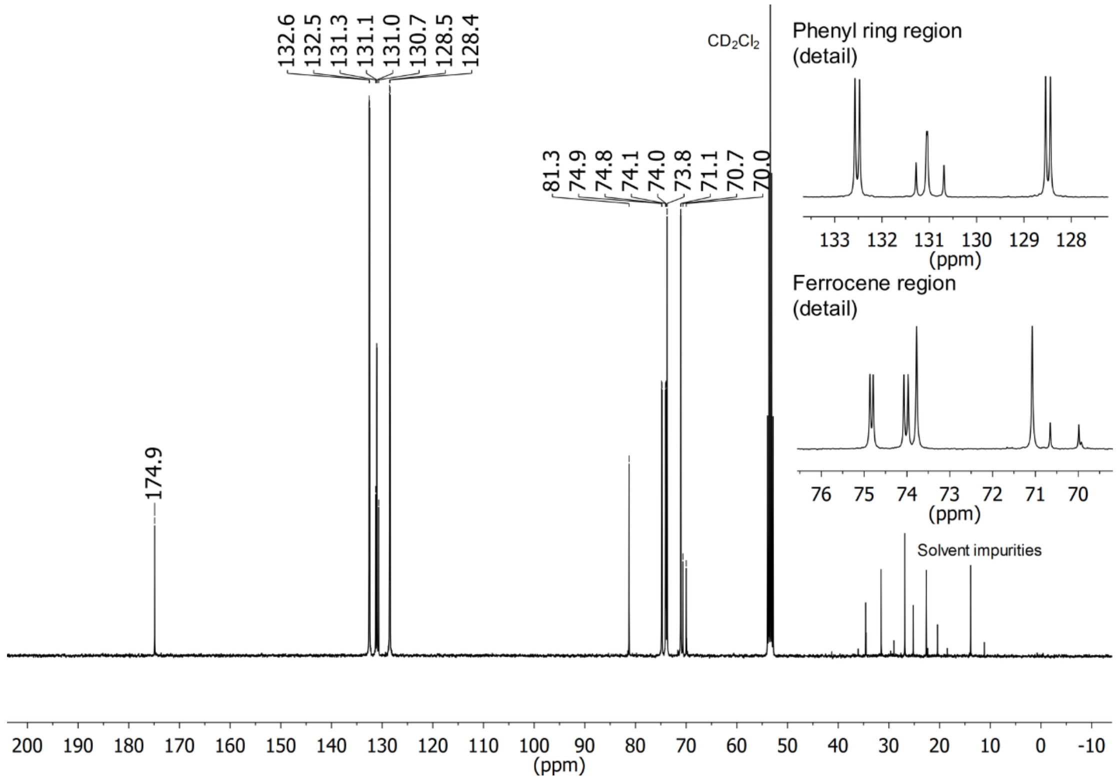

Figure S10. $^{13}C\{^{1}H\}$ NMR spectrum of **$1BH_3$** in CD_2Cl_2, the inserts show details of the phenyl (top right) and the ferrocenyl region (bottom right) of the spectrum.

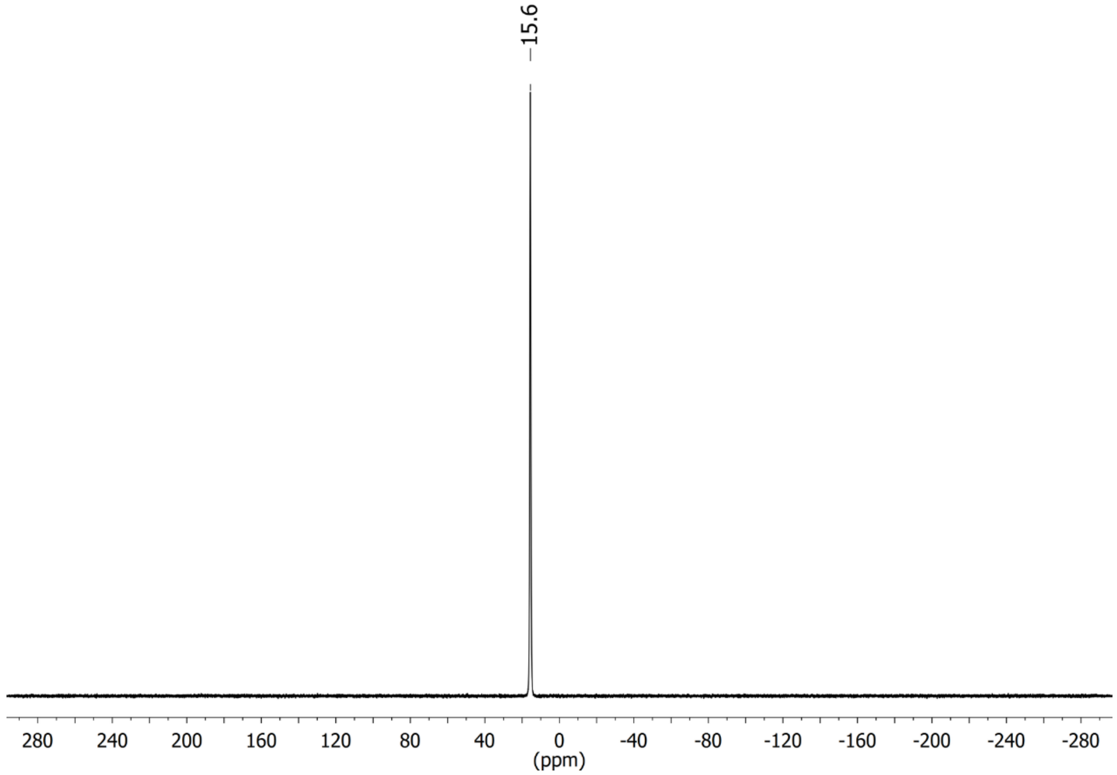

Figure S11. $^{31}P\{^{1}H\}$ NMR spectrum of **$1BH_3$** in CD_2Cl_2.

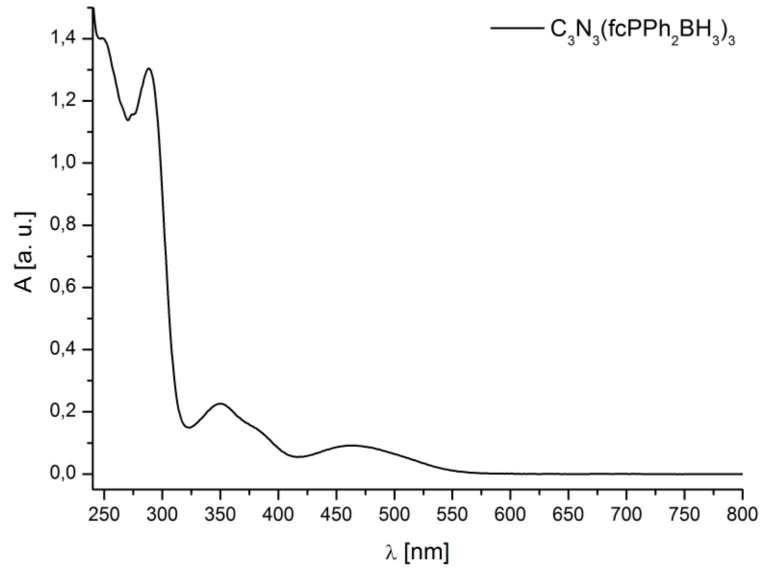

Figure S12. UV/Vis spectrum of **$1BH_3$** in CH_2Cl_2.

1.5. Synthesis of {2,4,6-tris(1-diphenylphosphanyl-1'-ferrocenylene)-1,3,5-triazine-κP^3} copper(I) triflate (1Cu)

Under stirring, a solution of 41.5 mg (110 µmol, 1.00 eq.) $[Cu(MeCN)_4]OTf$ in 12 mL CH_2Cl_2 was added to 150 mg (127 µmol, 1.15 eq.) **1** and kept stirring at room temperature for 1 hour. The reaction mixture was filtered *via* cannula, layered with 30 mL toluene and kept at 7 °C for 2 days. The so-obtained crystalline material, suitable for single crystal X-ray diffraction analysis, was isolated by filtration and dried *in vacuo* overnight at 40 °C, causing the deep red crystals to brittle, yielding a red powder (124 mg, 81% yield) which can be handled in air but which was stored under nitrogen.

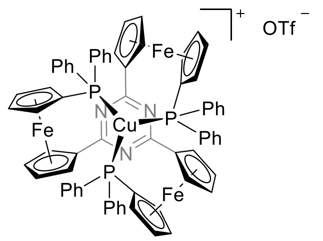

Cu_{cl}

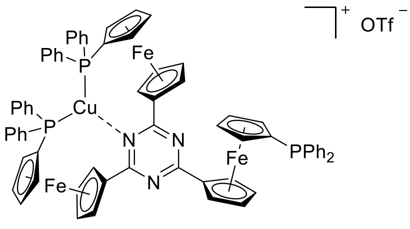

Cu_{op}

m.p. 197–198 °C; ^1H NMR (CD_2Cl_2): $\delta_{25\,°C}$ [ppm] = 7.68–7.10 (m, 30H, $P(C_6H_5)_2$), 5.50–5.22 (m, 6H, fcH), 4.67 (s, 6H, fcH), 4.46 (s (br), 6H, fcH), 4.00 (s (br), 6H, fcH); $\delta_{-80\,°C}$ [ppm][1] **Cu_{cl}** = 9.60 (m, 3H, $P(C_6H_5)_2$), 8.06 (pt, $J_{H,H}$ = 7.6 Hz, 3H, $P(C_6H_5)_2$), 7.68 (pt, $J_{H,H}$ = = 7.5 Hz, 3H, $P(C_6H_5)_2$), 7.31–7.18 (m, 9H, $P(C_6H_5)_2$), 6.38 (m, 3H, $P(C_6H_5)_2$), 6.30 (pt, $J_{H,H}$ = 7.6 Hz, 3H, $P(C_6H_5)_2$), 6.12 (m, 3H, $P(C_6H_5)_2$), 5.66 (m, 3H, $P(C_6H_5)_2$), 5.52 (s, 3H, fcH), 5.29 (m, 6H, fcH), 4.76 (s, 3H, fcH), 4.63 (s, 3H, fcH), 4.19 (m, 6H, fcH), 3.09 (s, 3H, fcH), 3.04 (s, 3H, fcH); **Cu_{op}** = 7.66–7.52 (m, 12H, $P(C_6H_5)_2$), 7.49–7.43 (m, 6H, $P(C_6H_5)_2$), 7.41–7.26 (m, 12H, $P(C_6H_5)_2$), 5.66 (s, 1H, fcH), 5.56 (m, 2H, fcH), 5.38 (m, 1H, fcH), 5.26 (s, 1H, fcH), 4.91 (s, 1H, fcH), 4.83 (s, 1H, fcH), 4.80 (s, 1H, fcH), 4.76 (s, 1H, fcH), 4.73 (m, 1H, fcH), 4.70 (s, 1H, fcH), 4.64 (s, 1H, fcH), 4.49 (s, 1H, fcH), 4.46 (s, 1H, fcH), 4.41 (s, 1H, fcH), 4.29 (m, 3H, fcH), 4.23 (s, 1H, fcH), 4.15 (s, 1H, fcH), 4.03 (s, 1H, fcH), 3.99 (s, 1H, fcH), 3.91 (s, 1H, fcH), 3.88 (s, 1H, fcH); ^1H NMR (CD_3CN): $\delta_{25\,°C}$ [ppm] = 7.43–7.37 (m, 6H, P(*p*-$C_6H_5)_2$), 7.36–7.24 (m, 24H, $P(C_6H_5)_2$), 5.32 (s (br), 6H, fc-H), 4.45 (s, 6H, fc-H), 4.43 (s, 6H, fc-H), 4.03 (s, 6H, fc-H); $^{13}C\{^1H\}$ NMR (CD_2Cl_2): $\delta_{25\,°C}$ [ppm] = 174.9 (m, C_3N_3), 137.7 (m, $P(C_6H_5)_2$), 132.8 (m, $P(C_6H_5)_2$), 131.1 m, $P(C_6H_5)_2$), 129.2 (m, m, $P(C_6H_5)_2$), 128.4 (m, m, $P(C_6H_5)_2$), 78.9 (m, fc-C), 74.9 (m, fc-C), 74.3 (m, fc-C), 73.4 (m, fc-C), 73.0 (m, fc-C), 72.3 (m, fc-C); $^{13}C\{^{31}P,^1H\}$ NMR (CD_3CN): $\delta_{25\,°C}$ [ppm] = 175.3 (s, C_3N_3), 133.0 (s, $P(C_6H_5)_2$), 129.8 (s, P(*p*-$C_6H_5)_2$), 128.6 (s, $P(C_6H_5)_2$), 116.3 (s (br), P(*i*-$C_6H_5)_2$), 80.5 (s, C_3N_3–C_{Cp}), 74.8 (s, fc-C), 73.9 (s, P-C_{Cp}), 73.6 (s, fc-C), 73.3 (s, fc-C), 71.2 (s, fc-C); $^{19}F\{^1H\}$ NMR (CD_2Cl_2): $\delta_{25\,°C}$ [ppm] = −78.9 (s, O_3SCF_3); $^{31}P\{^1H\}$ NMR (CD_2Cl_2): $\delta_{25\,°C}$ [ppm] = −7.3 (s, 2P, $P(Ph)_2$–Cu), −20.6 (s, 1P, $P(Ph)_2$); $\delta_{-80\,°C}$ [ppm] = −7.9 (m, $^2J_{P,P}$ = 122 Hz, 2P, $P(Ph)_2$–Cu, **Cu_{op}**), −14.2 (s, 3P, $P(Ph)_2$–Cu, **Cu_{cl}**), −23.5 (s, 1P, $P(Ph)_2$, **Cu_{op}**); $^{31}P\{^1H\}$ NMR (CD_3CN): $\delta_{25\,°C}$ [ppm] = −12.7 (s (br), 3P); IR (KBr): $\tilde{\nu}$ [cm^{-1}] = 2964 (w), 2925 (w), 1721 (w), 1512 (s), 1486 (m), 1436 (w), 1319 (w), 1263 (m), 1163 (m), 1096 (m), 1031 (m), 831 (w), 750 (m), 697 (m), 637 (w), 504 (m); UV/Vis (CH_2Cl_2): λ_{max} [nm] (ε [$mol^{-1}\cdot L\cdot cm^{-1}$]) = 508 (4300), 348 (8400), 290 (36200), 249 (47400);

[1] For the sake of clarity, the two overlapping spectra for the C_3-symmetric form **$1Cu_{cl}$** and for the C_1-symmetric open form **$1Cu_{op}$** are presented one after the other. For **Cu_{op}** (Figure S16) not all of the 24 expected proton signals can be unambiguously identified, since there is considerable overlap with signals attributable to **$1Cu_{cl}$** (Figure S15).

HRMS (ESI): m/z calcd. for $C_{69}H_{54}CuFe_3N_3P_3^+$: 1248.0878 [*M*−OTf]$^+$, found: 1248.0894; elemental analysis calcd. [%] for $C_{70}H_{54}CuF_3Fe_3N_3O_3P_3S$: C 60.13, H 3.89, N 3.01; found: C 60.62, H 3.95, N 2.92[2].

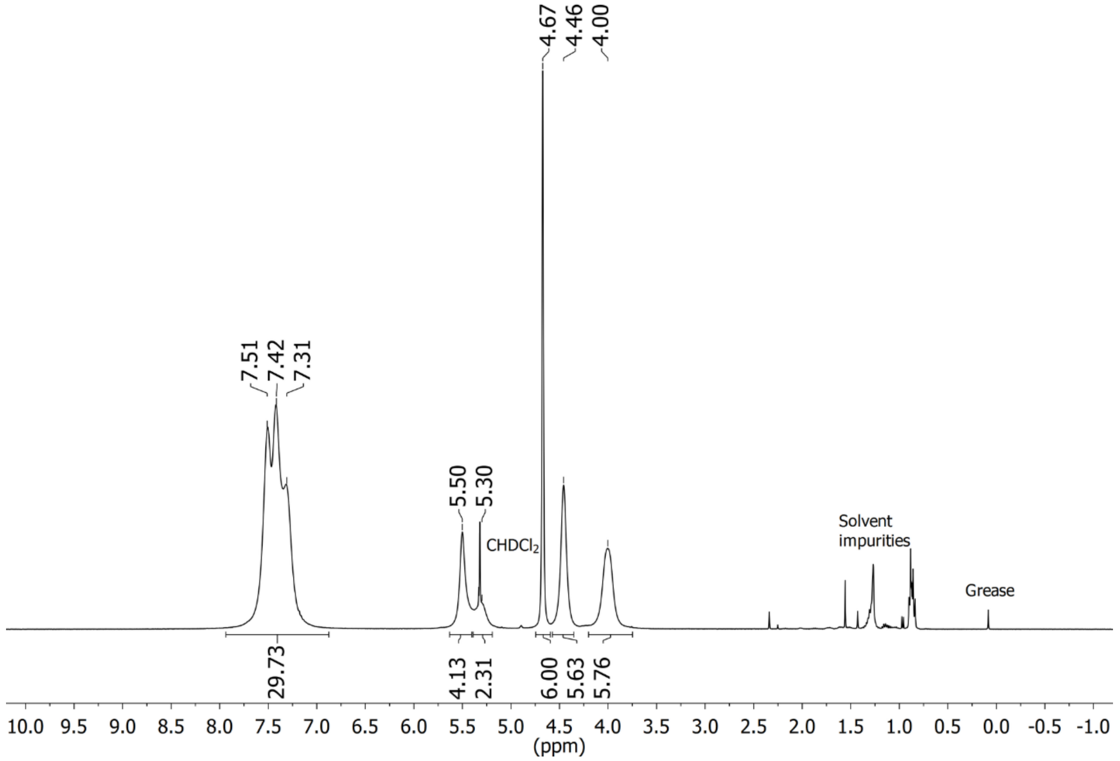

Figure S13. ^{1}H NMR spectrum of **1Cu** in CD_2Cl_2 recorded at 25 °C.

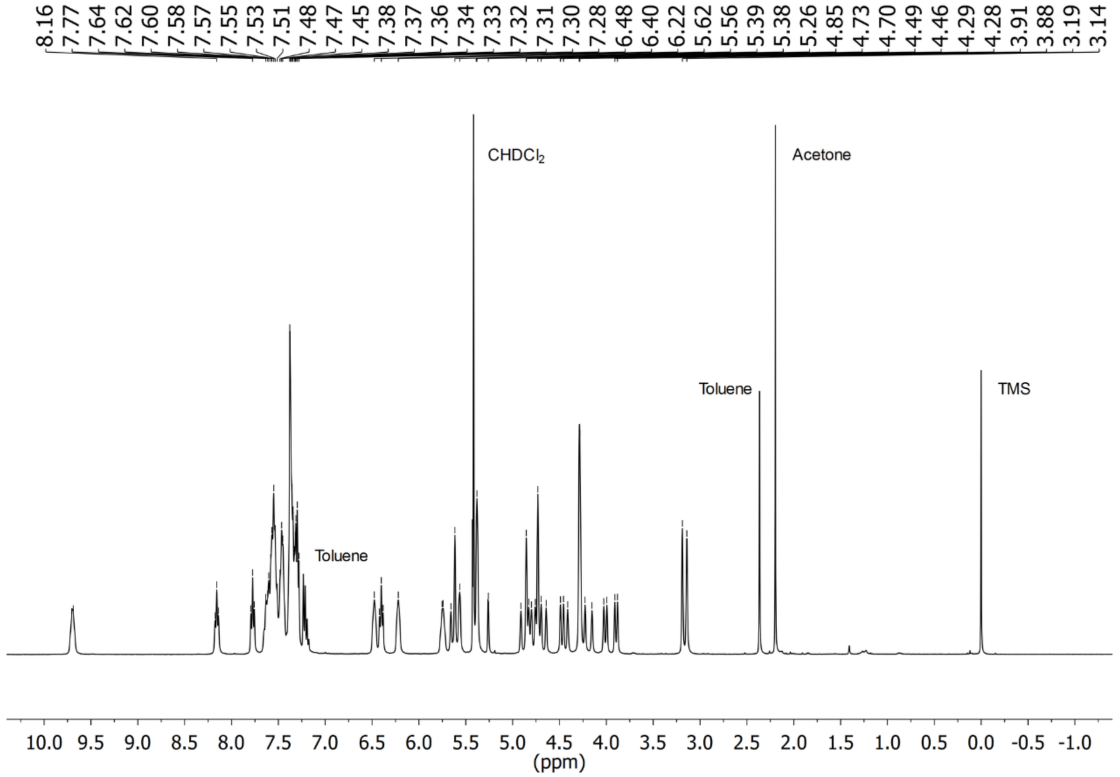

Figure S14. ^{1}H NMR spectrum of **1Cu** in CD_2Cl_2 recorded at −80 °C, displaying the equilibrium between **Cu$_{cl}$** (Figure S15) and **Cu$_{op}$** (Figure S16).

---

[2] Even after prolonged drying *in vacuo*, residual toluene and/or grease might account for the deviations.

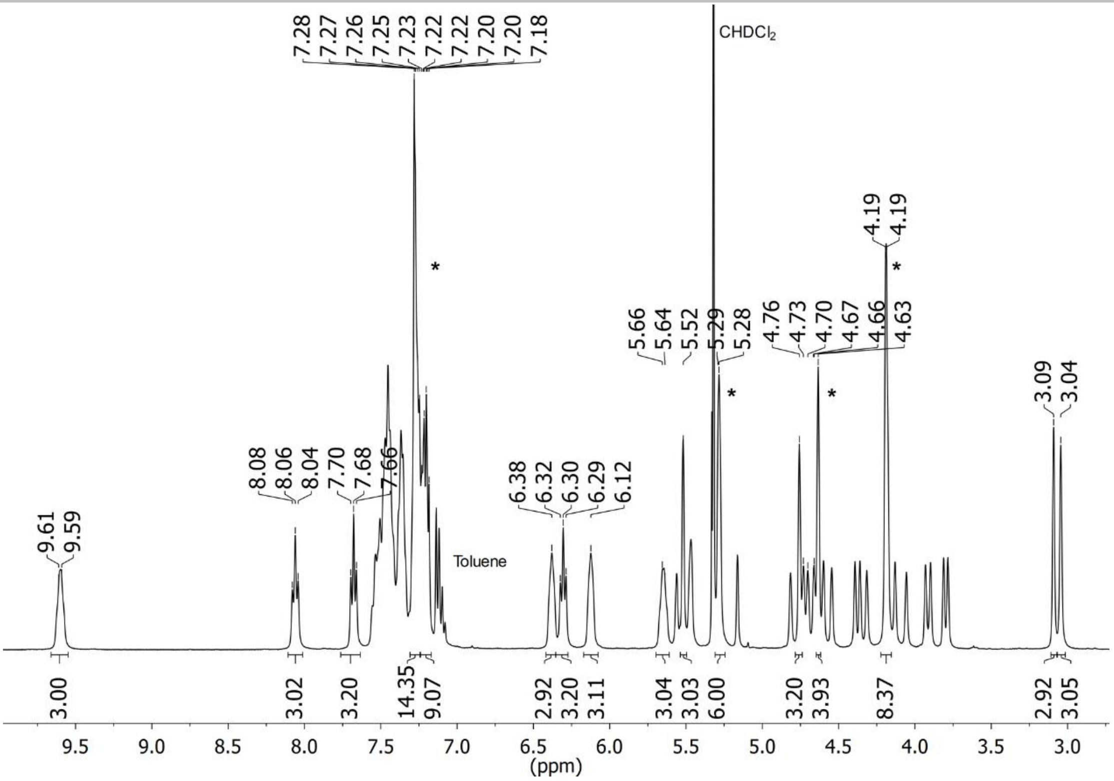

Figure S15. Detail of the ^{1}H NMR spectrum of **1Cu** in CD_2Cl_2 recorded at −80 °C. Only peaks attributable to **Cu$_{cl}$** are labelled. Signals marked with an asterisk * denote an overlap with signals arising from **1Cu$_{op}$**, thus their integrals are overestimated.

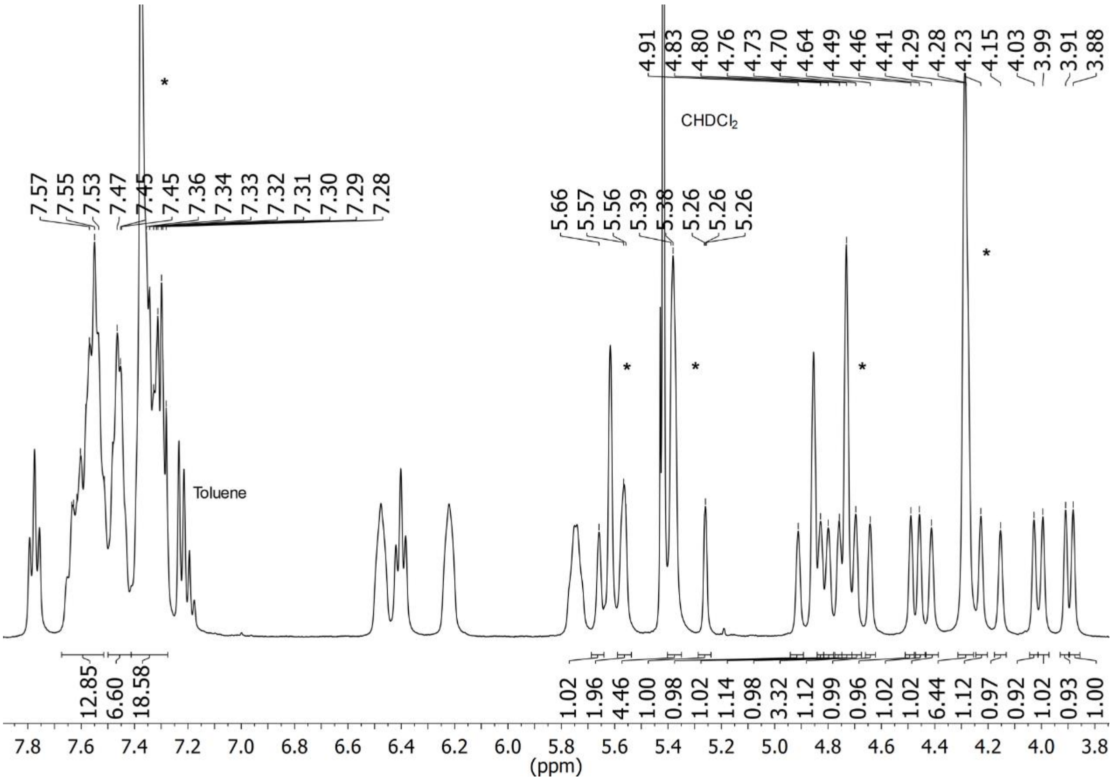

Figure S16. Detail of the ^{1}H NMR spectrum of **1Cu** in CD_2Cl_2 recorded at −80 °C. Only peaks attributable to **Cu$_{op}$** are labelled. Signals marked with an asterisk * denote an overlap with signals arising from **1Cu$_{cl}$**, thus their integrals are overestimated.

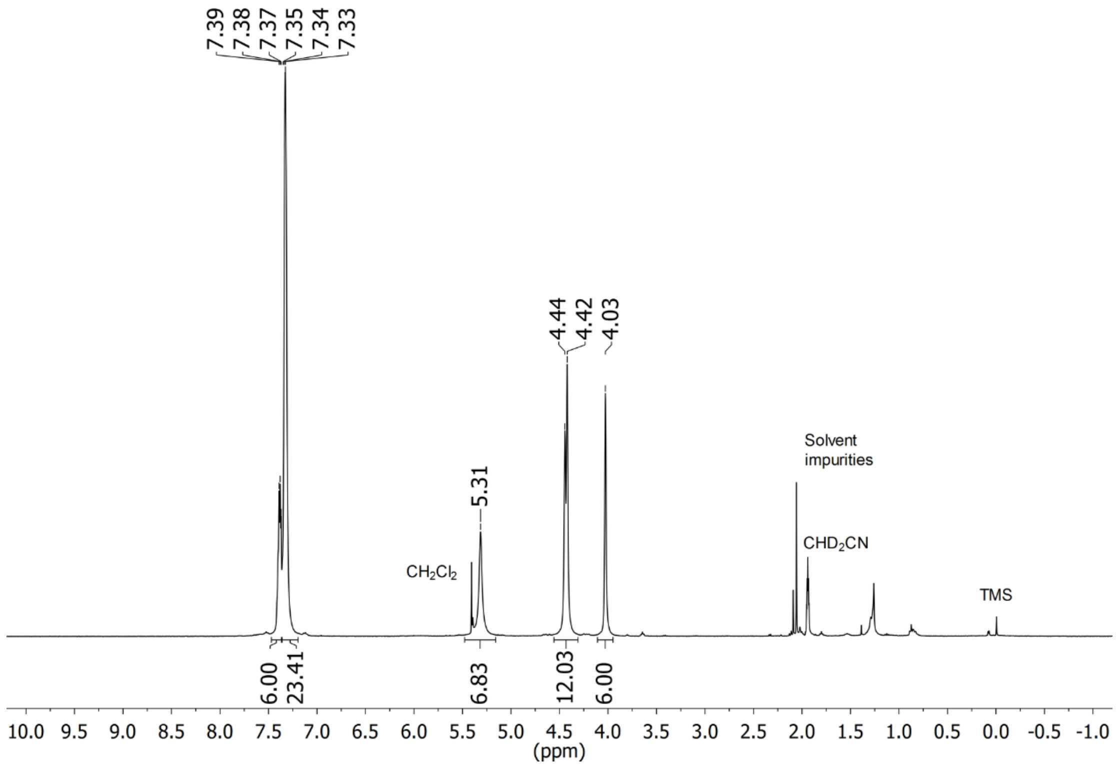

Figure S17. ^{1}H NMR spectrum of **1Cu** in CD_3CN recorded at 25 °C.

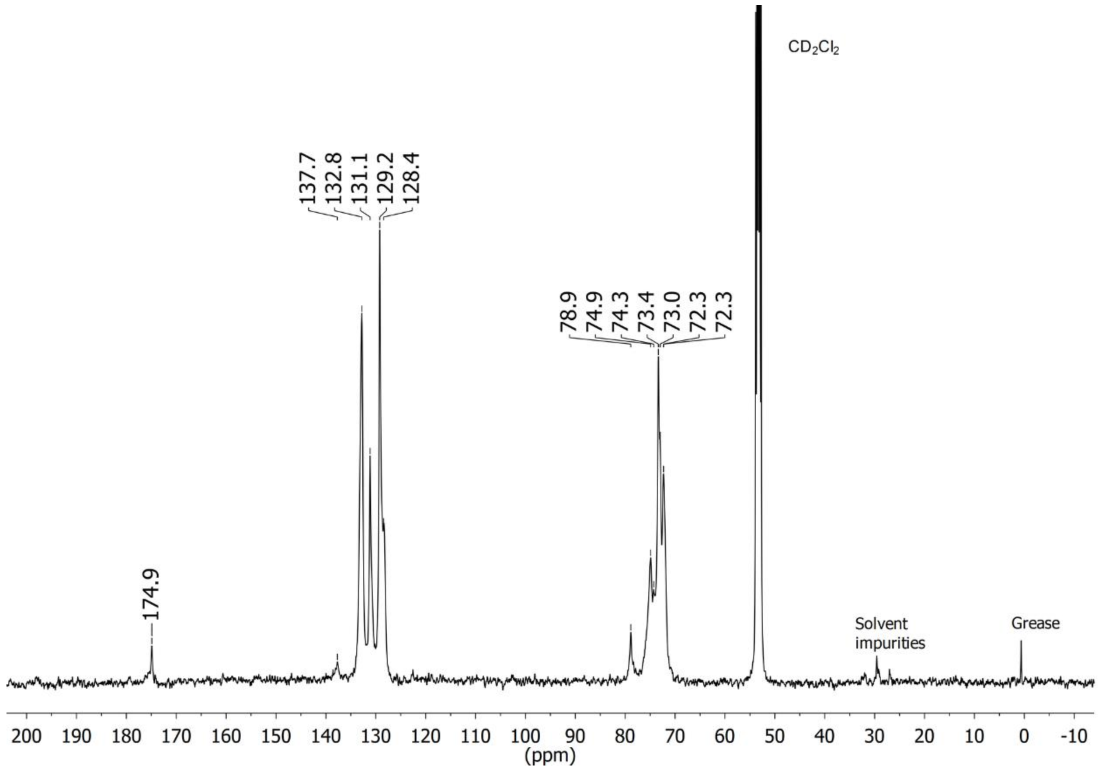

Figure S18. $^{13}C\{^{1}H\}$ NMR spectrum of **1Cu** in CD_2Cl_2 recorded at 25 °C using the z_gp_g pulse sequence for fast-relaxing nuclei (NS = 14400, 18 h).

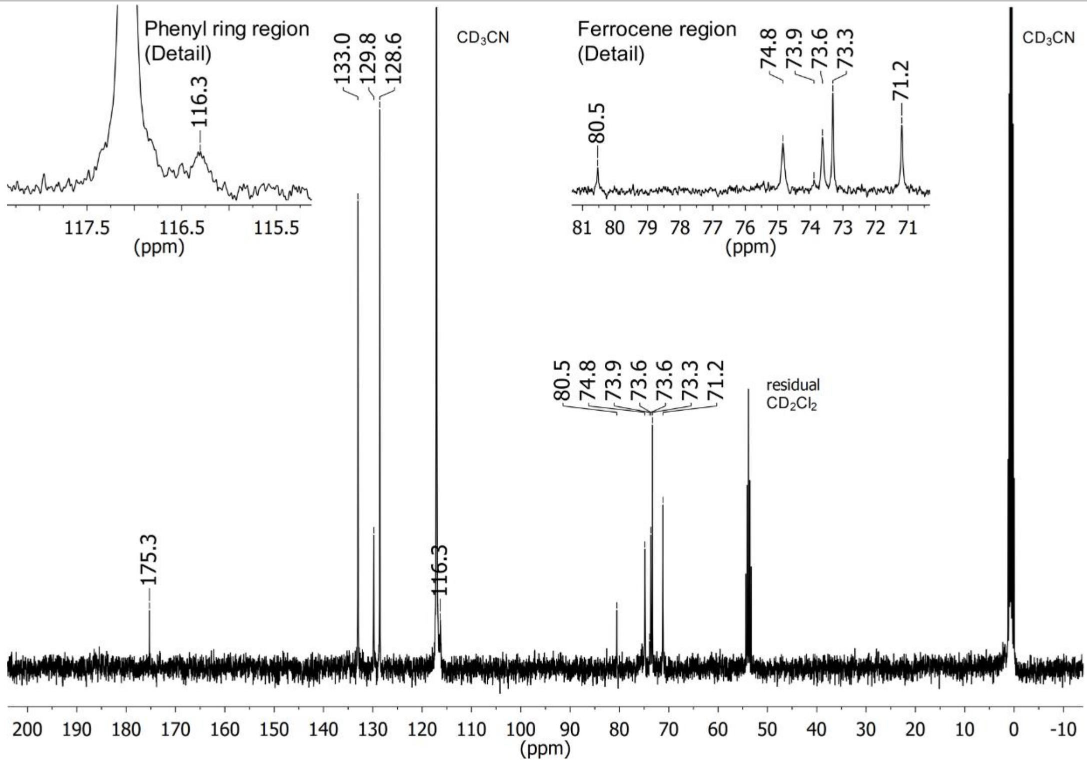

Figure S19. $^{13}C\{^{31}P,^{1}H\}$ NMR spectrum of **1Cu** in CD_3CN recorded at 25 °C (NS = 8192, 10 h), the inserts show details of the phenyl (left) and the ferrocenyl region (right) of the spectrum.

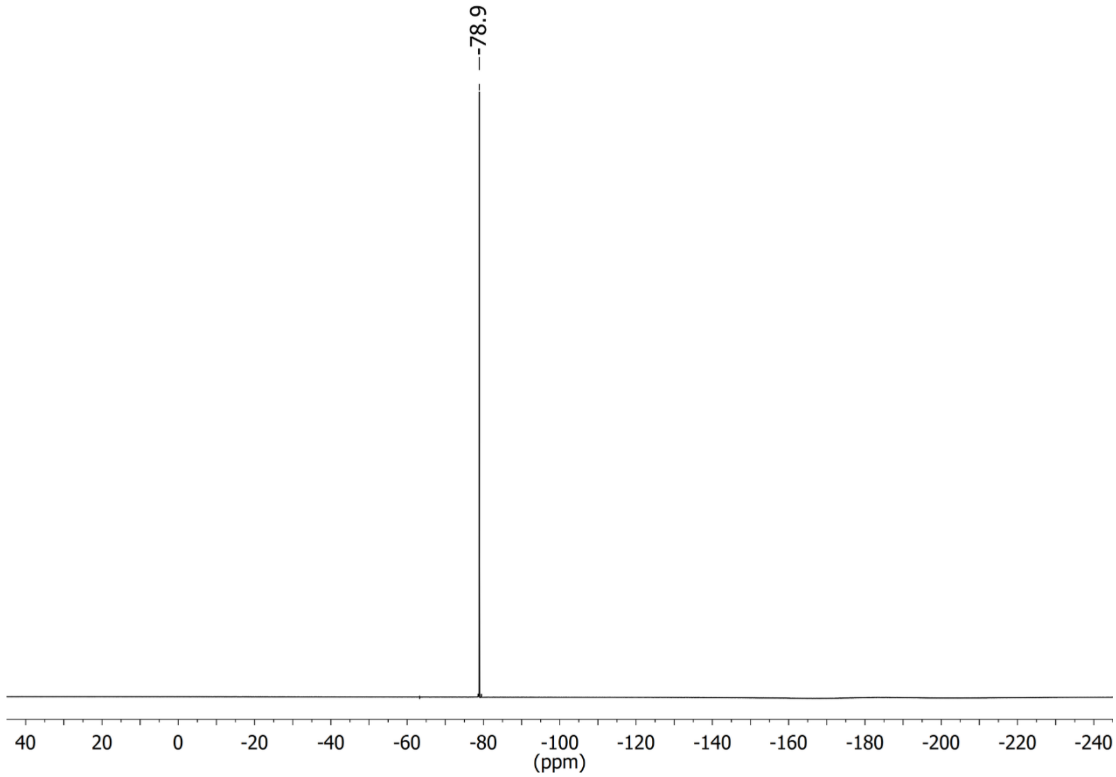

Figure S20. $^{19}F\{^{1}H\}$ NMR spectrum of **1Cu** in CD_2Cl_2 recorded at 25 °C.

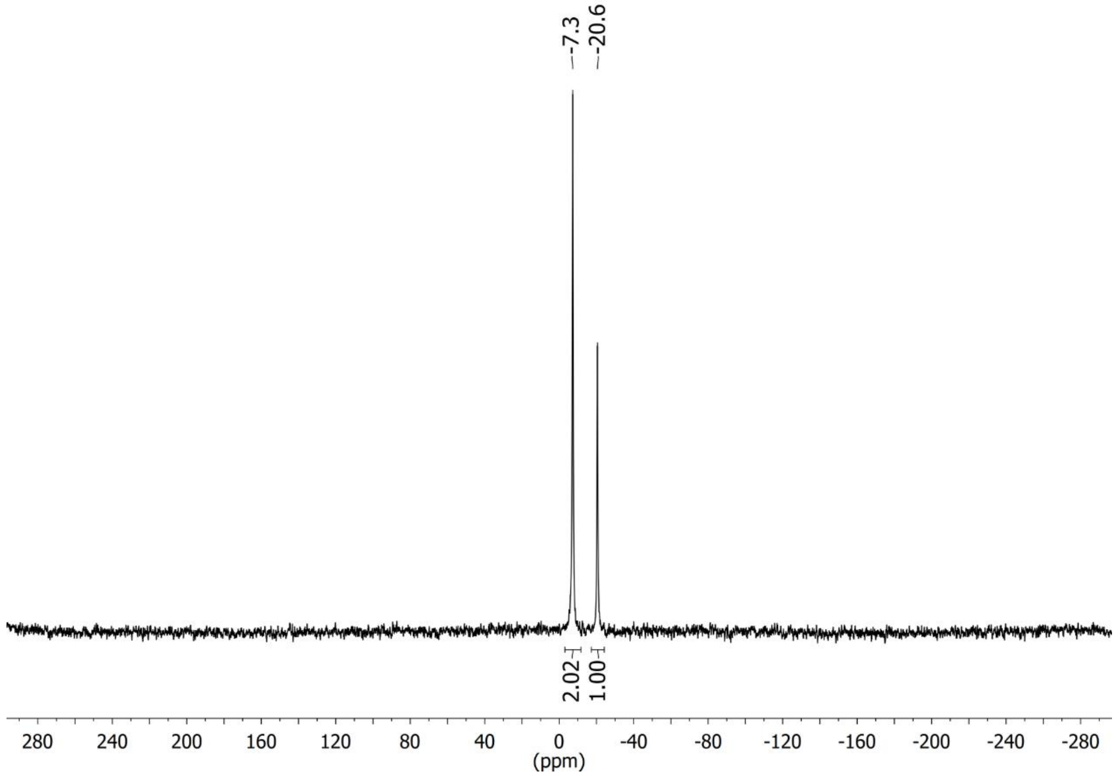

Figure S21. $^{31}P\{^{1}H\}$ NMR spectrum of **1Cu** in CD_2Cl_2 recorded at 25 °C (exponential lb = 20 Hz)

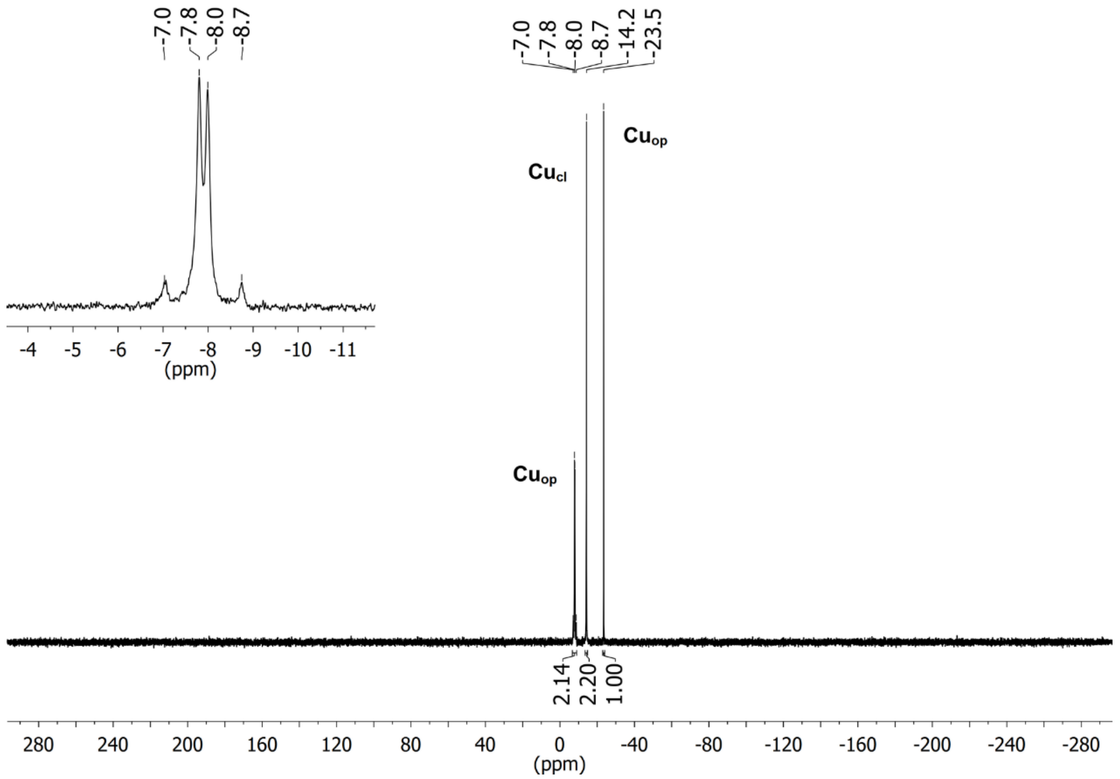

Figure S22. $^{31}P\{^{1}H\}$ NMR spectrum of **1Cu** in CD_2Cl_2 recorded at −80 °C, displaying signals for both the C_3-symmetric **Cu$_{cl}$** and the C_1-symmetric **Cu$_{op}$** isomers. The expansion displays the Cu-mediated $^{2}J_{P,P}$ coupling of **Cu$_{op}$** resulting in the multiplet of an AA' spin system in detail.

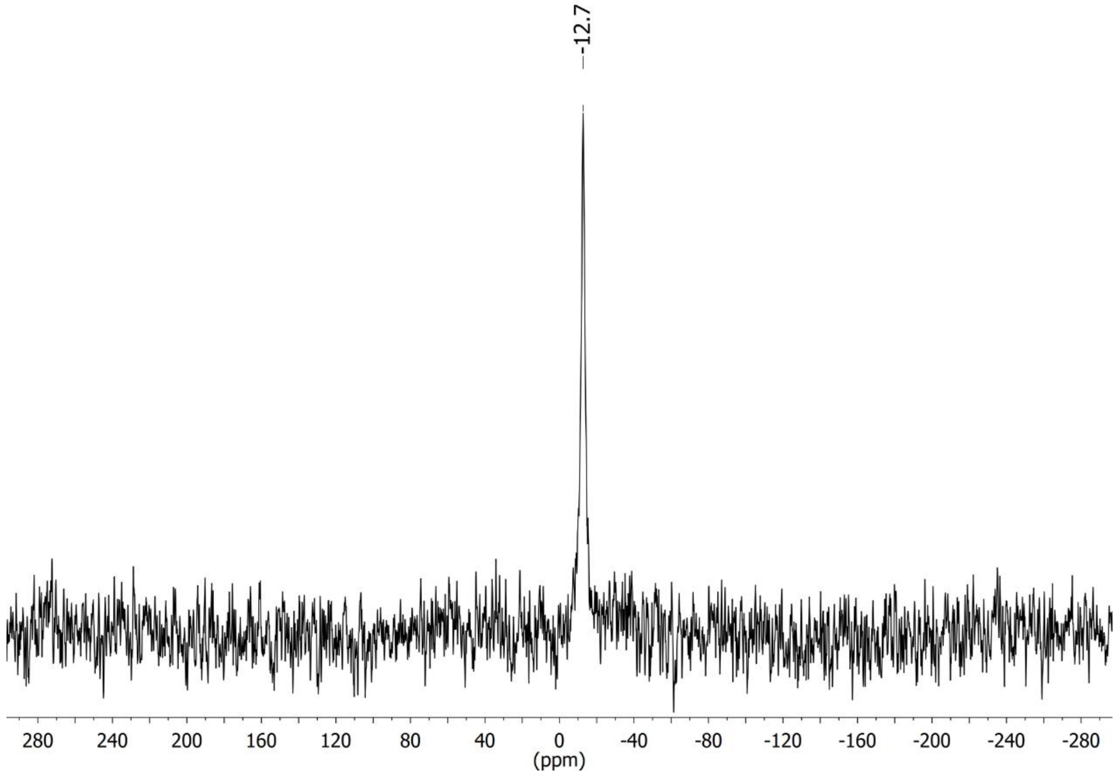

Figure S23. $^{31}P\{^1H\}$ NMR spectrum of **1Cu** in CD_3CN recorded at 25 °C (exponential lb = 50 Hz)

1.6. Synthesis of {2,4,6-tris(1-diphenylphosphanyl-1'-ferrocenylene)-1,3,5-triazine-κP³} copper(I) tetrafluoroborate (1CuBF₄)

Under stirring, a solution of 23.1 mg (73.3 µmol, 1.00 eq.) $[Cu(MeCN)_4]BF_4$ in 8 mL CH_2Cl_2 was added to 100 mg (84.3 µmol, 1.15 eq.) **1** and kept stirring at room temperature for 1 hour. The reaction mixture was filtered *via* cannula, concentrated to half its original volume, layered with 12 mL toluene and kept at 7 °C for 2 days. The so-obtained crystalline material, suitable for single crystal X-ray diffraction analysis, was isolated by filtration and dried *in vacuo* overnight at 40 °C, causing the deep red crystals to brittle, yielding a red powder (75 mg, 77% yield) which can be handled in air, but which was stored under nitrogen.

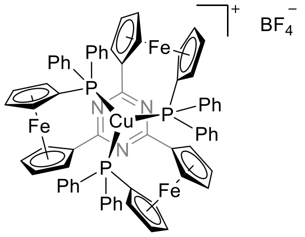

m.p. 200–202 °C; ^1H NMR (CD_2Cl_2): $\delta_{25\,°C}$ [ppm] = 7.77–7.12 (m, 30H, $P(C_6H_5)_2$), 5.63–5.20 (m, 6H, fcH), 4.67 (s, 6H, fcH), 4.45 (s (br), 6H, fcH), 4.00 (s (br), 6H); $^{13}C\{^1H\}$ NMR (CD_2Cl_2): $\delta_{25\,°C}$ [ppm] = 176.3–175.0 (m, C_3N_3), 133.2 (s (br), $P(C_6H_5)_2$), 131.3 (s (br), $P(C_6H_5)_2$), 129.9–128.2 (m, $P(C_6H_5)_2$), 79.9–78.2 (m, fc-C), 75.6–71.3 (m, fc-C); $^{19}F\{^1H\}$ NMR (CD_2Cl_2): $\delta_{25\,°C}$ [ppm] = −153.4 (s, $^{10}BF_4$), −153.5 (s, $^{11}BF_4$); $^{31}P\{^1H\}$ NMR (CD_2Cl_2): $\delta_{25\,°C}$ [ppm] = −7.3 (s, 2P, $P(Ph)_2$–Cu), −20.5 (s, 1P, $P(Ph)_2$); IR (KBr): $\tilde{\nu}$ [cm^{-1}] = 2965 (w), 2924 (w), 1512 (s), 1485 (m), 1436 (w), 1320 (w), 1262 (w), 1166 (w), 1123 (w), 1084 (m), 1031 (m), 831 (w), 747 (m), 697 (m), 503 (m); UV/Vis (CH_2Cl_2): λ_{max} [nm] (ε [$mol^{-1}\cdot L\cdot cm^{-1}$]) = 512 (4400), 349 (8800), 290 (36200), 249 (47300); HRMS (ESI): m/z calcd. for $C_{69}H_{54}CuFe_3N_3P_3^+$: 1248.0878 $[M{-}BF_4]^+$, found: 1248.0842; elemental analysis calcd. [%] for $C_{69}H_{54}BCuF_4Fe_3N_3P_3$: C 62.03, H 4.07, N 3.15; found: C 62.54, H 4.10, N 3.04.

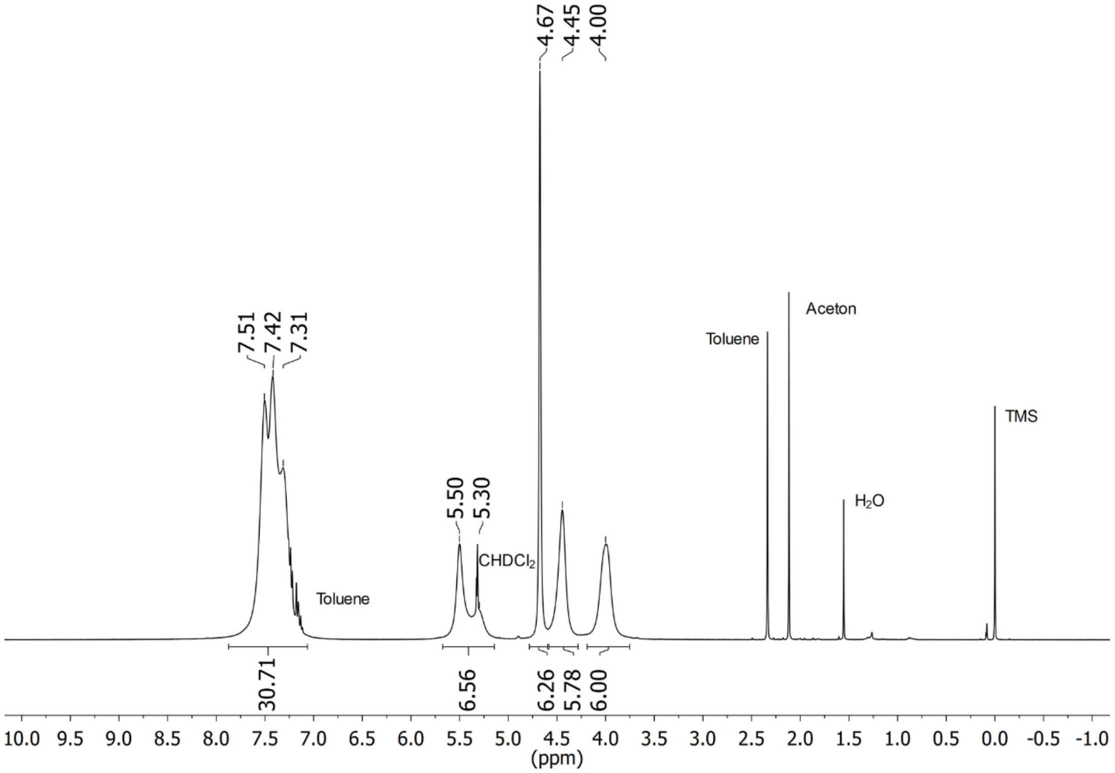

Figure S24. ^{1}H NMR spectrum of **1CuBF$_4$** in CD_2Cl_2.

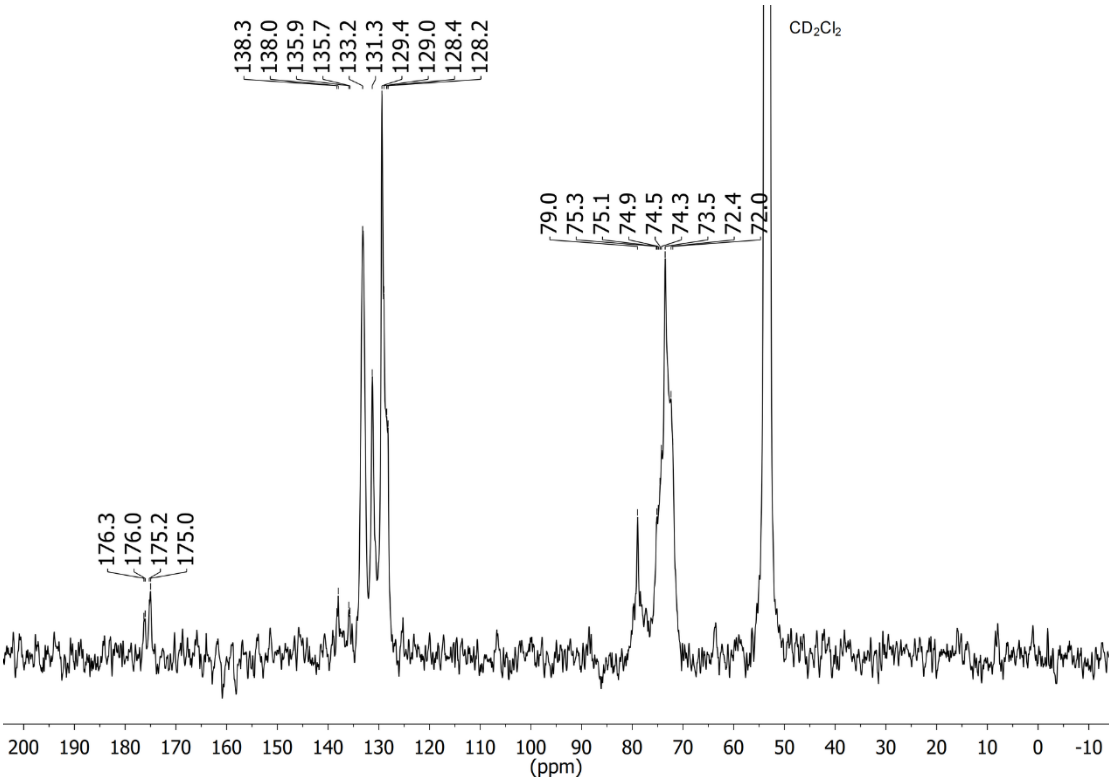

Figure S25. $^{13}C\{^{1}H\}$ NMR spectrum of **1CuBF$_4$** in CD_2Cl_2 (exponential lb = 20 Hz).

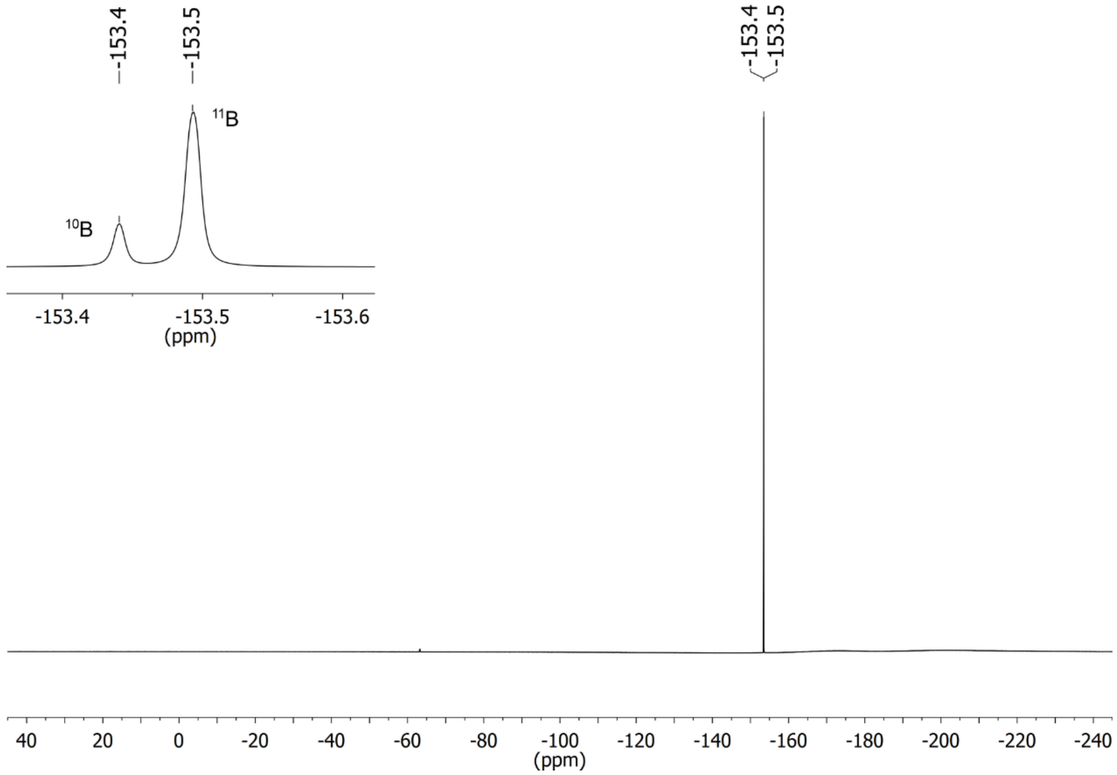

Figure S26. $^{19}F\{^{1}H\}$ NMR spectrum of **1CuBF$_4$** in CD_2Cl_2, the expansion showing the two isotopomeric ^{19}F shifts due to $^{10/11}B$ in detail.

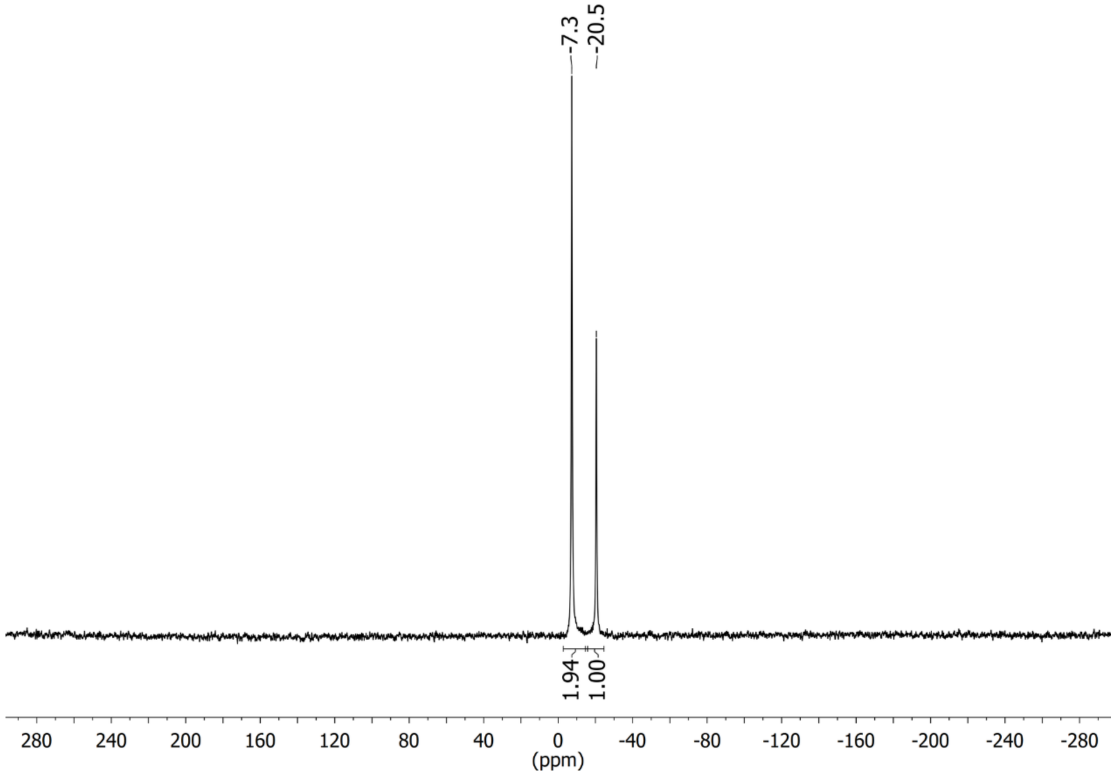

Figure S27. $^{31}P\{^{1}H\}$ NMR spectrum of **1CuBF$_4$** in CD_2Cl_2 (exponential lb = 20 Hz).

1.7. Synthesis of {2,4,6-tris(1-diphenylphosphanyl-1'-ferrocenylene)-1,3,5-triazine-κP³} copper(I) tetrakis{3,5-bis(trifluoromethyl)phenyl}borate (1CuBArF4)

In a Schlenk flask, 15 mg (11 µmol, 1.0 eq.) **1CuBF4** and 48 mg NaBAr$^F_4$ (56 µmol, 5.0 eq.) were dissolved in 2 mL CH_2Cl_2 and stirred for 30 min at room temperature. The deep red solution was cooled to −20 °C (ice/NaCl 3:1) and subsequently filtered from the colourless solid. After removal of the volatiles, the solidified red oil was dried *in vacuo* and immediately used for VT NMR studies, thus no yield was determined. Crystals suitable for single crystal X-ray diffraction analysis were obtained from layering a CH_2Cl_2 solution of **1CuBArF4** with hexanes and storing it at 7 °C overnight.

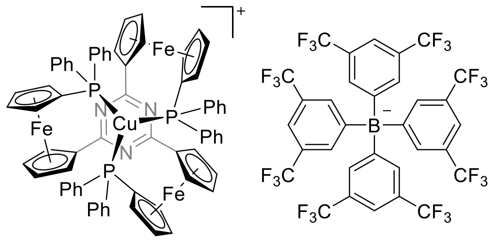

^{1}H NMR (CD_2Cl_2): $\delta_{25\,°C}$ [ppm] = 7.75–7.69 (m, 8H, B–C_6(H)(***o*-H**)$_2$(CF_3)$_2$), 7.57–7.54 (m, 4H, B–C_6(***p*-H**)H_2(CF_3)$_2$), 7.65–7.10 (m, 30H, P(C_6H_5)$_2$),), 5.80–5.21 (m, 6H, fcH), 4.65 (pt, $J_{H,H}$ = 2.0 Hz, 6H, fcH), 4.43 (s (br), 6H, fcH), 3.99 (s (br), 6H, fcH).; ^{11}B{^{1}H} NMR (CD_2Cl_2): $\delta_{25\,°C}$ [ppm] = −6.7 (s, BAr_4); ^{19}F{^{1}H} NMR (CD_2Cl_2): $\delta_{25\,°C}$ [ppm] = −62.9 (s, CF_3); ^{31}P{^{1}H} NMR (CD_2Cl_2): $\delta_{25\,°C}$ [ppm] = −7.2 (s, 2P, P(Ph)$_2$–Cu), −20.6 (s, 1P, P(Ph)$_2$); HRMS (ESI): m/z calcd. for $C_{69}H_{54}CuFe_3N_3P_3^+$: 1248.0878 [*M*−BAr$^F_4$]$^+$, found: 1248.0886.

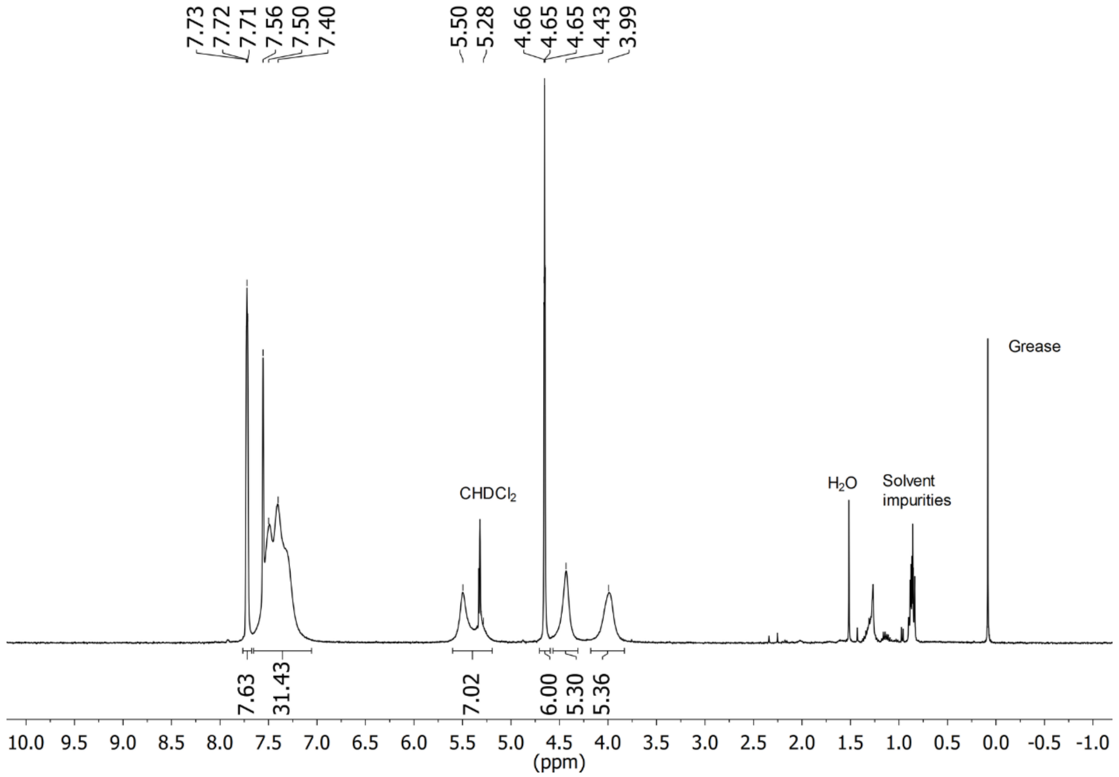

Figure S28. ^{1}H NMR spectrum of **1CuBArF4** in CD_2Cl_2.

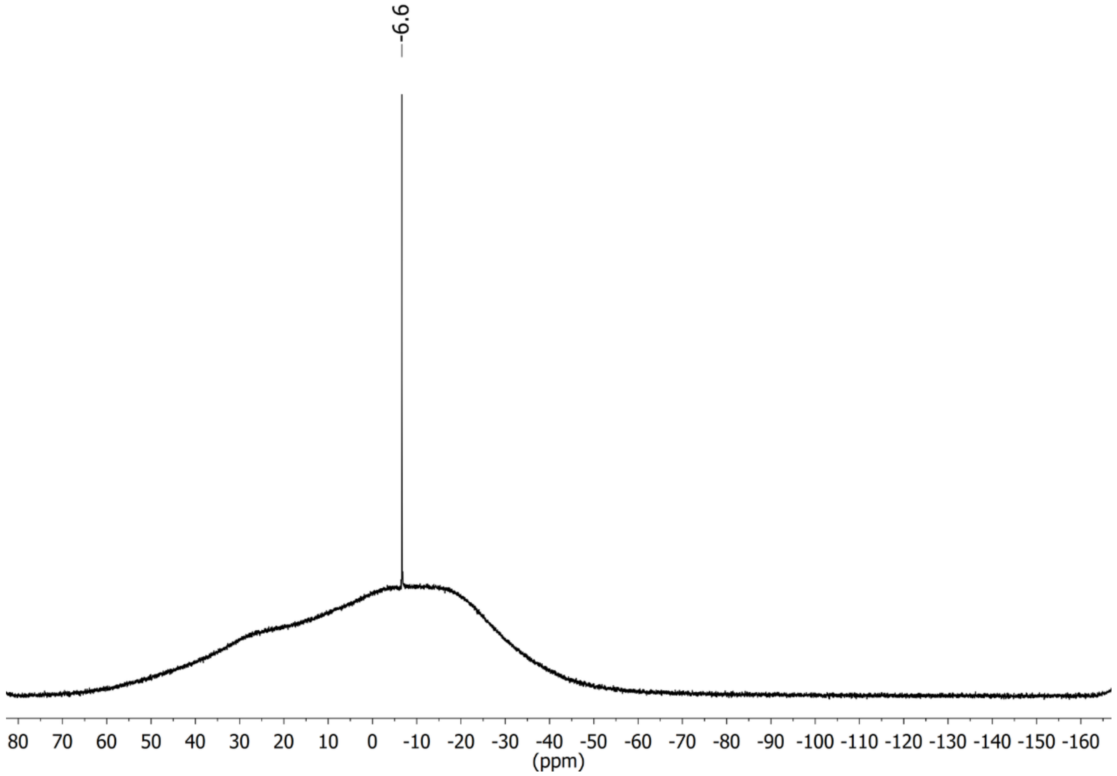

Figure S29. $^{11}B\{^{1}H\}$ NMR spectrum of **1CuBAr$^{F}_{4}$** in CD_2Cl_2.

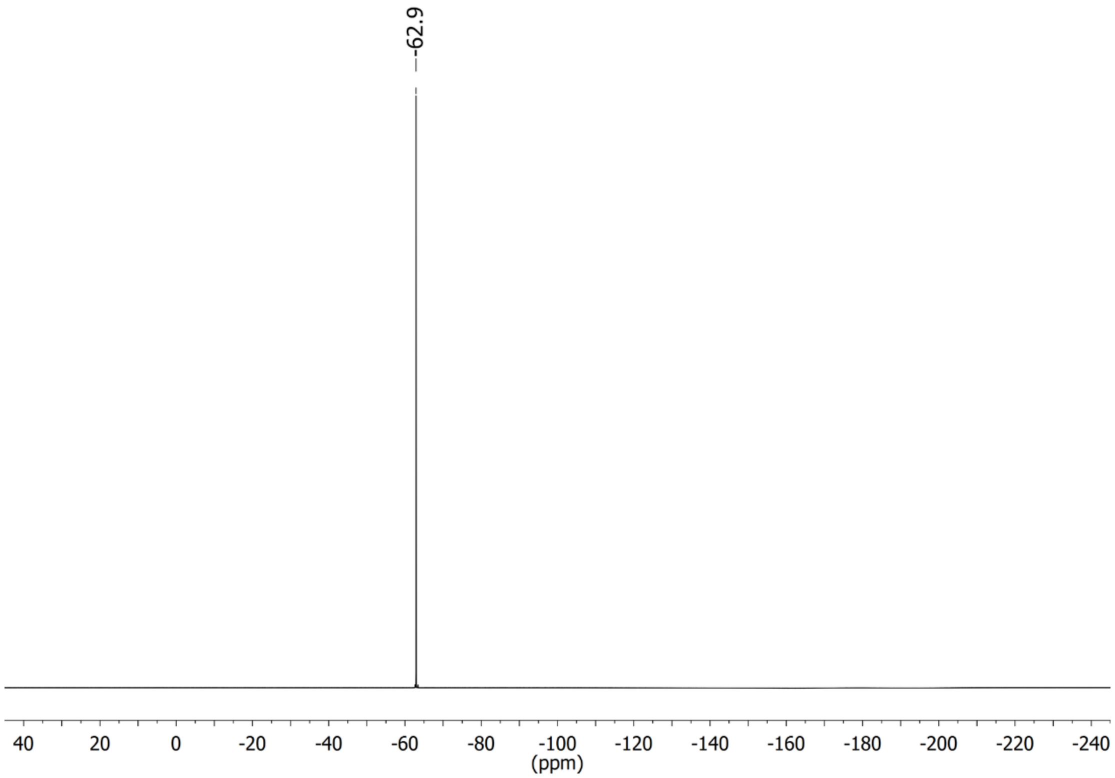

Figure S30. $^{19}F\{^{1}H\}$ NMR spectrum of **1CuBAr$^{F}_{4}$** in CD_2Cl_2.

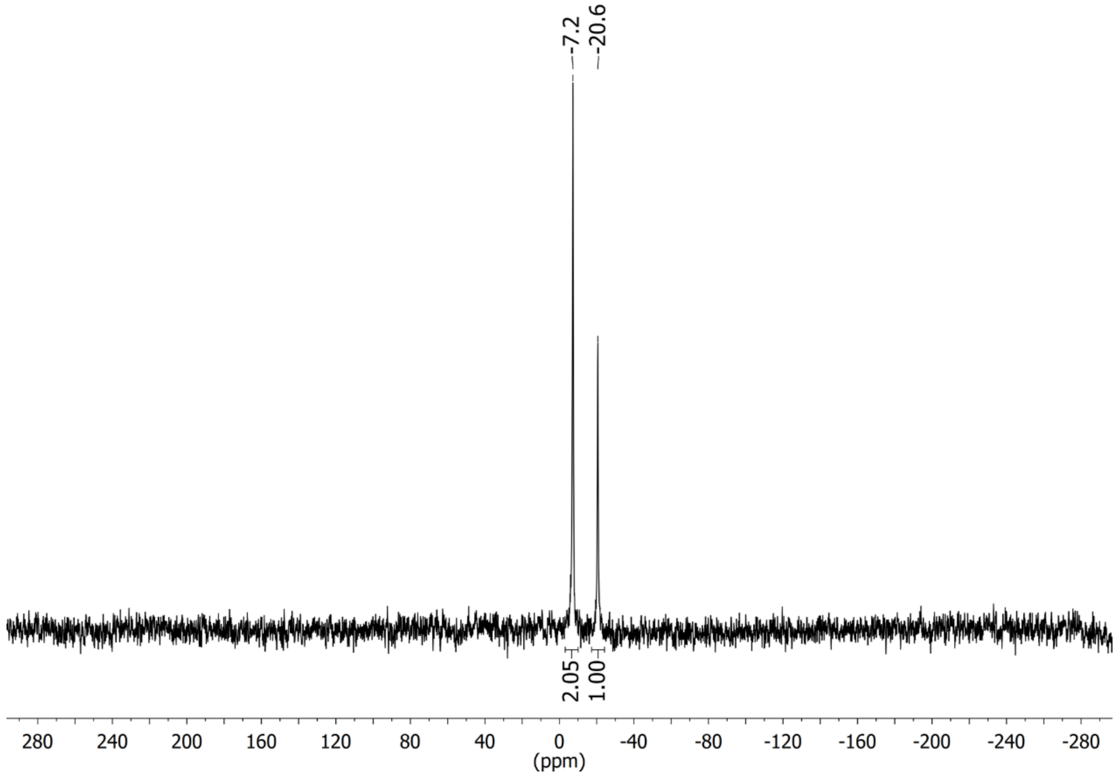

Figure S31. $^{31}P\{^1H\}$ NMR spectrum of **1CuBAr$^F_4$** in CD_2Cl_2 (exponential lb = 20 Hz).

1.8. Synthesis of {2,4,6-tris(1-diphenylphosphanyl-1'-ferrocenylene)-1,3,5-triazine-κP^3} silver(I) triflate (1Ag)

Under stirring and protected from direct light, a solution of 150 mg (127 µmol, 1.15 eq.) **1** in 12 mL CH_2Cl_2 was added to 28.3 mg (110 µmol, 1.00 eq.) AgOTf and kept stirring at room temperature for 1 hour, after which the protection from light was discontinued. The reaction mixture was filtered *via* cannula, layered with 36 mL toluene and kept at 7 °C overnight. The so-obtained crystalline material was isolated by filtration and dried *in vacuo* overnight at 40 °C, causing the deep red crystals to brittle, yielding a light-insensitive red powder (131 mg, 82% yield) which can be handled in air, but which was stored under nitrogen. Crystals suitable for single crystal X-ray diffraction analysis were obtained from layering a 1,2-dichloroethane solution of **1Ag** with toluene, stored at 7 °C for several days.

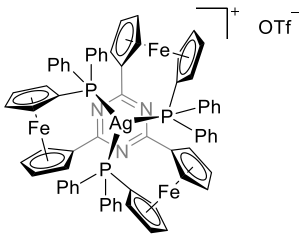

m.p. 270 °C (decomp.); ^1H NMR (CD_2Cl_2): $\delta_{25\,°C}$ [ppm] = 8.50–5.85 (m (br), 30 H, $P(C_6H_5)_2$), 5.46 (s (br), 6H, fc-H), 4.72 (s, 6H, fc-H), 4.29 (s, 6H, fc-H), 3.67 (s (br), 6H).; $\delta_{-60\,°C}$ [ppm] = 9.66 (m, 3H), 8.10 (pt, $J_{H,H}$ = 7.3 Hz, 3H), 7.75 (pt, $J_{H,H}$ = 7.4 Hz, 3H), 7.39–7.26 (m, 9H), 6.48 (m, 6H), 6.29 (m, 3H), 5.86 (m, 3H), 5.62 (s, 3H), 5.31 (s, 3H), 4.85 (s, 3H), 4.69 (s, 3H), 4.33 (s, 3H), 4.28 (s, 3H), 3.79 (s, 3H), 3.46 (s, 3H); $^{13}C\{^1H\}$ NMR (CD_2Cl_2): $\delta_{25\,°C}$ [ppm] = 176.2 (s, C_3N_3), 133.1 (s (br), $P(C_6H_5)_2$), 131.5 (dd, $^1J_{C,P}$ = 19.1 Hz, $^2J_{C,Ag}$ = 10.1 Hz, AgP(i-$C_6H_5)_2$), 130.7 (s (br), P(p-$C_6H_5)_2$), 129.0 (s, $P(C_6H_5)_2$), 81.3 (s, C_3N_3–C_{Cp}), 75.8 (m (br), fc-C), 74.7–71.0 (m (br), fc-C), 73.4 (dd, $^1J_{C,P}$ = 23.9 Hz, $^2J_{C,Ag}$ = 13.1 Hz, AgP-C_{Cp}), 72.5 (m (br), fc-C); $^{19}F\{^1H\}$ NMR (CD_2Cl_2): $\delta_{25\,°C}$ [ppm] = −78.9 (s, O_3SCF_3); $^{31}P\{^1H\}$ NMR (CD_2Cl_2): $\delta_{25\,°C}$ [ppm] = −6.0 (dd, $^1J_{P,^{107}Ag}$ = 323 Hz, $^1J_{P,^{109}Ag}$ = 373 Hz, 3P); IR (KBr): $\tilde{\nu}$ [cm^{-1}] = 3096 (w), 3055(w), 2963 (w), 2854 (w), 1722 (w), 1506 (vs), 1436 (m), 1383 (m), 1360 (w), 1319 (w), 1264 (s), 1223 (w), 1156 (m), 1095 (m) 1071 (w), 1030 (s), 927 (w), 829 (m), 805 (m), 747 (m), 697 (m), 637 (m), 757 (w), 500 (m), 452 (w); UV/Vis (CH_2Cl_2): λ_{max} [nm] (ε [$mol^{-1}\cdot L\cdot cm^{-1}$]) = 502 (3270); 369 (4910), 250 (71100); HRMS (ESI): m/z calcd. for $C_{69}H_{54}AgFe_3N_3P_3^+$: 1294.0642 [$M$−OTf]$^+$, found: 1294.0662; elemental analysis calcd. [%] for $C_{70}H_{54}AgF_3Fe_3N_3O_3P_3S$: C 58.28, H 3.77, N 2.91; found: C 58.75, H 3.81, N 2.83.

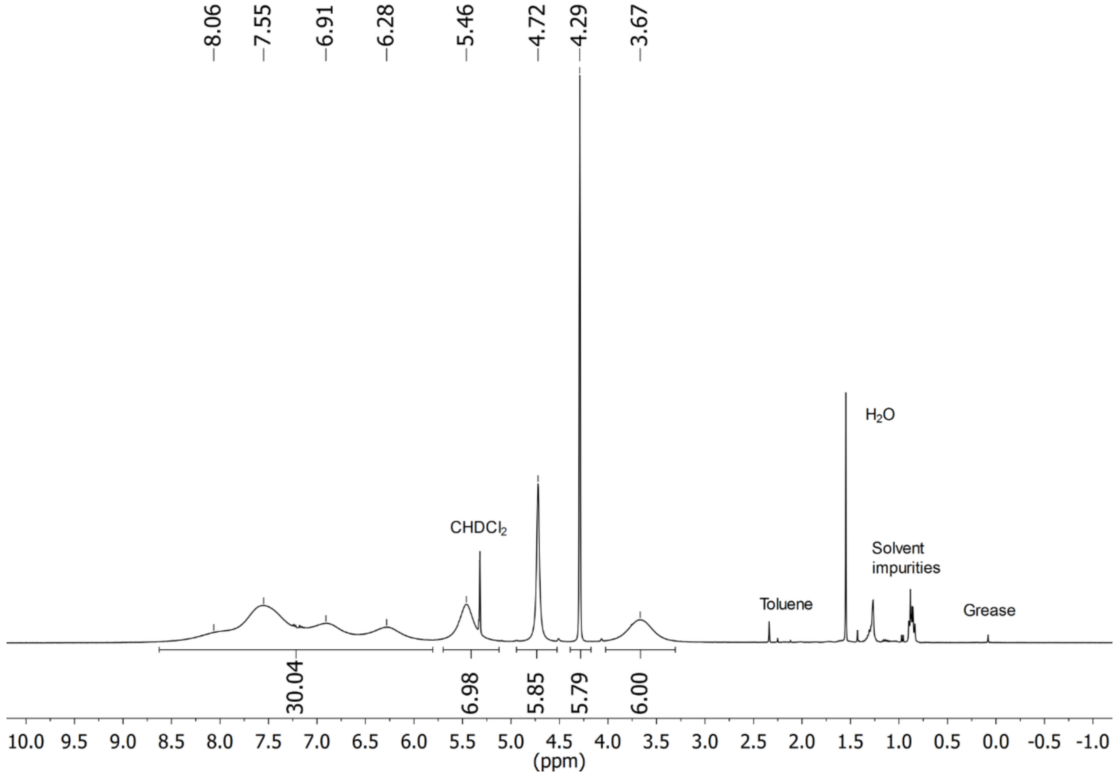

Figure S32. ^{1}H NMR spectrum of **1Ag** in CD_2Cl_2 recorded at 25 °C.

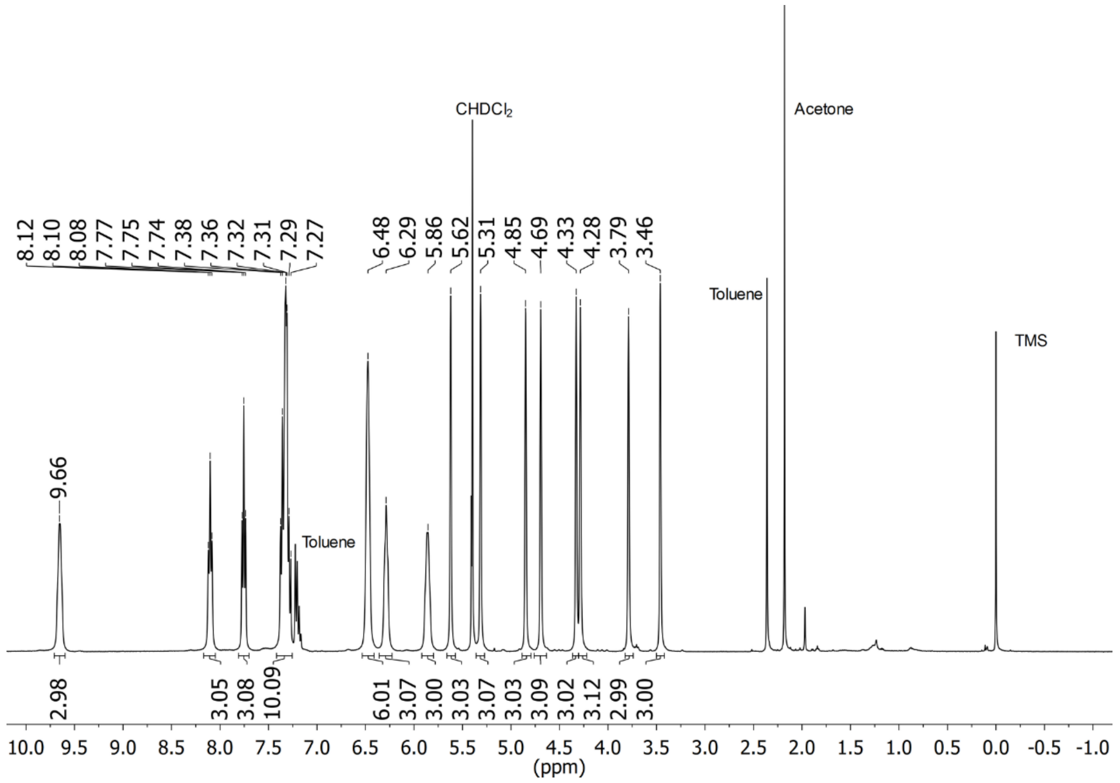

Figure S33. ^{1}H NMR spectrum of **1Ag** in CD_2Cl_2 recorded at −60 °C.

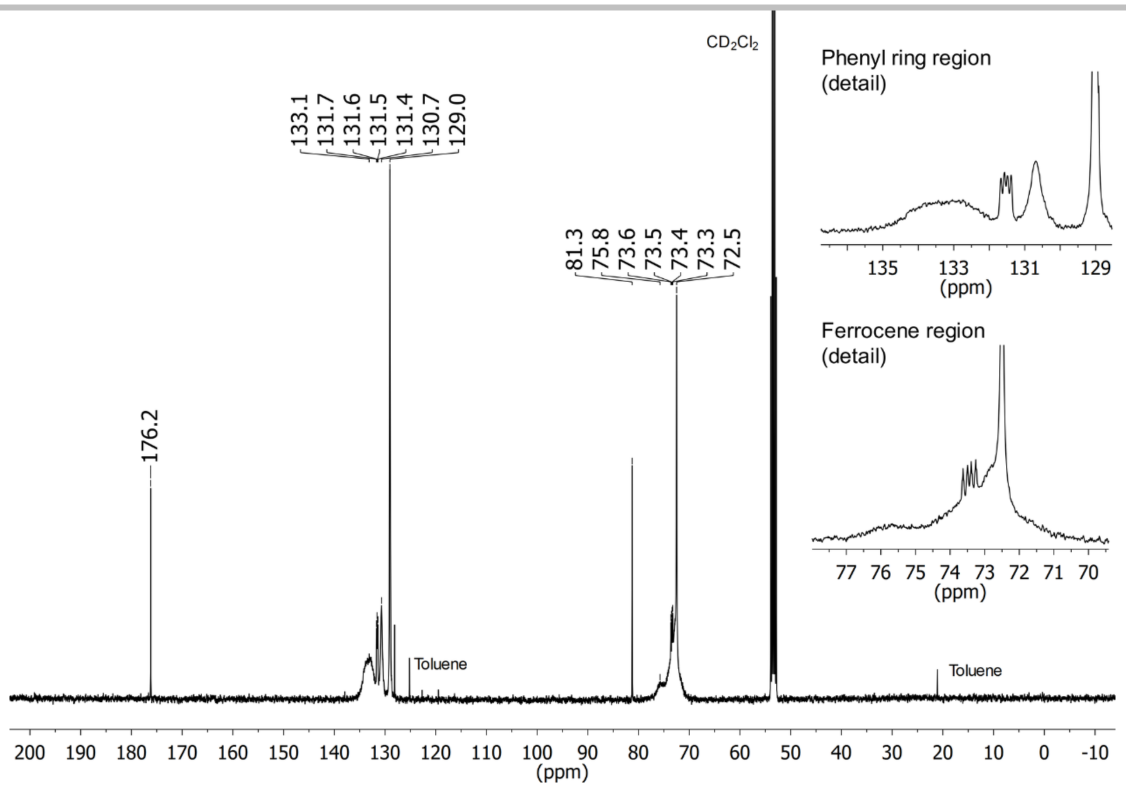

Figure S34. ^{13}C{^{1}H} NMR spectrum of **1Ag** in CD_2Cl_2 recorded at 25 °C using the z_gp_g pulse sequence for fast-relaxing nuclei (NS = 5760, 7 h). The inserts show details of the phenyl (top right) and of the ferrocenyl region (bottom right), showing the two pseudo-quadruplets arising from both $^{1}J_{C,P}$ and $^{2}J_{C,Ag}$ coupling as supported by the corresponding ^{13}C{^{31}P,^{1}H} NMR experiment (cf. Figure S35) in detail.

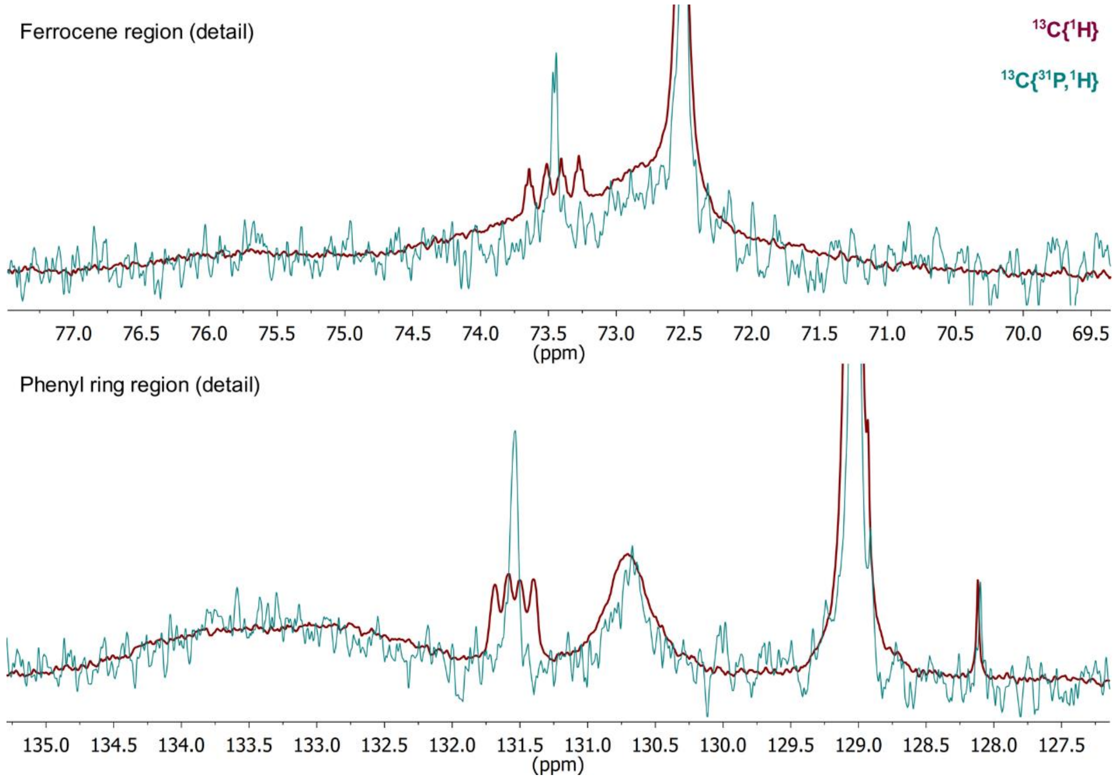

Figure S35. Overlay of the ^{13}C{^{1}H} (bold maroon) and ^{13}C{^{31}P,^{1}H} (thin teal) NMR spectra of **1Ag** in CD_2Cl_2 recorded at 25 °C, highlighting the collapse of the pseudo quadruplets in both the ferrocene (top) and the phenyl ring (bottom) region of the spectrum when ^{31}P decoupling is employed. Residual coupling to $^{107/109}$Ag can be registered for the signal at 73.5 ppm (top), while such coupling is not resolved in case of the signal at 131.5 ppm (bottom).

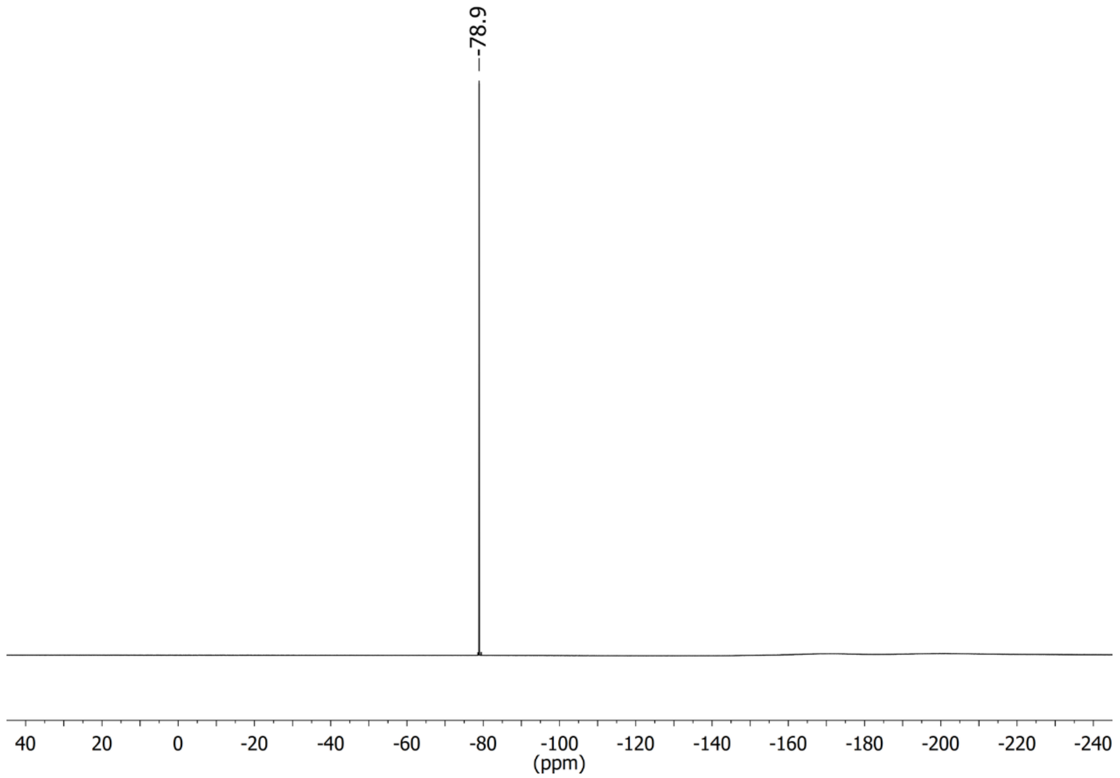

Figure S36. $^{19}F\{^1H\}$ NMR spectrum of **1Ag** in CD_2Cl_2 recorded at 25 °C.

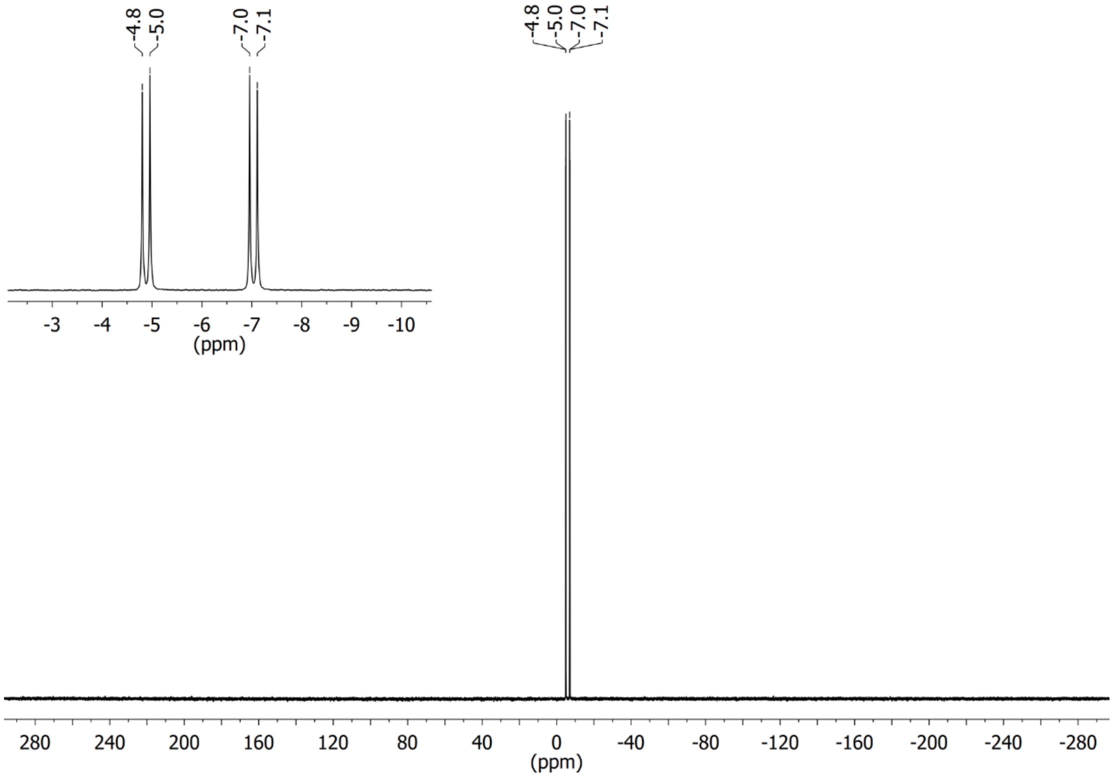

Figure S37. $^{31}P\{^1H\}$ NMR spectrum of **1Ag** in CD_2Cl_2 recorded at 25 °C, the insert showing the set of two doublets representing the two isotopomers due to distinguishable $^1J_{P,Ag}$ coupling to both ^{107}Ag and ^{109}Ag in detail.

1.9. Synthesis of {2,4,6-tris(1-diphenylphosphanyl-1'-ferrocenylene)-1,3,5-triazine-κP^3} gold(I) triflate (1Au)

Under stirring and protected from direct light, a solution of 235 mg (198 µmol, 1.13 eq.) **1** in 20 mL CH_2Cl_2 was added to 110 mg (175 µmol, 1.00 eq.) $[Au(nbe)_3]OTf$ and kept stirring at room temperature for 1 hour, after which the protection from light was discontinued. The reaction mixture was filtered *via* cannula, layered with 50 mL toluene and kept at 7 °C for 2 days. The so-obtained crystalline material was isolated by filtration and dried at 40 °C *in vacuo* overnight, causing the deep red crystals to brittle, yielding a light-insensitive red powder (190 mg, 71% yield) which can be handled in air, but which was stored under nitrogen. Crystals suitable for single crystal X-ray diffraction analysis were obtained from layering a 1,2-dichloroethane solution of **1Au** with toluene, stored at 7 °C for several days.

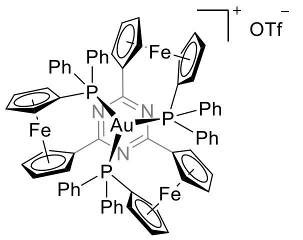

m.p. 228 °C (decomp.); ^{1}H NMR (CD_2Cl_2): $\delta_{25\,°C}$ [ppm] = 7.75–6.65 (m (br), 24H, $P(C_6H_5)_2$) 7.57–7.47 (m (br), 6H, $P(C_6H_5)_2$), 5.43 (s (br), 6H, fcH), 4.55 (s (br), 6H, fcH), 4.41 (s (br), 6H, fcH), 3.66 (s (br), 6H, fcH); $\delta_{-60\,°C}$ [ppm] = 9.72 (m, 3H), 8.18 (pt, $J_{H,H}$ = 7.6 Hz, 3H), 7.79 (pt, $J_{H,H}$ = 7.5 Hz, 3H), 7.37 (pt, $J_{H,H}$ = 7.7 Hz, 6H), 7.31 (pt, $J_{H,H}$ = 7.6 Hz, 3H), 6.49 (m, 3H), 6.39 (pt, $J_{H,H}$ = 7.7 Hz, 3H), 6.34 (m, 3H), 5.80 (pq, J = 7.2 Hz, 3H), 5.63 (s, 3H), 5.37 (s, 3H), 4.86 (s, 3H), 4.73 (s, 3H), 4.37 (s, 3H), 4.30 (s, 3H), 3.42 (s, 3H), 3.33 (s, 3H); $^{13}C\{^1H\}$ NMR (CD_2Cl_2)[3]: $\delta_{25\,°C}$ [ppm] = 175.4 (s, C_3N_3), 133.3 (s (br), $P(C_6H_5)_2$), 131.0 (s, P(*p*-$C_6H_5)_2$), 129.0 (s, $P(C_6H_5)_2$), 81.5 (s, C_3N_3–C_{Cp}), 75.2 (s (br), fc-C), 73.3 (s, br), fc-C), 72.8 (s, fc-C), 72.1 (s, fc-C); $^{19}F\{^1H\}$ NMR (CD_2Cl_2): $\delta_{25\,°C}$ [ppm] = −79.0 (s, O_3SCF_3); $^{31}P\{^1H\}$ NMR (CD_2Cl_2): $\delta_{25\,°C}$ [ppm] = 26.8 (s (br), 3P); IR (KBr): $\tilde{\nu}$ [cm^{-1}] = 3093 (w), 3052 (w), 3018 (w), 1510 (vs), 1488 (s), 1435 (m), 1384 (m), 1362 (m), 1320 (w), 1265 (s), 1222 (w), 1151 (m), 1095 (w), 1030 (s), 925 (w), 882 (w), 830 (m), 748 (m), 696 (m), 636 (m), 568 (w), 513 (m), 500 (m), 488 (m), 458 (m), 422 (w); UV/Vis (CH_2Cl_2): λ_{max} [nm] (ε [$mol^{-1}\cdot L\cdot cm^{-1}$]) = 492 (2650), 363 (4930), 249 (62200); HRMS (ESI): m/z calcd. for $C_{69}H_{54}AuFe_3N_3P_3^+$: 1382.1245 [*M*−OTf]$^+$, found: 1382.1233; elemental analysis calcd. [%] for $C_{70}H_{54}AuF_3Fe_3N_3O_3P_3S$: C 54.89, H 3.55, N 2.74; found: C 55.02, H 3.52, N 2.73.

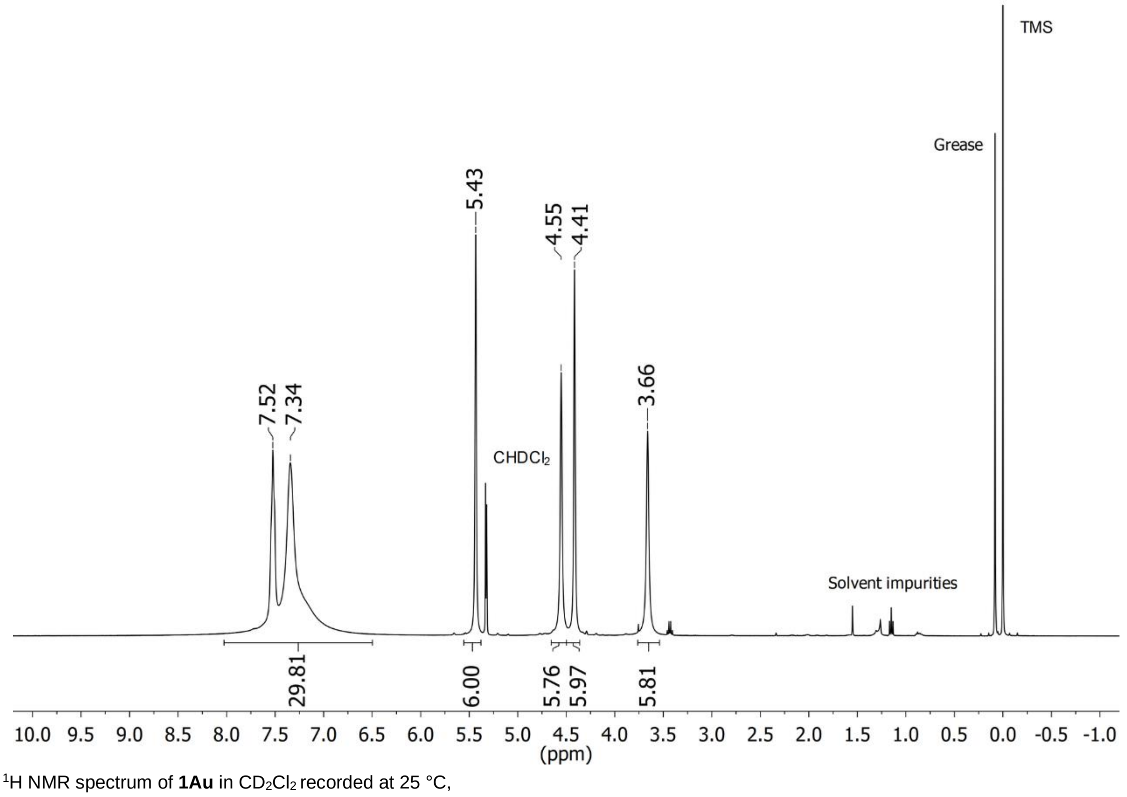

Figure S38. ^{1}H NMR spectrum of **1Au** in CD_2Cl_2 recorded at 25 °C,

[3] The quaternary *ipso* carbon atoms of the *P*-bound phenyl and cyclopentadienyl rings are not detectable, owing to both the $^1J_{P,C}$ coupling and the intramolecular dynamics regarding the inversion of helicity.

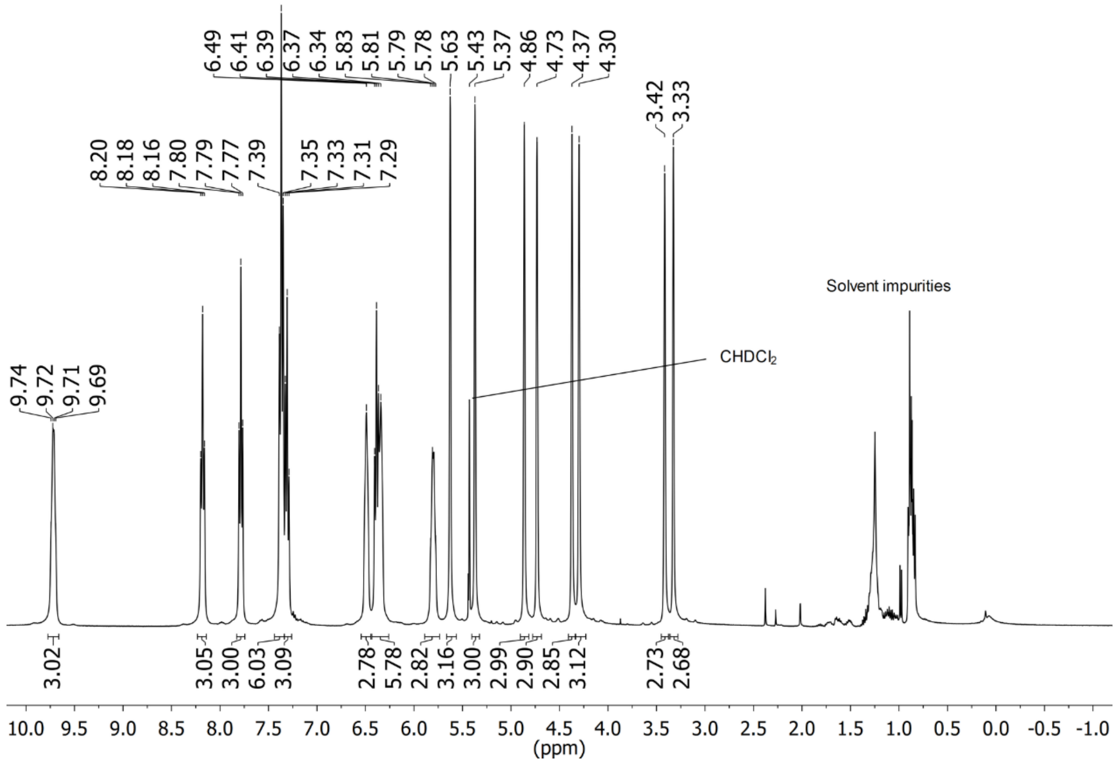

Figure S39. ^{1}H NMR spectrum of **1Au** in CD_2Cl_2 recorded at −60 °C.

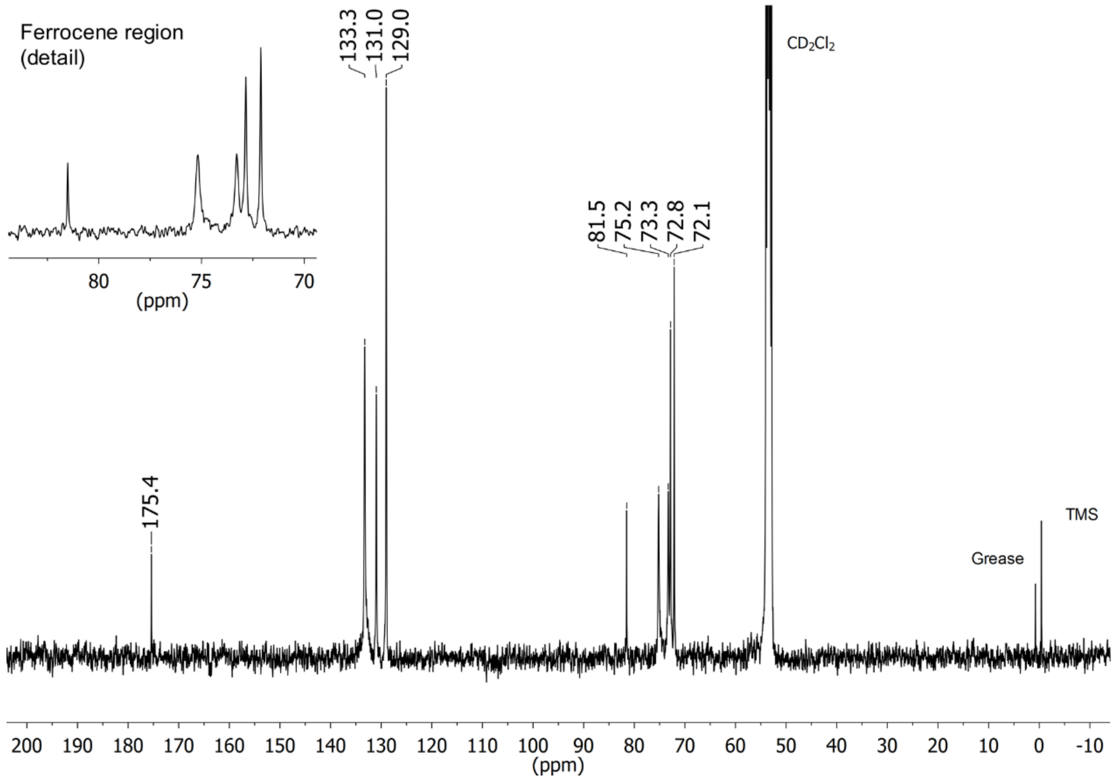

Figure S40. $^{13}C\{^{1}H\}$ NMR spectrum of **1Au** in CD_2Cl_2 (exponential lb = 5 Hz) recorded at 25 °C, the insert shows details of the ferrocene region.

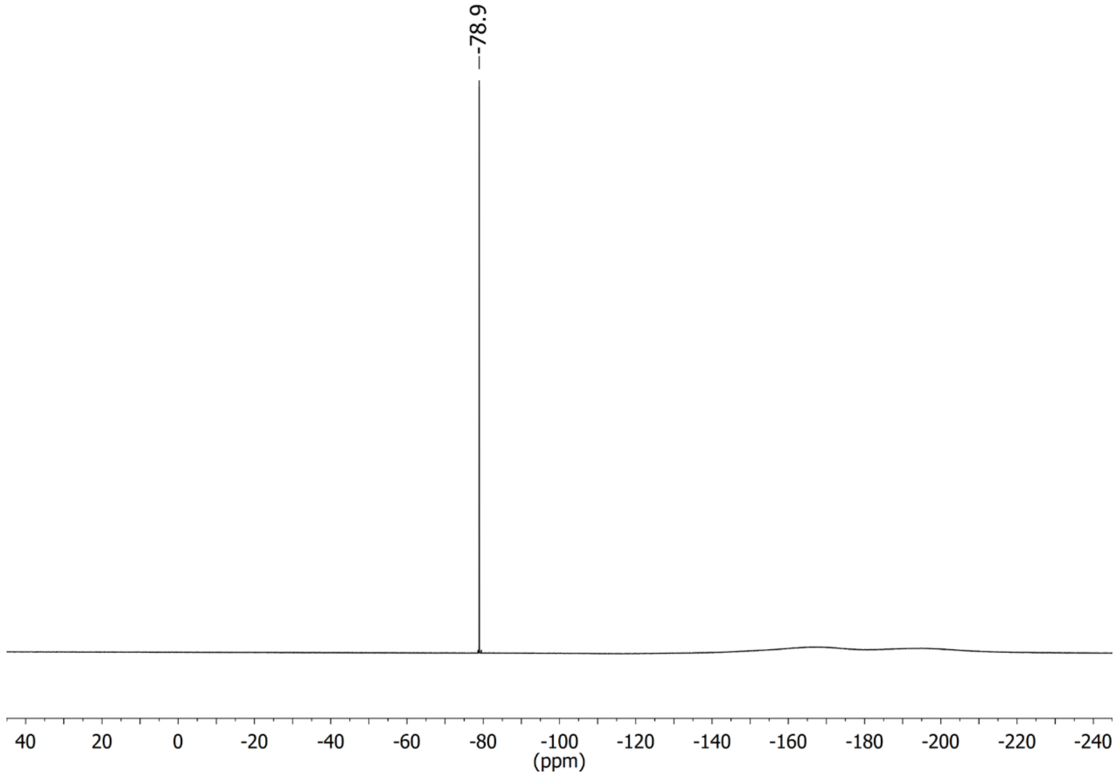

Figure S41. $^{19}F\{^1H\}$ NMR spectrum of **1Au** in CD_2Cl_2.

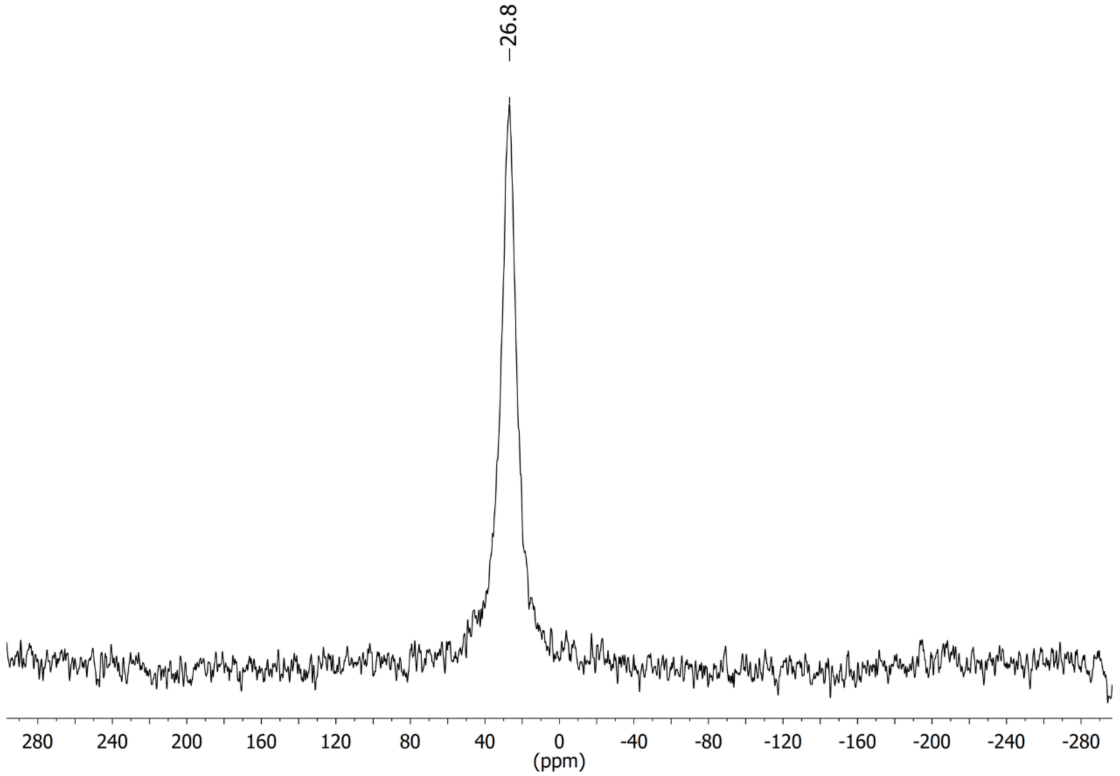

Figure S42. $^{31}P\{^1H\}$ NMR spectrum of **1Au** in CD_2Cl_2 (exponential lb = 100 Hz) recorded at 25 °C.

1.10. Oxidation of 1Au to yield {2,4-bis(1-diphenylphosphanyl-1'-ferrocenylene)-6-(1-diphenylphosphoryl)-1,3,5-triazin-1-um-κP^2} gold(I) bis[tetrakis{3,5-bis(trifluoromethyl)phenyl}borate] (5)

Under argon and maintaining gentle stirring, 23 mg (27 µmol, 6.0 eq.) $NaBAr^F_4$ and 12 mg (14 µmol, 3.0 eq.) $NaBAr^F_4$ were separately suspended in 5 mL CH_2Cl_2 and 3 mL CH_2Cl_2 over molecular sieves (4 Å) respectively and kept stirring overnight.[4] Both suspensions were decanted from the molecular sieves *via* cannula onto 2.8 mg [thianthrene]tetrafluoroborate (9.2 µmol, 2.0 eq.) and 7.0 mg **1Au** (4.6 µmol, 1.0 eq.), respectively, and were stirred for 30 min at room temperature. The intensely purple suspension of [thianthrenium]BAr^F_4 was cooled to 0 °C (ice/water), allowed to settle, and filtered into the suspension of the gold complex. This suspension was stirred for another 30 min at room temperature. Filtration from the colourless precipitate afforded a vibrant violet solution from which an HR-ESI mass spectrum, a $^{19}F\{^1H\}$, and a $^{31}P\{^1H\}$ spectrum were recorded, identical to the analytical data obtained from the crystalline material (*vide infra*).
The volatiles were removed *in vacuo*, the solid violet residue was washed with hexanes (2x 5 mL) and dried *in vacuo*. Crystals suitable for single crystal X-ray diffraction analysis were obtained from layering a 1,2-dichloroethane solution of **5** with hexanes, stored at 7 °C for several days and subsequent tempering at 35 °C for 4 days. No yield was determined, and the product obtained by crystallisation was found to be impure by means of NMR spectroscopy, yet an attributable set of spectra could be recorded and analysed at 25 °C (^{11}B, ^{19}F, ^{31}P) and −40 °C (^1H).[5]

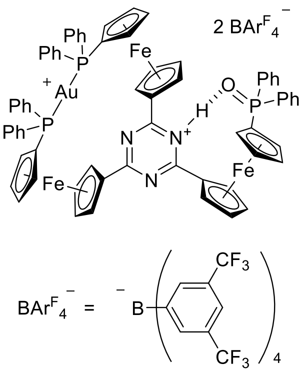

^1H NMR (CD_2Cl_2): $\delta_{-40\,°C}$ [ppm] = 8.13–7.83 (m, 1H, P(C_6H_5)), 7.80–7.73 (m, 3H. P(C_6H_5)), 7.73–7.69 (m, 8H, B($C_6H_3(CF_3)_2$)), 7.67–7.55 (m, 1H, P(C_6H_5)), 7.53–7.49 (m, 3H, B($C_6H_3(CF_3)_2$)), 7.48–7.27 (m, 2H, P(C_6H_5)), 7.27–7.17 (m, 1H, P(C_6H_5)), 6.37 (m, 1H, fcH(**A^1**)), 5.84 (m, 1H, fcH(**B^1**)), 5.49 (m, 2H, fcH(**A^2+C^1**)), 5.38 (m, 1H, fcH(**C^2**)), 5.19 (m, 1H, fcH(**B^2**)), 5.08 (m, 1H, fcH(**D^1**)), 5.00 (m, 1H, fcH(**D^2**)), 4.91 (m, 2H, fcH(**A^3+E^1**)), 4.83 (m, 2H, fcH(**F^1+F^2**)), 4.81 (m, 1H, fcH(**B^3**)), 4.78 (m, 1H, fcH(**B^4**)), 4.74 (m, 1H, fcH(**C^3**)), 4.56 (m, 2H, fcH(**D^3+F^3**)), 4.39 (m, 1H, fcH(**E^2**)), 4.34 (m, 1H, fcH(**E^3**)), 4.17 (m, 1H, fcH(**D^4**)), 4.06 (m, 1H, fcH(**E^4**)), 3.99 (m, 1H, fcH(**F^4**)), 3.38 (m, 1H, fcH(**C^4**)), 3.32 (m, 1H, fcH(**A^4**))[6]; $^{11}B\{^1H\}$ NMR (CD_2Cl_2): $\delta_{25\,°C}$ [ppm] = −6.7 (s, B($C_6H_3(CF_3)_2$); $^{19}F\{^1H\}$ NMR (CD_2Cl_2): $\delta_{25\,°C}$ [ppm] = −62.8 (s, B($C_6H_3(CF_3)_2$)); $^{31}P\{^1H\}$ NMR (CD_2Cl_2): $\delta_{25\,°C}$ [ppm] = 41.3 (s, 2P, P–Au–P), 34.9 (s, 1P, P=O); IR (KBr): $\tilde{\nu}$ [cm^{-1}] = IR (neat): $\tilde{\nu}$ [cm^{-1}] = 2960 (w), 2927 (w), 2856 (w), 1595 (m), 1563 (m), 1520 (m), 1480 (m), 1438 (m), 1384 (w), 1353 (s), 1272 (vs), 1114 (vs, broad, P=O), 1030 (m), 999 (m) 927 (w), 886 (m), 838 (m), 813 (m), 744 (m), 728 (s), 692 (m), 680 (s), 668 (s), 625 (w), 568 (m), 539 (w), 501 (m, broad), 477 (m), 450 (m), 431 (m), 406 (m); ; UV/Vis (CH_2Cl_2)[7]: λ_{max} [nm] = 553, 378 (shoulder), 328, 268 (shoulder); HRMS (ESI): m/z calcd. for $C_{69}H_{55}AuFe_3N_3OP_3^{2+}$: 699.5634 [$M$−2$BAr^F_4$]$^{2+}$, found: 699.5592.

[4] We found that using [thianthrenium]BF_4 rather than the anion-exchanged oxidant led to decomposition of the BF_4^- anion, as monitored by $^{19}F\{^1H\}$ NMR spectroscopy. Drying the suspension of $NaBAr^F_4$ in CH_2Cl_2 over 4 Å molecular sieves has also been found crucial for obtaining **5**; however, residual water in $NaBAr^F_4$ might still account as the source for oxygen and the proton at the C_3N_3 core.

[5] The ^1H NMR spectrum recorded at 25 °C is just dominated by the ^1H resonances of the $BAr^F_4{}^-$ anion, while all other signals of interest are considerably broadened. As for the heteronuclear spectra, no difference between 25 °C and −40 °C can be detected, thus the signal sharpening is attributed to slowed intramolecular dynamics of the ferrocenylene moieties which do not affect the heteronuclei.

[6] Due to the impurities and other species, the ^1H NMR signal for the proton at the C_3N_3 core cannot unambigiously be allocated. It is further expected to be significantly broadened due to its binding to quadrupolar ^{14}N and, possibly, due to coupling to the ^{31}P nucleus of the phosphoryl moiety.

[7] Since **5** was found impure even after crystallisation, no extinction coefficients can be provided as no exact concentration of the species in solution could be determined. It should furthermore be noted that the UV/Vis spectrum (Figure S47), due to the impurities, is a sum spectrum of all species in solution and not all transitions might thus originate from **5** itself.

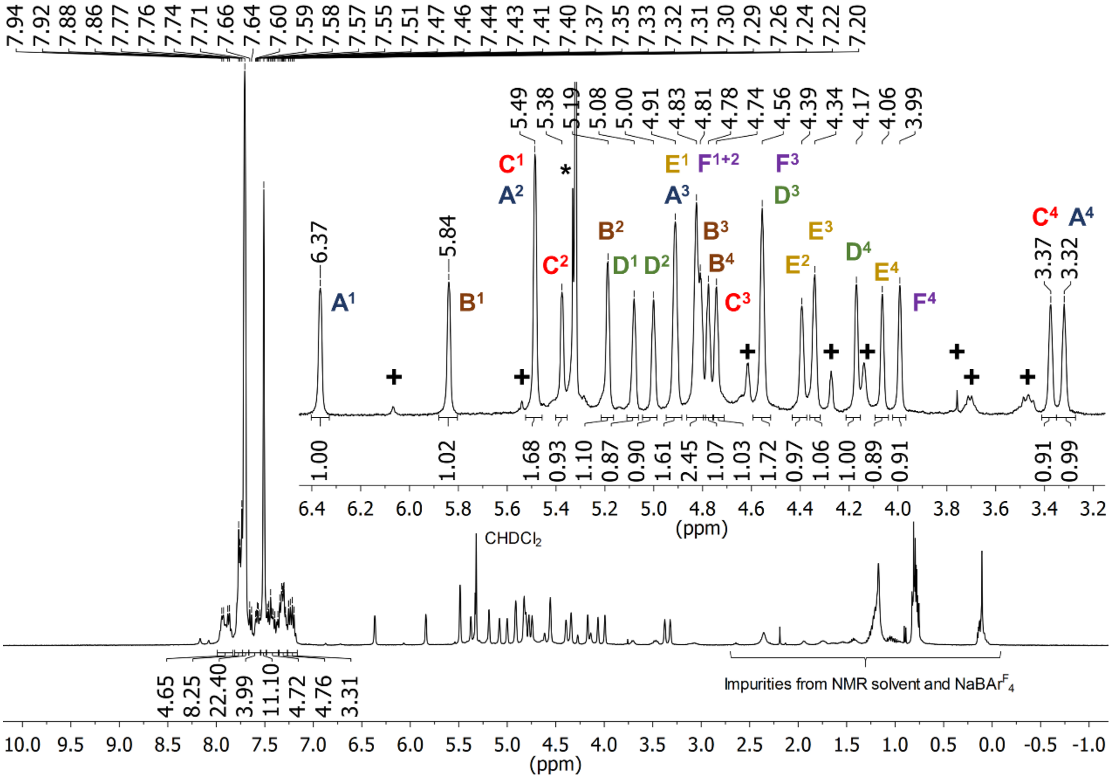

Figure S43. ^{1}H NMR spectrum of **5** in CD_2Cl_2 recorded at −40 °C; the insert magnifies the region of the ferrocene protons which, through a $^{1}H,^{1}H$ COSY NMR experiment conducted at −40 °C, have been assigned to six individual C_5H_4 rings **A** to **F**. The plus sign (**+**) denotes signals attributable to a second species in solution, the asterisk (*****) denotes the solvent signals (CH_2Cl_2, $CHDCl_2$). The integrals for the ^{1}H resonances of the $BAr^{F}{}_4{}^{-}$ anion are too large, most likely due to the presence of dissolved $NaBAr^{F}{}_4$.

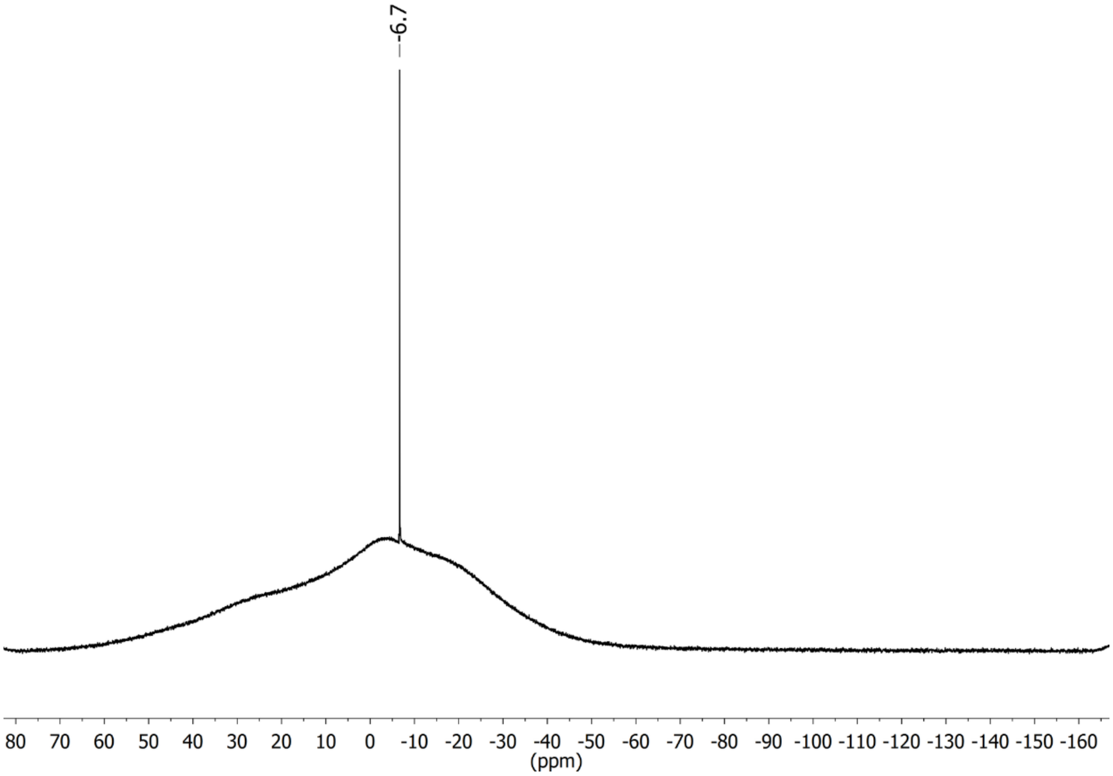

Figure S44. $^{11}B\{^{1}H\}$ NMR spectrum of **5** in CD_2Cl_2 recorded at 25 °C.

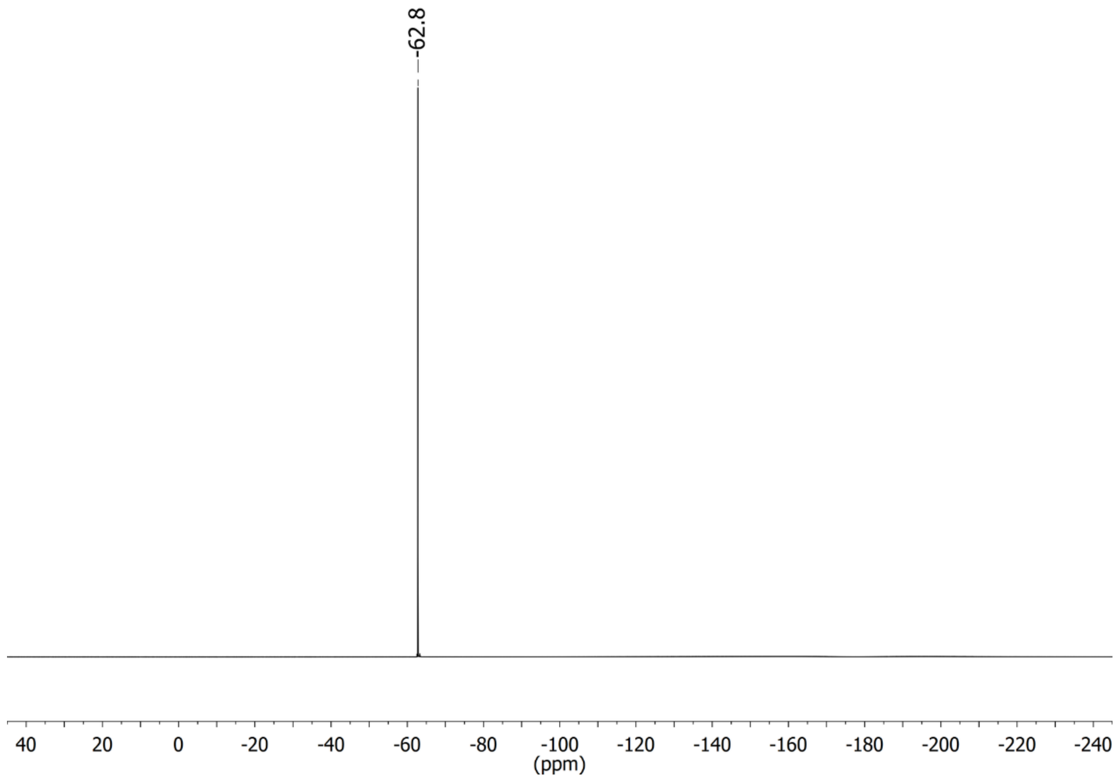

Figure S45. $^{19}F\{^1H\}$ NMR spectrum of **5** in CD_2Cl_2 recorded at 25 °C.

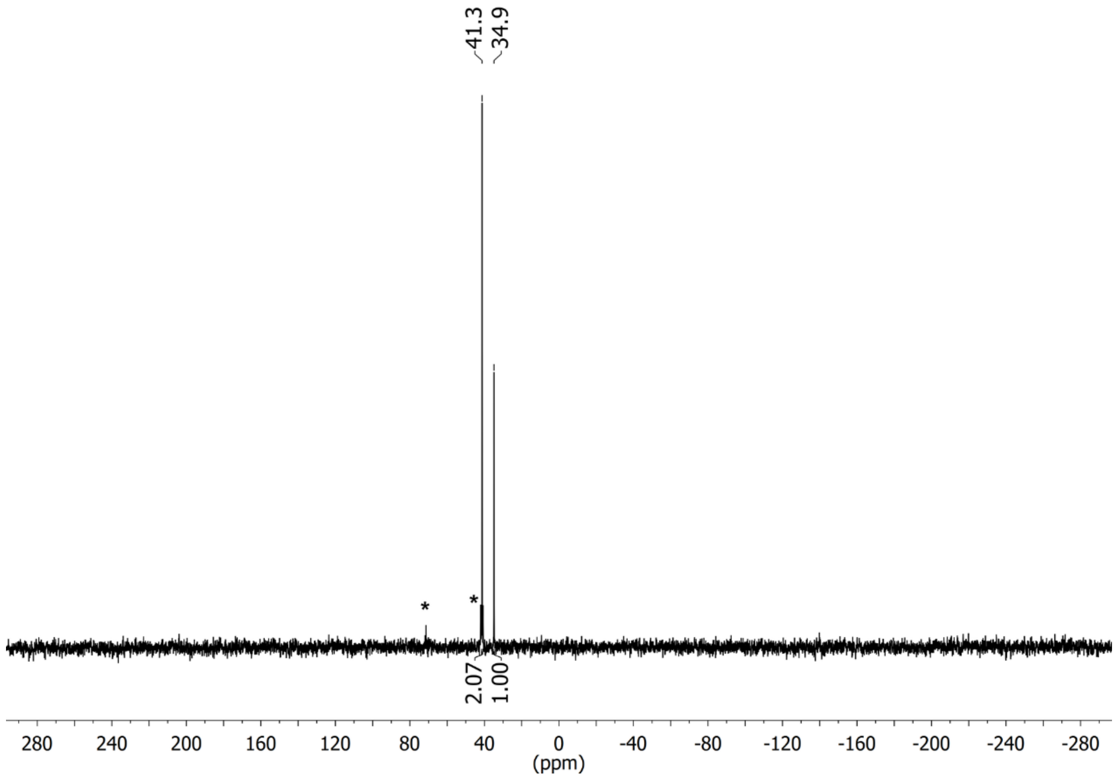

. **Figure S46.** $^{31}P\{^1H\}$ NMR spectrum of **5** in CD_2Cl_2 recorded at 25 °C. The asterisks (*****) denote signals attributable to a second component in solution.

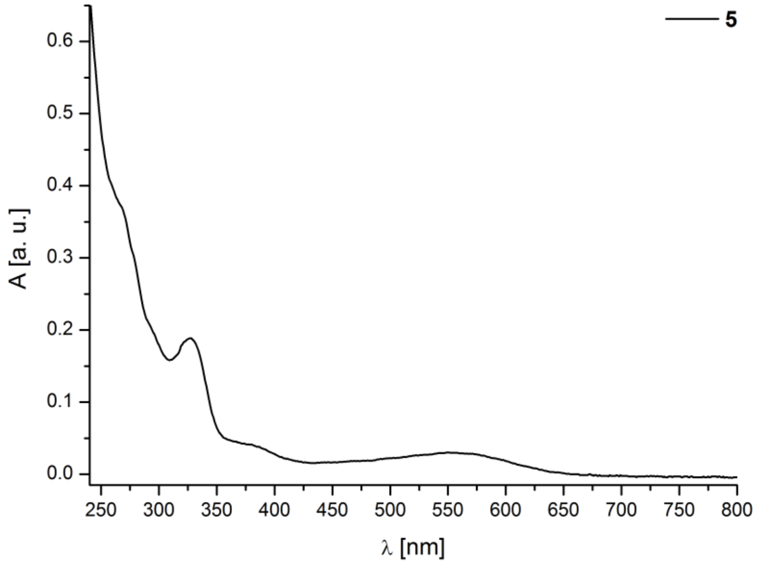

Figure S47. UV/Vis spectrum of **5** in CH_2Cl_2.

2. Results and Discussion

2.1. Single Crystal X-Ray Diffraction Analyses

Table S1. Crystallographic data for ligand **1**, borane-protected **$1BH_3$**, and complexes **1Cu**, **$1CuBF_4$**, and **$1CuBAr^F{}_4$**.

	**1**	**$1BH_3$**	**1Cu**	**$1CuBF_4$**	**$1CuBAr^F{}_4$**
Empirical formula[a]	$C_{69}H_{54}Fe_3N_3P_3 \cdot CH_2Cl_2$	$C_{69}H_{63}B_3Fe_3N_3P_3$	$C_{69}H_{54}CuFe_3N_3P_3 \cdot CF_3O_3S \cdot 1.33(C_7H_8) \cdot 1.35(CH_2Cl_2)$	$C_{69}H_{54}CuFe_3N_3P_3 \cdot BF_4 \cdot C_7H_8 \cdot CH_2Cl_2$	$C_{69}H_{54}CuFe_3N_3P_3 \cdot C_{32}H_{12}BF_{24}$
Formula weight [$g \cdot mol^{-1}$]	1270.53	1227.11	1638.40	1513.02	2112.37
T [K]	130(2)	130(2)	130(2)	130(2)	130(2)
Crystal system / Space group	Triclinic / $P\bar{1}$	Trigonal / $R\bar{3}$	Triclinic / $P\bar{1}$	Triclinic / $P\bar{1}$	Monoclinic / $P2_1/c$
a, b, c [Å]	13.401(1), 15.021(1), 17.009(1)	28.3139(4), 28.3139(4), 12.6860(3)	13.2878(3), 16.5913(5), 18.2263(6)	13.0612(2), 17.2516(3), 17.8635(4)	13.9342(2), 34.1657(4), 20.1810(3)
α, β, γ [°]	99.247(6), 104.500(6), 93.598(6)	90, 90, 120	113.779(3), 93.192(2), 102.445(2)	112.720(2), 94.454(2), 104.621(2)	90, 96.808(1), 90
V [$Å^3$]	3252.5(4)	8807.5(3)	3544.7(2)	3522.7(1)	9539.9(2)
Z	2	6	2	2	4
ρ_{calc} [$g \cdot cm^{-3}$]	1.297	1.388	1.535	1.426	1.471
Θ_{max} [°]	28.282	26.367	30.585	29.065	27.968
F(000)	1308	3816	1677	1548	4264
Reflns collected	29172	30216	57576	51940	54648
Independent reflns	16105	4009	19559	16609	20727
R_1/wR_2 {$I > 2\sigma(I)$}	0.1103/0.2369	0.0350/0.0705	0.0480/0.0947	0.0455/0.1076	0.0552/0.1094
R_1/wR_2 (all data)	0.2641/0.3013	0.0489/0.0757	0.0797/0.1069	0.0651/0.1152	0.0906/0.1242
Largest diff. peak/hole [$e \cdot Å^{-3}$]	1.970/−1.011	0.306/−0.238	0.755/−0.687	1.291/−0.574	0.823/−0.595

[a] Given as Compound·Solvent or Cation·Anion·Solvent for reasons of clarity.

Table S2. Crystallographic data for complexes **1Ag**, **1Au**, and **5**.

	**1Ag**	**1Au**	**5**
Empirical formula[a]	$C_{69}H_{54}AgFe_3N_3P_3 \cdot CF_3O_3S \cdot C_2H_4Cl_2$	$C_{69}H_{54}AuFe_3N_3P_3 \cdot CF_3O_3S \cdot C_{2.5}H_5Cl_{2.5}$	$C_{69}H_{55}AuFe_3N_3OP_3 \cdot (C_{32}H_{12}BF_{24})_2 \cdot C_2H_4Cl_2 \cdot C_{2.7}H_{6.3}Cl_{0.14}$
Formula weight [g·mol^{-1}]	1541.50	3310.67	3268.87
T [K]	130(2)	130(2)	130(2)
Crystal system / Space group	Triclinic / $P\bar{1}$	Triclinic / $P\bar{1}$	Triclinic / $P\bar{1}$
a, b, c [Å]	13.3745(3), 16.8049(4), 17.9030(4)	13.3154(4), 16.8045(5), 17.9304(6)	16.3845(3), 17.6125(3), 25.0125(4)
α, β, γ [°]	111.620(2), 94.970(2), 103.191(2)	112.378(3), 94.692(2), 103.020(2)	103.380(2), 90.449(1), 110.656(2)
V [Å^3]	3576.7(2)	3551.5(2)	6538.8(2)
Z	2	2	2
ρ_{calc} [g·cm^{-3}]	1.431	1.548	1.660
Θ_{max} [°]	30.508	30.570	30.508
F(000)	1564	1653	3250
Reflns collected	72078	40691	154729
Independent reflns	21809	19248	39928
R_1/wR_2 {$I > 2\sigma(I)$}	0.0404/0.0848	0.0586/0.1336	0.0425/0.0951
R_1/wR_2 (all data)	0.0599/0.0910	0.0972/0.1496	0.0604/0.1048
Largest diff. peak/hole [e·Å^{-3}]	0.641/−0.648	3.005/−1.218	1.399/−1.318

[a] Given as Cation·Anion·Solvent(s) for reasons of clarity.

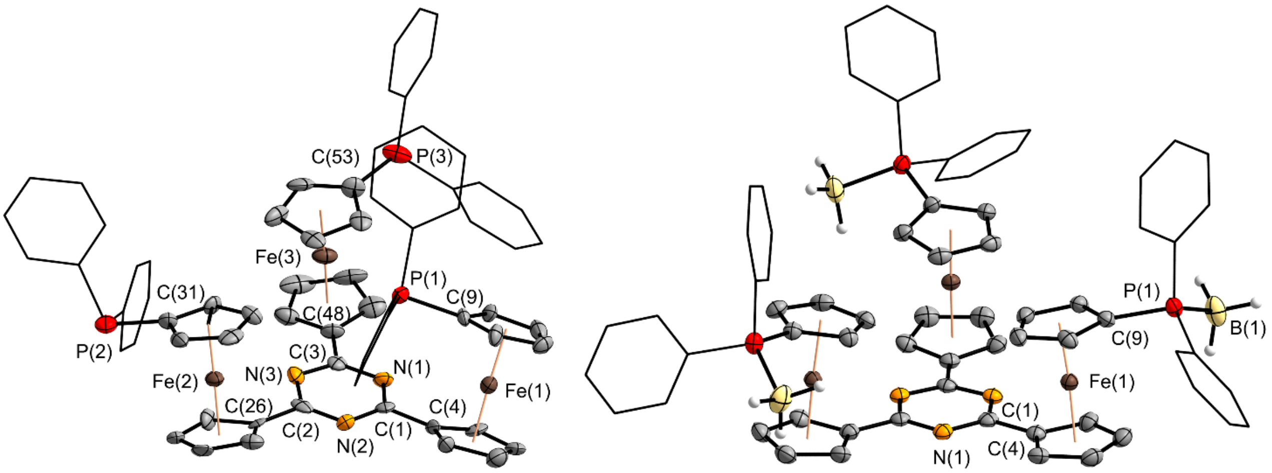

Figure S48. Molecular structures of **1** (left) and **1BH₃** (right) and their atom labelling schemes with thermal displacement ellipsoids shown at the 50% probability level. For clarity, *P*-bound phenyl rings are depicted in wireframe style, and solvent molecules and H atoms (except for the BH_3 groups in **$1BH_3$**) are not shown.

Ligand **1** crystallises with no crystallographic symmetry, but key structural parameters for the three ferrocenylene moieties are in good accordance with each other and within the typical values reported for related compounds. The all-*syn* arrangement of the three 1-diphenylphosphanyl groups with respect to the central triazine core does not seem to arise from either intra- or intermolecular contacts. For the closely related compounds 1,3,5-tris(ferrocenyl)benzene[25] and 2,4,6-tris(ferrocenyl)pyridine[26] *syn,anti,anti* conformations are found in the solid state with two ferrocenyl groups on one side of the arene core and the third on the other, while 2,4,6-tris(ferrocenyl)-1,3,5,2,4,6-triselenatriborinane[27] (also containing an aromatic six-membered core) crystallises in the all-*syn* conformation, as does the 1,3,5-tris(ferrocenyl)benzene moiety with the benzene core η^6-bound to a RuCp fragment (**V** in Figure 1 in the main article).[28]
The tris(borane) adduct **$1BH_3$** crystallises with crystallographic C_3 symmetry, obviously also resulting in an all-*syn* conformation. Its structural parameters are in line with expectations towards such phosphanyl boranes.

Table S3. Selected bond lengths [Å], distances [Å], and angles [°] of ligand **1** and borane-protected **$1BH_3$**. Numbering scheme according to the respective depiction of the solid-state structures.

	**1**	**$1BH_3$**[a]		**1**	**$1BH_3$**
C(9)–P(1) / C(31)–P(2) / C(53)–P(3)	1.815(8) / 1.809(9) / 1.83(1)	1.784(2)	Ct^C(1)–Fe(1)–Ct^P(1) / Ct^C(2)–Fe(2)–Ct^P(2) / Ct^C(3)–Fe(3)–Ct^P(3) (α)	177.82 / 178.72 178.89	176.59
C(1)–C(4) / C(2)–C(26) / C(3)–C(48)	1.47(1) / 1.46(1) / 1.45(1)	1.460(3)	Θ(fc(1)) / Θ(fc(2)) / Θ(fc(3))[d]	3.32 / 2.15 / 0.33	4.72
Ct^C(1)–Fe(1) / Ct^C(2)–Fe(2) / Ct^C(3)–Fe(3)[b]	1.653 / 1.634 / 1.653	1.648	τ(fc(1)) / τ(fc(2)) / τ(fc(3))[e]	24.42 / 125.05 / 131.27	27.21
Ct^P(1)–Fe(1) / C^{tP}(2)–Fe(2) / C^{tP}(3)–Fe(3)[c]	1.654 / 1.630 / 1.637	1.642	P(1)–B(1) / P(2)–B(2) / P(3)–B(3) /	–	1.919(3)

[a] Due to crystallographic C_3 symmetry, only one value is shown. [b] Ct^C denotes the calculated centre of gravity of the carbon-substituted cyclopentadienyl ring. [c] Ct^P denotes the calculated centre of gravity of the phosphorus-substituted cyclopentadienyl ring. [d] Angle between the mean planes through the substituted cyclopentadienyl (Cp^R) rings. [e] Torsion about the Cp^P(centroid)···Fe···Cp^C(centroid) axes.

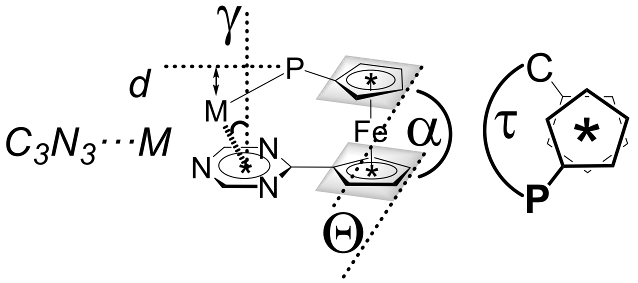

Figure S49. Schematic representation of key structural parameters for the compounds under investigation.

Table S4. Selected bond lengths [Å], distances [Å], and angles [°] of complexes **1Cu**, **1Ag**, and **1Au**. Numbering scheme according to the respective depiction of the solid-state structures (cf Figure 2 in the main article).

	**1Cu**	**1Ag**	**1Au**		**1Cu**	**1Ag**	**1Au**
C(9)–P(1) / C(31)–P(2) / C(53)–P(3)	1.803(2) / 1.792(3) / 1.807(3)	1.796(2) / 1.801(2) / 1.794(2)	1.798(6) / 1.802(7) / 1.789(7)	P(1)–M(1)–P(2) / P(2)–M(1)–P(3) / P(3)–M(1)–P(1)	117.14(3) / 117.64(3) / 120.35(3)	119.46(2) / 117.59(2) / 116.02(2)	119.63(6) / 118.48(6) / 117.28(6)
C(1)–C(4) / C(2)–C(26) / C(3)–C(48)	1.471(4) / 1.459(3) / 1.459(3)	1.475(4) / 1.460(3) / 1.463(3)	1.472(9) / 1.451(8) / 1.459(8)	Ct^{C}(1)–Fe(1)–Ct^{P}(1) / Ct^{C}(2)–Fe(2)–Ct^{P}(2) / Ct^{C}(3)–Fe(3)–Ct^{P}(3) (α)	173.20 / 171.94 / 172.91	173.32 / 173.49 / 172.85	173.55 / 173.22 / 172.57
P(1)–M(1) / P(2)–M(1) / P(3)–M(1)	2.3266(7) / 2.3178(7) / 2.3338(6)	2.4880(7) / 2.4968(5) / 2.4881(6)	2.393(2) / 2.402(1) / 2.393(2)	Θ(fc(1)) / Θ(fc(2)) / Θ(fc(3))[c]	7.49 / 8.15 / 6.79	7.28 / 6.48 / 6.98	7.60 / 6.31 / 7.34
Ct^{C}(1)–Fe(1) / Ct^{C}(2)–Fe(2) / Ct^{C}(3)–Fe(3)[a]	1.652 / 1.660 / 1.663	1.664 / 1.661 / 1.660	1.659 / 1.668 / 1.654	τ(fc(1)) / τ(fc(2)) / τ(fc(3))[d]	22.33 / 24.03 / 20.22	24.73 / 22.52 / 25.36	23.13 / 22.43 / 24.58
Ct^{P}(1)–Fe(1) / C^{tP}(2)–Fe(2) / C^{tP}(3)–Fe(3)[b]	1.662 / 1.646 / 1.655	1.651 / 1.653 / 1.650	1.649 / 1.667 / 1.647				

[a] Ct^{C} denotes the calculated centre of gravity of the carbon-substituted cyclopentadienyl ring. [b] Ct^{P} denotes the calculated centre of gravity of the phosphorus-substituted cyclopentadienyl ring. [c] Angle between the mean planes through the substituted cyclopentadienyl (Cp^{R}) rings. [d] Torsion about the Cp^{P}(centroid)···Fe···Cp^{C}(centroid) axes.

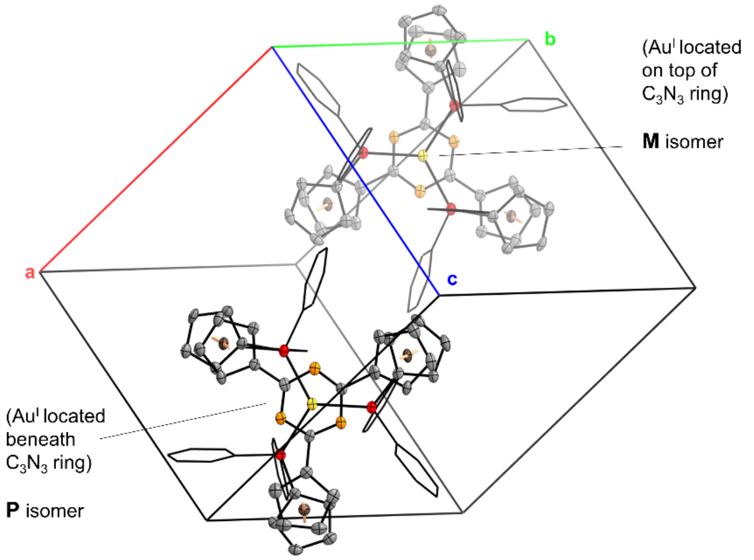

Figure S50. Exemplary depiction of the unit cell for complexes **1M** in the case of **1Au** with special emphasis on the *P* (turned away from viewer, gold beneath C_3N_3 plane) and *M* (facing the viewer, gold above C_3N_3 plane) enantiomers both being present in the unit cell due to the inversion centre. Thermal displacement ellipsoids shown at the 50% probability level. For clarity, P-bound phenyl rings are depicted in wireframe style, and solvent molecules, anions, and H atoms are not shown.

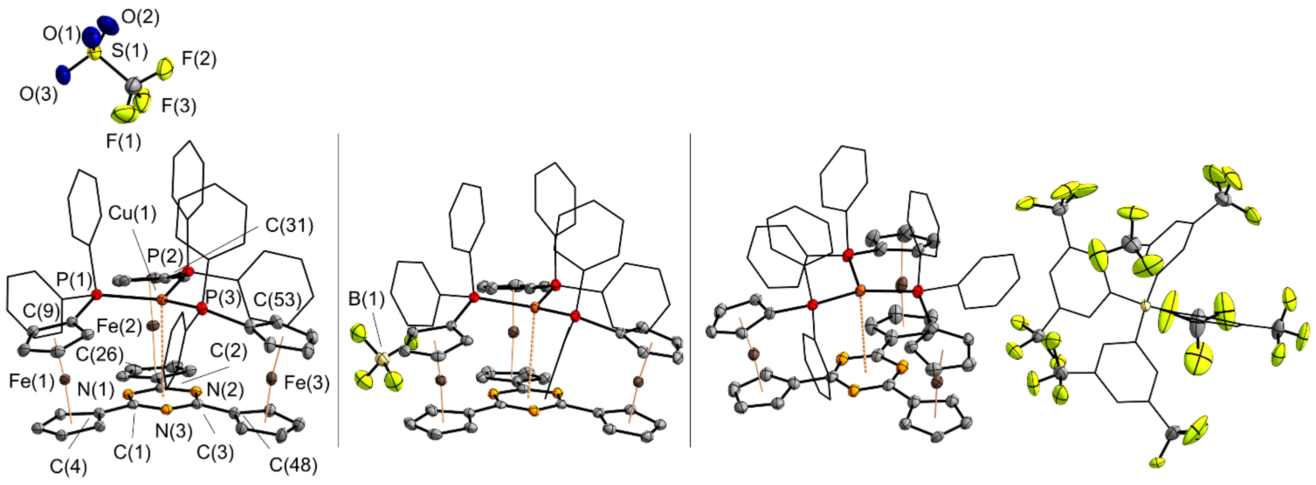

Figure S51. Molecular structures of **1Cu** (left), **1CuBF$_4$** (middle), and **1CuBAr^F$_4$** (right) and exemplary atom labelling scheme for **1Cu** with thermal displacement ellipsoids shown at the 50% probability level. For clarity, *P*- and *B*-bound phenyl rings are depicted in wireframe style, and solvent molecules, disorder and H atoms are not shown.

The three copper(I) complexes **1Cu**, **1CuBF$_4$**, and **1CuBAr^F$_4$** (Figure S51) share their key structural parameters (cf. Table S5), thus not revealing any anion influence in their solid-state structures. **1CuBAr^F$_4$** crystallises in the monoclinic space group $P2_1/c$, in contrast to both **1Cu** and **1CuBF$_4$** which both crystallise in the monoclinic space group $P\bar{1}$, most likely due to the significantly changed spatial requirements of the $BAr^F{}_4{}^-$ anion.

Table S5. Selected bond lengths [Å], distances [Å], and angles [°] of complexes **1Cu**, **1CuBF$_4$**, and **1CuBAr^F$_4$**. Numbering scheme according to the respective depiction of the solid-state structures (cf. Figure S51).

	1Cu	1CuBF$_4$	1CuBAr^F$_4$		1Cu	1CuBF$_4$	1CuBAr^F$_4$
C(9)–P(1) /<br>C(31)–P(2) /<br>C(53)–P(3)	1.803(2) /<br>1.792(3) /<br>1.807(3)	1.803(3) /<br>1.801(3) /<br>1.798(3)	1.801(3) /<br>1.807(3) /<br>1.795(3)	P(1)–Cu(1)–P(2) /<br>P(2)–Cu(1)–P(3) /<br>P(3)–Cu(1)–P(1)	117.14(3) /<br>117.64(3) /<br>120.35(3)	120.67(3) /<br>116.79(3) /<br>117.64(3)	120.01(3) /<br>117.18(3) /<br>118.03(3)
C(1)–C(4) /<br>C(2)–C(26) /<br>C(3)–C(48)	1.471(4) /<br>1.459(3) /<br>1.459(3)	1.466(4) /<br>1.454(3) /<br>1.474(4)	1.452(5) /<br>1.468(4) /<br>1.456(4)	Ct^C(1)–Fe(1)–Ct^P(1) /<br>Ct^C(2)–Fe(2)–Ct^P(2) /<br>Ct^C(3)–Fe(3)–Ct^P(3)<br>(α)	173.20 /<br>171.94 /<br>172.91	173.76 /<br>173.83/<br>172.59	173.13 /<br>173.73 /<br>173.31
P(1)–Cu(1) /<br>P(2)–Cu(1) /<br>P(3)–Cu(1)	2.3266(7) /<br>2.3178(7) /<br>2.3338(6)	2.3324(7) /<br>2.3418(6) /<br>2.3245(7)	2.340(1) /<br>2.3444(9) /<br>2.3316(8)	Θ(fc(1)) / Θ(fc(2)) /<br>Θ(fc(3))[c]	7.49 / 8.15 /<br>6.79	6.63 / 5.72 /<br>7.12	7.21 / 6.63 /<br>7.16
Ct^C(1)–Fe(1) /<br>Ct^C(2)–Fe(2) /<br>Ct^C(3)–Fe(3)[a]	1.652 / 1.660 /<br>1.663	1.664 / 1.660 /<br>1.657	1.658 / 1.655 /<br>1.666	τ(fc(1)) / τ(fc(2)) /<br>τ(fc(3))[d]	22.33 / 24.03 /<br>20.22	21.43 / 21.94 /<br>21.69	21.25 / 20.78 /<br>25.89
Ct^P(1)–Fe(1) /<br>C^{tP}(2)–Fe(2) /<br>C^{tP}(3)–Fe(3)[b]	1.662 / 1.646 /<br>1.655	1.652 / 1.651 /<br>1.647	1.656 / 1.651 /<br>1.656				

[a] Ct^C denotes the calculated centre of gravity of the carbon-substituted cyclopentadienyl ring. [b] Ct^P denotes the calculated centre of gravity of the phosphorus-substituted cyclopentadienyl ring. [c] Angle between the mean planes through the substituted cyclopentadienyl (Cp^R) rings. [d] Torsion about the Cp^P(centroid)···Fe···Cp^C(centroid) axes.

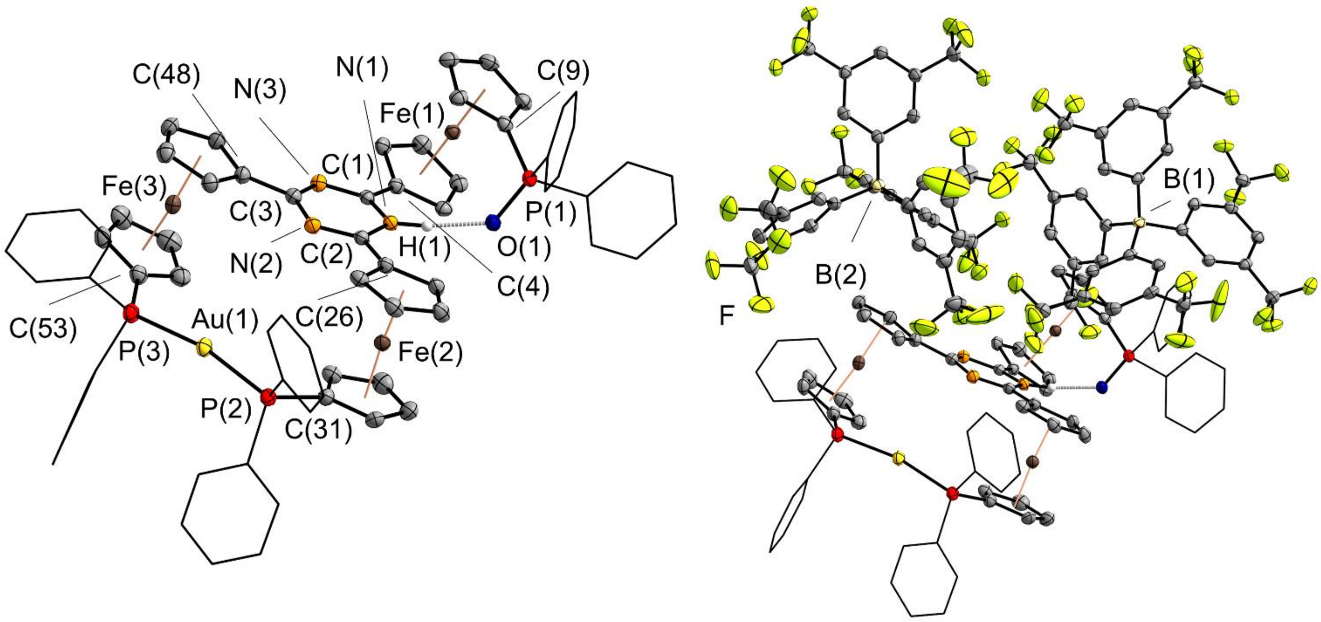

Figure S52. Molecular structure and corresponding labelling of **5** without (left) and including (right) the two $BAr^{F}_{4}{}^{-}$ anions present in the asymmetric unit. Thermal displacement ellipsoids are shown at the 50% probability level. For clarity, *P*-bound phenyl rings are depicted in wireframe style, and solvent molecules as well as H atoms with the exception of H(1) are not shown.

Table S6. Selected bond lengths [Å], distances [Å], and angles [°] of complexes **1Au** and its oxidation product **5**. Numbering scheme according to the respective depiction of the solid-state structures (cf. Figure 2 in the main article and Figure S52).

	**1Au**	**5**		**1Au**	**5**
C(9)–P(1) / C(31)–P(2) / C(53)–P(3)	1.798(6) / 1.802(7) / 1.789(7)	1.782(3) / 1.781(3) / 1.778(3)	P(1)–O(1) / O(1)···H(1) / O(1)···N(1) / N(1)–H(1) / N(1)–H(1)···O(1)	–	1.500(2) / 1.72(4) / 2.678(3) / 0.96(4) / 172(3)
C(1)–C(4) / C(2)–C(26) / C(3)–C(48)	1.472(9) / 1.451(8) / 1.459(8)	1.442(4) / 1.449(4) / 1.452(4)	P(1)–Au(1)–P(2) / P(2)–Au(1)–P(3) / P(3)–Au(1)–P(1)	119.63(6) / 118.48(6) / 117.28(6)	– / 168.08(3) / –
P(1)–Au(1) / P(2)–Au(1) / P(3)–Au(1)	2.393(2) / 2.402(1) / 2.393(2)	– / 2.302(1) / 2.298(1)	Ct^C(1)–Fe(1)–Ct^P(1) / Ct^C(2)–Fe(2)–Ct^P(2) / Ct^C(3)–Fe(3)–Ct^P(3) (α)	173.55 / 173.22 / 172.57	177.26 / 176.23 / 174.95
Ct^C(1)–Fe(1) / Ct^C(2)–Fe(2) / Ct^C(3)–Fe(3)[a]	1.659 / 1.668 / 1.654	1.649 / 1.651 / 1.658	Θ(fc(1)) / Θ(fc(2)) / Θ(fc(3))[c]	7.60 / 6.31 / 7.34	1.42 / 2.73 / 4.52
Ct^P(1)–Fe(1) / C^{tP}(2)–Fe(2) / C^{tP}(3)–Fe(3)[b]	1.649 / 1.667 / 1.647	1.647 / 1.650 / 1.649	τ(fc(1)) / τ(fc(2)) / τ(fc(3))[d]	23.13 / 22.43 / 24.58	67.41 / 93.65 / 88.86
C(1)–N(1) / N(1)–C(2) / C(2)–N(2) / N(2)–C(3) / C(3)–N(3) / N(3)–C(1) /	1.345(7) / 1.346(8) / 1.349(7) / 1.341(7) / 1.348(8) / 1.330(7)	1.366(4) / 1.362(3) / 1.325(4) / 1.348(4) / 1.351(4) / 1.319(4)	C(1)–N(1)–C(2)–N(2) / N(1)–C(2)–N(2)–C(3) / C(2)–N(2)–C(3)–N(3) / N(2)–C(3)–N(3)–C(1) / C(3)–N(3)–C(1)–N(1) / N(3)–C(1)–N(1)–C(2)	3.0(9) / −3.9(9) / 1.6(9) / 1.3(9) / −2.3(9) / 0.4(9)	−3.9(4) −2.2(4) 7.3(4) −5.6(4) −1.2(4) 5.7(4)

[a] Ct^C denotes the calculated centre of gravity of the carbon-substituted cyclopentadienyl ring. [b] Ct^P denotes the calculated centre of gravity of the phosphorus-substituted cyclopentadienyl ring. [c] Angle between the mean planes through the substituted cyclopentadienyl (Cp^R) rings. [d] Torsion about the Cp^P(centroid)···Fe···Cp^C(centroid) axes, given as its modulus.

Comparing oxidation product **5** to its starting material **1Au** is very instructive. The two $BAr^F{}_4{}^-$ anions compensate the positive charges located at the gold(I) ion and the protonated triazine core (N1) and not, as could have been expected from the oxidation of **1Au**, positive charges located at any of the iron centres. This is backed by the $Ct^{C/P}$–Fe distances (Table S6) which do not differ strongly between **1Au** and **5** and among themselves as would be the case if any of the iron centres in **5** would be in oxidation state +III.

The most obvious change relates to the change of denticity from 3 in **1Au** to only 2 in **5**; accordingly, the P–Au–P angles change from just below 120° in **1Au** to 168° in **5**, in line with the loss of the C_3N_3···Au(I) interaction in **1Au**. The deviation of the ideal linear binding mode (180°) is most likely due to a weak interactions between N(2) and Au(1) (d = 4.412(2) Å). This bidentate mode seems to put slightly less strain on the general geometry of ligand **1** than the tricoordinate mode found in **1M**, as the more relaxed values of Θ (closer to 0°) and α (closer to 180°) suggest. The P–O distance of 1.500(2) Å fits well with a previously reported value (1.4866(9) Å) for 1-diphenylphosphoryl-1'-cyanoferrocene, as do the other relevant structural parameters, confirming the phosphoryl moiety in **5**.[29] Relating to the hydrogen bond in **5** between the phosphoryl group and the protonated triazine, all its parameters fall within the expected range,[30] allowing it to be classified as a heteronuclear resonance-assisted hydrogen bond (RAHB), given the ferrocenylene group is treated as a resonant spacer in the sense of Gilli.[31] It is worth noting that the protonation of N(1) and its involvement in hydrogen bonding lead to a distortion of the otherwise regular C_3N_3 geometry that can be observed in **1Au**. The C(1/2)–N(1) bonds become elongated, while the other non-involved C–N bonds shorten; in the same way, the torsion angles in the C_3N_3 ring increase (7.3° to −5.6° in **5** *vs.* 3.0° to −3.9° in **1Au**). This puckering is also observable in N(1) moving away from a calculated C_3N_2 plane by 0.07 Å and from H(1) being away from a calculated C_3N_3 plane by 0.253 Å.

2.2. CSD Searches and Results for Comparison

In the following tables (Table S7 – Table S9), molecular structures of trigonal-planar coinage metal complexes with three phosphane ligands are assembled and their individual and average P–M–P bond angles β and <β> as well as their individual and average P–M bond lengths are listed. For the CSD search, the number of ligands on the metal M was not restricted. No restraints on the P–M bond lengths were set. The three bond angles β were set to a range of 115 to 125 ° each, to retrieve only trigonal-planar structures. Metal clusters and ligands other than phosphanes (*i.e.*, phosphanides and (aromatic) phosphacycles) were manually sorted out. Additional donor atoms or groups in close contact to the metal M are indicated, and the column "1:1 Complex" is checked when a tris-phosphane ligand was found to bind the metal.
The number of such complexes is obviously rather small given the vast number of (phosphane) complexes of the coinage metals. As becomes apparent, trigonal-planar 1:1 complexes are even rarer and seem to require the presence of a supporting apical ligand or group (*e.g.*, an arene).

Table S7. Comparison of solid-state molecular structures of trigonal-planar tris-phosphane Cu^I complexes regarding (and sorted according to) their Cu–P bond lengths and P–Cu–P bond angles $β_{1–3}$ (set to 115–125°). Only trivalent phosphane donors were considered, *i.e.* phosphacycles and phosphanides are not part of this overview. CSD entries with more than one CuP_3 unit in the same solid-state structure are labelled with (1) and (2).

CSD Identifier Code	$β_1$ [°]	$β_2$ [°]	$β_3$ [°]	<β> [°]	P(1)–Cu [Å]	P(2)–Cu [Å]	P(3)–Cu [Å]	<P–Cu> [Å]	Apical atom(s)	1:1 Complex
**PUDVUG**	120.179	115.007	120.179	118.46	2.220	2.226	2.226	2.224	I^-	-
**FAYYEK (1)**	118.745	119.525	121.186	119.82	2.233	2.238	2.239	2.237	-	-
**WEWKIU**	115.597	115.597	115.597	115.60	2.239	2.239	2.239	2.239	I^-	-
**RAZHEI**	120.23	119.498	119.904	119.88	2.239	2.245	2.237	2.240	-	-
**MEZXAS**	118.557	121.937	117.034	119.18	2.238	2.241	2.255	2.245	B^-	X
**TONSAQ (1)**	121.617	121.238	116.57	119.81	2.249	2.232	2.253	2.245	-	-
**AHUKAP (1)**	118.408	120.093	121.221	119.91	2.239	2.250	2.246	2.245	-	-
**FAYYEK (2)**	120.124	118.113	121.667	119.97	2.248	2.246	2.252	2.249	-	-
**SEFRIH**	117.979	115.232	115.232	116.15	2.250	2.250	2.246	2.249	I^-	-
**WEMNAD**	119.507	122.353	116.654	119.50	2.266	2.258	2.249	2.258	N	X
**AHUKAP (2)**	121.166	117.646	121.174	120.00	2.257	2.257	2.261	2.258	-	-
**MEZWOF**	117.892	121.201	119.935	119.68	2.271	2.265	2.275	2.270	B	X
**TONSAQ (2)**	119.218	117.356	121.236	119.27	2.266	2.274	2.277	2.272	-	-
**FEPVUU**	122.58	118.45	117.751	119.59	2.272	2.268	2.284	2.275	-	-
**UKOKOV**	116.996	120.091	122.755	119.95	2.257	2.291	2.280	2.276	-	-
**UJITIR**	116.512	117.587	116.963	117.02	2.281	2.274	2.276	2.277	O^-	-
**EDICOL**	123.983	119.124	116.765	119.96	2.279	2.265	2.288	2.277	-	-
**QOZHOC (1)**	118.261	124.151	117.485	119.97	2.282	2.262	2.294	2.279	-	-
**OKAXOP**	115.836	118.725	123.524	119.36	2.288	2.280	2.273	2.280	-	-
**QOZHOC (2)**	120.263	122.307	116.792	119.79	2.281	2.263	2.299	2.281	-	-
**SUXCIX**	119.991	119.991	119.991	119.99	2.286	2.286	2.286	2.286	-	-
**WIMLEK**	121.025	119.086	119.783	119.96	2.278	2.292	2.29	2.287	-	-
**XOCNIO (1)**	120.252	120.132	118.879	119.75	2.278	2.290	2.297	2.288	Al	X[a]
**LAXKIE**	117.933	123.156	117.005	119.36	2.303	2.287	2.293	2.294	-	-
**TPCUFB (1)**	115.213	115.215	115.208	115.21	2.295	2.295	2.295	2.295	-	-
**DEZYUC**	119.965	122.569	117.464	120.00	2.293	2.296	2.297	2.295	-	-

**ZEPYEY**	123.495	115.035	121.261	119.93	2.291	2.302	2.294	2.296	-	-
**TPCUFB (2)**	115.894	115.894	115.894	115.89	2.296	2.296	2.296	2.296	-	-
**XOCNIO (2)**	118.803	119.321	121.288	119.80	2.291	2.293	2.305	2.296	Al	X[a]
**VIVPUL**	119.026	119.015	119.091	119.04	2.301	2.303	2.301	2.302	-	-
**TPCUFB (3)**	115.819	115.814	115.812	115.82	2.302	2.302	2.302	2.302	-	-
**EDIDAY**	119.851	120.775	115.658	118.76	2.314	2.310	2.317	2.314	-	-
**FAKLEI**	115.599	115.667	115.613	115.63	2.318	2.317	2.316	2.317	O^-	-
**FISGOE**	115.619	123.561	120.476	119.89	2.344	2.297	2.33	2.324	O	-
**XOMTOK**	116.252	116.24	116.268	116.25	2.329	2.330	2.330	2.330	I^-	-
**RIRYUM**	116.745	115.754	116.336	116.28	2.349	2.345	2.348	2.347	O^-	X
**QOSWEB**	117.15	117.213	117.155	117.17	2.359	2.358	2.358	2.358	B, Cl^-	X

[a] Even though the CSD does not recognise the Al–Cu^I distances as signifier of a bond, the group of Bourissou presents convincing arguments for treating the Al–Cu^I distance of 3.004(2) Å as a weak Z-type interaction.[32]

Table S8. Comparison of solid-state molecular structures of trigonal-planar tris-phosphane Ag^I complexes regarding (and sorted according to) their Ag–P bond lengths and P–Ag–P bond angles β_{1-3} (set to 115–125°). Only trivalent phosphane donors were considered, *i.e.* phosphacycles and phosphanides are not part of this overview. CSD entries with more than one AgP_3 unit in the same solid-state structure are labelled with (1) and (2).

CSD Identifier Code	β_1 [°]	β_2 [°]	β_3 [°]	<β> [°]	P(1)–Ag [Å]	P(2)–Ag [Å]	P(3)–Ag [Å]	<P–Ag> [Å]	Apical atom(s)	1:1 Complex
**FAYYIO**	119.693	122.156	117.879	119.91	2.44	2.425	2.438	2.434	Ag^+	X[a]
**POPVIZ**	120.209	121.811	117.353	119.79	2.443	2.443	2.467	2.451	-	-
**QAHKAL (1)**	120.328	120.171	119.428	119.98	2.45	2.442	2.461	2.451	-	-
**PIFYUY**	115.392	115.392	115.392	115.39	2.463	2.463	2.463	2.463	N[b]	X
**REDHUE**	118.712	118.832	119.675	119.07	2.461	2.457	2.473	2.464	-	-
**HAJTOC**	116.962	117.203	118.325	117.50	2.458	2.463	2.470	2.464	O^-	-
**QAHKAL (2)**	117.674	118.349	121.698	119.24	2.456	2.467	2.472	2.465	-	-
**HEJXEZ**	122.894	115.655	119.639	119.40	2.455	2.462	2.484	2.467	-	-
**TUGTUK**	116.494	121.02	122.466	119.99	2.483	2.476	2.457	2.472	Ag^+	X[a]
**FUJQIM (1)**	120.745	117.733	121.472	119.98	2.484	2.488	2.479	2.484	Ag^+	X[a]
**IBOQIZ**	115.611	116.575	117.447	116.54	2.503	2.478	2.495	2.492	-	-
**QOSWAX**	116.665	116.719	116.66	116.68	2.500	2.498	2.499	2.499	B, Cl^-	X
**MUCVOV**	120.963	123.525	115.472	119.99	2.522	2.509	2.498	2.510	Ag^+	X[a]
**RIRZAT**	116.218	117.06	116.579	116.62	2.519	2.510	2.517	2.515	O^-	X
**FUJQIM (2)**	116.839	121.871	121.234	119.98	2.523	2.518	2.533	2.525	Ag^+	X[a]
**HIBCUQ**	117.515	123.24	116.943	119.23	2.533	2.522	2.531	2.529	-	-
**KIGZAB**	117.395	117.391	117.396	117.39	2.544	2.544	2.544	2.544	F[c]	-

[a] In these cases, the silver(I) ion can be considered to form part of a metalloligand (**FAYYIO**,[33] **TUGTUK**,[34] **MUCVOC**,[35] **FUJQIM** [36]). [b] The authors state that "the N atom is non-interactive at 2.662 Å",[37] the lone pair of electrons at nitrogen is directed towards the silver(I) ion and the distance is significantly below the sum of the van der Waals radii (3.27–4.19 Å),[38] yet also clearly above the sum of the covalent radii (2.156 Å).[39] [c] Part of a disordered tetrafluoroborate anion; the authors state a 50% probability of the Ag–F bond.[40]

Table S9. Comparison of solid-state molecular structures of trigonal-planar tris-phosphane Au^I complexes regarding (and sorted according to) their Au–P bond lengths and P–Au–P bond angles $\beta_{1–3}$ (set to 115–125°). Only trivalent phosphane donors were considered, *i.e.* phosphacycles and phosphanides are not part of this overview. CSD entries with more than one AuP_3 unit in the same solid-state structure are labelled with (1) and (2).

CSD Identifier Code	β_1 [°]	β_2 [°]	β_3 [°]	<β> [°]	P(1)–Au [Å]	P(2)–Au [Å]	P(3)–Au [Å]	<P–Au> [Å]	Apical atom(s)	1:1 Complex
**IZOGAF**	119.736	118.499	119.214	119.15	2.337	2.345	2.316	2.333	Tl^+	X[a]
**TIPZEX**	117.965	117.965	117.965	117.97	2.334	2.334	2.334	2.334	$I^–$	-
**IKASIY**	119.595	119.551	119.638	119.59	2.338	2.340	2.339	2.339	-	-
**IKASOE**	117.300	120.450	119.797	119.18	2.348	2.351	2.334	2.344	-	-
**DUVYUO (1)**	121.543	120.659	117.795	120.00	2.345	2.342	2.356	2.348	Au^+	X[a]
**VUSJEY (1)**	122.087	122.102	115.789	119.99	2.366	2.338	2.355	2.353	-	-
**DUVYUO (2)**	120.079	117.601	122.298	119.99	2.349	2.354	2.358	2.354	Au^+	X[a]
**PIFYEI**	116.412	117.305	117.281	117.00	2.367	2.351	2.354	2.357	N	X[b]
**YAZTEY**	119.072	119.072	117.431	118.53	2.362	2.352	2.362	2.359	Au^+	-
**SEKVOT**	118.703	118.69	118.649	118.68	2.361	2.360	2.362	2.361	-	X[c]
**PIFYIM**	116.931	118.148	115.372	116.82	2.369	2.361	2.362	2.364	N	X[b]
**POPVEV**	120.796	115.827	121.23	119.28	2.357	2.368	2.37	2.365	-	-
**HOVBAV (1)**	116.062	123.168	117.786	119.01	2.372	2.361	2.363	2.365	Na^+	-
**SEKSOQ**	120.501	120.583	115.790	118.96	2.367	2.364	2.367	2.366	-	X[c]
**VUSJEY (2)**	118.055	118.578	121.967	119.53	2.357	2.384	2.362	2.368	-	-
**HOVBAV (2)**	119.377	120.273	117.564	119.07	2.366	2.370	2.369	2.368	Na^+	-
**LODNUN (1)**	116.963	120.297	120.787	119.35	2.371	2.368	2.37	2.370	-	-
**NAVTAG**	124.611	115.426	117.492	119.18	2.366	2.351	2.392	2.370	$Cl^–$	-
**MIPBAO**	120.102	120.436	119.334	119.96	2.369	2.369	2.374	2.371	-	-
**YIQKIU**	115.044	115.044	115.044	115.04	2.372	2.372	2.372	2.372	Sn	-
**AVACUV**	119.978	119.978	119.978	119.98	2.374	2.374	2.374	2.374	Ag^+	X[a]
**NODZUD**	119.975	120.004	120.018	120.00	2.374	2.375	2.374	2.374	-	-
**ZETBIJ (1)**	123.897	119.747	116.353	120.00	2.376	2.365	2.383	2.375	-	-
**HOTZUL (1)**	116.68	123.773	118.068	119.51	2.381	2.368	2.377	2.375	Tl^+	X[a]
**PABDOO**	116.548	118.734	124.409	119.90	2.369	2.390	2.367	2.375	-	-
**RABDEE**	119.395	119.446	119.443	119.43	2.376	2.376	2.375	2.376	-	-
**WUHSID**	119.482	119.482	119.482	119.48	2.378	2.378	2.378	2.378	Si	X[d]
**XOCNEK (1)**	120.619	119.018	119.291	119.64	2.376	2.370	2.388	2.378	Al	X[d]
**LODNUN (2)**	122.232	116.725	118.573	119.18	2.377	2.371	2.387	2.378	-	-
**LOFRON**	115.077	124.420	120.347	119.95	2.383	2.372	2.38	2.378	$Cl^–$	-
**ZETBIJ (2)**	121.518	118.238	120.244	120.00	2.375	2.382	2.378	2.378	-	-
**NIWKOV**	119.998	115.181	121.956	119.05	2.359	2.403	2.382	2.381	-	-
**HOTZUL (2)**	118.848	120.150	120.181	119.73	2.38	2.388	2.381	2.383	Tl^+	X[a]
**ROZREE (1)**	120.818	119.219	118.653	119.56	2.376	2.384	2.39	2.383	Ga	X[d]
**XOCNEK (2)**	118.648	121.469	118.968	119.70	2.392	2.373	2.39	2.385	Al	X[d]

**KIGWAY**	115.016	120.908	116.271	117.40	2.416	2.336	2.409	2.387	P	-
**OMUGAG (1)**	118.647	120.532	120.766	119.98	2.393	2.385	2.387	2.388	Au^+	X[a]
**ROZREE (2)**	118.922	121.605	118.375	119.63	2.39	2.393	2.383	2.389	Ga	X[d]
**QOSWIF**	118.364	118.302	120.114	118.93	2.388	2.387	2.392	2.389	B	X[d]
**OMUGAG (2)**	121.383	120.512	118.042	119.98	2.391	2.389	2.389	2.390	Au^+	X[a]
**ATIRIF**	119.111	120.684	119.624	119.81	2.390	2.390	2.392	2.391	In	X[d]
**BACVOQ01**	118.575	117.609	115.289	117.16	2.406	2.387	2.380	2.391	S	-
**PABDII (1)**	117.398	117.039	119.316	117.92	2.394	2.408	2.379	2.394	Cl^-	-
**PABDII (2)**	117.88	118.081	116.748	117.57	2.393	2.396	2.403	2.397	Cl^-	-
**AUBPHA**	116.017	119.259	121.431	118.90	2.408	2.406	2.392	2.402	-	-
**OMUFUZ (1)**	119.997	119.997	119.997	120.00	2.406	2.406	2.406	2.406	Au^+	X[a]
**OMUFUZ (2)**	119.999	119.999	119.999	120.00	2.408	2.408	2.408	2.408	Au^+	X[a]
**BOJJEP**	116.062	119.627	116.62	117.44	2.396	2.430	2.403	2.410	Cl^-	-
**MABTEP**	115.391	115.342	115.377	115.37	2.417	2.418	2.418	2.418	I^-	-
**KOTROA**	119.871	119.871	119.871	119.87	2.421	2.421	2.421	2.421	-	-
**YIXCIS**	118.48	118.48	118.481	118.48	2.424	2.424	2.424	2.424	B, Cl^-	X[d]
**RUGZID**	117.268	119.638	117.049	117.99	2.453	2.457	2.448	2.453	Ga, Cl^-	X[d]

[a] In these cases, the palladium(0) atom (**IZOGAF** [41]), the silver(I) (**AVACUV** [42]) or gold(I) ions (**DUVYUO**,[43] **HOTZUL**,[44] **OMUGAG** and **OMUFUZ** [45]) can be considered to form part of a metalloligand. [b] The authors state that there is "at most a weak interaction with the nitrogen atom of the *NP₃* ligand" considering the Au–N distance of 2.683(6) Å.[37] This distance is significantly below the sum of the van der Waals radii (3.21–3.98 Å),[38] yet also clearly above the sum of the covalent radii (2.07 Å).[39] [c] These compounds could be considered unsupported 1:1 trigonal-planar Au^I complexes if the Cu^I (**SEKSOQ**) and Cd^{II} ions (**SEKVOT**) were to be considered as part of a metalloligand.[46] [d] Even though the CSD does not recognise the element–Au^I distances as signifiers of true bonds in all cases, the group of Bourissou presents convincing arguments for treating these distances, shorter than the respective sum of the van der Waals radii in all cases (except for **ROZREE** [47]), as Z-type interactions.[32,47,48]

The following two tables, Table S10 (coinage metals) and Table S11 (all other metals), give an overview of published solid-state structures of metal complexes featuring C_3N_3···M contacts, including the respective C_3N_3···M distances and deviation angles γ. For the search in the CSD, the maximum distance between the C_3N_3-centroid and the metal was set to 4 Å and the maximum angle γ between the C_3N_3 plane normal and the C_3N_3···M vector was set to 20° in accordance with the definition of delocalised C_3N_3···M interactions by Tiekink and co-workers.[49]

Table S10. Overview of published solid-state molecular structures of coinage-metal complexes featuring C_3N_3···M contacts alongside (and sorted according to) their respective C_3N_3···M distances (≤ 4 Å) and the angles between their C_3N_3···M axis and the normal of the C_3N_3 plane γ (γ ≤20°). The coordination number of the metal C.N. is given without considering the C_3N_3 ring as additional donor. All contacts are observed between discrete molecules or within polymeric structures.

CSD Identifier Code	C_3N_3···M [Å]	γ [°]	M	C. N.
**WIPDOQ**	3.763	5.80	Ag	5
**IKUHUT**	3.746	18.44	Cu	4
**VOBTAJ**	3.746	18.35	Cu	4
**VOBTAJ02**	3.745	18.38	Cu	4
**ZOTFEV**	3.722	13.80	Cu	4
**HEBDAV01**	3.678	16.65	Ag	3
**RIGCOB**[a]	3.570	15.05	Ag	2
**RIGCER**[a]	3.511	10.70	Au	2

[a] These two related structures mark the only cases in which a C_3N_3···M contact closer than in our system has been described.[50] For both copper(I) and silver(I), no shorter contact has been published to the best of our knowledge. In no case has an intramolecular C_3N_3···M contact been disclosed so far.

Table S11. Overview of published solid-state molecular structures of non-coinage metal complexes featuring C_3N_3···metal contacts alongside (and sorted according to) their respective C_3N_3···M distances d (d_{max} = 4 Å) and the angles between their C_3N_3···M axis and the normal of the C_3N_3 plane γ (γ ≤ 20°). The coordination number of the metal (C.N.) is given without considering the C_3N_3 ring as additional donor. Intramolecular contacts (*i.e.* those that are not observed between discrete molecules or within polymeric structures) are highlighted in light grey. CSD entries with more than one C_3N_3···metal contact in the same solid-state structure are labelled with (1) and (2).

CSD Identifier Code	d [Å]	γ [°]	M	C. N.
**RECJOB**	3.996	7.64	Zn	4
**YEHBUL (1)**	3.994	14.88	W	6
**VUKYUX**	3.993	7.60	Zn	4
**YEHBUL (2)**	3.991	15.27	W	6
**YEHBOF**	3.977	5.05	W	6
**CENCAC**	3.972	5.85	Zn	4
**RECHUF**	3.970	6.13	Zn	4
**CENCIK**	3.967	8.53	Zn	4
**RECJAN**	3.962	8.83	Zn	4
**IYOHOU**	3.955	7.25	Zn	4
**RECJER (1)**	3.931	5.47	Zn	4
**RECJER (2)**	3.930	7.94	Zn	4
**RECKAO**	3.901	5.89	Zn	4
**CENBIJ**	3.877	4.52	Zn	4
**WASQAJ (1)**[a]	3.560	16.15	Pt	4
**PADVUO**	3.535	15.87	Pd	4
**KEGQAQ (1)**[b]	3.533	12.84	Pt	4
**WASQAJ (2)**[a]	3.530	14.04	Pt	4
**KEGQAQ (2)**[b]	3.497	6.63	Pt	4
**POZJOE (1)**[c]	3.458	18.51	Ni	4
**POZJOE (2)**[c]	3.427	15.79	Ni	4
**PIZRAS (1)**[d]	3.414	5.41	Pt	4
**PIZRAS (2)**[d]	3.397	2.87	Pt	4

[a] Platinum is, as two stacked (d(Pt–Pt) = 3.32 Å) bis(acetylacetonato)platinum(II) ions, encapsulated in a C_3-symmetric coordination cage; the authors do not comment on the close C_3N_3···Pt distances.[51] [b] Platinum is, as one discrete molecule of bis(acetylacetonato)platinum(II), encapsulated in a C_3-symmetric coordination cage; the authors note the close C_3N_3···Pt distance but attribute the encapsulation mainly to donor–acceptor π···π stacking interactions.[52] [c] Nickel is, as two stacked molecules of (*N*,*N*-ethylenebis(acetylacetoneiminato))nickel(II), encapsulated in a C_3-symmetric coordination cage; the authors do not comment on the close C_3N_3···Ni distances.[53] [d] Platinum is, as one discrete molecule of bis(acetylacetonato)platinum(II), encapsulated in a C_3-symmetric coordination cage; the authors note the small C_3N_3···Pt separation but do not link it to the successful encapsulation.[54]

2.3. VT NMR Studies

Variable-temperature ^{1}H and $^{31}P\{^{1}H\}$ NMR studies for all complexes have been conducted in the temperature ranges according to solvent and/or solubility limitations. For **1Ag** and **1Au**, the breakdown from C_{3v} to C_3 symmetry due to the inversion of helicity becoming slow at the NMR timescale can be followed nicely. For **1Cu**, the high-temperature limit spectra in CD_2Cl_2 and CD_3CN suggest C_{3v} symmetry, while cooling down reveals a slow equilibrium between the C_3-symmetric **Cu$_{cl}$** and a bidentate, open form **Cu$_{op}$** No significant anion influence can be discerned going from **1Cu** over **1CuBF$_4$** to **1CuBAr^F$_4$** in CD_2Cl_2.

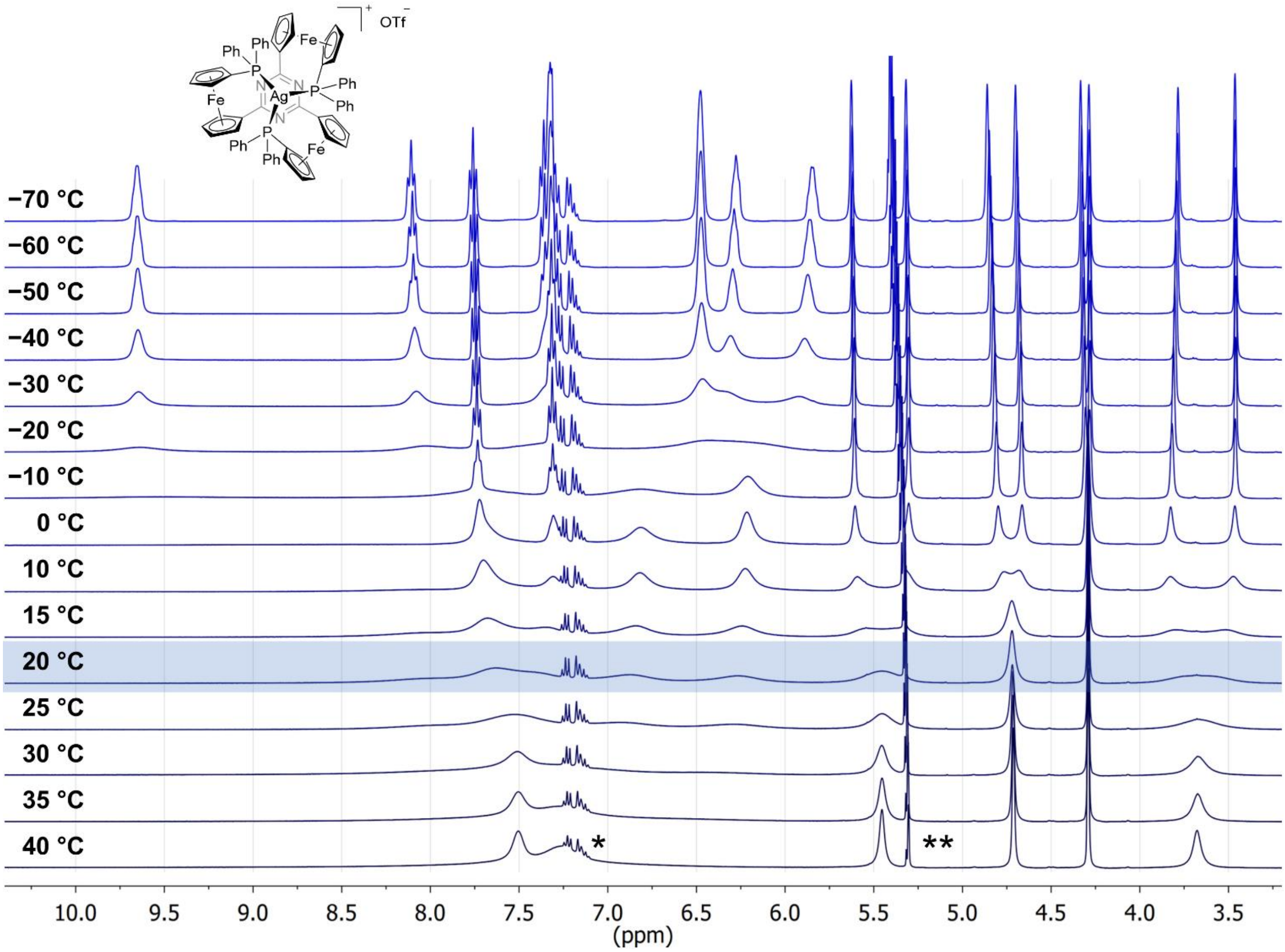

Figure S53. Stacked VT ^{1}H NMR spectra of **1Ag** in CD_2Cl_2 over a temperature range from 40 °C to −70 °C. The asterisks denote toluene (*) and the residual $CHDCl_2$ (**) solvent signals. The coalescence-temperature spectrum is highlighted in light blue.

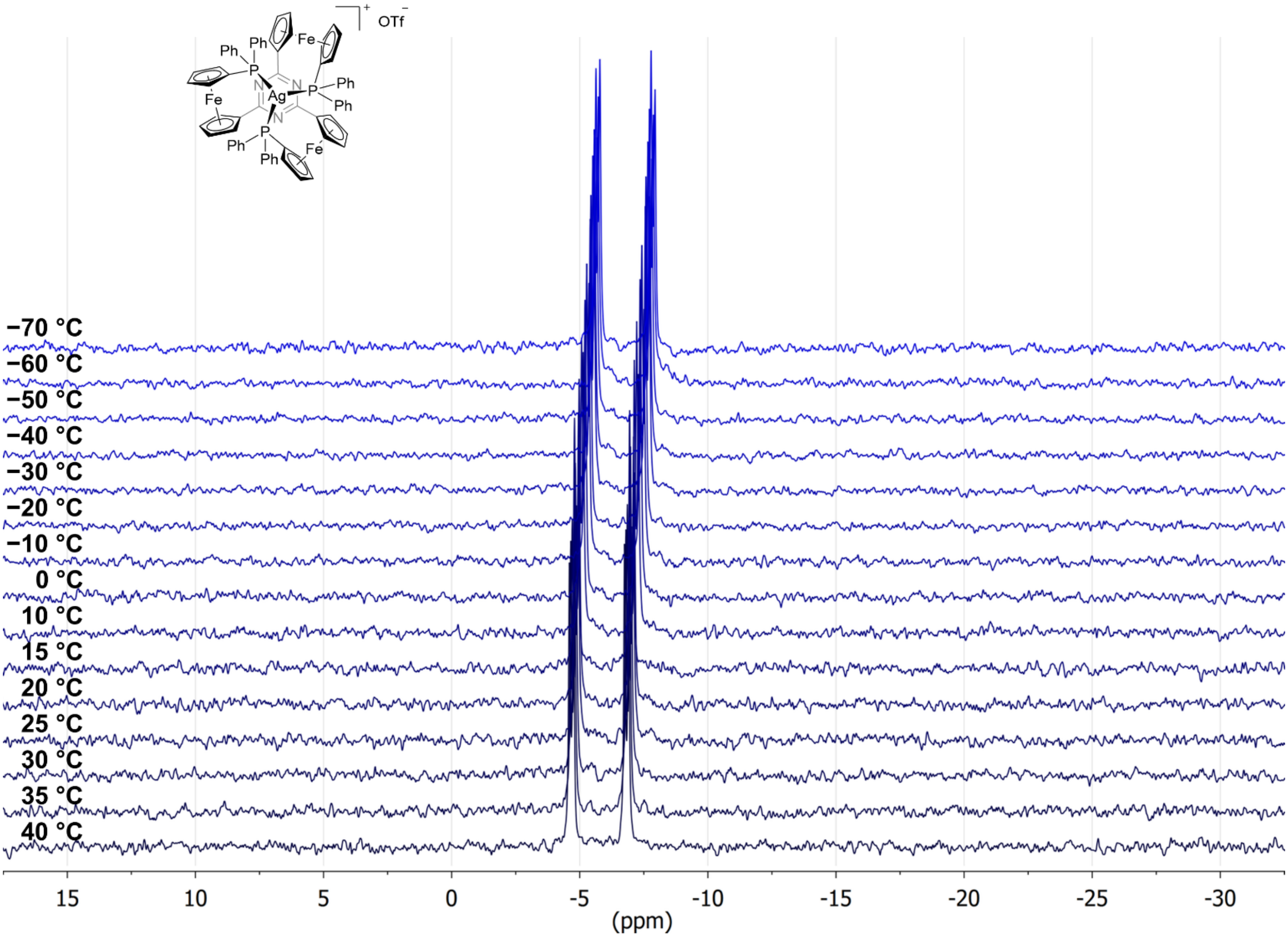

Figure S54. Stacked VT ^{31}P{^{1}H} NMR spectra (exponential lb = 10 Hz) of **1Ag** in CD_2Cl_2 over a temperature range from 40 °C to −70 °C. No changes in coordination mode are observed.

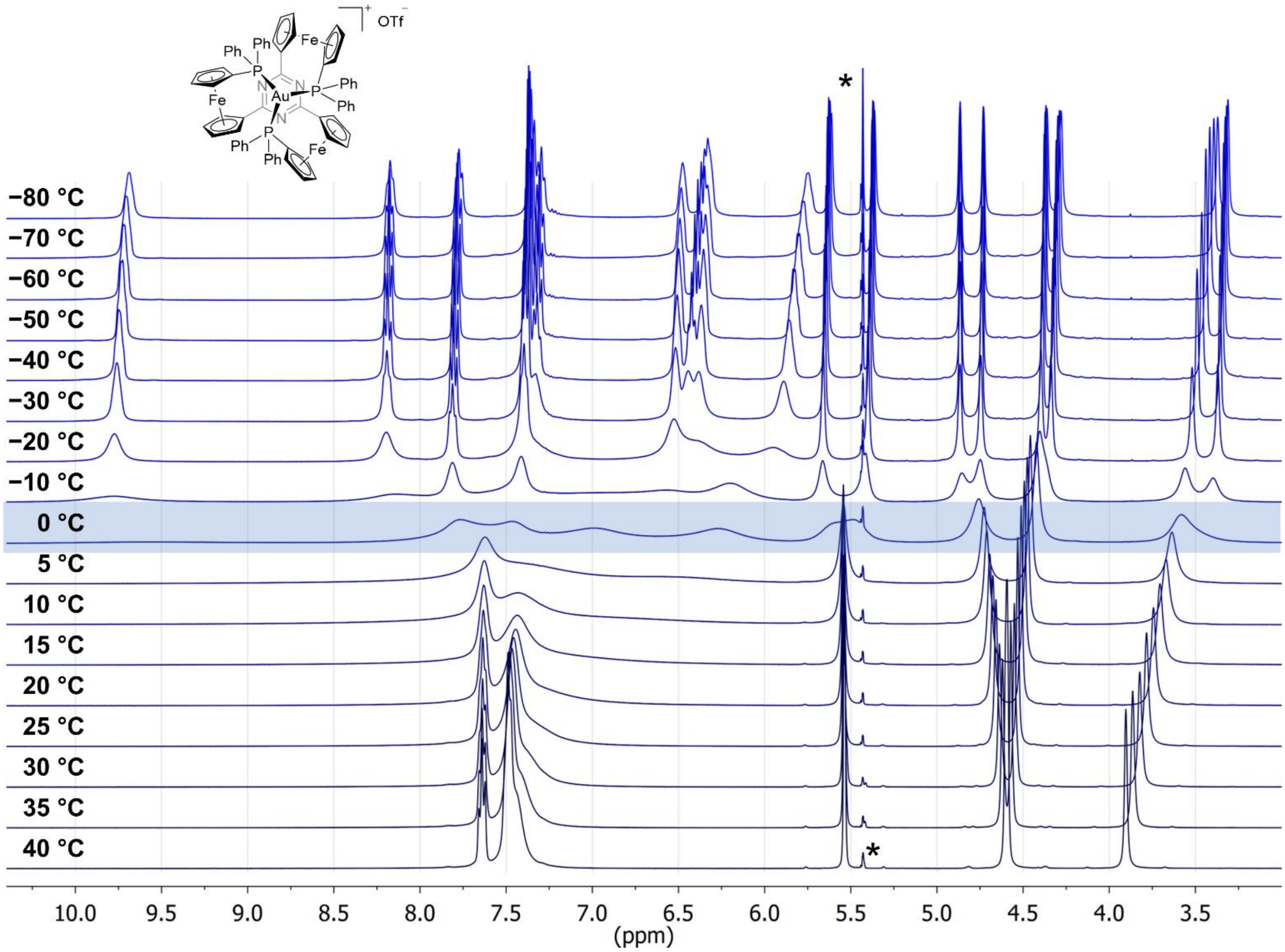

Figure S55. Stacked VT ^{1}H NMR spectra of **1Au** in CD_2Cl_2 over a temperature range from 40 °C to −80 °C. The asterisks denote the $CHDCl_2$ signal.

For **1Au**, a $^{1}H,^{1}H$ COSY experiment at −50 °C was carried out (Figure S57) to determine the connectivities in the phenyl rings, in order to understand the reason for the significant shielding and deshielding of certain resonances (Figure S55). The cross peaks observed in this experiment also unambiguously establish the connectivities in the substituted cyclopentadienyl rings (**v**, orange, and **w**, purple) of this complex and, given the similarities between the low-temperature ^{1}H NMR spectra, also of **1Ag** (and, to the **Cu$_{Cl}$** fraction of **1Cu**, **1CuBF$_4$**, and **1CuBAr$^F_4$**). After the two phenyl rings had been separated regarding their resonances (**x**, green, and **o**, red), DFT calculations were carried out to assign the resonances to the DFT-optimised structure (depicted as simplified model in Figure S56).

Shielding tensors were calculated for all 54 protons of **1Au** (cf. section 1.1, **Computational methods**), combined into isotropic chemical shielding (in ppm), averaged for the three protons that, due to the C_3 symmetry of the ^{1}H NMR spectra at low temperatures, are expected to be equivalent for the solution-state structure of **1Au** (standard deviations are given in brackets), and referenced to the chemical shift for TMS obtained by the same method. Results are summarised in Table S12. Since the error of the method is quite high and the chemical shifts so obtained are far from realistic values, only the general trend can be discussed. For an easier discussion of these trends, the calculated shifts have been scaled to match the experimental spectra separately for the ferrocenyl and the phenyl region. From the calculations, it is obvious that proton **x^1** yields the most deshielded resonance, while proton **o^5** appears as most shielded. Following the cross peaks showing only $^{3}J_{H,H}$ couplings for the phenyl rings, the other resonances can then be tentatively assigned to the respective protons. For the ferrocenylene moieties, both $^{3}J_{H,H}$ and $^{4}J_{H,H}$ coupling result in cross peaks, thus no unambiguous assignment is possible.

The unusual proton shifts for **x^1** (proton 1 in Figure S56) and **o^5** (proton 10 in Figure S56) are in line with their distinguished positions in close vicinity to the respective metal ions. Protons **x^1** have a mean Fe···H distance of 3.60 Å, the sum of the van der Waals radii being 3.61 Å;[38] however, this distance is far from that of a covalent bond for which distances between 1.34 Å and 1.62 Å have been experimentally determined.[55] It is interesting to note that for an Fe-bound hydride in $[Cp_2Fe(H)]PF_6$, a ^{1}H NMR chemical shift of −1.3 ppm was recorded (in HF/PF_5) , showing intense shielding in contrast to the deshielding observed for **x^1**.[55] Alternatively, the strong deshielding could thus also relate to the vicinity to the C_3N_3 nitrogen atoms (mean N···H distance 2.36 Å, sum of van der Waals radii 2.86 Å). For protons **o^5**, the mean Au···H distance amounts to 3.31 Å with the sum of the van der Waals radii equalling 3.66 Å.[38]

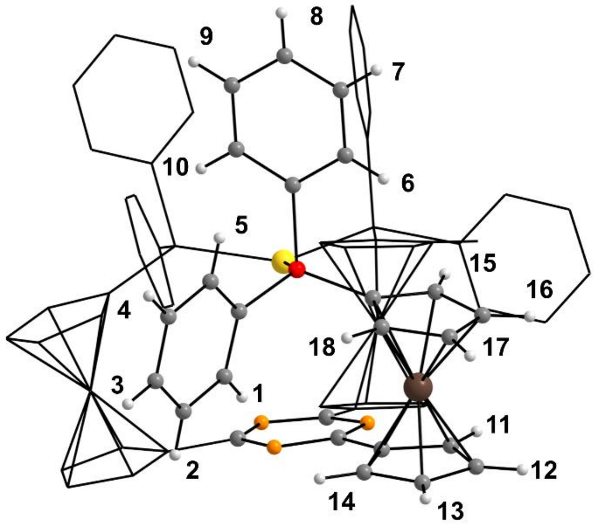

Figure S56. Optimised structure of **1Au** used for the calculation of its ^{1}H NMR shifts.

Table S12. Results of a DFT calculation of the isotropic shielding parameters for **1Au**, the corresponding chemical shifts *vs.* TMS, and a tentative assignment to the signals experimentally found through VT NMR experiments. Phenyl protons 1 – 10 are highlighted in light grey.

Proton[a]	Averaged shielding δ (± MD)[b] [ppm]	Chemical shift vs. TMS [ppm]	Scaled shift[c] [ppm]	Tentative Assignment[d] (experimental shift [ppm])
1	3.76 (±5.12)	27.96	9.62	**x¹** (9.62)
2	13.31 (± 3.12)	18.41	9.00	**x²** (8.07)
3	20.24 (± 3.29)	11.48	8.55	**x³** (7.68)
4	28.02 (±2.94)	3.70	8.05	**x⁴** (7.27)
5	39.86 (± 4.88)	-8.14	7.28	**x⁵** (6.39)
6	35.38 (± 5.22)	-3.66	7.57	**o⁴** (6.24)
7	34.12 (±3.03)	-2.40	7.65	**o²** (7.20)
8	38.53 (± 0.97)	-6.81	7.37	**o¹** (7.27)
9	44.49 (± 3.15)	-12.77	6.98	**o³** (6.30)
10	64.09 (± 6.29)	-32.37	5.72	**o⁵** (5.72)
11	15.35 (± 1.38)	16.37	5.53	**v$^{1/2}$** (5.53/5.27)
12	20.70 (± 2.71)	11.02	4.37	**v$^{3/4}$** (4.75/4.62)
13	21.10 (± 3.31)	10.62	4.29	**v$^{3/4}$** (4.75/4.62)
14	15.72 (± 4.06)	16.00	5.45	**v$^{1/2}$** (5.53/5.27)
15	22.61 (± 6.87)	9.11	3.96	**w$^{1/2}$** (4.27/4.20)
16	25.94 (± 1.94)	5.78	3.23	**w$^{3/4}$** (3.33/3.23)
17	25.55 (± 3.86)	6.17	3.32	**w$^{3/4}$** (3.33/3.23)
18	23.89 (± 4.87)	7.83	3.68	**w$^{1/2}$** (4.27/4.20)

[a] Referring to the numbering scheme shown in Figure S56. [b] Mean deviations (MD) were calculated as the deviations of the three individual shielding parameters obtained for the non-identical protons at the chemically equivalent positions. [c] Scaling was carried out separately for the phenyl (1–10) and for the ferrocenylene (11–18) resonances by setting the experimental chemical shifts as respective boundaries and solely serves for easier assignment. [d] Referring to the signals depicted in the corresponding $^{1}H,^{1}H$ COSY NMR. spectrum (Figure S57) and based on the DFT results and the connectivity inferred from the COSY NMR experiment.

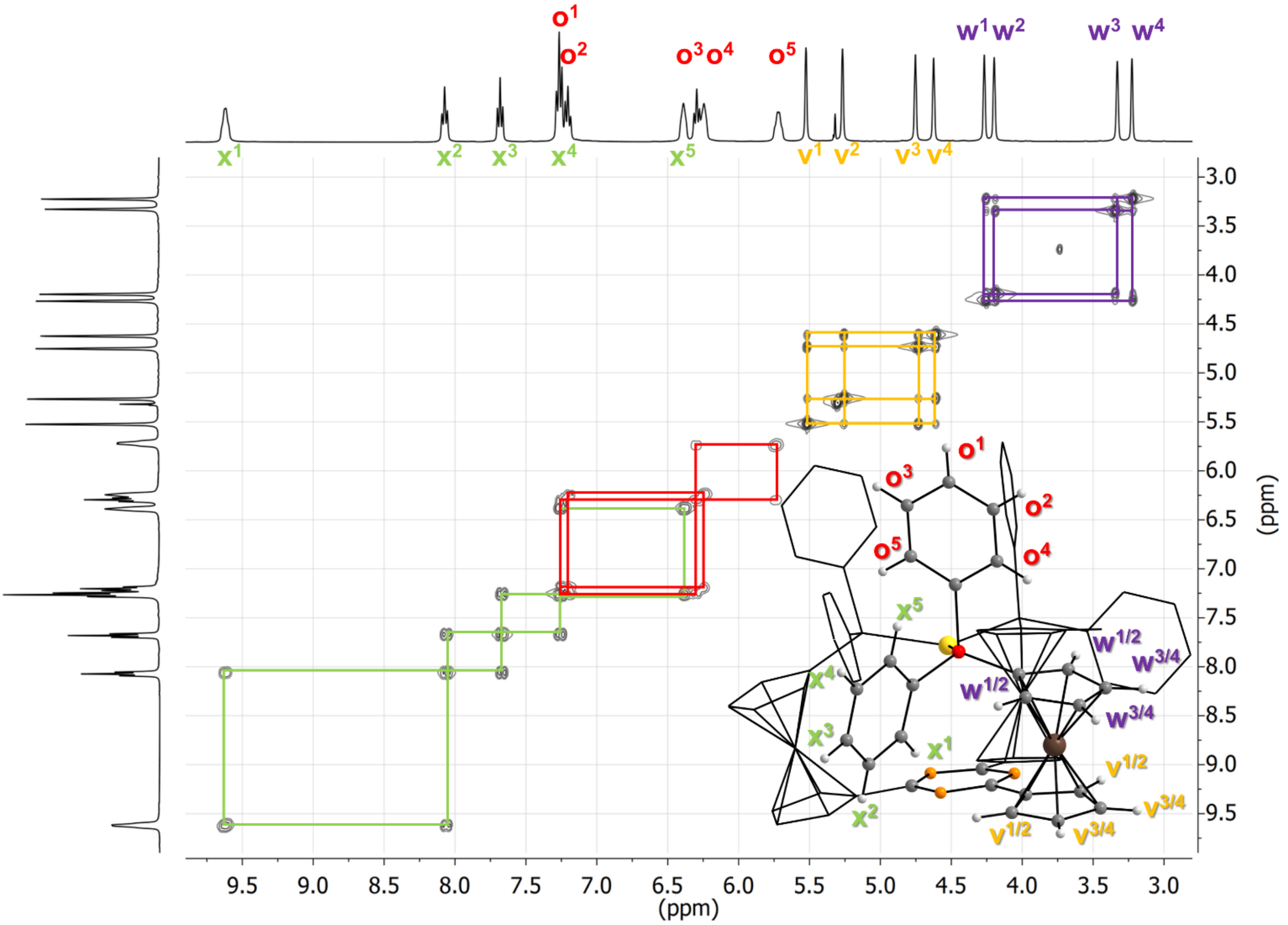

Figure S57. ^{1}H,^{1}H COSY NMR spectrum of **1Au** in CD_2Cl_2 recorded at −50 °C. Externally measured ^{1}H NMR spectra of **1Au** are used instead of the original traces due to their higher resolution. Cross peaks between *J*-coupled resonances are connected using colour-coded lines (green: phenyl ring **x**, red: phenyl ring **o**, orange: cyclopentadienyl ring **v**, purple: cyclopentadienyl ring **w**). The tentative assignments for the phenyl resonances in upper-case number are based on the results of a DFT calculation (s. text and Table S12).

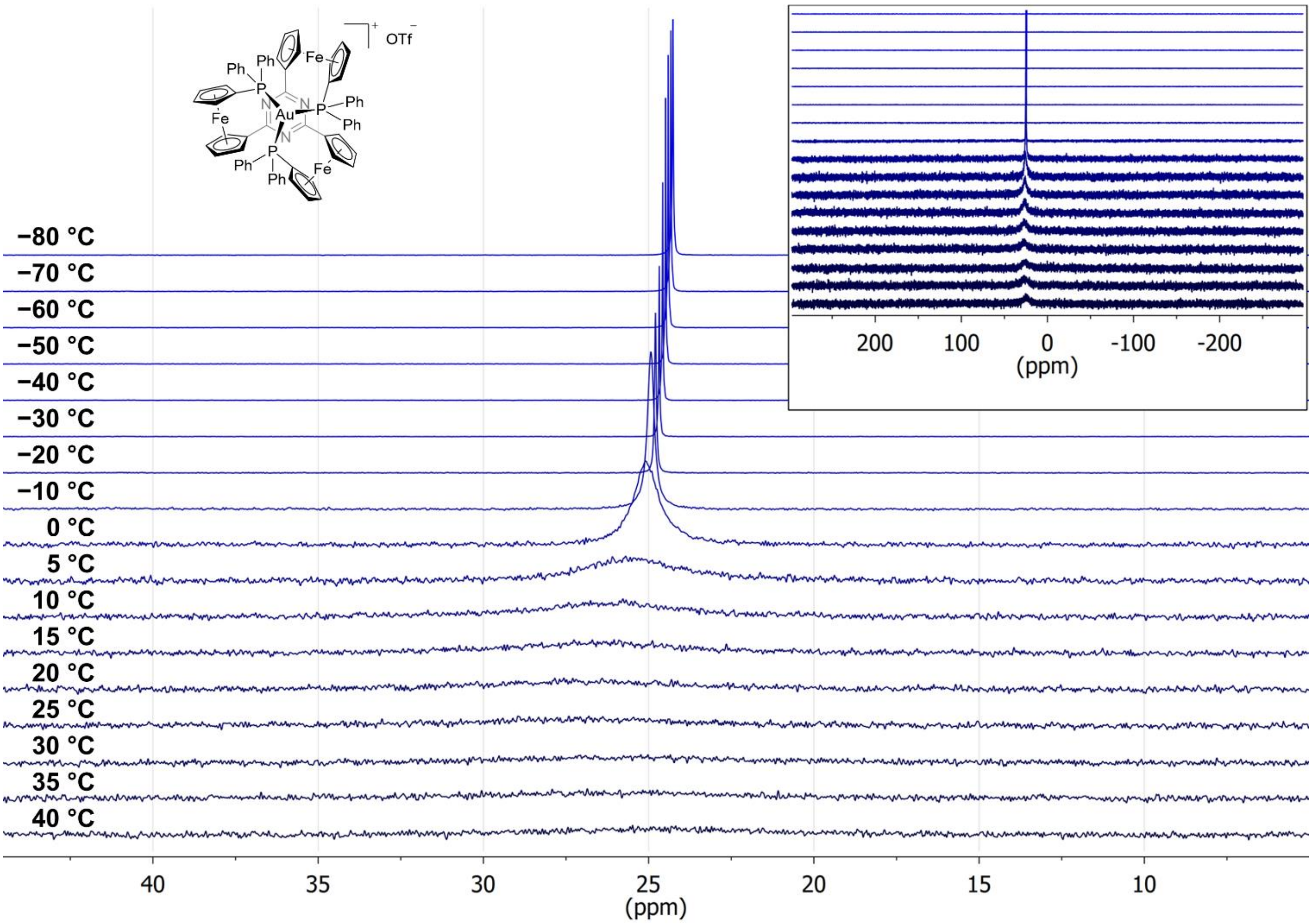

Figure S58. Stacked VT $^{31}P\{^1H\}$ NMR spectra of **1Au** in CD_2Cl_2 over a temperature range from 40 °C to −80 °C. No changes in coordination mode are observed. The insert aims to give a better view of the broad resonance at higher temperatures.

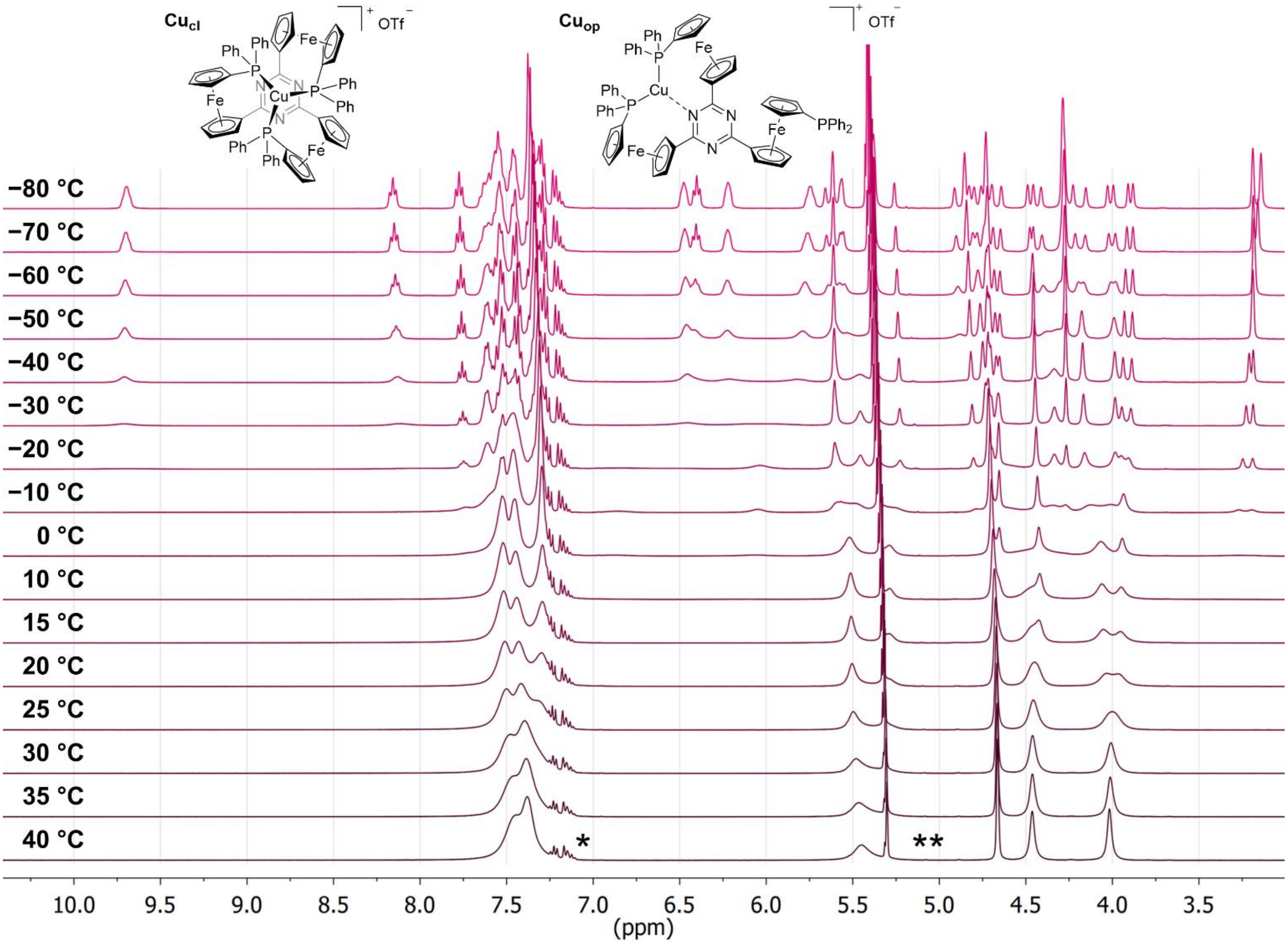

Figure S59. Stacked VT ^{1}H NMR spectra of **1Cu** in CD_2Cl_2 over a temperature range from 40 °C to −80 °C. The asterisks denote toluene (*) and the residual $CHDCl_2$ (**) solvent signals. The decoalescence into overlapping spectra of both **Cu$_{cl}$** and **Cu$_{op}$** takes place gradually between 25 °C and −10 °C.

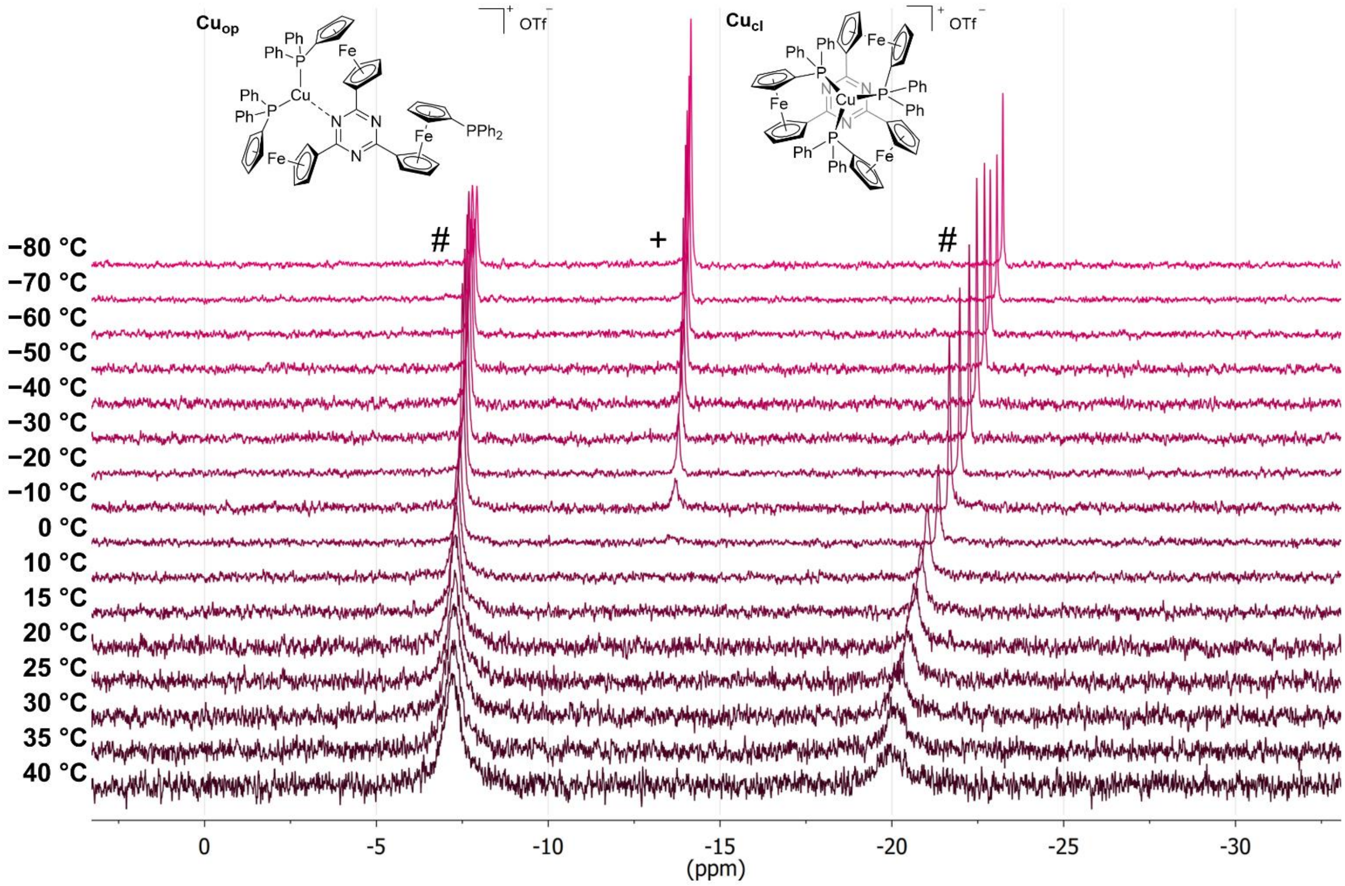

Figure S60. Stacked VT $^{31}P\{^{1}H\}$ NMR spectra of **1Cu** in CD_2Cl_2 over a temperature range from 40 °C to −80 °C. The low-temperature signals for the C_3-symmetric **Cu$_{cl}$** (+) and the bidentate **Cu$_{op}$** (#) are correspondingly labelled.

Although at 40 °C **1Cu** appears C_{3v}-symmetric in CD_2Cl_2, its corresponding $^{31}P\{^{1}H\}$ NMR spectrum at the same temperature still hints at the swiftly equilibrating bidentate form **Cu$_{op}$**. This is most likely due to the greater difference in resonance frequencies for the two nuclei which leads to a higher temperature to attain coalescence in the $^{31}P\{^{1}H\}$ NMR spectrum.

In CD_3CN, **1Cu** appears C_{3v}-symmetric, as judged from both its ^{1}H and $^{31}P\{^{1}H\}$ NMR spectra, already at room temperature, the signals sharpening considerably at higher temperature. Lowering the temperature, decoalescence in both the ^{1}H and the $^{31}P\{^{1}H\}$ NMR spectrum is observable, indicating that CD_3CN acts as an auxiliary ligand promoting the fast equilibration between the three theoretical coordination modes for **1Cu$_{op}$** (for this, see also the titration experiment with CN^{-} (followed by UV/Vis) in section 2.5). In the ^{1}H NMR spectra at −20 °C, **1Cu$_{cl}$** cannot be detected from its well-distinguishable resonances **x^{1}** at 9.7 ppm (cf. Figure S57 and Figure S59), suggesting that CD_3CN acts as ligand rather than the third phosphanyl group.

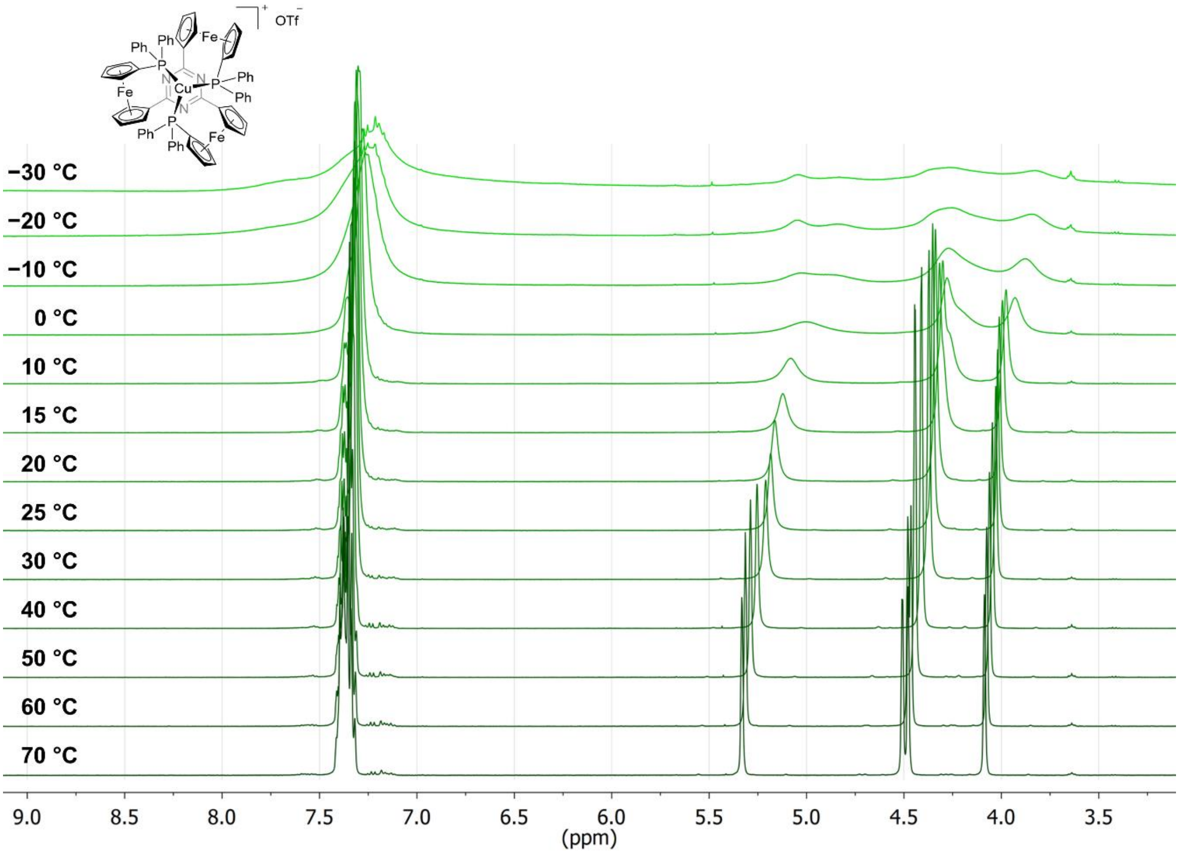

Figure S61. Stacked VT ^{1}H NMR spectra of **1Cu** in CD_3CN over a temperature range from 70 °C to −30 °C.

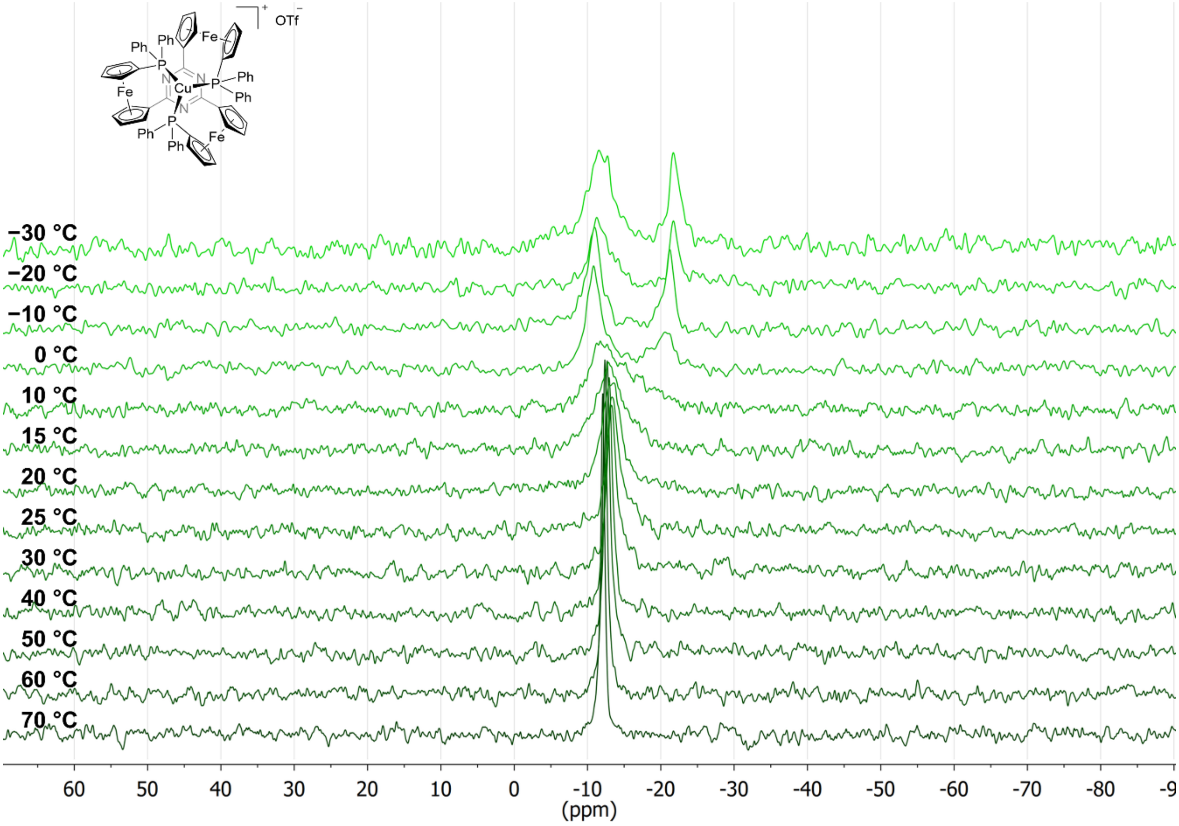

Figure S62. Stacked VT $^{31}P\{^{1}H\}$ NMR spectra (exponential lb = 50 Hz) of **1Cu** in CD_3CN over a temperature range from 70 °C to −30 °C.

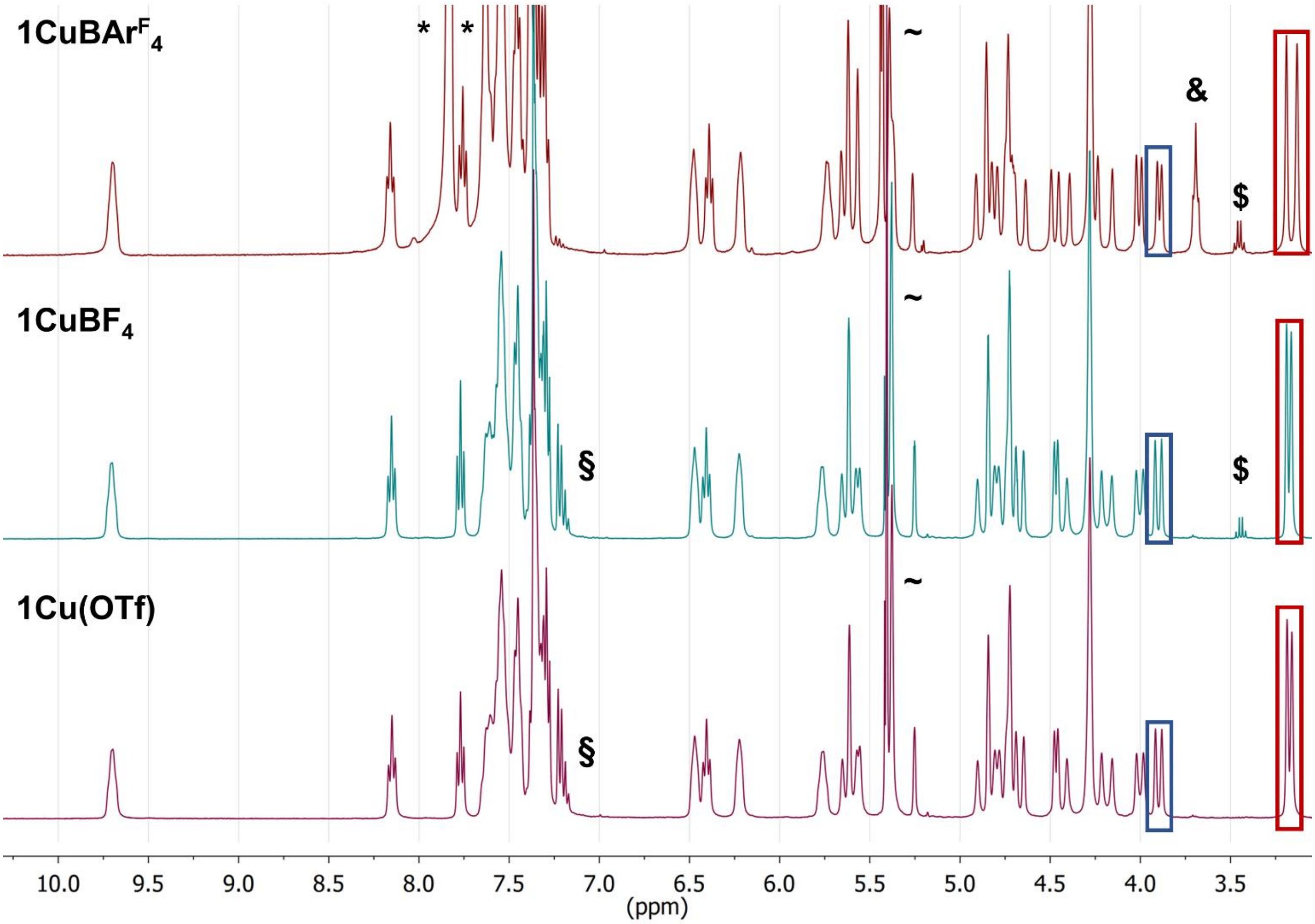

Figure S63. Stacked ^{1}H NMR spectra of **1Cu** (purple, bottom), **1CuBF₄** (teal, middle), and **1CuBAr^{F}₄** (maroon, top) in CD_2Cl_2 recorded at −70 °C. The signals for the *ortho* and *para* protons of the $BAr^{F}{}_4^{-}$ anion are labelled with asterisks (*), residual solvents (toluene: **§**, $CHDCl_2$: **~**, THF: **&**, Et_2O: **$**) are labelled as well. The boxes denote the signals used for the determination of the ratio of **Cu$_{cl}$** : **Cu$_{op}$.**

A comparison of the ^{1}H NMR spectra of three Cu^{I} complexes with triflate (**1Cu**), tetrafluoroborate (**1CuBF$_4$**), and $BAr^{F}{}_4$ (**1CuBAr$^{F}{}_4$**) as their respective anion at −70 °C (Figure S63) does not reveal significant differences in both position and multiplicity for the signals attributed to **1Cu$_{cl}$** and **1Cu$_{op}$**. With respect to their relative intensities, the two signal sets centred at 3.90 ppm (blue box, **1Cu$_{op}$**) and 3.17 ppm (red box, **1Cu$_{cl}$**) have been used. The results are assembled in Table S13 and illustrate that, for **1CuBAr$^{F}{}_4$**, a slight increase in the relative amount of **1Cu$_{cl}$** in this equilibrium can be detected, potentially hinting at a weak involvement of the smaller anions in stabilising the bidentate, open form **1Cu$_{op}$**. In the corresponding ^{19}F{^{1}H} NMR spectra in CD_2Cl_2, no change in the respective signals can be found. In their ^{31}P{^{1}H} NMR spectra at −70 °C (Figure S64), the signal sets appear very similar. Slight differences in signal width and exact position of the free phosphanyl group of **1Cu$_{op}$** in the case of **1CuBAr$^{F}{}_4$** are noticeable. The relative integrals are similar, but their exact comparison is hampered by the pulse sequence (z_gp_g for fast-relaxing nuclei, ^{1}H-decoupled) and the uneven baseline.

Table S13. Relative integral intensities and corresponding ratio of **Cu$_{cl}$** : **Cu$_{op}$** for **1Cu**, **1CuBF$_4$**, and **1CuBAr$^{F}{}_4$** in CD_2Cl_2 at −70 °C.

	Relative integral 3.90 ppm (2H)	Relative integral 3.17 ppm (6H)	Ratio **Cu$_{cl}$** : **Cu$_{op}$**
**1Cu**	1.00	2.25	9 : 12
**1CuBF$_4$**	1.00	2.25	9 : 12
**1 CuBAr$^{F}{}_4$**	1.00	2.67	9 : 10

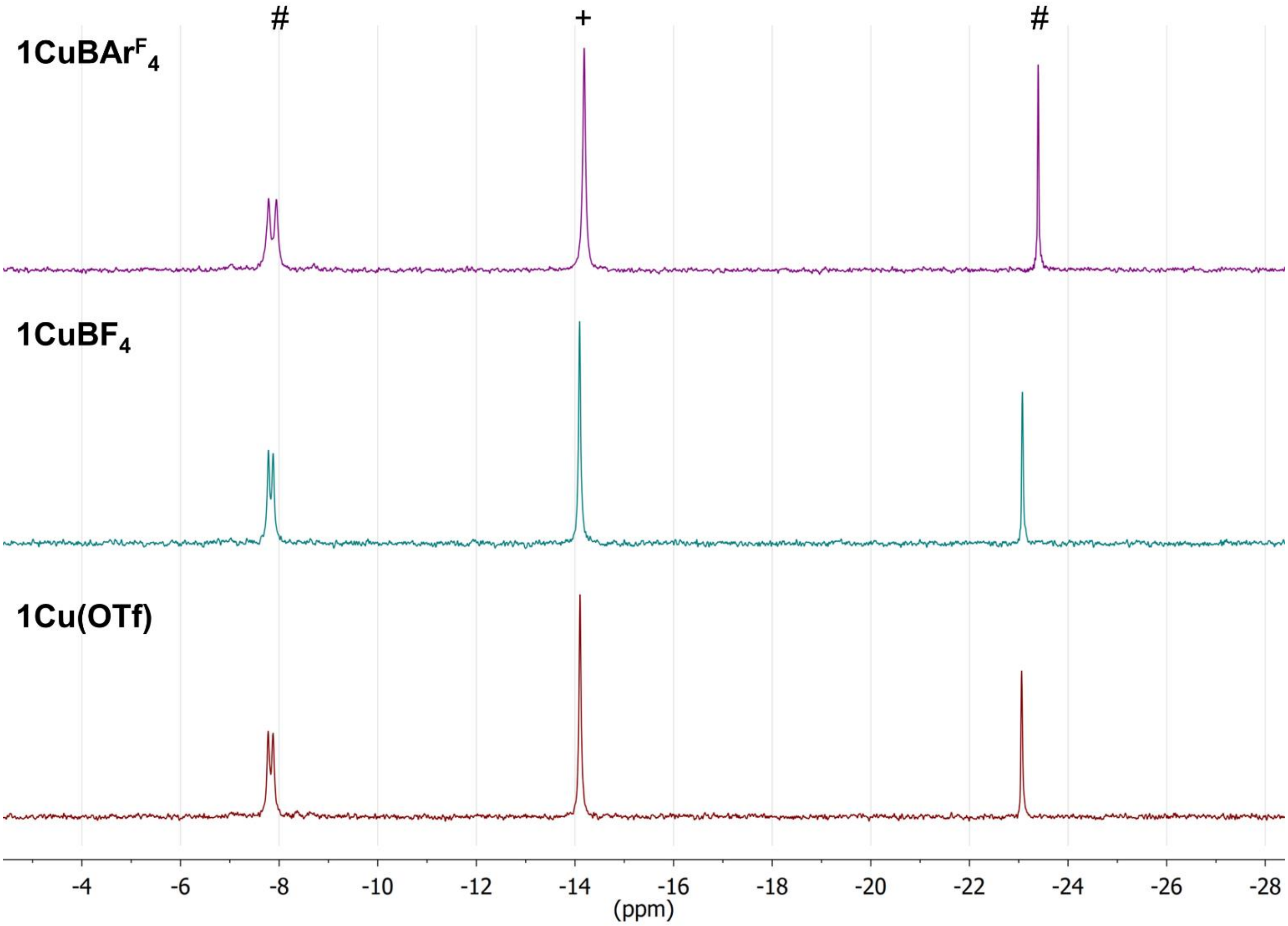

Figure S64. Stacked $^{31}P\{^1H\}$ NMR spectra of **1Cu** (purple, bottom), **$1CuBF_4$** (teal, middle), and **$1CuBAr^F_4$** (maroon, top) in CD_2Cl_2 recorded at −70 °C. The signal sets for the C_3-symmetric **Cu_{cl}** (+) and the bidentate **Cu_{op}** (#) are correspondingly labelled.

The ratio between **$1Cu_{cl}$** and **$1Cu_{op}$** is, however, solvent-dependent. In MeCN-d_3, the room-temperature ^1H and $^{31}P\{^1H\}$ spectra suggest C_{3v} symmetry (Section 1.5, Figure S61, Figure S62); cooling down again decoalesces the ^{31}P resonance (Figure S62). Donor solvents like MeCN and THF might stabilise **$1Cu_{op}$** through weak coordination of the Cu^I ion to different degrees, even though such complexes cannot be detected by HR-ESI MS from solutions of **1Cu** in pure MeCN or THF. In this way, **1Cu**(OTf) and **$1CuBF_4$** exhibit different solvent behaviour in THF-d_8 (Figure S65). While **$1CuBF_4$** behaves similarly in THF-d_8 and in CD_2Cl_2 when lowering the temperature as monitored by ^1H (Figure S66) and $^{31}P\{^1H\}$ (Figure S67) NMR spectroscopy – signals for both **Cu_{cl}** (+) and **Cu_{op}** (#) are detectable, the ratio being strongly shifted towards **Cu_{op}** which seems to be stabilised by THF-d_8 – **1Cu**(OTf) gives markedly different results. Most prominently, other complex species seem to emerge at lower temperature as seen from the $^{31}P\{^1H\}$ NMR spectrum at −60 °C (Figure S68, top left). The ^{19}F resonance, sharp at 25 °C ($\Delta\nu$ = 7.1 Hz), broadens considerably (and much more so than the ^{19}F resonance of **$1CuBF_4$**, increasing from 4.8 to 16 Hz, determined for the more intense peak attributed to $^{11}BF_4^-$) and splits up into at least three signals, one of which is very broad ($\Delta\nu$ = 400 Hz), speaking for an interaction with the quadrupolar $^{63/65}Cu$ (I = 3/2) nuclei of **1Cu**.
Even more so, the stark colour difference between solutions of **1Cu** in DCM, THF and MeCN (Figure S75) is in line with this finding. Notably, **1Cu** and **$1CuBF_4$** yield different UV/Vis spectra in THF (but not in CH_2Cl_2 and MeCN), futher supporting an involvement of the anion in this case, triflate being a better donor for Cu^I than BF_4^-, thus breaking the C_3N_3–Cu^I bond in combination with THF, hypsochromically shifting the absorption maximum to that found for both Cu^I complexes in MeCN.

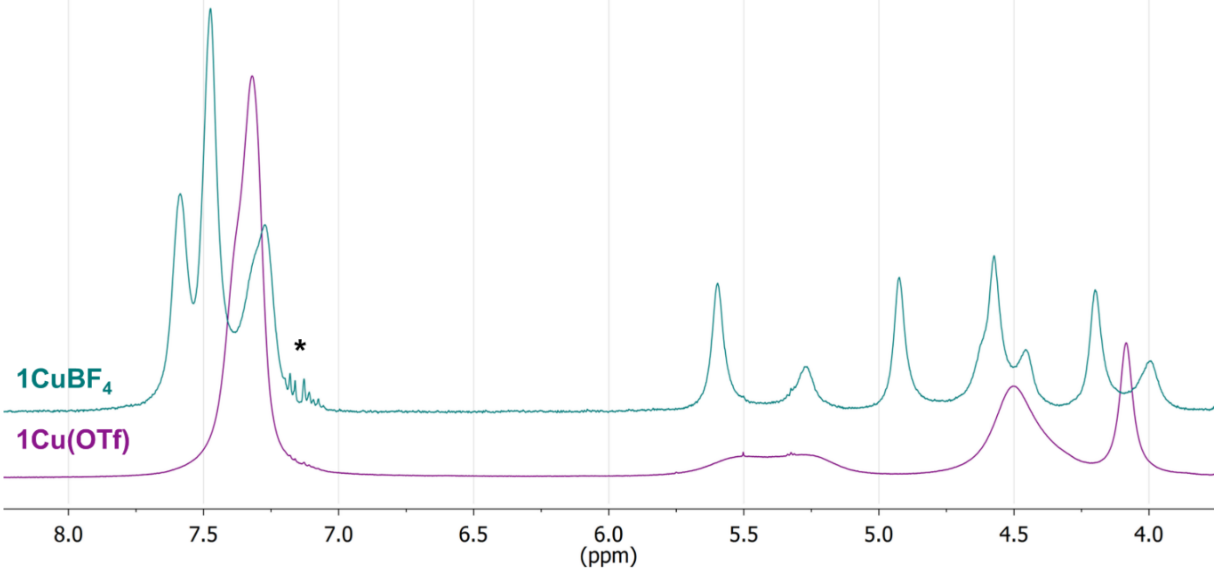

Figure S65. Stacked ^{1}H NMR spectra of **1Cu(OTf)** (purple, bottom) and **$1CuBF_4$** (teal, top) in THF-d_8 recorded at 25 °C. The asterisk (*) denotes residual toluene.

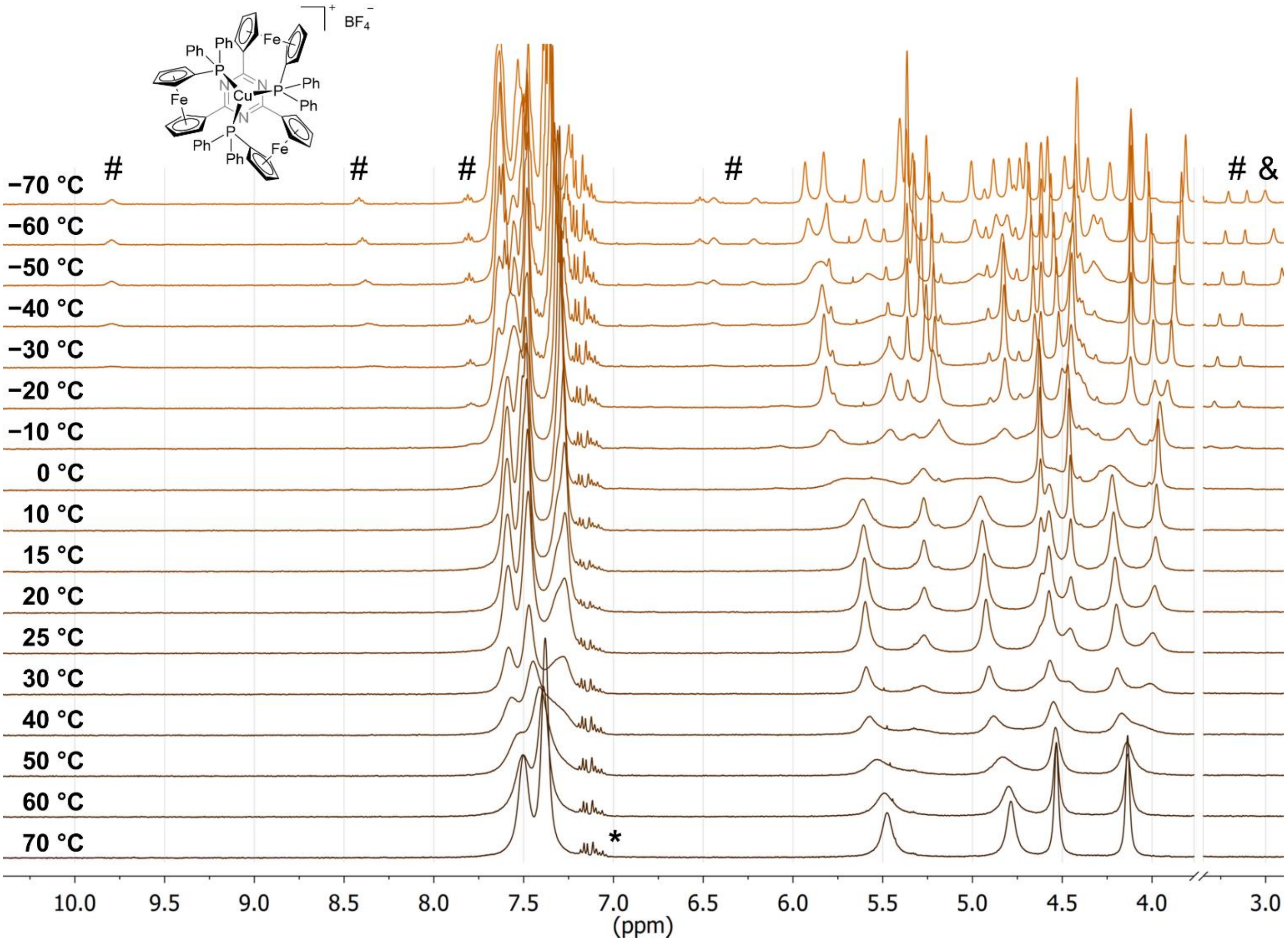

Figure S66. Stacked VT ^{1}H NMR spectra of **$1CuBF_4$** in THF-d_8 over a temperature range from 70 °C to −70 °C. The asterisk (*) denotes residual toluene signals, and the ampersand (&) denotes a solvent impurity that comes into focus at very low temperatures. Well-distinguishable signals arising from C_3-symmetric **Cu_{cl}** are labelled with the hashtag (#) symbol.

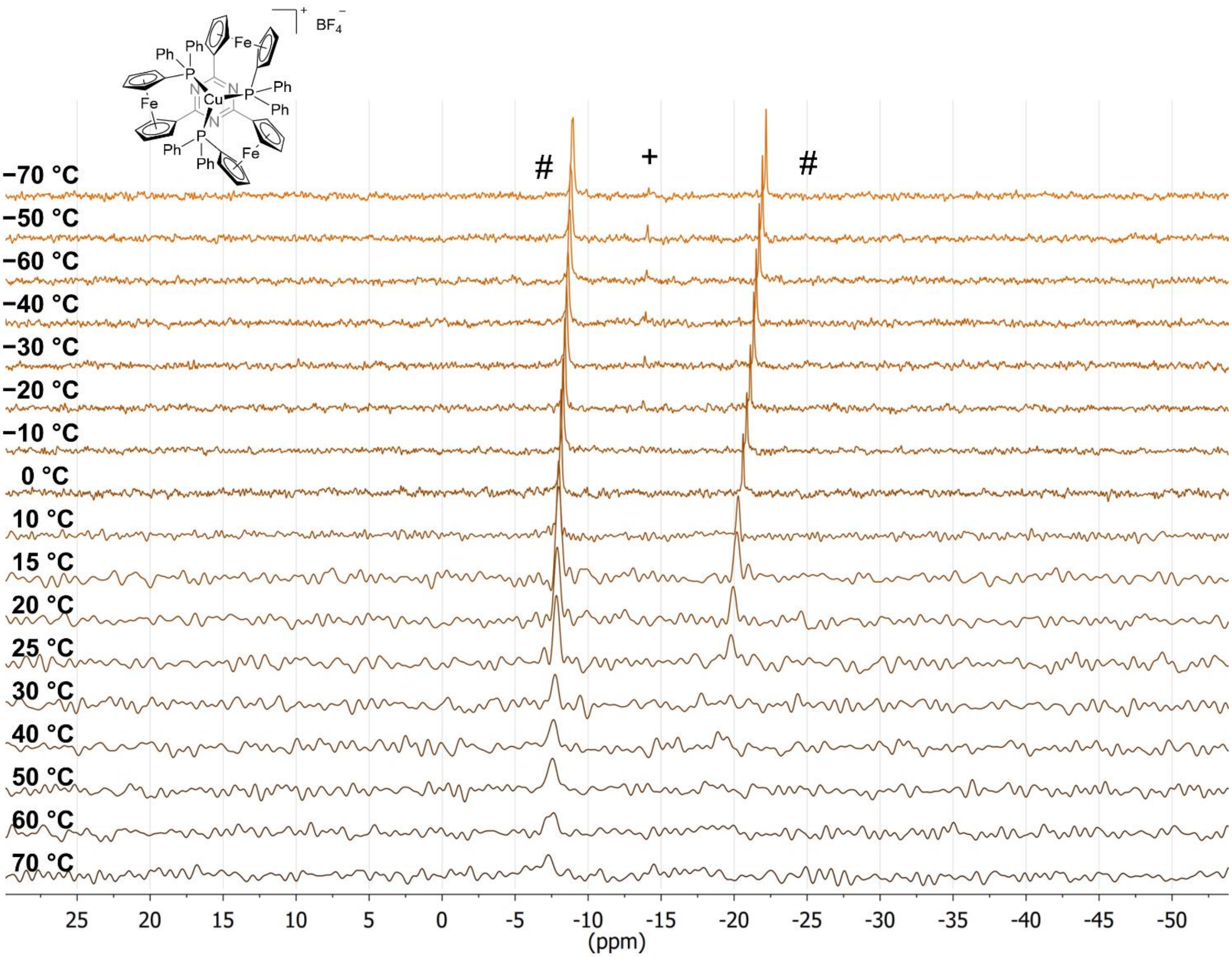

Figure S67. Stacked VT $^{31}P\{^{1}H\}$ NMR spectra of **$1CuBF_4$** in THF-d_8 over a temperature range from 70 °C to −70 °C. The low-temperature signals for the (almost indiscernible) C_3-symmetric **Cu_{cl}** (+) and the bidentate **Cu_{op}** (#) are correspondingly labelled

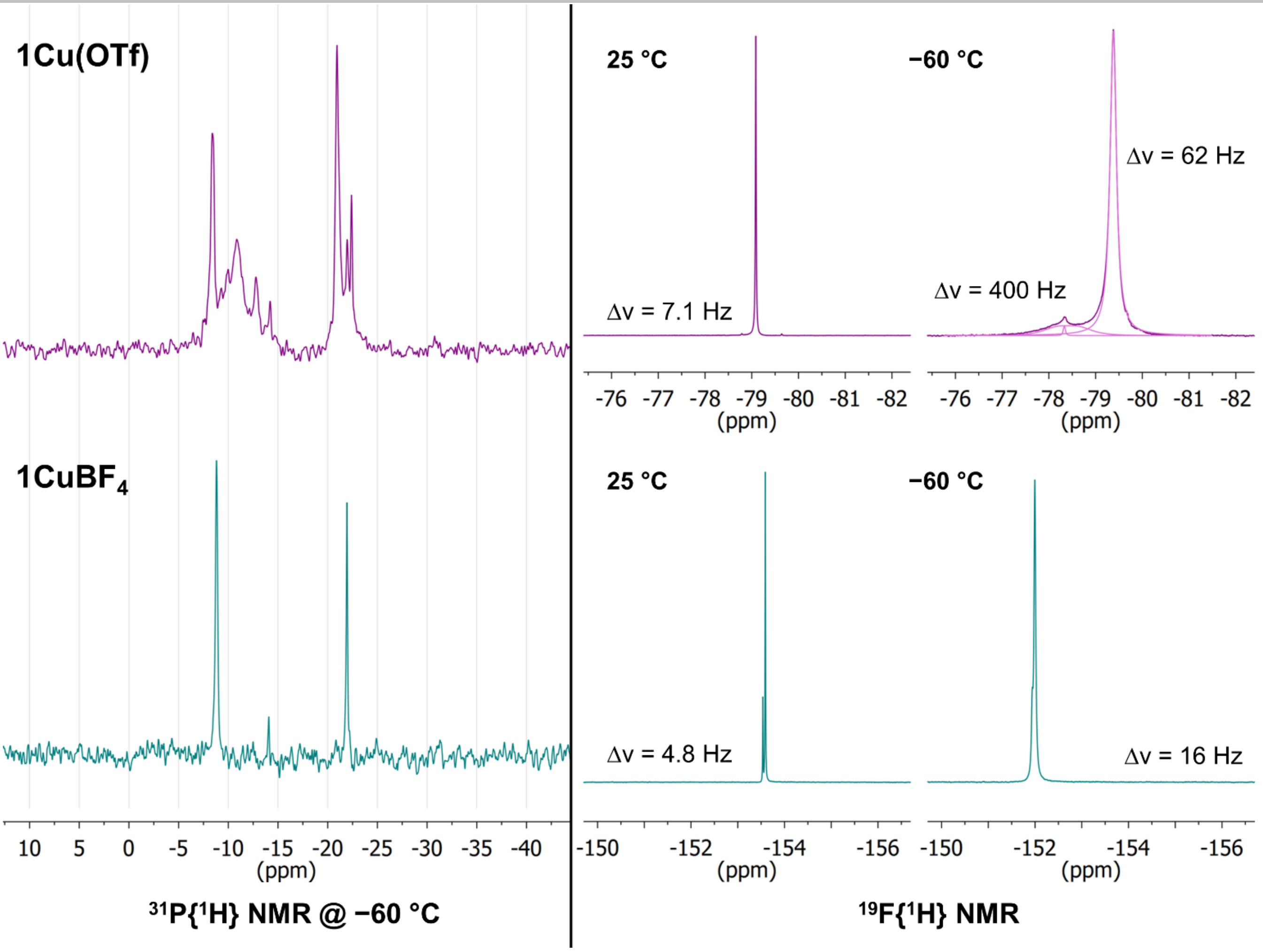

Figure S68. Comparison between **1Cu**(OTf) (purple, top) and **$1CuBF_4$** (teal, bottom) regarding their ^{31}P (left) and ^{19}F (right) NMR resonances in THF-d_8 at −60 °C, supporting the notion of a close contact/binding between Cu^{I} and the triflate anion for **1Cu** in THF-d_8. Line halfwidths were determined using the fitting module of MestreNova; for **1Cu** at −60 °C, the fitted peaks of the $^{19}F\{^1H\}$ NMR spectrum (top right) are shown with pink lines. The second ^{19}F resonance (25 °C) / shoulder (−60 °C) at slightly lower field is due to the isotopic shift between ^{10}B and ^{11}B (cf. Figure S26).

2.4. Computational studies

2.4.1. Investigations on the metal-triazine interactions

The calculated Wiberg bond indices (WBI) between the respective metal and the carbon and nitrogen atoms of the triazine ring in **1Cu**, **1Ag**, and **1Au**, as found in their solid-state geometries, are listed in Table S14. From the comparison of the data it can be concluded that the interaction between the triazine core and the coinage metals follows the order Cu < Au < Ag, thus reflecting the covalent radii of the coinage metals.

Table S14. WBI between the triazine ring atoms and the respective coinage metal ion in **1Cu**, **1Ag**, and **1Au**. Numbering scheme according to Figure S51.

	1Cu	1Ag	1Au
N(1)	0.0223	0.0252	0.0234
N(2)	0.0223	0.0252	0.0235
N(3)	0.0222	0.0253	0.0234
C(1)	0.0059	0.0063	0.0061
C(2)	0.0059	0.0064	0.0061
C(3)	0.0059	0.0063	0.0061

Next, we investigated the interaction of the parent triazine ($C_3H_3N_3$) with the coinage metal ions. Therefore, the geometry of the triazine core and the position of the coinage metal were restrained to the structural parameters obtained for the gas-phase structures of **1Cu**, **1Ag**, and **1Au**, respectively. Then, the interaction energy between metal ion and triazine was calculated as $E_{int} = E_{Triazine–M} − E_M − E_{Triazine}$. The results are summarised in Table S15. The same trend as derived from analysis of the WBI of the complexes **1Cu**, **1Ag**, and **1Au** are found with silver showing the largest interaction energy.

Table S15. Interaction energies E_{int} (in kcal·mol^{-1}) between triazine and the coinage metal ions atoms obtained at the BP86-D3BJ/def2-TZVP level of theory.

	1Cu	1Ag	1Au
E_{int} [kcal·mol^{-1}]	−8.1	−10.6	−9.7

To get insights into the nature of the interaction between the triazine core and the coinage metals, energy decomposition analyses (EDA) based on natural orbitals of chemical valence (EDA-NOCV)[56] were carried out at the BP86-D3BJ/def2-TZVP level of theory. In this approach, the interaction between two fragments is decomposed into pairs of corresponding donor and acceptor orbitals. For our model systems, $C_3H_3N_3$−M^+, three such pairs account for 71% of the total interaction energy for M = Cu (Ag: 73%, Au: 75%). Looking at the shape of these pairs (Table S16), they clearly correspond to cation···π interactions, namely the donation of electron density from the occupied π orbitals of the triazine core to empty s and p orbitals of the coinage metal ions. Other significant contributions to the interaction energy (Cu: 23 %, Ag: 22 %, Au: 20%) are rearrangements of electron density (polarisation) within the triazine moiety, probably induced by the coordination of the positively charged coinage metals (one pair is exemplarily shown for each metal in Table S16). The remaining part of the interaction energy can be assigned to back-bonding from occupied d orbitals of the metals to the triazine core (M−π* back-bonding, one pair is exemplarily shown for each metal in Table S16). Based on the analyses of the NOCV, the triazine molecule can be classified as a donor ligand toward coinage metal cations, whilst back-bonding interactions play an insignificant role.

Table S16. Corresponding pairs of natural orbitals of chemical valence (NOCV) for **$C_3H_3N_3$–Cu^+**, **$C_3H_3N_3$–Ag^+**, and **$C_3H_3N_3$–Au^+**. The donor orbital is rendered in blue while the acceptor orbital is rendered yellow (isosurface values set to 0.06–0.08).

	**$C_3H_3N_3$–Cu^+**	**$C_3H_3N_3$–Ag^+**	**$C_3H_3N_3$–Au^+**
	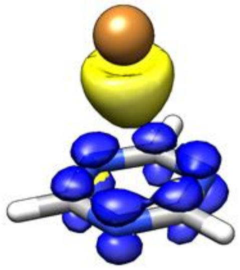	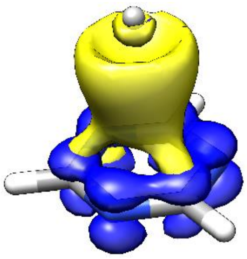	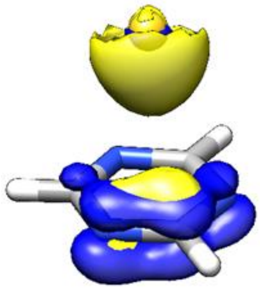
**π−M donation**	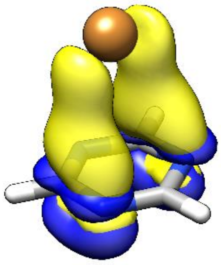	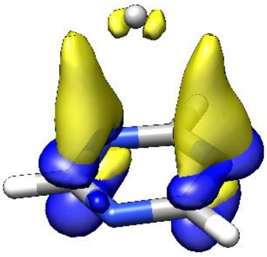	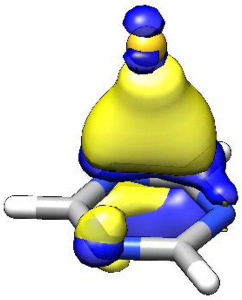
	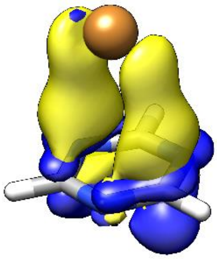	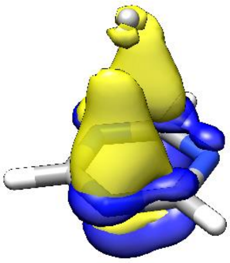	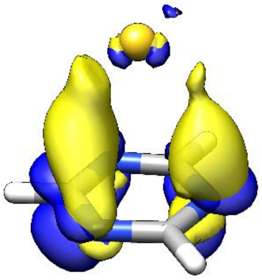
**polarisation of triazine**	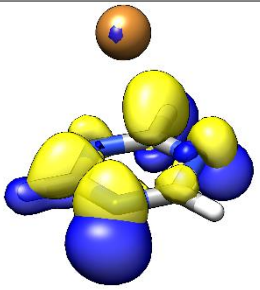	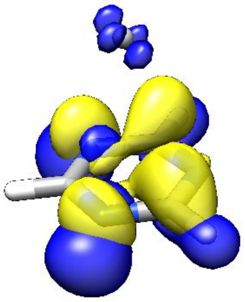	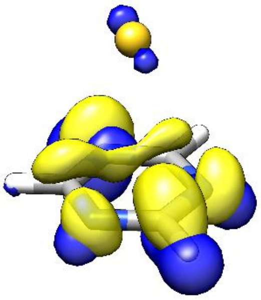

**M−π* back-bonding**	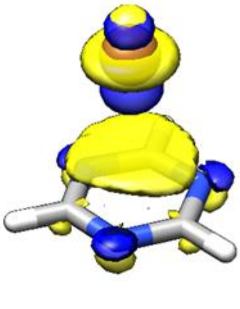	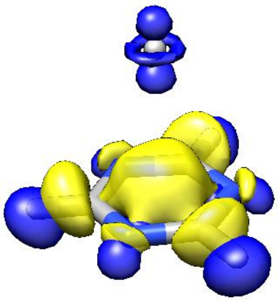	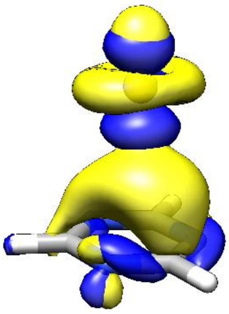

2.4.2. Frontier molecular orbitals of complexes 1M

Table S17. Plots of frontier molecular orbitals (HOMO−1, HOMO, and LUMO) of coinage metal complexes **1Cu**, **1Ag**, and **1Au** (isosurface values set to 0.05), their respective energies rounded to the closest decimal and given below the individual plots.

	HOMO−1	HOMO	LUMO
**1Cu**	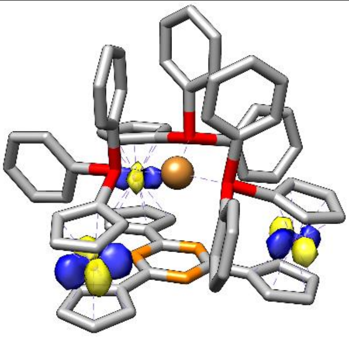 −6.9 eV	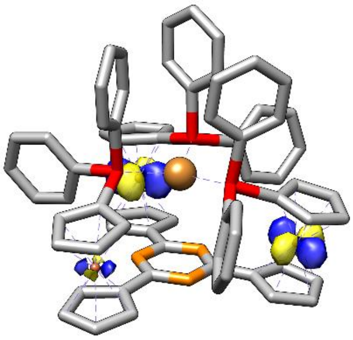 −6.9 eV	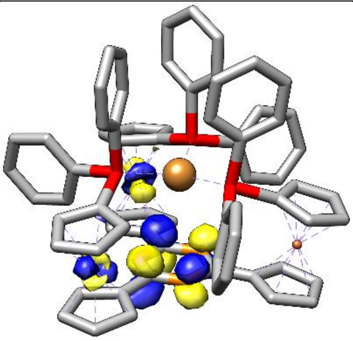 −4.9 eV
**1Ag**	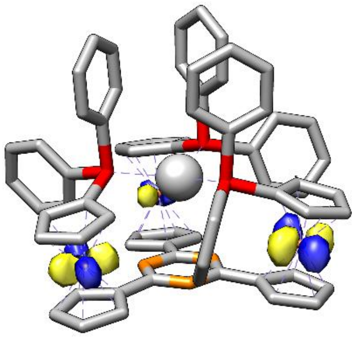 −6.9 eV	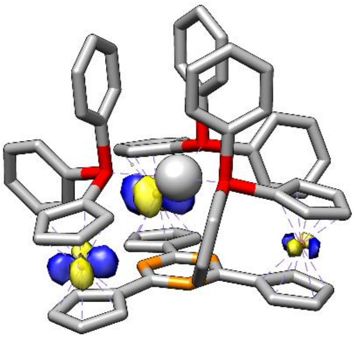 −6.9 eV	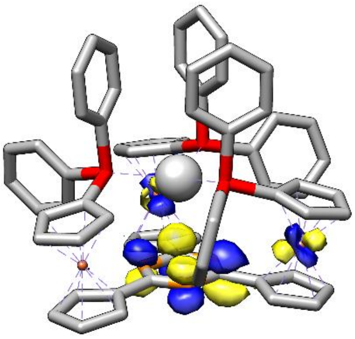 −4.9 eV
**1Au**	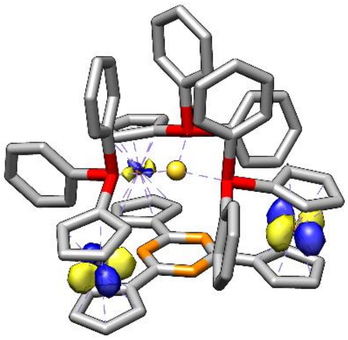 −6.9 eV	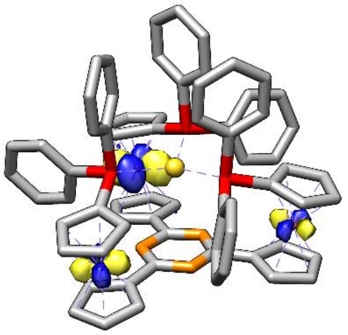 −6.9 eV	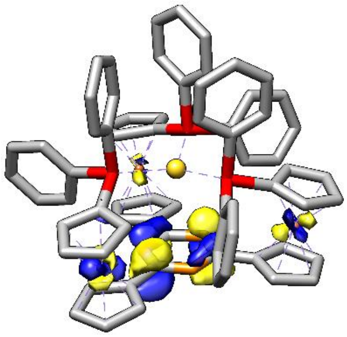 −4.8 eV

The frontier molecular orbitals of the complexes (calculated without considering their respective anions; for **1Cu**, the closed, C_3-symmetric form **Cu$_{cl}$** has been considered solely) are degenerate, owing to the threefold symmetry of these molecules. Table S17 thus only representatively shows HOMO−1, HOMO and LUMO, alongside their respective energies in eV, to illustrate that the HOMO is located at and distributed over the three iron atoms of the ferrocenylene moieties, while the LUMO features contributions of both the triazine core and the ferrocenylene moieties.

2.4.3. Calculation of UV/Vis spectra

TDDFT calculations on **1Cu**, **1Ag**, and **1Au** were conducted at the TPSSh/def2-TZVP [17c, 57] level of theory in the gas phase. For the sake of computational cost, only 100 transitions were calculated for **1Cu**, whilst 240 transitions were calculated for both **1Ag** and **1Au**. The calculated spectra are in good agreement with the experimental spectra (cf. section 2.5.1) measured in dichloromethane (Figure S73 – Figure S75). In the visible part, the spectra are dominated by 3d-π* transitions from the iron centres to the triazine core (see Table S18 for selected difference densities of **1Ag**). At higher wavelengths, π-π* as well as M-π* transitions are observed as well.
As can be seen from Figure S72, the hypothetical structure for **1Cu$_{op}$** with its presumed Cu–C_3N_3 contact (Figure S72) does lead to a broadening of the 3d-π* and 3d-3d transitions for the absorption band at about 500 nm, in line with the experimental UV/Vis spectrum which might be understood as the sum of both forms, **1Cu$_{cl}$** and **1Cu$_{op}$.**

Table S18. Selected difference densities for **1Ag** together with the corresponding calculated wavelength. Transitions proceed from blue to yellow (isosurface values set to 0.001).

**502 nm – 3d-π* and 3d-3d transition**	**394 nm – 3d-π* and 3d-3d transition**
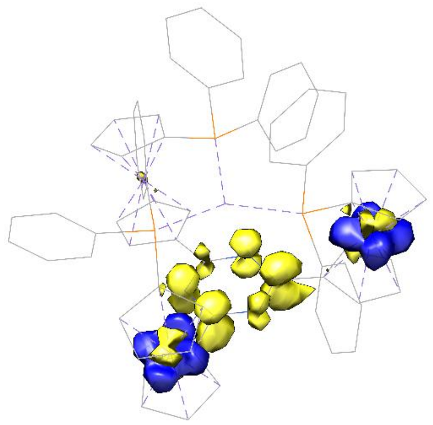	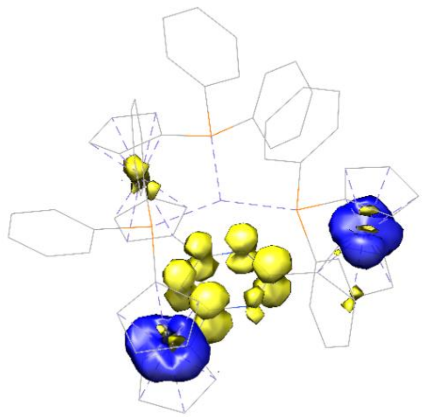
**284 nm – π-π* transition**	**280 nm – M(Fe,Ag)-π* transition**
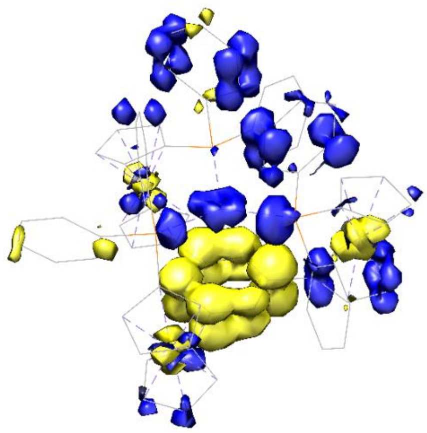	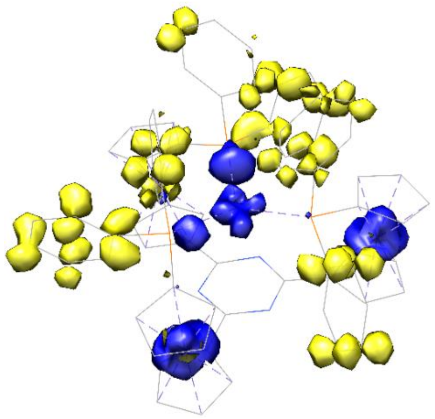

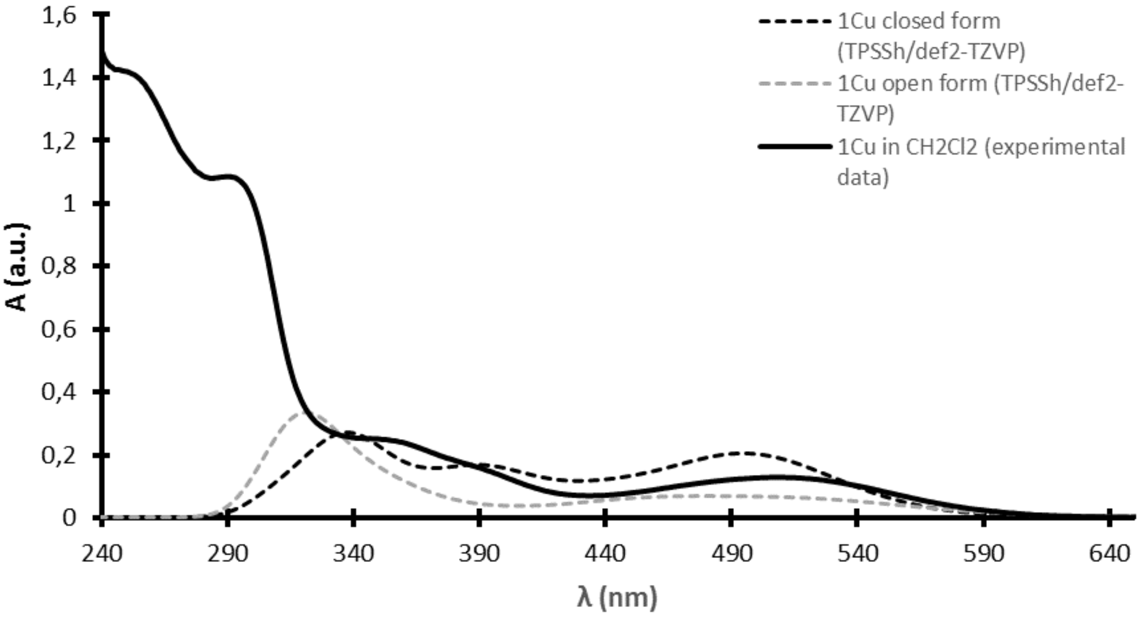

Figure S69. Measured (bold, in CH_2Cl_2) and calculated (**$1Cu_{cl}$** – dashed black; **$1Cu_{op}$** – dashed grey) UV/Vis spectra of **1Cu.**

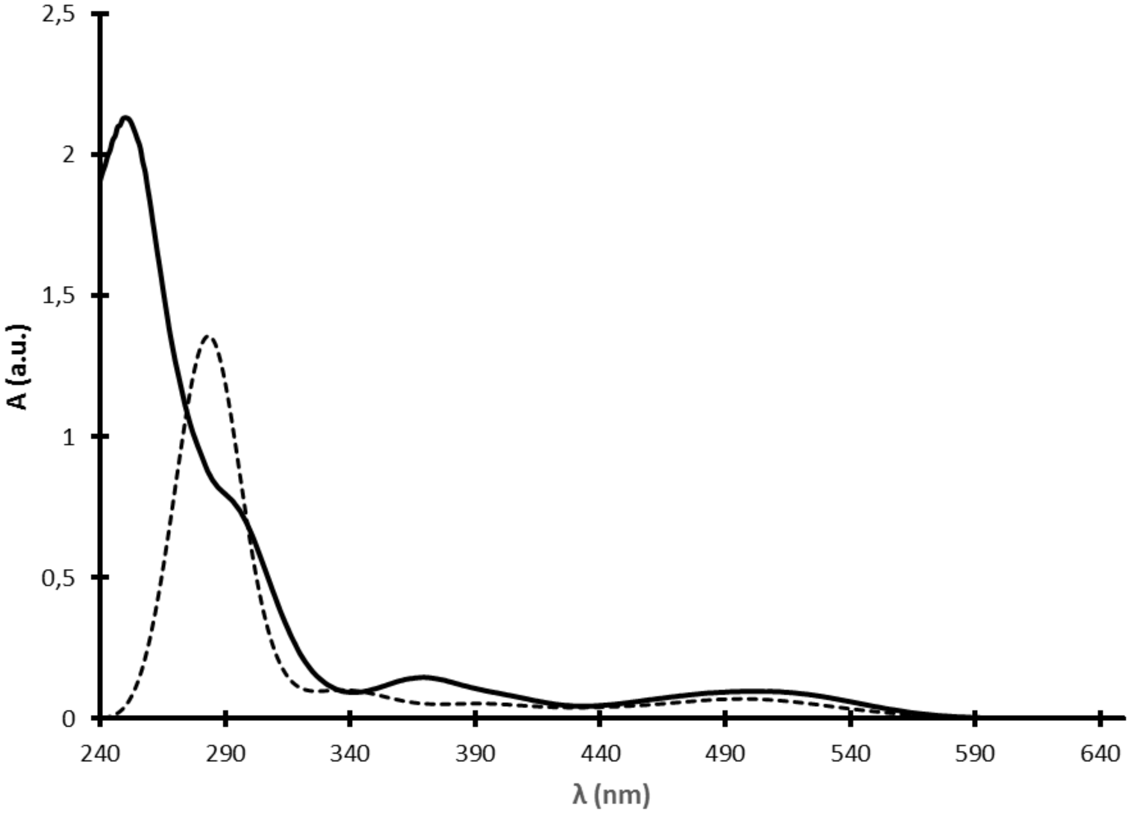

Figure S70. Measured (bold, in CH_2Cl_2) and calculated (dotted line) UV/Vis spectra of **1Ag.**

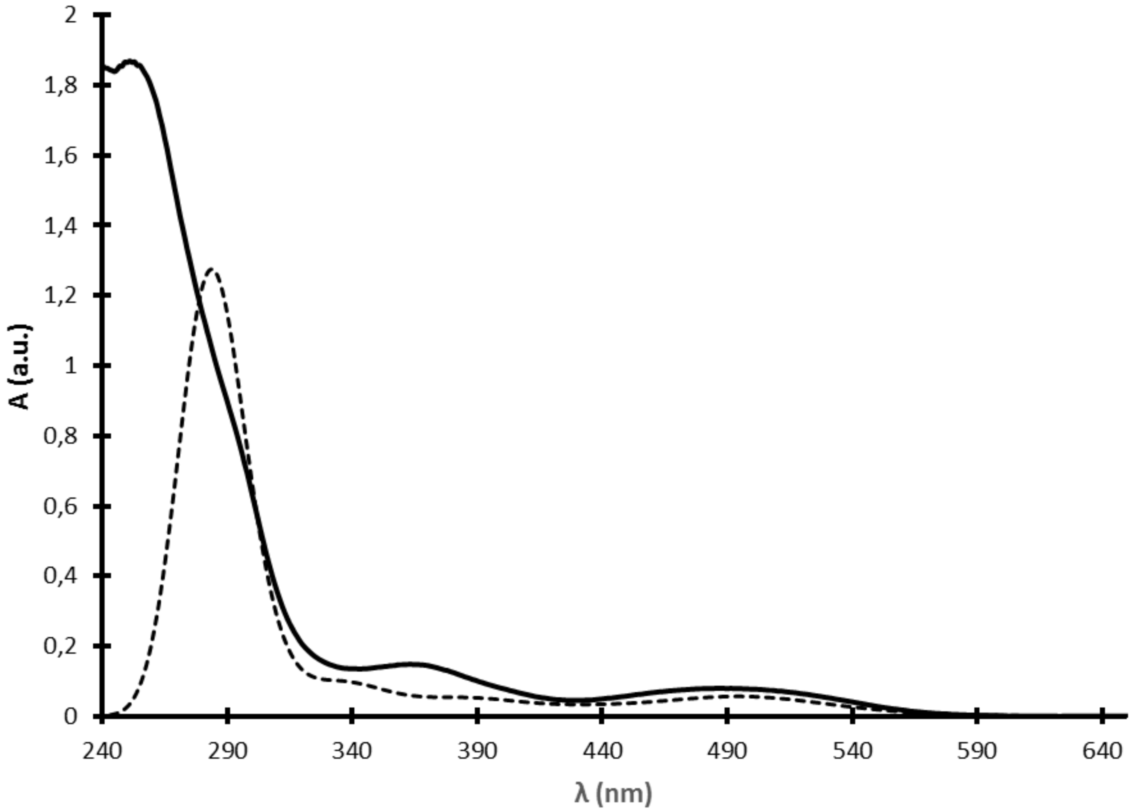

Figure S71. Measured (bold, in CH_2Cl_2) and calculated (dotted line) UV/Vis spectra of **1Au**.

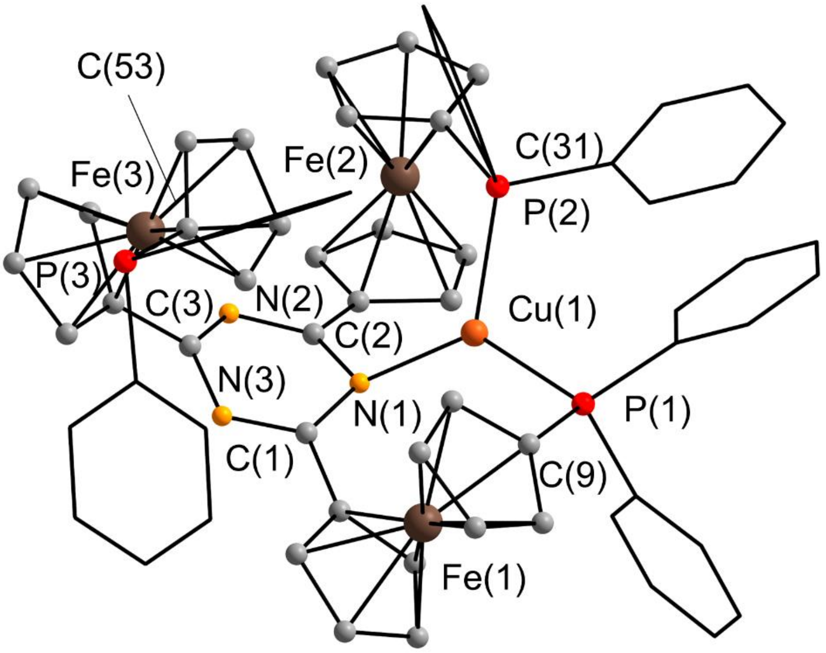

Figure S72. Possible structure for **1Cu$_{op}$** as obtained from a geometry optimisation at the DFT level of theory. For the sake of computational cost, the triflate anion was not included in the geometry optimisation. For clarity, the phenyl rings are drawn in wireframe style and hydrogen atoms have been omitted.

Table S19. Selected bond lengths [Å] and angles [°] for the hypothetical structure of **1Cu$_{op}$** as shown in Figure S72.

	d [Å]		Angle [°]
P(1)–Cu(1)	2.247	P(1)–Cu(1)–P(2)	110.40
P(2)–Cu(1)	2.269	P(1)–Cu(1)–N(1)	127.62
N(1)–Cu(1)	2.026	P(1)–Cu(1)–N(1)	121.24
P(1)–C(9)	1.800	C(1)–N(1)–C(2)	113.08
P(2)–C(31)	1.806	N(1)–C(2)–N(2)	123.54
P(3)–C(53)	1.821	C(2)–N(2)–C(3)	115.29
		N(2)–C(2)–N(3)	122.22
		C(3)–N(3)–C(1)	115.97
		N(3)–C(1)–N(1)	122.97

2.5. UV/Vis spectroscopy

2.5.1. UV/Vis spectra of metal complexes in CH_2Cl_2, THF, and CH_3CN

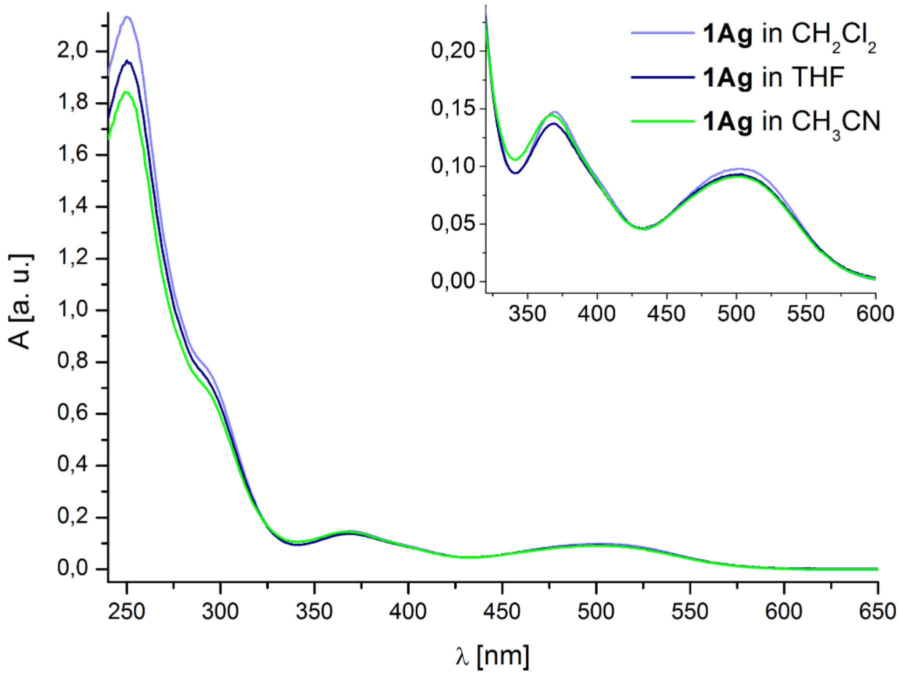

Figure S73. UV/Vis spectra of **1Ag** in CH_2Cl_2 (slate blue), THF (navy), and CH_3CN (green), the insert magnifying the d(Fe)-π*(C_3N_3) transitions at about 500 nm to show the almost negligible solvent influence in detail.

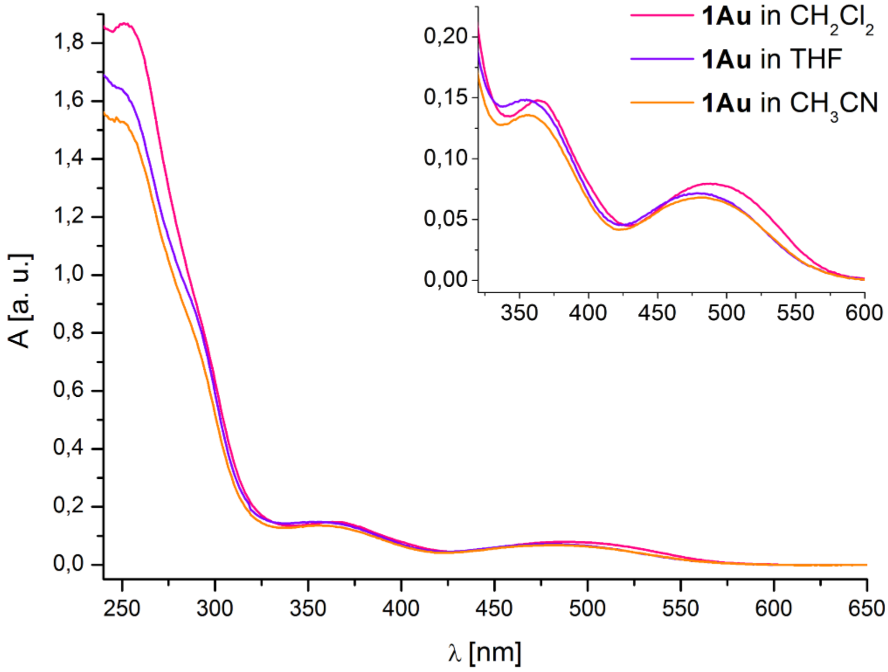

Figure S74. UV/Vis spectra of **1Au** in CH_2Cl_2 (pink), THF (purple), and CH_3CN (orange), the insert magnifying the d(Fe)-π*(C_3N_3) at about 500 nm to show the almost negligible solvent influence in detail.

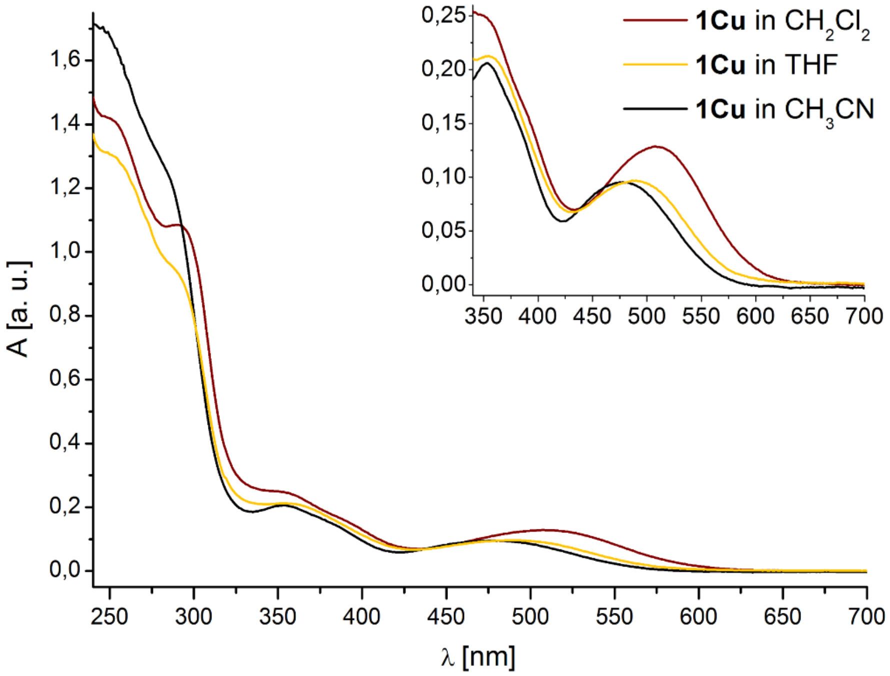

Figure S75. UV/Vis spectra of **1Cu** in CH_2Cl_2 (maroon), THF (yellow), and CH_3CN (black), the insert magnifying the d(Fe)-π*(C_3N_3) transitions at about 500 nm to show the prominent solvent influence in detail.

While for **1Ag** (Figure S73) and **1Au** (Figure S74) the solvent does not result in significant shifts of both the position and the extinction coefficient of the electronic transitions in the UV/Vis region (the strongest influence can be seen for the ligand-centred π–π* and the M(Fe,Au)–π* transitions which decrease in the order CH_2Cl_2 > THF > CH_3CN, correlating with the donor strength of the solvent), **1Cu** (Figure S75) shows a more pronounced solvent effect, especially regarding the d(Fe)-π*(C_3N_3)/d(Fe)-d(Fe) transitions which, in CH_2Cl_2 (maroon curve), are batho- and hyperchromically shifted as well as broadened with respect to the peaks in THF (yellow) and CH_3CN (black). This effect also becomes apparent when comparing the three complexes with each other; the visibly more intense and darker colour of **1Cu** in CH_2Cl_2 when compared to its heavier homologues is reflected in the UV/Vis spectra (Figure S76). As also noted in the VT NMR experiments, the anion plays a role for **1Cu** in THF (Figure S77). In contrast to the BF_4^- anion, the triflate anion in **1Cu** seems to bind to Cu^I, disturbing the C_3N_3–Cu^I contact responsible for the bathochromic shift.

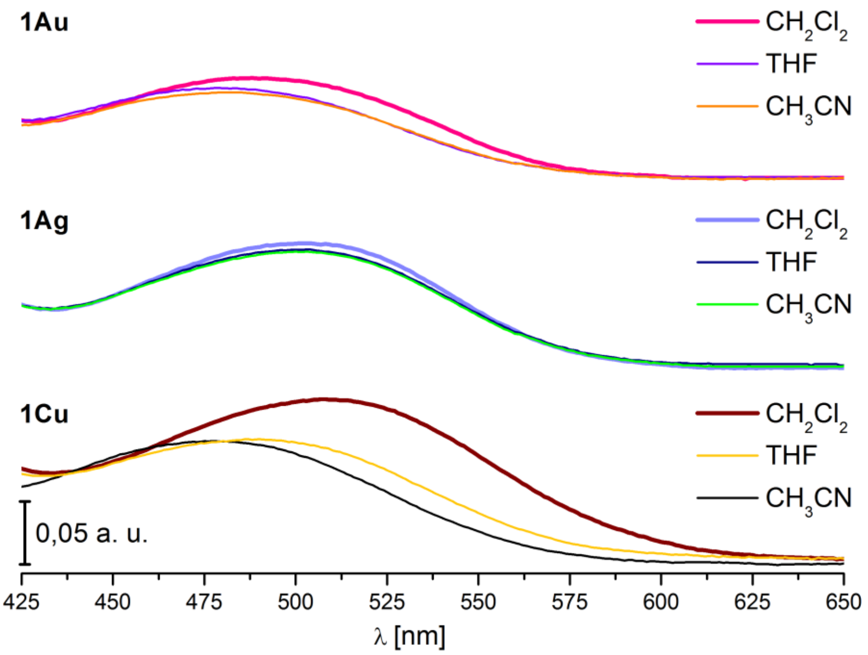

Figure S76. Comparison of the d(Fe)-π*(C_3N_3) transitions in the UV/Vis spectra of **1Au** (top), **1Ag** (middle), and **1Cu** (bottom) in the three solvents under investigation (CH_2Cl_2 marked in bold), highlighting the stark solvent influence that **1Cu** experiences.

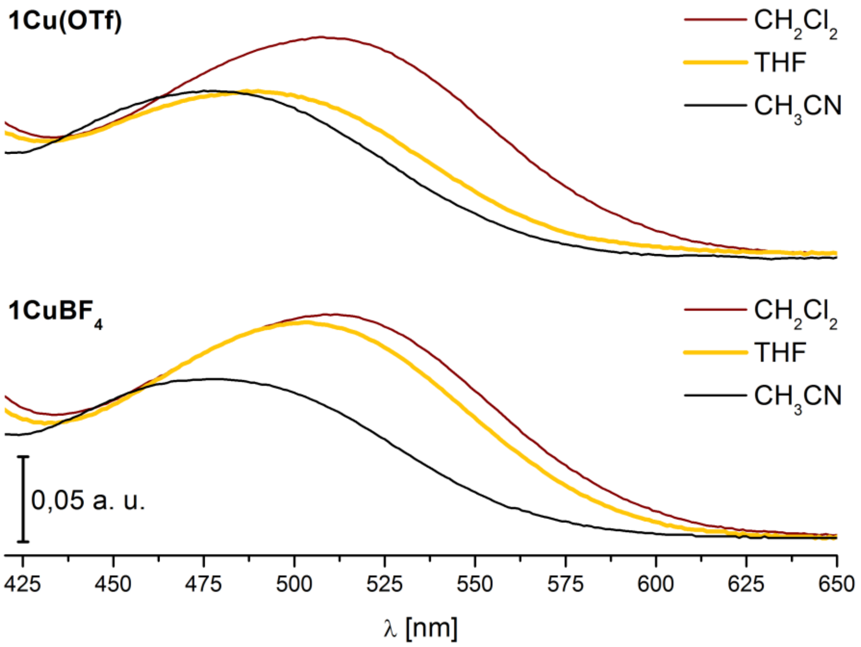

Figure S77. Comparison of the d-d transitions in the UV/Vis spectra of **1Cu(OTf)** (top) and **$1CuBF_4$** (bottom) in the three solvents under investigation (THF marked in bold), highlighting the anion effect resulting in a hypsochromic shift for **1Cu** with respect to **$1CuBF_4$** in THF.

2.5.2. UV/Vis titration of 1Cu with ($n$$Bu_4N$)CN

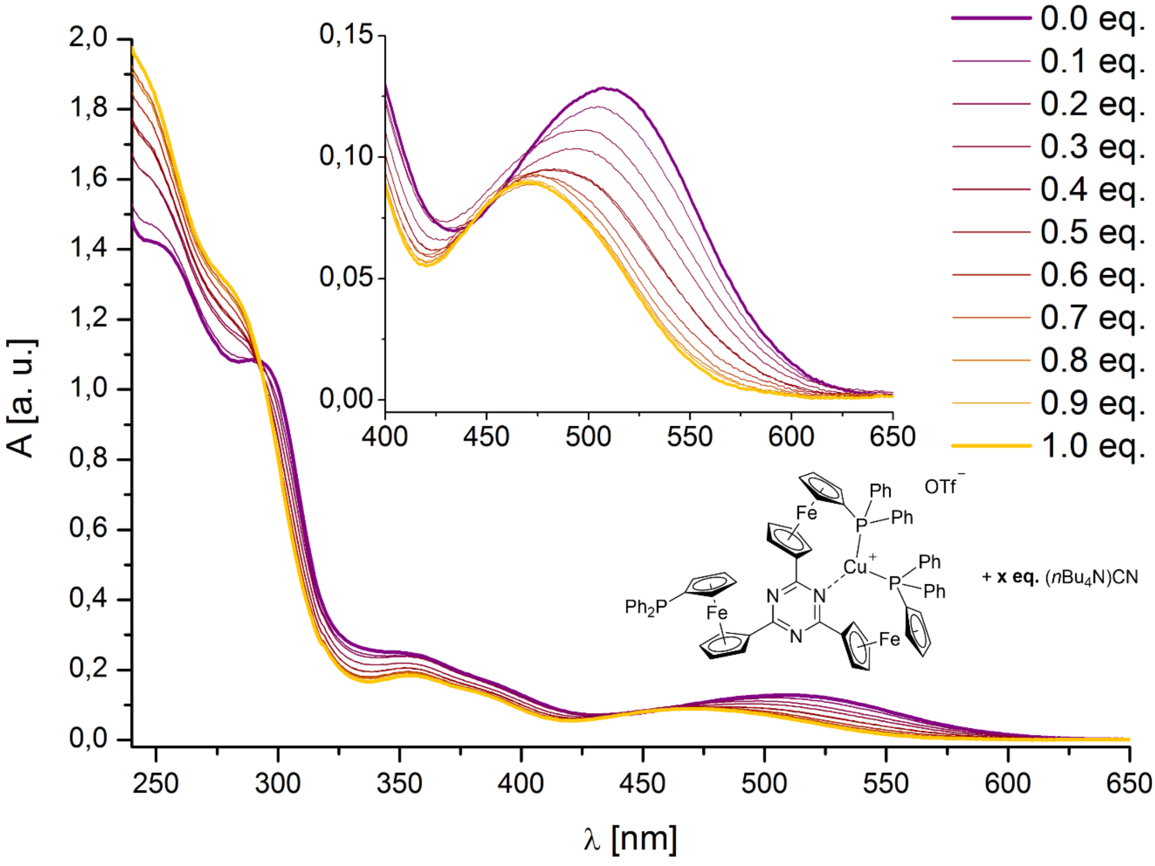

Figure S78. UV/Vis-monitored titration of **1Cu** with ($n$$Bu_4N$)CN in CH_2Cl_2 in steps of 0.1 eq. (start and endpoint of titration marked in bold), the insert magnifying the ferrocene-based d-d transition and its hypsochromic shift upon continued addition of CN^- anions.

The C_3N_3–Cu^I contact in **1Cu**, persistent in CH_2Cl_2, can be disrupted by adding a strong donor such as CN^-. Upon addition of up to 1.0 eq. CN^- (Figure S78), the d(Fe)-π*(C_3N_3)/d(Fe)-d(Fe) transition of **1Cu** is shifted hypsochromically, suggesting the breaking of the C_3N_3–Cu^I contact which, by removing electron density from the triazine core and thus also affecting the electronic transitions of the ferrocenylene moieties, is responsible for the original bathochromic shift of **1Cu** compared to **1Ag** and **1Au**. Addition of a second equivalent of CN^- (Figure S79) leads to little change concerning the peak position but induces a slight hyperchromic shift of the d-d transition associated with the ferrocenylene groups. This might, comparing the resulting spectrum to that of the free ligand **1** (black, bold), potentially hint at a ligand exchange, yielding free **1** and $[Cu(CN)_2]^-$ or $[Cu(CN)_4]^{3-}$. An HR-ESI mass spectrometric analysis of **1Cu** and two equivalents of ($n$$Bu_4N$)CN did not yield signals for either **1**, **[1Cu−OTf]$^+$** or related anionic species (*e.g.*, **[1Cu−OTf+2CN]$^-$**. Trace amounts of $[Cu(CN)_2]^-$ have been detected.

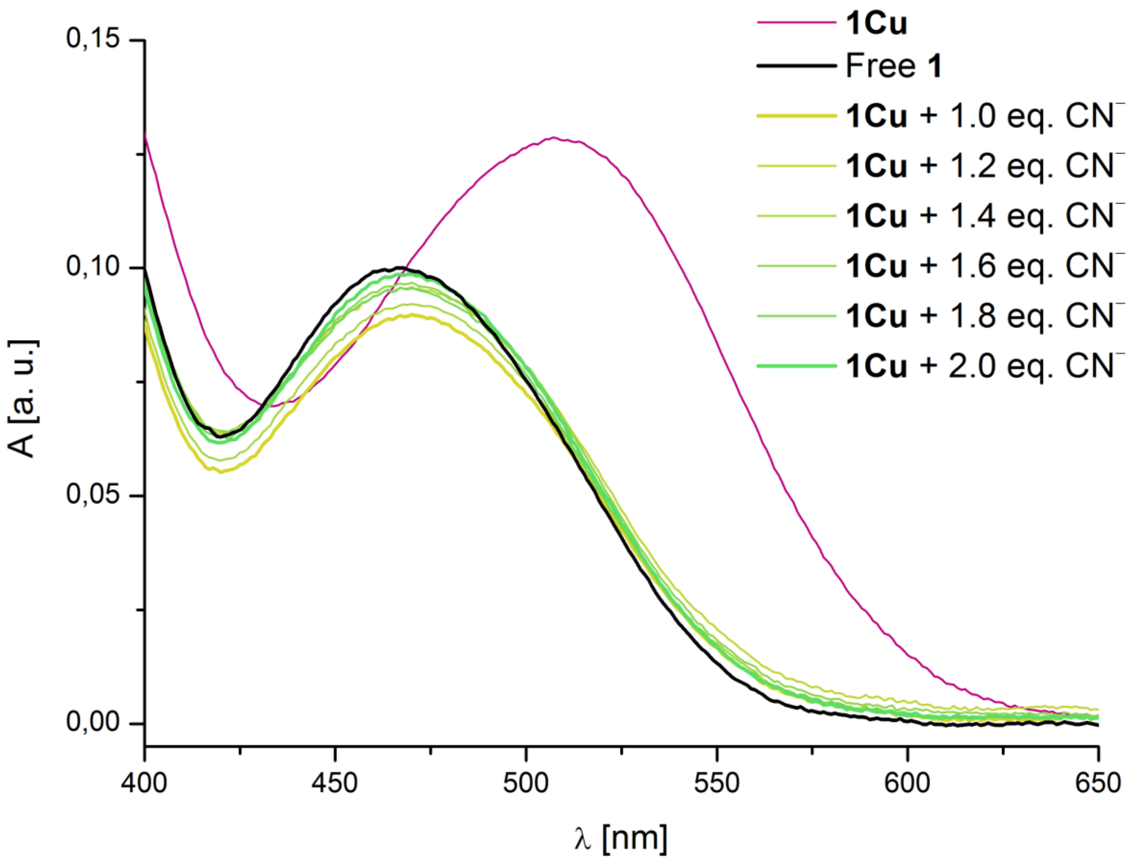

Figure S79. Detail of the d(Fe)-π*(C_3N_3) transitions in the titration of **1Cu** with ($n$$Bu_4N$)CN in CH_2Cl_2 (1:1, then in steps of 0.2 eq.; monitored by UV/Vis spectroscopy, start and end point of titration marked in bold) in comparison to the d(Fe)-π*(C_3N_3) transitions of free ligand **1** (black) and complex **1Cu**.

2.6. Electrochemistry

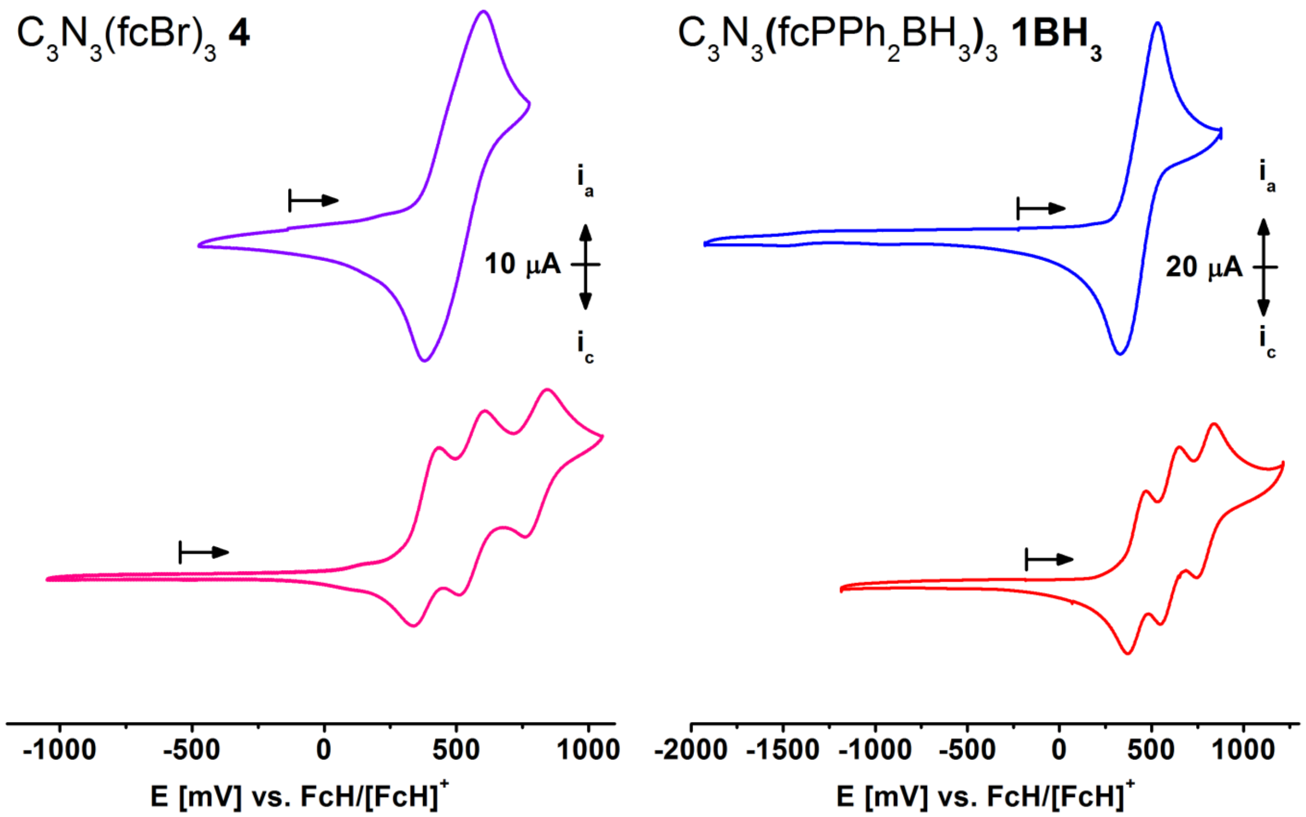

Figure S80. Cyclic voltammograms of 2,4,6-tris(1-bromo-1'-ferrocenylene)-1,3,5-triazine (**4**) (left) and borane-protected ligand $\mathbf{1BH_3}$ (right) in tetrafluoroborate- (top) and $BAr^F{}_4{}^-$-based (bottom) supporting electrolytes (SE) in CH_2Cl_2. In all cases, the second of three consecutively measured cycles is shown. Arrows represent the starting potential and initial scan direction (100 mV·s^{-1}).

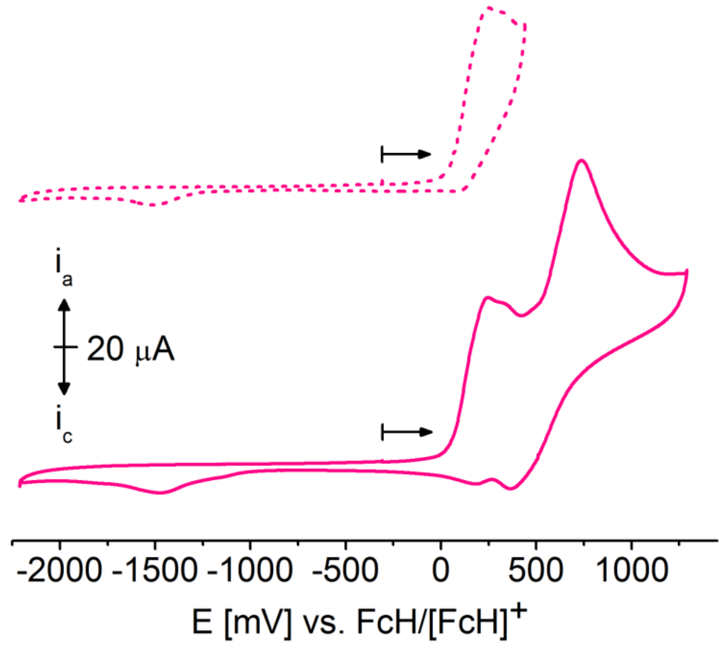

Figure S81. Cyclic voltammograms of ligand **1** in (nBu$_4$N)BF$_4$/CH$_2$Cl$_2$ with different turning potentials. In both cases, the second of three consecutively measured cycles is shown. Arrows represent the starting potential and initial scan direction (100 mV·s^{-1}).

While both the ligand precursor **4** and the borane-protected ligand **1BH3** are reversibly oxidisable in both the BF_4^-- and the $BAr^F{}_4^-$-based SE (Figure S80) and even display three individual redox events when measured in the very weakly coordinating $BAr^F{}_4^-$-based SE, free ligand **1** yields non-reversible oxidation events and, tied to the first of several oxidation events, a delayed reduction hinting at an electron transfer-chemical reaction (EC) oxidation mechanism (Figure S81).

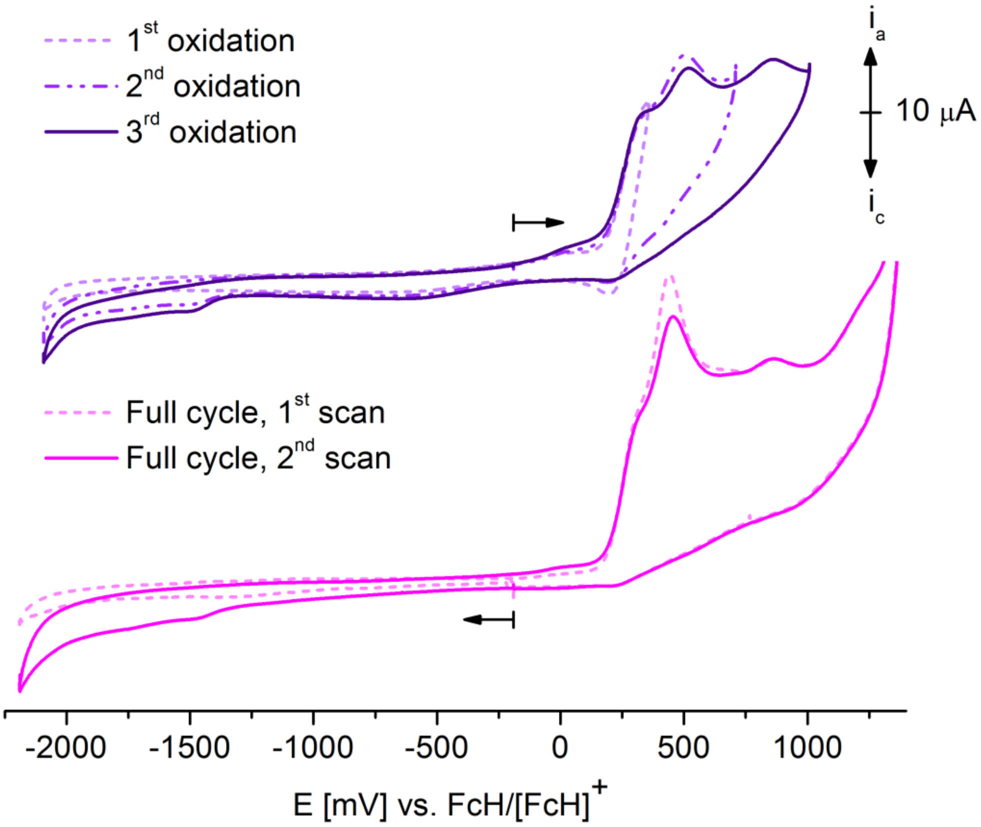

Figure S82. Cyclic voltammograms of ligand **1** in (nBu$_4$N)$BAr^F{}_4$/CH_2Cl_2 with different turning potentials (top, 2nd cycles shown), indicating the presence of three oxidation events, and the dependency of the broad reductive events at about –1.5 V on prior oxidation (bottom). Arrows represent the starting potential and initial scan direction (100 mV·s^{-1}). The most anodic scan (pink, bottom) is cropped due to reasons of clarity, as the sharp increase in current only relates to the electrolyte.

The electrochemical behaviour of ligand **1** in the $BAr^F{}_4^-$-based SE is shown in Figure S82. While the first oxidation at about 340 mV (top, dashed lilac) is reversible to some degree (*vide infra*), the later oxidations at 500 mV (top, dash-dotted lavender) and 860 mV (top, solid blue) are irreversible and cancel out the partial reversibility of the first oxidation. Similar to the properties of **1** in the BF_4^--based SE, the second and third oxidation induce broad yet weak reductive events at cathodic potentials around −1.5 V which are absent if the scan direction is reversed immediately after the first oxidation. The bottom part of Figure S82 confirms this behaviour. If the cycle starts with a cathodic scan, no reductive events can be identified, while the broad reductive features appear after the first oxidations have been carried out in the second cycle.
Recording the first oxidation with three different scanning speeds (20, 100, and 500 mV·s^{-1}) and normalising the currents according to the Randles-Sevčik equation for the square root of the scanning speed (Figure S83),[58] the increase of reversibility indicates an EC(EE) mechanism operating in that case. The first oxidation, likely iron-centred, thus generates a species undergoing a chemical transformation yielding an electroactive product which participates in further redox events.

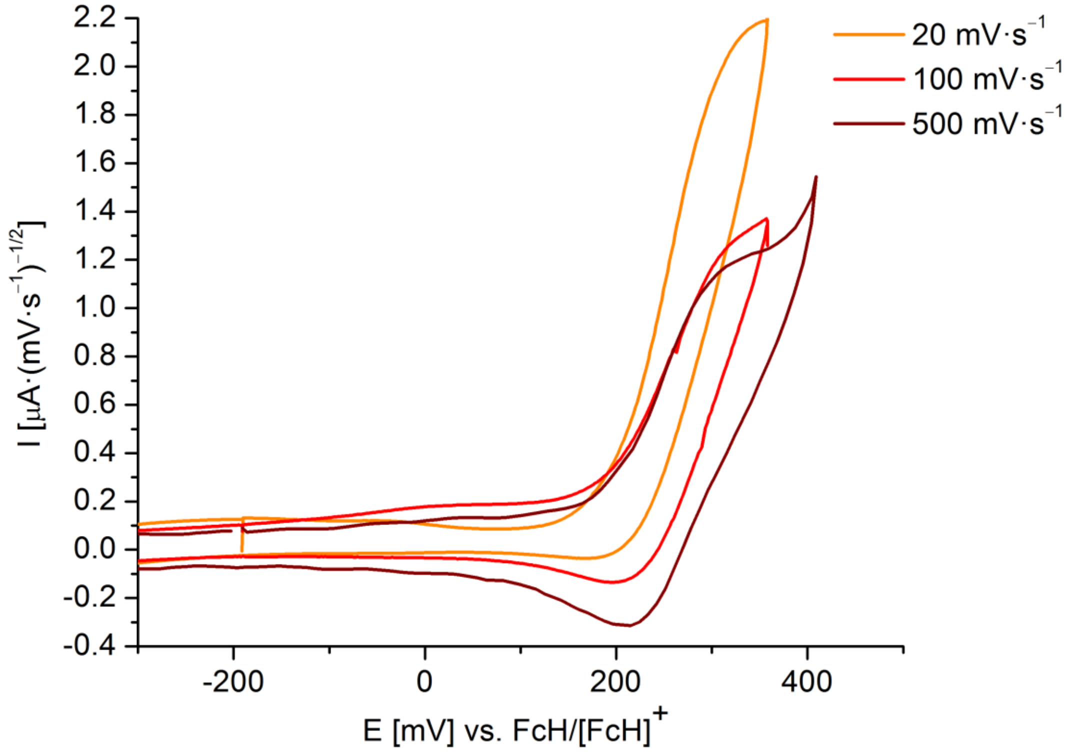

Figure S83. First oxidation of ligand **1** in (*n*Bu_4N)BAr^F_4/CH_2Cl_2 at and normalised for different scanning speeds (Randles-Sevčik plot), showing the increasing degree of reversibility of this oxidation at faster scanning. In all cases, the second of three consecutively measured cycles is shown.

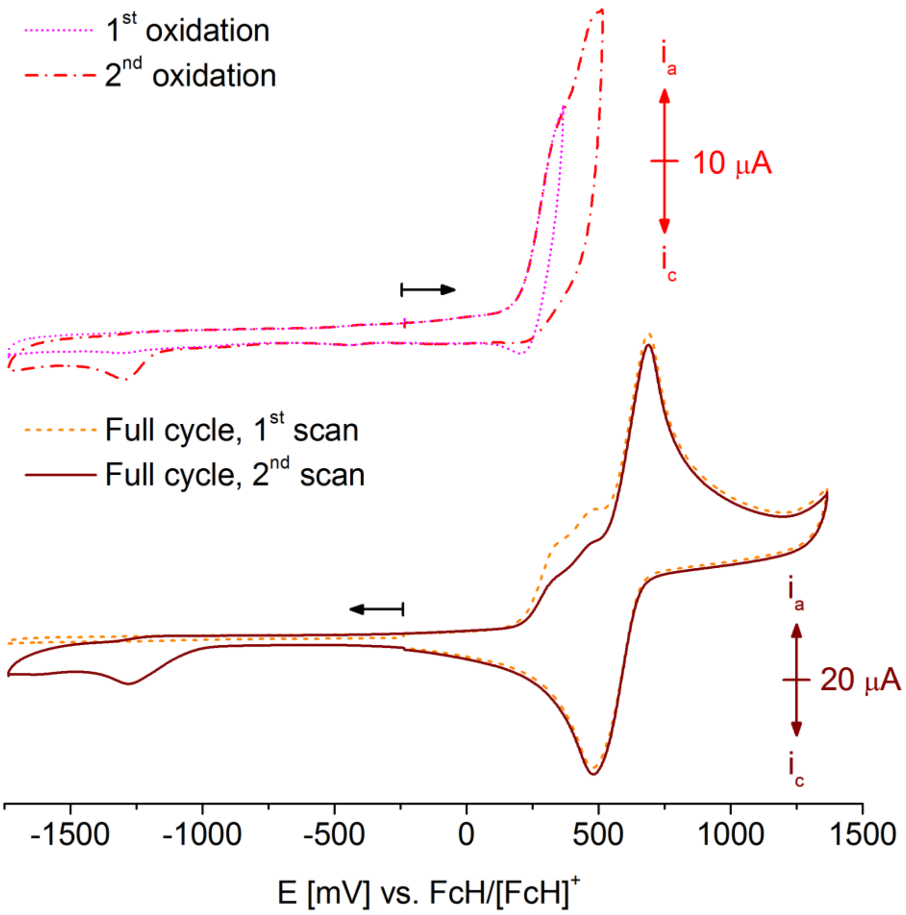

Figure S84. Cyclic voltammograms of complex **1Au** in (*n*Bu_4N)BF_4/CH_2Cl_2 with different turning potentials (top, 2nd cycles shown), indicating the presence of three oxidation events, and the dependency of the broad reductive events at about –1.2 V on prior oxidation (bottom). Arrows represent the starting potential and initial scan direction (100 mV·s^{-1}).

The electrochemical characterisation of **1Au** in the BF_4^--based SE (Figure S84) reveals similar behaviour of this complex in comparison to free ligand **1**. Two oxidation events at 350 mV (pink dotted, top) and 490 mV (red dash-dotted, top) can be isolated by CV, the first of which has some degree of reversibility, while the second is linked to a cathodically shifted reduction at −1.3 V. This dependence on prior oxidation is also found when the scan direction is reversed (orange dashed, bottom). A third oxidation event at 690 mV appears to be (quasi)reversible, yet leads to a further broadening at the reductive event at around −1.3 V. Analysing a 1:1 mixture of **1** (red dotted) and **1Au** (blue dotted) under the same conditions (Figure S85) however demonstrates that the electrochemical characteristics of **1Au** are most likely not a result of **1Au** degrading into **1** during the experiment, since the recorded cyclic voltammogram (black bold) appears as the sum of both individual traces.

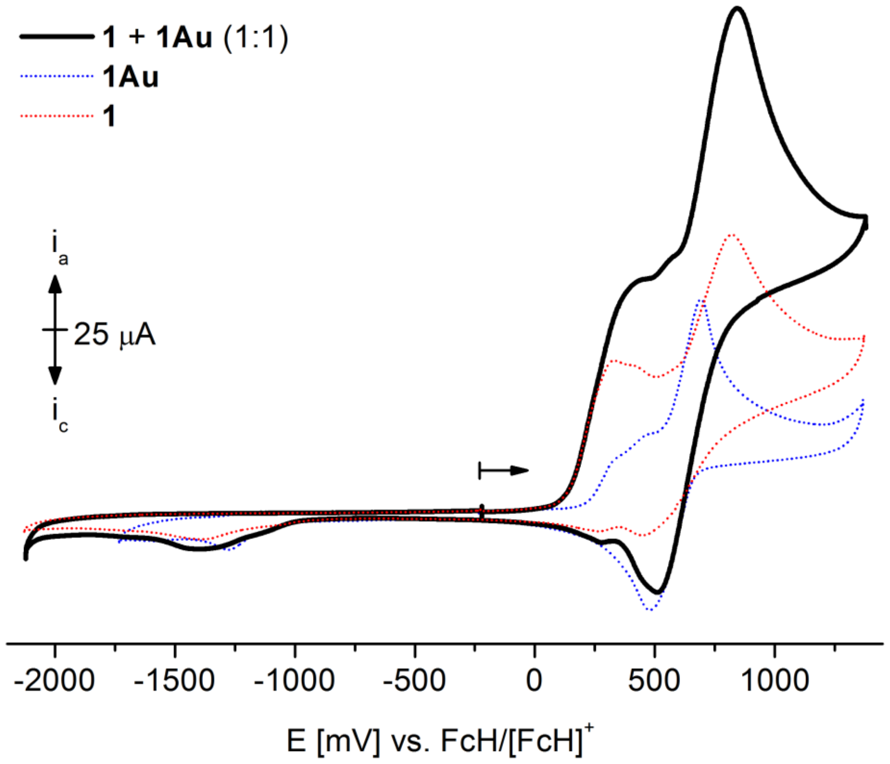

Figure S85. Cyclic voltammograms of a 1:1 mixture of ligand **1** and complex **1Au** in (nBu$_4$N)BF$_4$/CH$_2$Cl$_2$. The arrow represents the starting potential and initial scan direction (100 mV·s^{-1}).

In the BAr$^F{}_4{}^-$-based SE (Figure S86), the electrochemistry of **1Au** displays marked differences. The first (dotted pink) and, to some degree, reversible, and the second (dashed red), less reversible, oxidations appear more separated than in the BF$_4{}^-$-based SE, in line with the lower ability of BAr$^F{}_4{}^-$ anions to compensate positive charges by coming in close vicinity to the positive charges.[59] The third oxidation at about 1.2 V is much less reversible. Most notably, the cathodically shifted reduction observed for **1Au** in the BF$_4{}^-$-based electrolyte seems almost absent. Only at higher scanning speeds does a reductive event at about −1.2 V become apparent (Figure S87), in line with describing the electrochemistry of **1Au** as following an ECE mechanism. This Randles-Sevčik plot also indicates changes in the reductions at more anodic potentials.

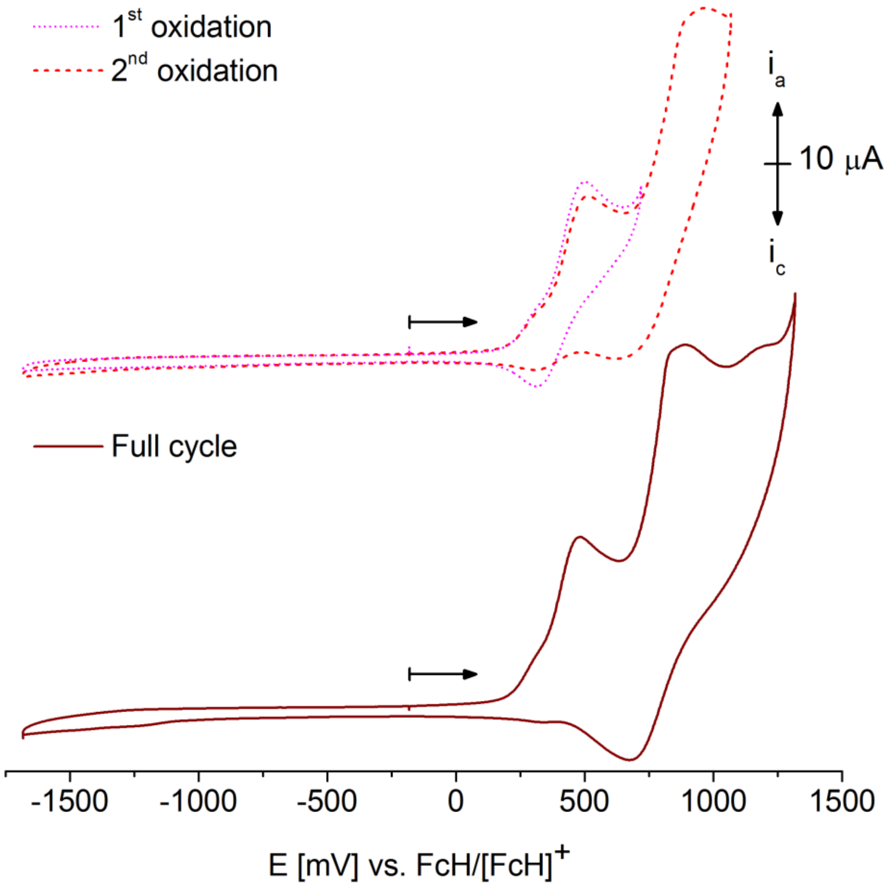

Figure S86. Cyclic voltammogram of complex **1Au** in (nBu$_4$N)BAr$^F{}_4$/CH$_2$Cl$_2$ with different turning potentials (top, 2nd cycles shown), indicating the presence of three oxidation events. Arrows represent the starting potential and initial scan direction (100 mV·s^{-1}).

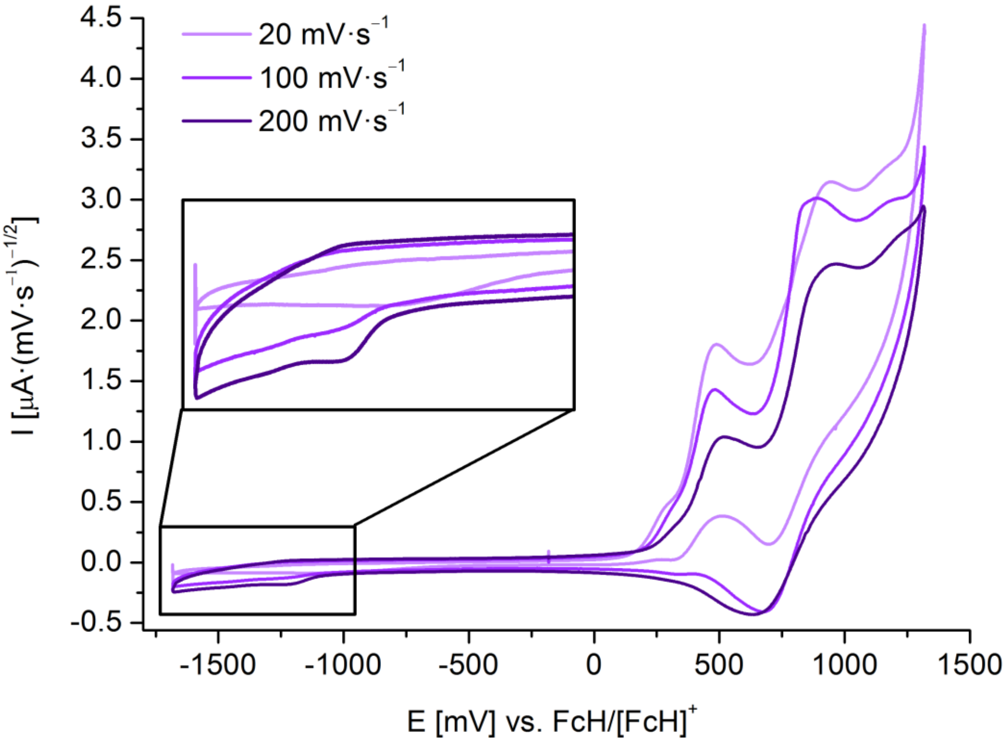

Figure S87. Cyclic voltammograms of complex **1Au** in (nBu$_4$N)BAr$^F{}_4$/CH$_2$Cl$_2$ at and normalised for different scanning speeds (Randles-Sevčik plot), showcasing the scan speed dependency. The insert shows the increasing visibility of a cathodically shifted reduction at about -1.2 V with increasing scan speed in detail. In all cases, the second of three consecutively measured cycles is shown.

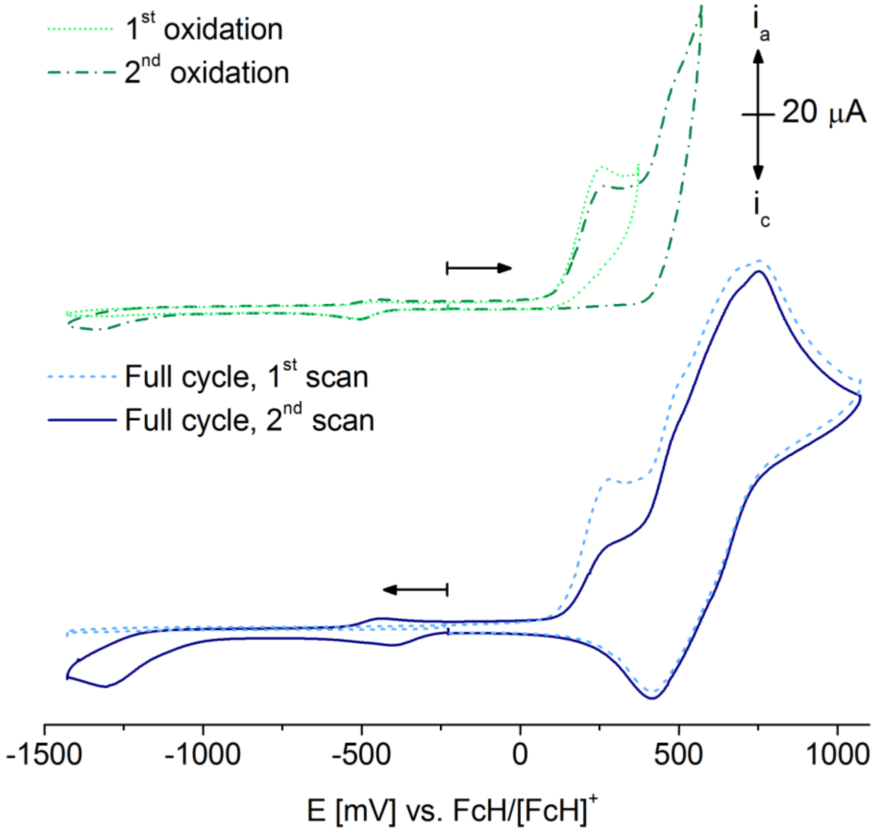

Figure S88. Cyclic voltammograms of complex **1Ag** in (*n*$Bu_4N)BF_4/CH_2Cl_2$ with different turning potentials (top, 2nd cycles shown), indicating the presence of at least three oxidation events, and the dependency of the reductive events at −500 mV and about –1.3 V on the prior oxidation (bottom). Arrows represent the starting potential and initial scan direction (100 mV·s^{-1}).

Regarding its electrochemical behaviour in the BF_4^--based SE (Figure S88), **1Ag** behaves similar to **1Au**. There are two oxidation events at 255 mV (dotted light green) and 500 mV (dash-dotted forest green) which are not reversible and entail cathodically shifted reduction events at −500 mV (linked to the first oxidation, itself again inducing another oxidation event at −450 mV) and −1.3 V (linked to the second oxidation). Further oxidation events at more anodic potentials intensify the reductive current at both potentials (solid navy), but none of the reductive events is present when the measurement is begun with a cathodic scan (bottom) but only appear after the first oxidative cycle.

Comparing the cyclic voltammograms at different scan rates (Figure S89) confirms these reductions to be linked to one or more chemical transformations that **1Ag** undergoes after initial and following oxidations, as both reductions disappear at slow scanning speeds. As for **1Au**, one or more EC(E) mechanisms seem to operate here.

In much the same way, following the electrochemistry of **1Ag** in the $BAr^F{}_4^-$-based SE (Figure S90) leads to a disappearance of the reduction events at about −500 mV, while the even more cathodic and oxidation-dependent reductions are still present. As for the BF_4^--based SE, slower scan speeds result in the disappearance of the reductive currents (not shown).

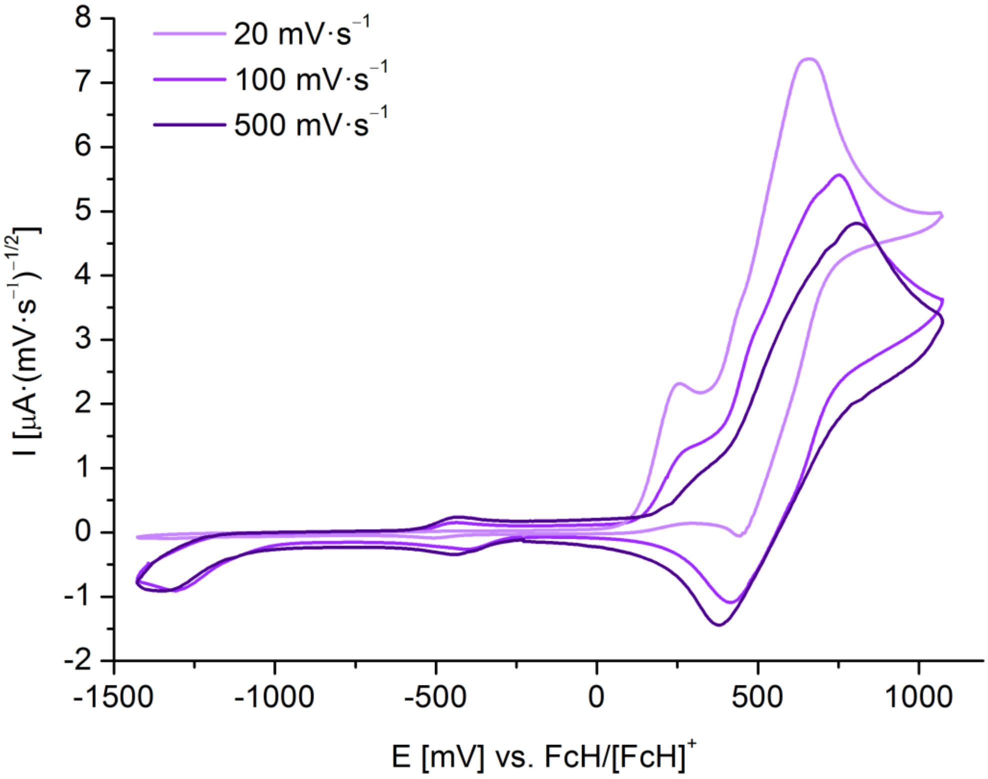

Figure S89. Cyclic voltammograms of complex **1Ag** in (nBu$_4$N)BF$_4$/CH$_2$Cl$_2$ at and normalised for different scanning speeds (Randles-Sevčik plot), showcasing the scan speed dependency particularly for the reduction events. In all cases, the second of three consecutively measured cycles is shown.

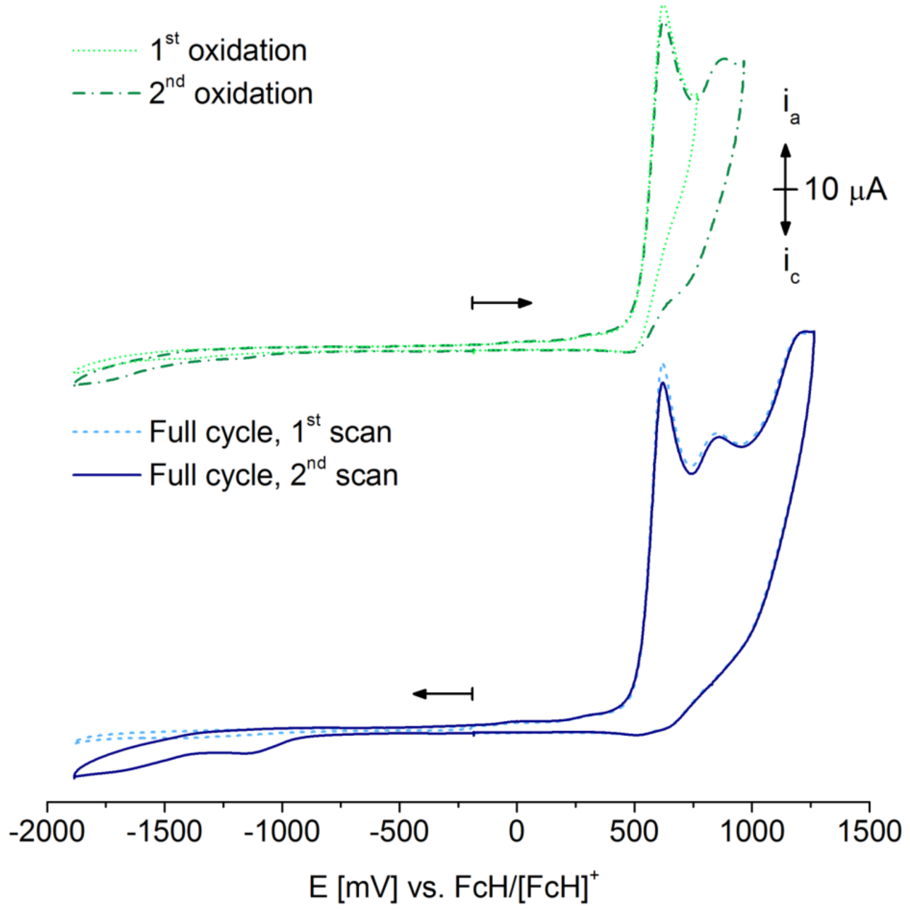

Figure S90. Cyclic voltammograms of complex **1Ag** in (nBu$_4$N)BAr$^F_4$/CH$_2$Cl$_2$ with different turning potentials (top, 2nd cycles shown), indicating the presence of three oxidation events. Arrows represent the starting potential and initial scan direction (100 mV·s^{-1}).

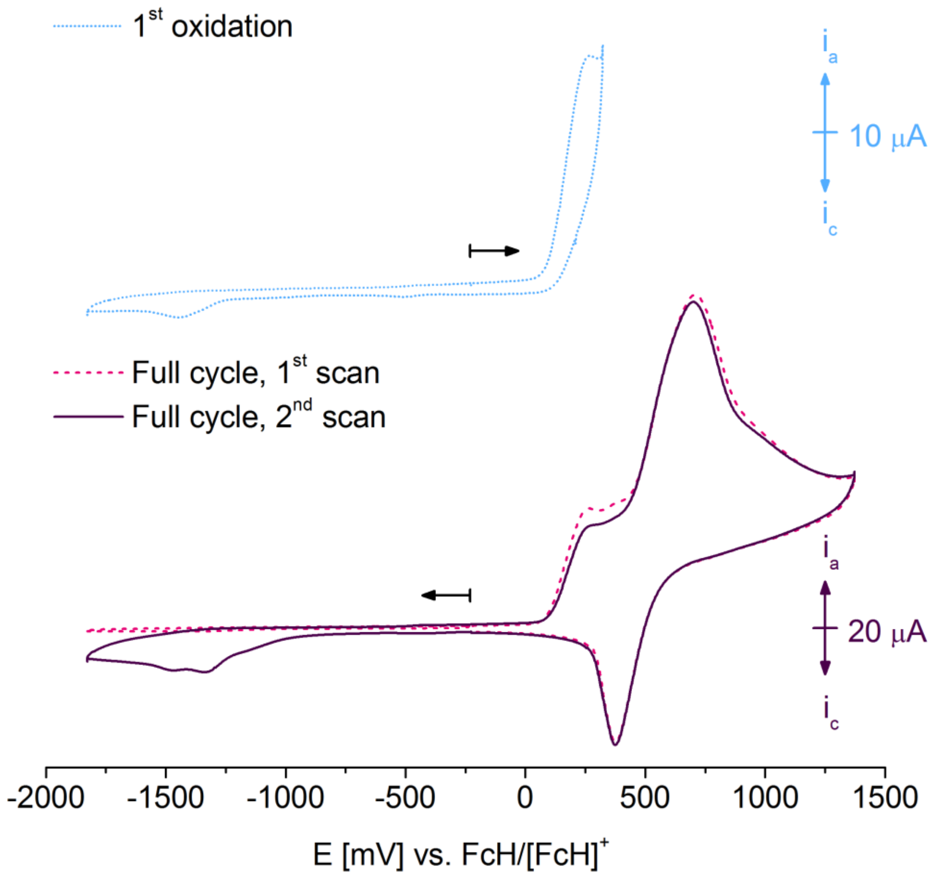

Figure S91. Cyclic voltammograms of complex **1CuBF₄** in (*n*Bu₄N)BF₄/CH₂Cl₂ with different turning potentials (top, 2nd cycles shown), indicating the presence of at least two oxidation events, and the dependency of the reductive events at about –1.5 V on the prior oxidation (bottom). Arrows represent the starting potential and initial scan direction (100 mV·s⁻¹).

The electrochemical characterisation of the copper(I) complexes in the BF_4^--based SE (Figure S91) was carried out using **1CuBF₄**. In contrast to **1Au** and **1Ag** under the same conditions, only one isolable first oxidation at 270 mV (top, dotted light blue) can be identified, tied to a cathodically shifted reduction event at about −1.4 V which is not present when the complex is initially scanned in cathodic direction (bottom, dashed berry) The first oxidation is followed by a broad oxidation event (bottom, solid dark purple) at about 700 mV (and potentially by further oxidations), which are linked to a comparatively sharp reduction peak at 380 mV and at least one more cathodically shifted reduction at about −1.3 V. While lowering the scanning speeds entails the disappearance of the reduction peaks (Figure S92), it also results in a second prominent yet also irreversible oxidation at higher potential (1.1 V), an unprecedented behaviour in comparison to both **1Au** and **1Ag** and potentially linked to the generation of an (unstable) Cu^0 species (*vide infra*).

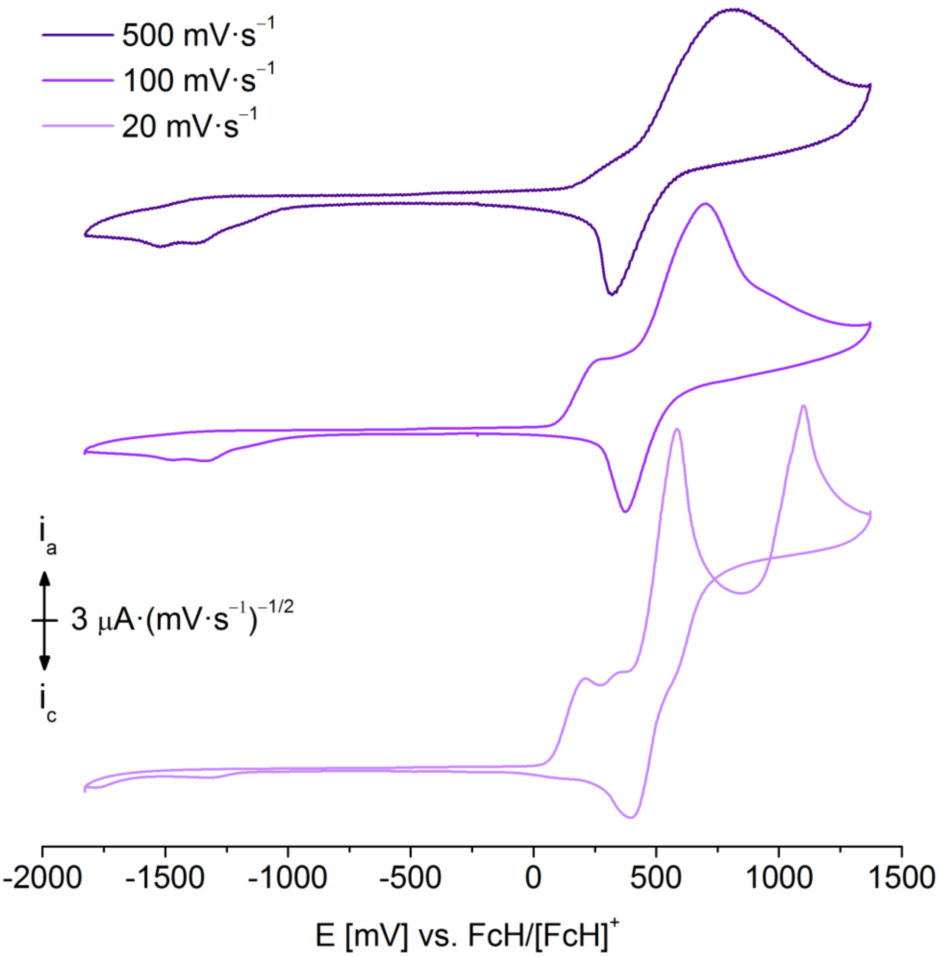

Figure S92. Cyclic voltammograms of complex **1CuBF₄** in (*n*$Bu_4N)BF_4/CH_2Cl_2$ at and normalised for different scanning speeds (Randles-Sevčik plot), showing the scan speed dependency particularly for the reduction events and the most anodic oxidation at about 1.1 V only visible at 20 mV·s^{-1}. In all cases, the second of three consecutively measured cycles is shown.

When following the electrochemistry of **1Cu** in the $BAr^F{}_4{}^-$-based SE (Figure S93), two oxidations at 640 mV (top, dotted light blue) and 890 mV (top, dash-dotted royal blue) can be identified again. In contrast to both **1Au** and **1Ag**, an initial cathodic scan (bottom, dashed berry) reveals a reduction at about −2 V original to **1Cu**, invariant to scanning speed (Figure S94) unlike the other reduction events at about −1.5 V (bottom, solid dark purple) related to the most anodic oxidation. The nature of this original reduction is only speculative but might correspond to the Cu^I/Cu^0 redox couple. The absence of a corresponding oxidation – no corresponding peak is found during the anodic scan even at higher scanning speeds and the oxidation events are independent of prior reduction – would be in line with the reported instability of mononuclear copper(0) complexes[60] which only lately have become synthetically available and for one of which, stabilised by cyclic alkylaminocarbene ligands, $E_{1/2}$ was determined to be −1.79 V vs. FcH/[FcH]$^+$ in THF.[60d]

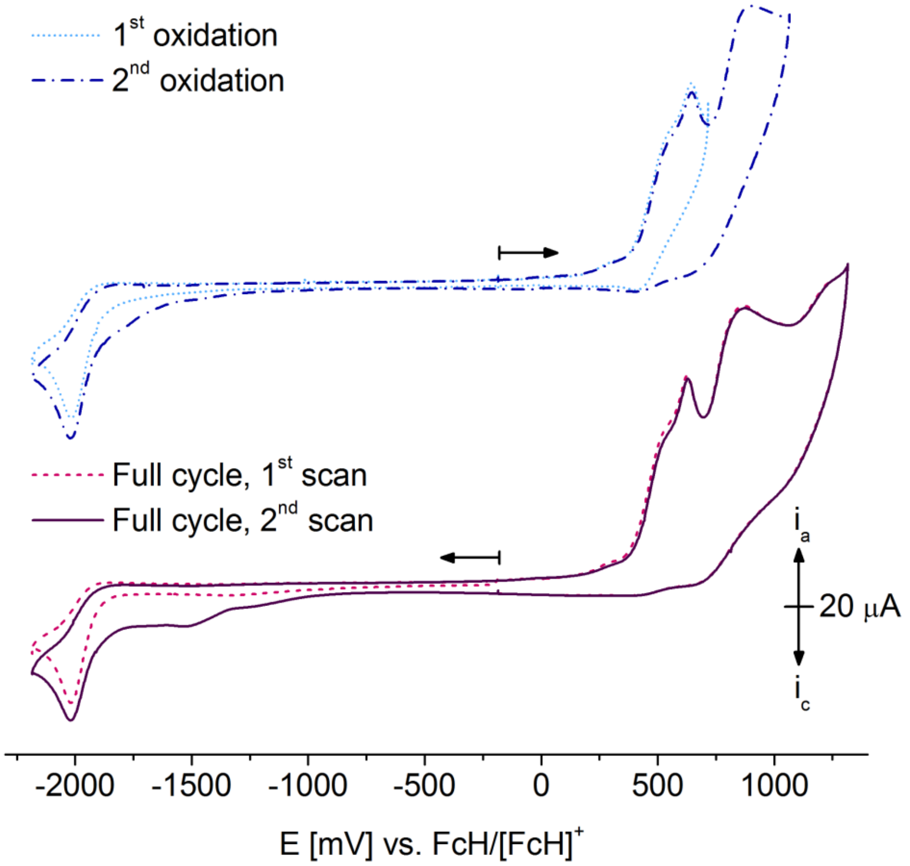

Figure S93. Cyclic voltammogram of complex **1Cu** in (*n*Bu_4N)BAr^F_4/CH_2Cl_2 with different turning potentials (top, 2nd cycles shown), indicating the presence of three oxidation events. Arrows represent the starting potential and initial scan direction (100 mV·s^{-1}).

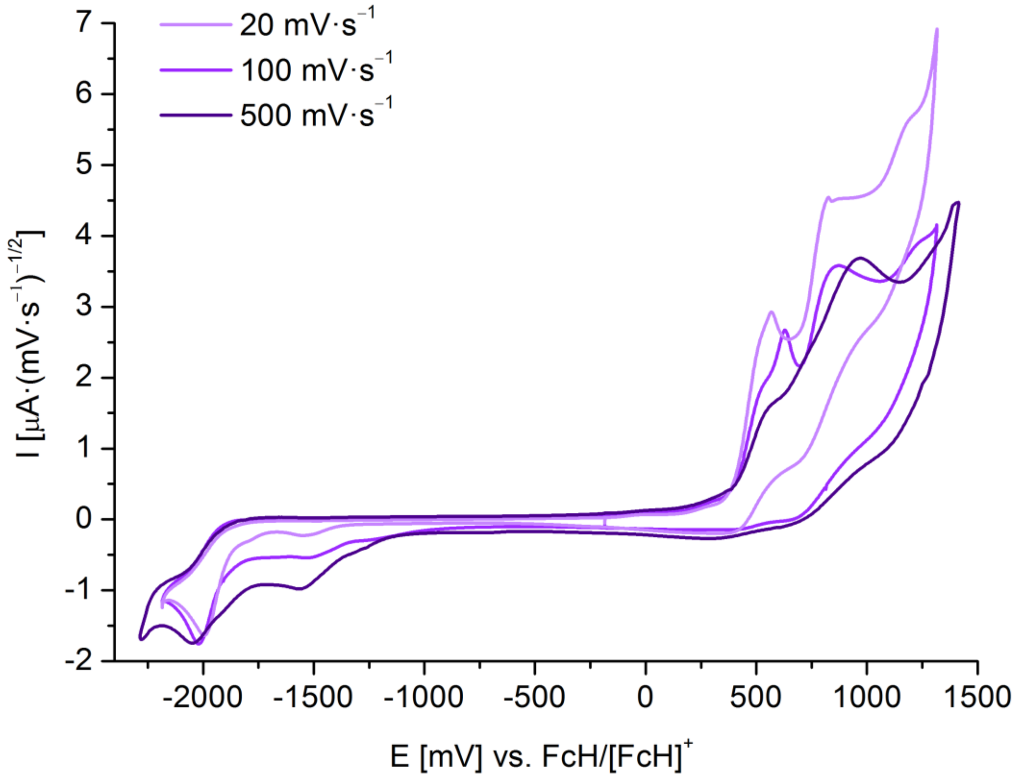

Figure S94. Cyclic voltammograms of complex **1Cu** in (nBu$_4$N)BAr$^F{}_4$/CH$_2$Cl$_2$ at and normalised for different scanning speeds (Randles-Sevčik plot), showing the scan speed dependency particularly for the reduction events at about −1.55 V and the contrasting invariance of the reduction at −2 V original to un-oxidised **1Cu**. In all cases, the second of three consecutively measured cycles is shown.

2.7. Spectroelectrochemistry

2.7.1. Spectroelectrochemical measurements of 1Au

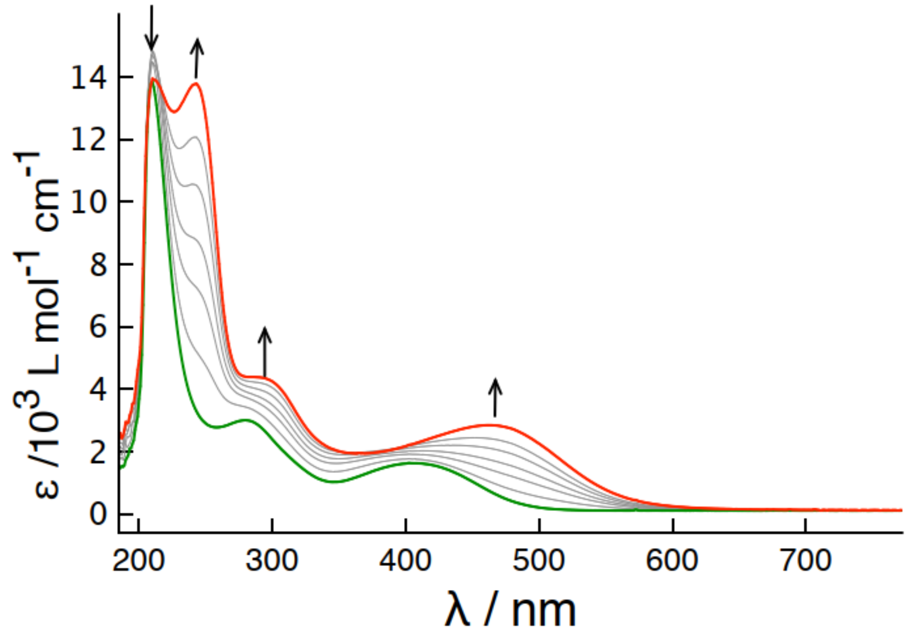

Figure S95. Time-dependent UV/Vis spectra recorded during the oxidation of **1Au** at 25 °C in (*n*Bu_4N)BAr^F_4/CH_2Cl_2. The green line represents the starting point, the red line represents the product after oxidation. Arrows indicate the direction of intensity changes.

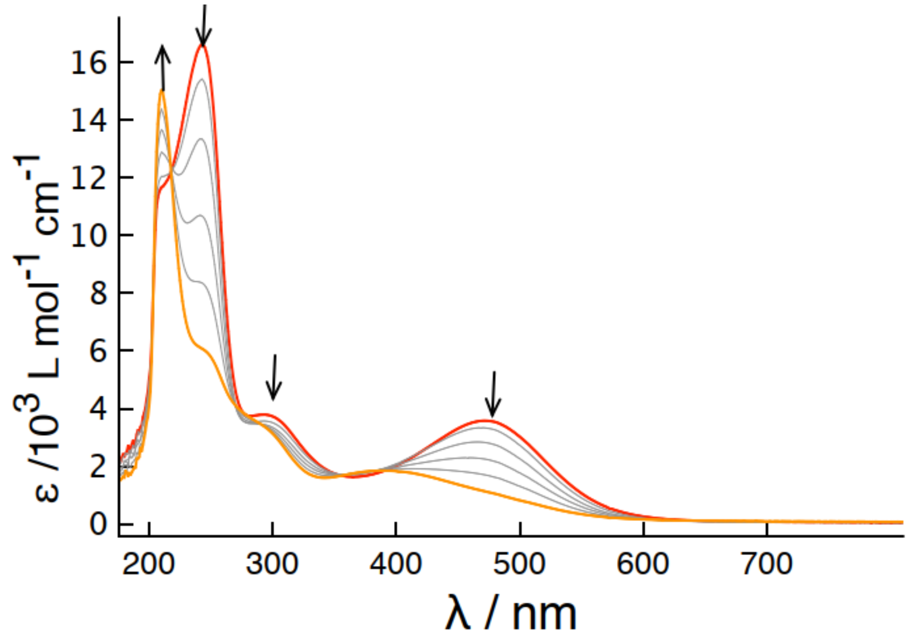

Figure S96. Time-dependent UV/Vis spectra recorded during the reduction after prior oxidation of **1Au** at 25 °C in (*n*Bu_4N)BAr^F_4/CH_2Cl_2. The red line represents the starting point – the spectrum obtained after prior oxidation – and the orange line represents the spectrum after oxidation. Arrows indicate the direction of intensity changes.

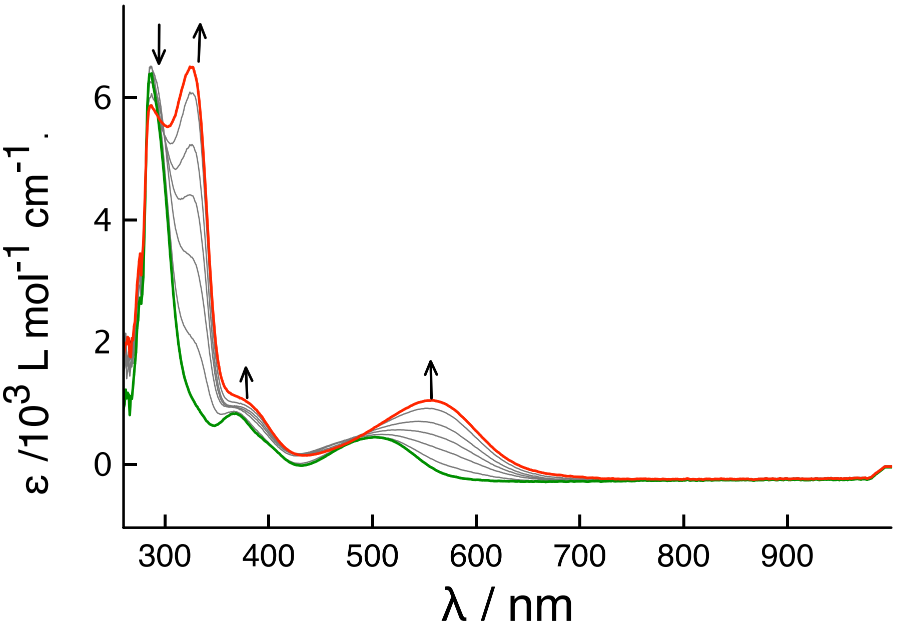

Figure S97. Time-dependent UV/Vis spectra recorded during the oxidation of **1Au** at −50 °C in (nBu$_4$N)BAr$^F{}_4$/CH$_2$Cl$_2$. The green line represents the starting point, the red line represents the product after oxidation. Arrows indicate the direction of intensity changes.

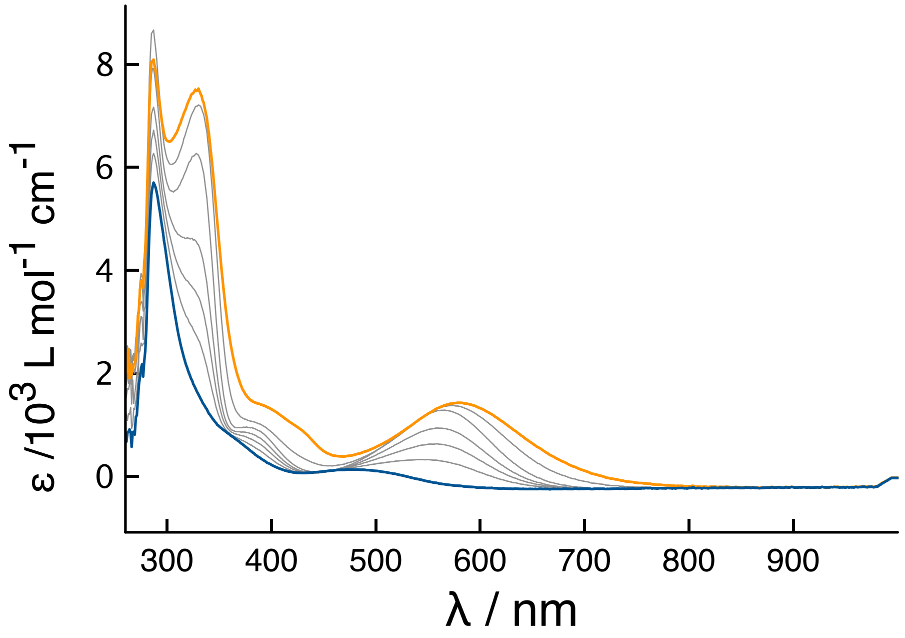

Figure S98. Time-dependent UV/Vis spectra recorded during the reduction after prior oxidation of **1Au** at −50 °C in (nBu$_4$N)BAr$^F{}_4$/CH$_2$Cl$_2$. The orange line represents the starting point – the spectrum obtained after prior oxidation – while the blue line represents the product after reduction.

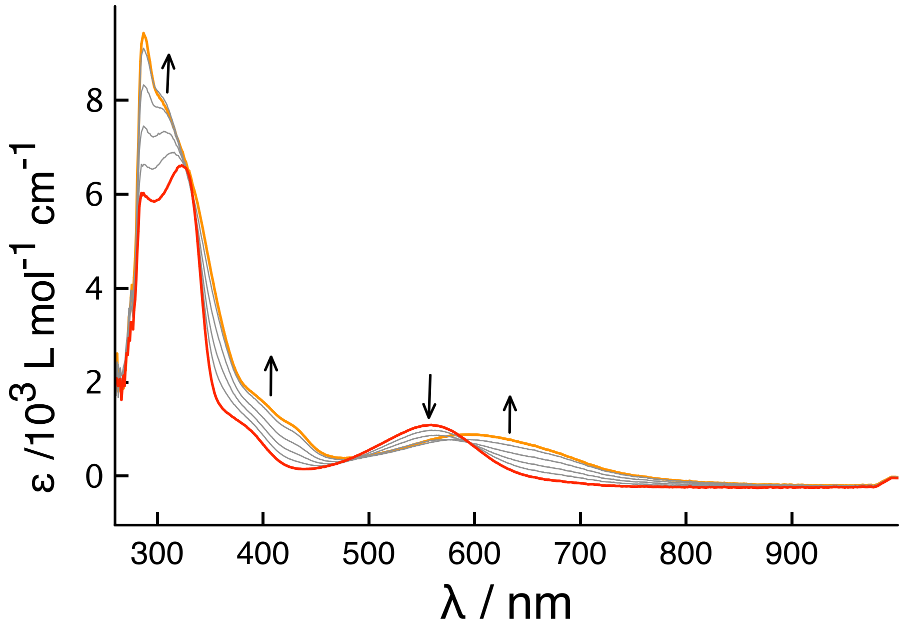

Figure S99. Time-dependent UV/Vis spectra recorded during the second oxidation of **1Au** at −50 °C in (nBu$_4$N)BAr$^F_4$/CH$_2$Cl$_2$. The red line represents the starting point – the spectrum obtained after prior oxidation – the orange line represents the product after second oxidation. Arrows indicate the direction of intensity changes.

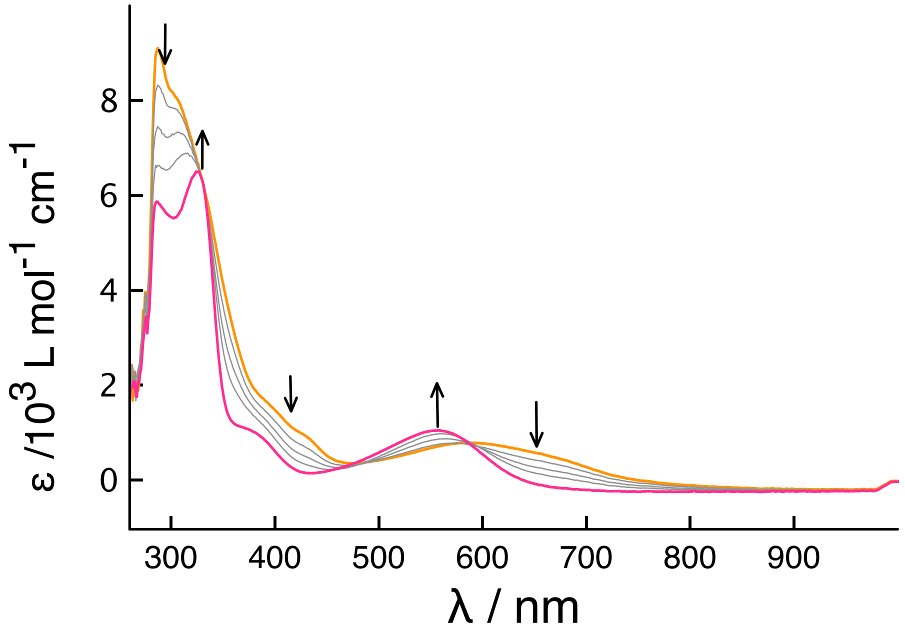

Figure S100. Time-dependent UV/Vis spectra recorded during the second reduction of **1Au** at −50 °C in (nBu$_4$N)BAr$^F_4$/CH$_2$Cl$_2$ after two previous oxidations. The orange line represents the starting point – the spectrum obtained after two prior oxidations – the pink line represents the product after the following reduction. Arrows indicate the direction of intensity changes.

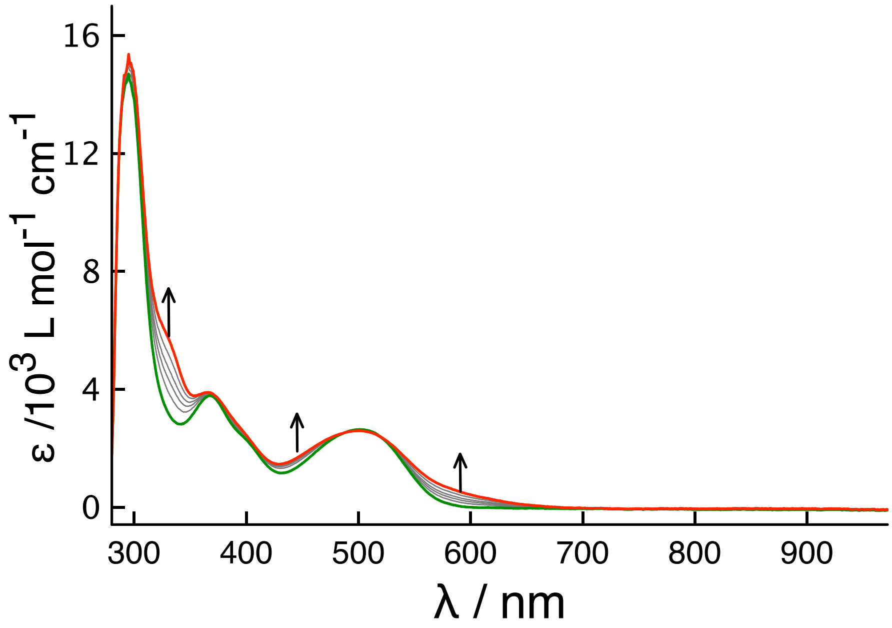

Figure S101. Time-dependent UV/Vis spectra recorded during the first oxidation of **1Au** at −80 °C in (nBu$_4$N)BAr$^F{}_4$/CH$_2$Cl$_2$. The green line represents the starting point and the red line represents the spectrum after oxidation. Arrows indicate the direction of intensity changes.

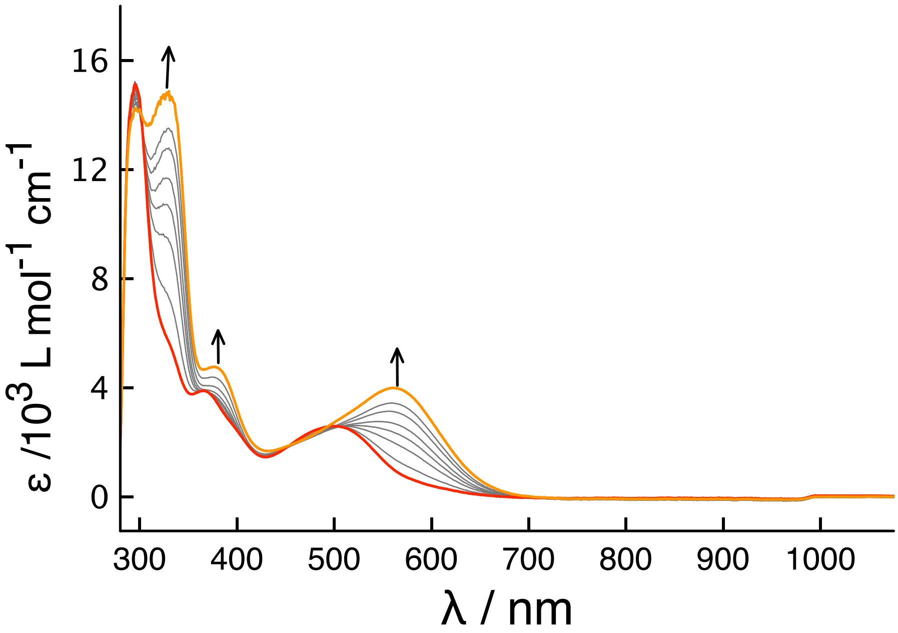

Figure S102. Time-dependent UV/Vis spectra recorded during the second oxidation of **1Au** at −80 °C in (nBu$_4$N)BAr$^F{}_4$/CH$_2$Cl$_2$. The red line represents the starting point – the spectrum obtained after the first oxidation – and the orange line represents the spectrum after the second oxidation. Arrows indicate the direction of intensity changes.

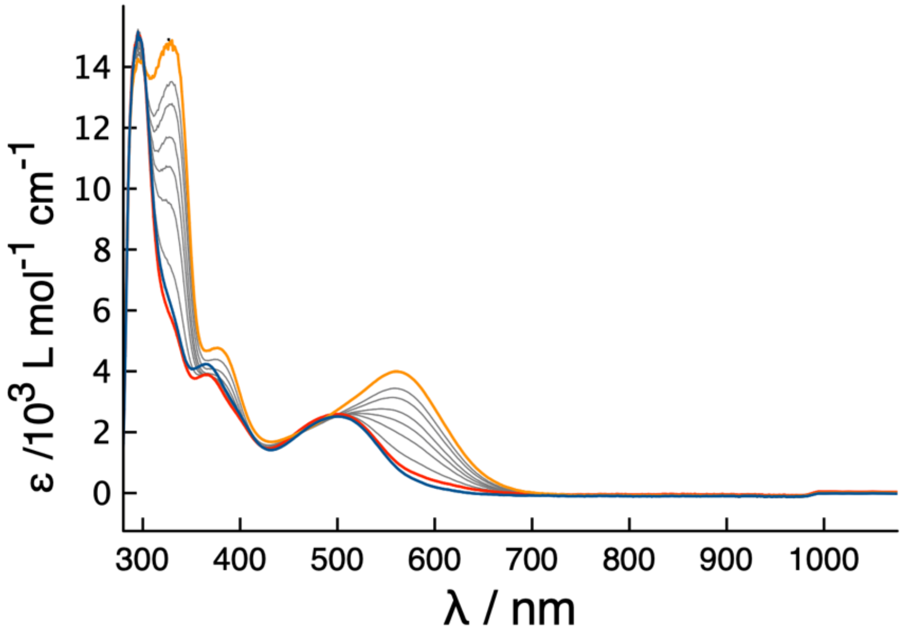

Figure S103. Time-dependent UV/Vis spectra recorded during the second oxidation of **1Au** at −80 °C in (nBu$_4$N)BAr$^F{}_4$/CH$_2$Cl$_2$. The red line represents the spectrum obtained after the first oxidation, the orange line represents the spectrum after the second oxidation, and the blue line represents the spectrum after reduction after the second oxidation.

As becomes apparent from the oxidation and reduction experiments, **1Au** cannot be reversibly oxidised at room temperature or at −50 °C; the UV/Vis spectra obtained after one oxidation-reduction cycle are not identical; hence, they most likely correspond to different compounds. The UV/Vis spectral change on the first oxidation at −80 °C is markedly different from the changes recorded at the other temperatures, and only the second oxidation brings about the same spectral features that have been found on oxidation at −50 °C and at 25 °C.

Comparing the spectra obtained from double oxidation at −80 °C (Figure S103, orange) and from the isolated oxidation product **5** (104, solid red) reveals them to be quite similar, particularly with respect to the intense absorption at about 330 nm and the shifted d(Fe)-π*(C_3N_3)/d(Fe)-d(Fe) transitions at about 575 nm. Though it is only speculative and a reaction with adventitious traces of water in the spectroelectrochemical setup seems unlikely, the similarity of the two spectra could point to the formation of **5** or a very similar species under these conditions, supporting the proposed ECE-type oxidation from **1Au** to **5** concurrent with oxidation at a phosphorus atom.

.

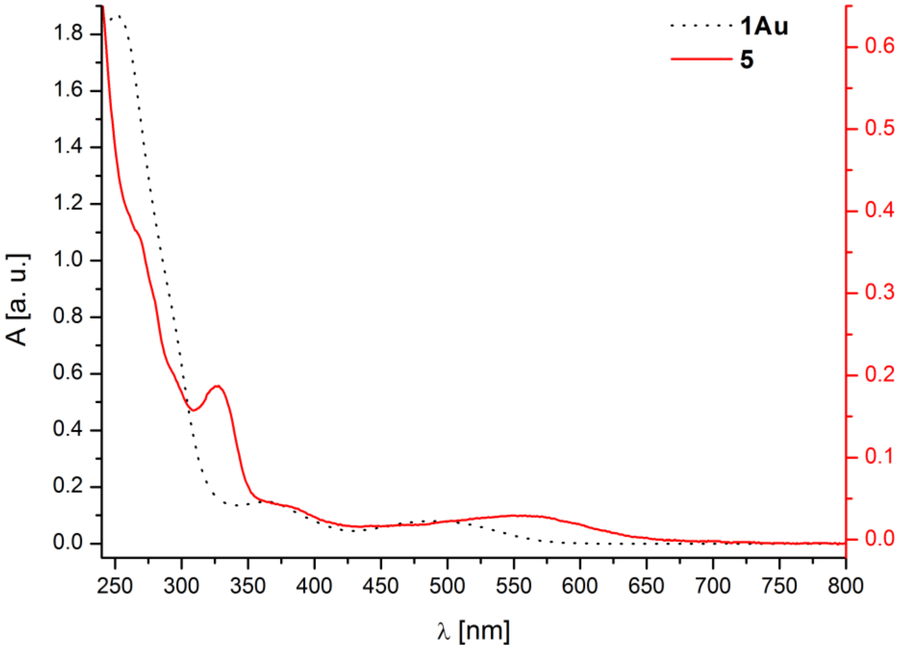

Figure S104. Comparison of the UV/Vis spectra of **1Au** (black, dotted) and isolated oxidation product **5** (red, solid), both in CH$_2$Cl$_2$. The spectra have been arbitrarily scaled for better comparability

2.7.2. Spectroelectrochemical measurements of 1Ag

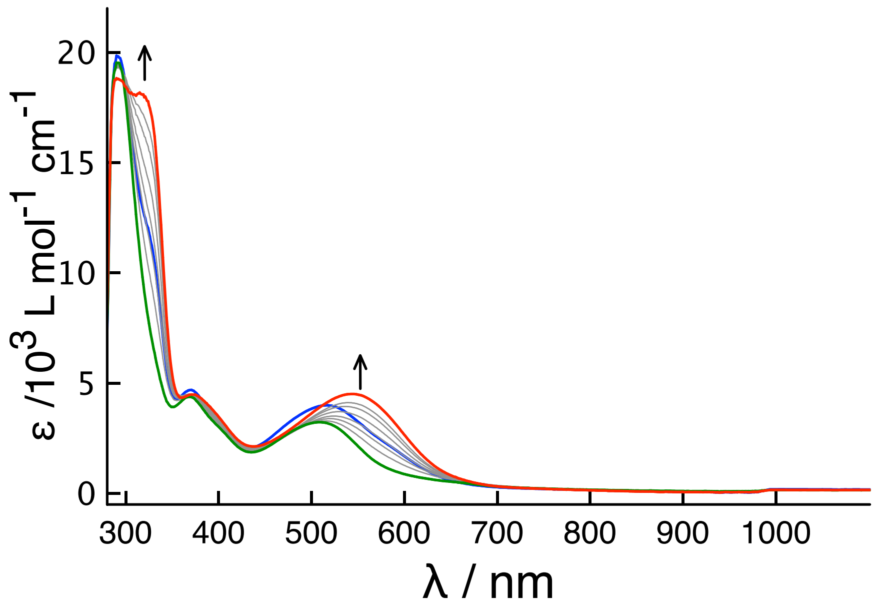

Figure S105. Time-dependent UV/Vis spectra recorded during the oxidation of **1Ag** at −80 °C in (nBu$_4$N)BAr$^F{}_4$/CH$_2$Cl$_2$. The green line represents the starting material, the red line represents the spectrum obtained after the oxidation and the blue line represents the spectrum after the subsequent reduction. Arrows indicate the direction of intensity changes during oxidation.

2.7.3. Spectroelectrochemical measurements of 1Cu

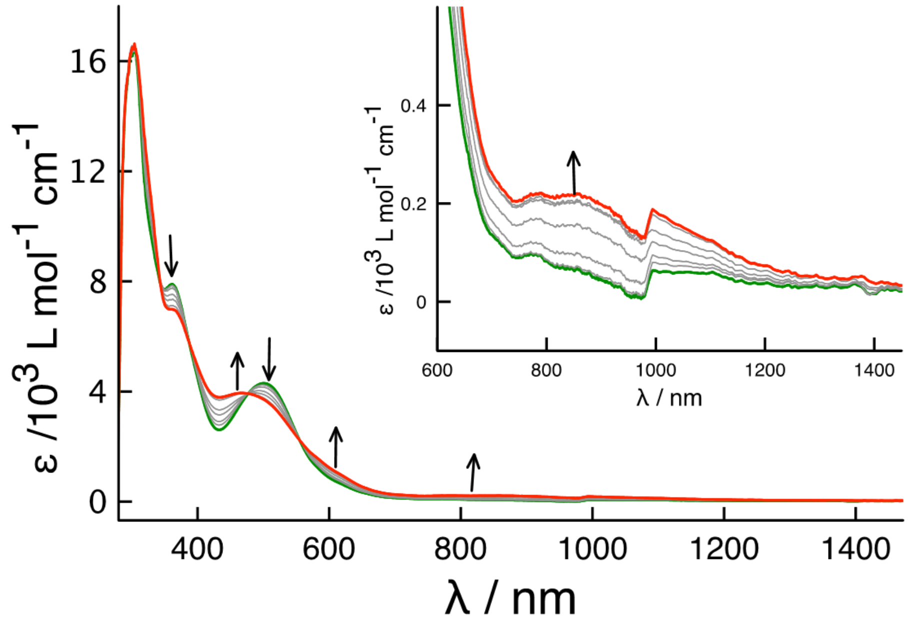

Figure S106. Time-dependent UV/Vis spectra recorded during the oxidation of **1Cu** at −80 °C in (nBu$_4$N)BAr$^F{}_4$/CH$_2$Cl$_2$. The green line represents the starting material, the red line represents the spectrum obtained after the oxidation. Arrows indicate the direction of intensity changes. The insert shows the region relevant to MMCT/IVCT transitions in detail.

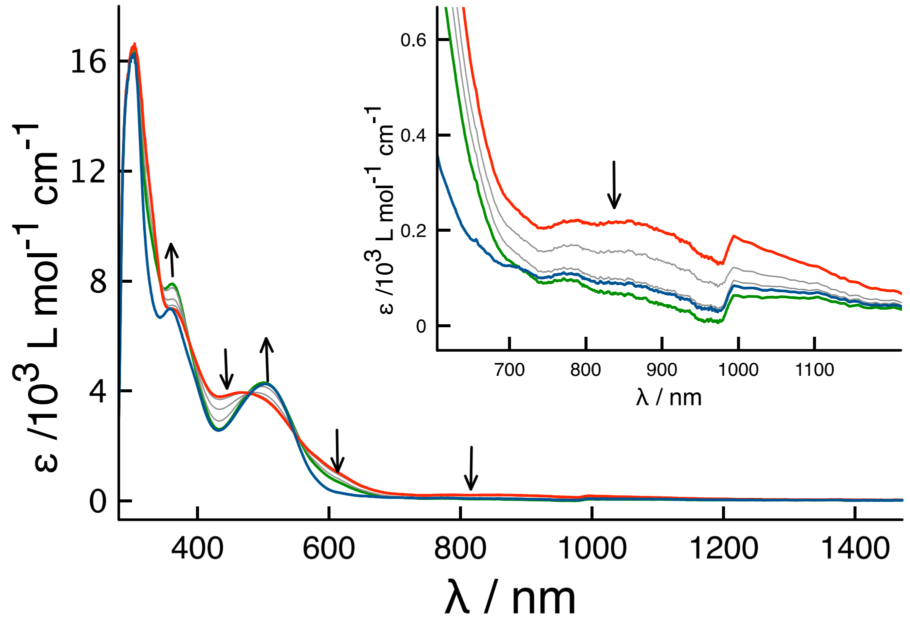

Figure S107. Time-dependent UV/Vis spectra recorded during the reduction of **1Cu** at −80 °C in (nBu$_4$N)BAr$^F{}_4$/CH$_2$Cl$_2$ after previous oxidation. The green line represents the starting material before oxidation, the red line represents the spectrum obtained after the oxidation and the blue line represents the spectrum obtained after subsequent reduction. Arrows indicate the direction of intensity changes during the reduction (red to blue). The insert shows the region relevant to MMCT/IVCT transitions in detail.

References

[1] H. C. Clark, A. B. Goel, R. G. Goel, S. Goel, W. O. Ogini, *Inorg. Chim. Acta* 1978, *31*, L441-L442.
[2] M. S. Inkpen, S. Du, M. Driver, T. Albrecht, N. J. Long, *Dalton Trans.* **2013**, *42*, 2813–2816.
[3] N. A. Yakelis, R. G. Bergman, *Organometallics* **2005**, *24*, 3579–3581.
[4] R. Taube, S. Wache, *J. Organomet. Chem.* **1992**, *428*, 431–442.
[5] B. Boduszek, H. J. Shine, *J. Org. Chem.* **1988**, *53*, 5142–5143.
[6] G. J. Kubas, B. Monzyk, A. L. Crumbliss, *Tetrakis(Acetonitrile)Copper(I) Hexafluorophosphate* in *Inorganic Syntheses* (Ed.: D. F. Shriver), John Wiley & Sons, Inc, Hoboken, NJ, USA, **1979**.
[7] T. N. Hooper, C. P. Butts, M. Green, M. F. Haddow, J. E. McGrady, C. A. Russell, *Chem. Eur. J.* **2009**, *15*, 12196–12200.
[8] R. K. Harris, E. D. Becker, S. M. Cabral de Menezes, R. Goodfellow, P. Granger, *Pure Appl. Chem.* **2001**, *73*, 1795–1818.
[9] I. Noviandri, K. N. Brown, D. S. Fleming, P. T. Gulyas, P. A. Lay, A. F. Masters, L. Phillips, *J. Phys. Chem. B* **1999**, *103*, 6713–6722.
[10] G. Gritzner, J. Kuta, *Pure Appl. Chem.* **1984**, *56*, 461–466.
[11] T. Mahabiersing, H. Luyten, R. C. Nieuwendam, F. Hartl, *Collect. Czech. Chem. Commun.* **2003**, *68*, 1687–1709.
[12] *CrysAlis Pro: Data collection and data reduction software package*, Agilent Technologies.
[13] *SCALE3 ABSPACK: Empirical Absorption Correction using Sperical Harmonics*, Oxford Diffraction, **2006**.
[14] G. M. Sheldrick, *Acta Crystallogr. Sect. A* **2008**, *64*, 112–122.
[15] C. F. Macrae, I. J. Bruno, J. A. Chisholm, P. R. Edgington, P. McCabe, E. Pidcock, L. Rodriguez-Monge, R. Taylor, J. van de Streek, P. A. Wood, *J. Appl. Crystallogr.* **2008**, *41*, 466–470.
[16] a) F. Neese, *Wiley Interdiscip. Rev. Comput. Mol. Sci.* **2012**, *2*, 73–78; b) F. Neese, *Wiley Interdiscip. Rev. Comput. Mol. Sci.* **2018**, *8*, e1327.
[17] a) J. P. Perdew, *Phys. Rev. B* **1986**, *33*, 8822–8824; b) A. D. Becke, *Phys. Rev. A* **1988**, *38*, 3098–3100; c) F. Weigend, R. Ahlrichs, *Phys. Chem. Chem. Phys.* **2005**, *7*, 3297–3305; d) S. Grimme, J. Antony, S. Ehrlich, H. Krieg, *J. Chem. Phys.* **2010**, *132*, 154104; e) S. Grimme, S. Ehrlich, L. Goerigk, *J. Comput. Chem.* **2011**, *32*, 1456–1465.
[18] D. Andrae, U. Häußermann, M. Dolg, H. Stoll, H. Preuß, *Theor. Chim. Acta* **1990**, *77*, 123–141.
[19] R. A. Kendall, H. A. Früchtl, *Theor. Chim. Acta* **1997**, *97*, 158–163.
[20] F. Neese, F. Wennmohs, A. Hansen, U. Becker, *Chem. Phys.* **2009**, *356*, 98–109.
[21] E. D. Glendening, A. E. Reed, J. E. Carpenter, F. Weinhold, *NBO* Version *3.1.*, Gaussian Inc., **2003**.
[22] M. J. Frisch, G. W. Trucks, H. B. Schlegel, G. E. Scuseria, M. A. Robb, J. R. Cheeseman, G. Scalmani, V. Barone, B. Mennucci, G. A. Petersson*, et al., Gaussian 09: Revision B.01*, Gaussian Inc., **2009**.
[23] a) W. R. Wadt, P. J. Hay, *J. Chem. Phys.* **1985**, *82*, 284–298; b) P. J. Hay, W. R. Wadt, *J. Chem. Phys.* **1985**, *82*, 270–283; c) P. J. Hay, W. R. Wadt, *J. Chem. Phys.* **1985**, *82*, 299–310.
[24] J. Tao, J. P. Perdew, V. N. Staroverov, G. E. Scuseria, *Phys. Rev. Lett.* **2003**, *91*, 146401.
[25] P. Štěpnička, I. Císařová, J. Sedláček, 10.1515/znb-1992-0103Jiří, M. Polášek, *Collect. Czech. Chem. Commun.* **1997**, *62*, 1577–1584.
[26] U. Pfaff, A. Hildebrandt, D. Schaarschmidt, T. Hahn, S. Liebing, J. Kortus, H. Lang, *Organometallics* **2012**, *31*, 6761–6771.
[27] H. Horn, F. Rudolph, R. Ahlrichs, K. Merzweiler, *Z. Naturforsch., B.: Chem. Sci.* **1992**, *47*, 1–4.
[28] S. Steffens, M. H. Prosenc, J. Heck, I. Asselberghs, K. Clays, *Eur. J. Inorg. Chem.* **2008**, 1999–2006.
[29] K. Škoch, I. Císařová, P. Štěpnička, *Inorg. Chem.* **2014**, *53*, 568–577.
[30] a) M. Pourayoubi, M. Toghraee, V. Divjakovic, A. van der Lee, T. Mancilla Percino, M. A. Leyva Ramírez, A. Saneei, *Acta Crystallogr. Sect. B* **2013**, *69*, 184–194; b) F. Hamzehee, M. Pourayoubi, M. Nečas, D. Choquesillo-Lazarte, *Acta Crystallogr. Sect. C* **2017**, *73*, 287–297.
[31] G. Gilli, P. Gilli, *J. Mol. Struct.* **2000**, *552*, 1–15.
[32] M. Sircoglou, N. Saffon, K. Miqueu, G. Bouhadir, D. Bourissou, *Organometallics* **2013**, *32*, 6780–6784.
[33] É. Fournier, A. Decken, P. D. Harvey, *Eur. J. Inorg. Chem.* **2004**, 4420–4429.
[34] J. Ellermann, J. Utz, F. A. Knoch, M. Moll, *Z. Anorg. Allg. Chem.* **1996**, *622*, 1871–1878.
[35] E. J. Sekabunga, M. L. Smith, T. R. Webb, W. E. Hill, *Inorg. Chem.* **2002**, *41*, 1205–1214.
[36] Q.-M. Qiu, X. Huang, Y.-H. Zhao, M. Liu, Q.-H. Jin, Z.-F. Li, Z.-W. Zhang, C.-L. Zhang, Q.-X. Meng, *Polyhedron* **2014**, *83*, 16–23.
[37] M. N. I. Khan, R. J. Staples, C. King, J. P. Fackler, R. E. P. Winpenny, *Inorg. Chem.* **1993**, *32*, 5800–5807.
[38] S. Alvarez, *Dalton Trans.* **2013**, *42*, 8617–8636.
[39] B. Cordero, V. Gómez, A. E. Platero-Prats, M. Revés, J. Echeverría, E. Cremades, F. Barragán, S. Alvarez, *Dalton Trans.* **2008**, 2832–2838.
[40] A. Baiada, F. H. Jardine, R. D. Willett, *Inorg. Chem.* **1990**, *29*, 4805–4808.
[41] V. J. Catalano, M. A. Malwitz, *J. Am. Chem. Soc.* **2004**, *126*, 6560–6561.
[42] V. J. Catalano, S. J. Horner, *Inorg. Chem.* **2003**, *42*, 8430–8438.
[43] W. Bensch, M. Prelati, W. Ludwig, *J. Chem. Soc., Chem. Commun.* **1986**, 1762.
[44] V. J. Catalano, B. L. Bennett, H. M. Kar, B. C. Noll, *J. Am. Chem. Soc.* **1999**, *121*, 10235–10236.
[45] K. Igawa, N. Yoshinari, M. Okumura, H. Ohtsu, M. Kawano, T. Konno, *Sci. Rep.* **2016**, *6*, 26002.
[46] W.-H. Chan, K.-K. Cheung, T. C. W. Mak, C.-M. Che, *J. Chem. Soc., Dalton Trans.* **1998**, 873–874.
[47] M. Sircoglou, M. Mercy, N. Saffon, Y. Coppel, G. Bouhadir, L. Maron, D. Bourissou, *Angew. Chem. Int. Ed.* **2009**, *48*, 3454–3457; *Angew. Chem.* **2009**, *121*, 3506–3509.
[48] a) S. Bontemps, G. Bouhadir, W. Gu, M. Mercy, C.-H. Chen, B. M. Foxman, L. Maron, O. V. Ozerov, D. Bourissou, *Angew. Chem. Int. Ed.* **2008**, *47*, 1481–1484; *Angew. Chem.* **2008**, *120*, 1503–1506; b) M. Sircoglou, S. Bontemps, G. Bouhadir, N. Saffon, K. Miqueu, W. Gu, M. Mercy, C.-H. Chen, B. M. Foxman, L. Maron*, et al.*, *J. Am. Chem. Soc.* **2008**, *130*, 16729–16738; c) E. J. Derrah, M. Sircoglou, M. Mercy, S. Ladeira, G. Bouhadir, K. Miqueu, L. Maron, D. Bourissou, *Organometallics* **2011**, *30*, 657–660; d) P. Gualco, S. Mallet-Ladeira, H. Kameo, H. Nakazawa, M. Mercy, L. Maron, A. Amgoune, D. Bourissou, *Organometallics* **2015**, *34*, 1449–1453.
[49] I. Caracelli, J. Zukerman-Schpector, E. R. T. Tiekink, *Gold Bull.* **2013**, *46*, 81–89.
[50] F. Almalioti, J. MacDougall, S. Hughes, M. M. Hasson, R. L. Jenkins, B. D. Ward, G. J. Tizzard, S. J. Coles, D. W. Williams, S. Bamford*, et al.*, *Dalton Trans.* **2013**, *42*, 12370–12380.
[51] M. Yoshizawa, K. Ono, K. Kumazawa, T. Kato, M. Fujita, *J. Am. Chem. Soc.* **2005**, *127*, 10800–10801.
[52] Y.-F. Han, H. Li, Z.-F. Zheng, G.-X. Jin, *Chem. Asian J.* **2012**, *7*, 1243–1250.
[53] K. Ono, M. Yoshizawa, M. Akita, T. Kato, Y. Tsunobuchi, S.-I. Ohkoshi, M. Fujita, *J. Am. Chem. Soc.* **2009**, *131*, 2782–2783.

[54] B. Therrien, G. Süss-Fink, P. Govindaswamy, A. K. Renfrew, P. J. Dyson, *Angew. Chem. Int. Ed.* **2008**, *47*, 3773–3776; *Angew. Chem.* **2008**, *120*, 3833–3836.
[55] M. Malischewski, K. Seppelt, J. Sutter, F. W. Heinemann, B. Dittrich, K. Meyer, *Angew. Chem. Int. Ed.* **2017**, *56*, 13372–13376; *Angew. Chem.* **2017**, *129*, 13557–13561.
[56] M. P. Mitoraj, A. Michalak, T. Ziegler, *J. Chem. Theory Comput.* **2009**, *5*, 962–975.
[57] V. N. Staroverov, G. E. Scuseria, J. Tao, J. P. Perdew, *J. Chem. Phys.* **2003**, *119*, 12129–12137.
[58] P. H. Rieger, *Electrochemistry,* Second edition, Springer Netherlands, Dordrecht, **1994**.
[59] F. Barrière, W. E. Geiger, *J. Am. Chem. Soc.* **2006**, *128*, 3980–3989.
[60] a) R. M. Wehmeyer, T.-C. Wu, G. W. Ebert, R. D. Rieke, *Tetrahedron* **1989**, *45*, 443–454; b) R. D. Rieke, B. T. Dawson, D. E. Stack, D. E. Stinn., *Synth. Commun.* **1990**, *20*, 2711–2721; c) G. Lefèvre, G. Franc, A. Tlili, C. Adamo, M. Taillefer, I. Ciofini, A. Jutand, *Organometallics* **2012**, *31*, 7694–7707; d) D. S. Weinberger, N. Amin SK, K. C. Mondal, M. Melaimi, G. Bertrand, A. C. Stückl, H. W. Roesky, B. Dittrich, S. Demeshko, B. Schwederski, *et al.*, *J. Am. Chem. Soc.* **2014**, *136*, 6235–6238.

Author Contributions

AS has carried out the syntheses and characterisation of the compounds, including the electrochemical and the (variable temperature) NMR experiments, and has written the original draft. PC has acquired and solved the solid-state structures of all compounds and carried out as well as analysed the computational investigations. MRR has carried out the variable-temperature spectroelectrochemical experiments and prepared the corresponding graphs as well as aided in their interpretation. EHH has supervised and administered the project, helped in acquiring funding for AS and PC and supported AS in the writing of the draft.

Appendix - Cartesian coordinates of optimised molecular structures

1Cu (as closed form $1Cu_{cl}$)

C	2.57650868769938	11.41722040597026	-2.83191164499309
H	2.65274119562630	12.27717465720373	-3.49935903034110
C	-1.53835658616125	10.54113456567428	2.37608613193408
C	2.49118602577796	9.09999758863714	1.89448191881326
C	3.47164478531743	14.69294409258391	0.86882586510248
C	5.34351192593789	13.74242533495080	-0.65140791506892
H	4.74391700307134	13.49348914961629	-1.51904698412542
C	1.97371331763883	15.75286772704642	2.22443972095483
C	-1.38174379624918	12.22451435894155	0.12146711315019
C	-2.64140280750835	12.71917597089316	-0.37416763987350
H	-3.38356089827165	13.25008253043284	0.21289562407041
C	4.52828078888990	10.96526453050693	1.33680638404215
C	-0.68846600262279	11.61110471007198	-0.98321789193220
H	0.28749004083969	11.14445573498934	-0.93915292006358
C	6.65396351778318	11.18819662203119	2.24062238829846
H	7.46453018547677	11.43099719384745	2.92140924784963
C	1.07343489527951	13.87483757920290	5.52720686320558
C	0.60690424181832	17.88871141970578	4.95404961773268
H	-0.16267253898206	18.46048576159984	5.46418261624701
C	1.69095041767577	16.51928213365997	3.43112921377194
C	2.39533888643006	9.21465612104817	-1.12702578407491
H	2.33616808370939	8.34833012377536	-0.46992444946169
C	4.84425802233974	14.29507192731300	0.58442438560123
C	5.96491182057145	14.46882755844150	1.46998349982353
H	5.90663162674758	14.85820111037793	2.47839162676721
C	1.60766742621350	14.55414721803469	6.68056078373789
H	2.62142943200845	14.45590491795532	7.05474419803219
C	1.33046133760464	15.03125498235086	0.15811010960379
C	2.31064656967335	9.03825926278178	-2.50999985163917
H	2.17836608725267	8.03526670333900	-2.91729004458267
C	-0.58953530382647	15.23674894559991	6.40151021295510
H	-1.53010665567330	15.76525601621779	6.52574254189695
C	2.62381400458454	16.84890326734276	4.48145055310206
H	3.65352388810577	16.51610101236660	4.53795564555807
C	1.33515880818621	11.06925773470143	5.54110973508160
C	-0.29182568926616	14.30547365596200	5.36037671343735
H	-0.96931670469915	13.97819814594622	4.58182427883425

C	5.48670409562626	10.56151173387638	0.33904878012185
H	5.24694307562028	10.23196256380854	-0.66655938680261
C	-0.60499603110044	14.72345717452718	-3.01005815812637
H	-0.76153895081572	14.42348442490080	-4.04200763085148
C	-2.82358735852865	15.61888224174636	2.90669428758597
H	-2.66185133763497	16.68918585951472	2.76828571417945
C	-1.51595952182153	11.72852960447768	-2.14139482440687
H	-1.25471373577648	11.39241006865465	-3.14049302096730
C	0.43422635600354	11.15380824544107	6.61242574292009
H	0.04091279511674	12.12151849626032	6.92137574311335
C	3.55146702806027	8.34000309458701	2.40891156087193
H	4.56337611337658	8.74327386645087	2.41024664046208
C	2.63780721118134	11.60465436908327	-1.45087632272819
H	2.71951682789507	12.61349455720327	-1.03928018609614
C	2.55639045368527	10.50045640357755	-0.58894715854230
C	-1.88289370193334	14.70822388319771	2.42516599559672
H	-0.96815509242120	15.06820126951918	1.94818028062933
C	-1.89877764082494	8.94182996013354	4.16593135516029
H	-1.78832139271028	8.67004974009303	5.21507807457484
C	3.61217353083032	12.75222739639523	5.20183032685814
C	-2.71692288965909	12.41368761542394	-1.76702562164236
H	-3.52677457849203	12.69293913517449	-2.43492123921547
C	0.43958466960624	17.15773666778286	3.74194563876352
H	-0.45957947661676	17.08930045143800	3.14316309312178
C	1.83902769358840	9.81578060289449	5.16266420283851
H	2.54365022240582	9.73528410430251	4.33646477765366
C	1.45539651212605	8.66466882206385	5.84826875613071
H	1.86153474373941	7.70086195676229	5.54354421099356
C	6.79231065879095	10.70358649915557	0.89978290687772
H	7.72894747407822	10.51787100027166	0.38195585997459
C	-1.40248758130467	10.16573037724163	3.72097663727604
H	-0.91642076450989	10.83432635456164	4.42958042202210
C	-2.09393367200646	13.33050298791555	2.58671918201647
C	-1.55591856893126	15.40233305655480	-2.17977288075698
H	-2.55565774466510	15.70683391673785	-2.47505102331308
C	2.40771066378976	10.13726742025612	-3.36656368706315
H	2.35195104141476	9.99647396925835	-4.44660148038109
C	1.19560414742993	8.56172898032716	1.89248956059793
H	0.36396940551408	9.13865827237988	1.49119894909725
C	0.34494648133497	15.00121544563506	-0.91480664812116
C	-4.16961677268936	13.80048986615844	3.75249143303278
H	-5.05759205511580	13.44078449767757	4.27353830227377
C	7.14024773444858	14.05112347417776	0.78019678985176
H	8.14684438094886	14.03839530548314	1.18763961211671
C	-3.24520930897424	12.88125669088384	3.25109860230961
H	-3.42424731288533	11.81467267685284	3.37818867205118
C	-3.96547350881644	15.17093450484612	3.57608572203350
H	-4.69296140083421	15.88693576181797	3.96017000299568
C	0.56239234716163	14.46781869796034	-2.23327316063370
H	1.46543496046617	13.96924881456658	-2.56158472944266
C	-0.98164772091934	15.56786733544666	-0.88440378389062
H	-1.44427619785522	16.04522476683535	-0.02855593350076
C	1.95283105444005	17.69973882748877	5.40921238752282
H	2.37882212534402	18.10534525965560	6.32211855708941
C	5.26215188544126	11.35383848784360	2.51495054125437
H	4.83133922473147	11.70748106262086	3.44318881054887
C	0.96280000296478	7.28072746192240	2.38952725463252
H	-0.04894822512811	6.87714571758151	2.37435269598057
C	-2.52257884090765	8.07155064940221	3.26743330902084
H	-2.90693512013204	7.11118263707735	3.61327547961112
C	0.58104627483330	15.39233929468310	7.21236611296088

H	0.68577905103701	16.06458163200949	8.05916574146429
C	3.31175918661123	7.06180888568193	2.92051383326512
H	4.14216168181301	6.47768619149669	3.31910977462645
C	6.75801904496304	13.60495651810722	-0.52718913213336
H	7.42549449581016	13.19819243130309	-1.28112338605624
C	4.15410707500267	11.94047944444686	6.20982116465471
H	3.56253569350594	11.13543556691699	6.64317879965138
C	5.67700057445311	14.01846062473676	5.13559313414042
H	6.27121901068344	14.83372345428152	4.71951391351147
C	2.02046641233610	6.52890125855489	2.90907084914347
H	1.83852685873001	5.52853441920869	3.30379742471884
C	-2.17156221924725	9.66912604959413	1.47882838367953
H	-2.28897785019499	9.95072137779783	0.43309256510998
C	4.38600952643415	13.78926994017257	4.65932065725379
H	3.98790940018124	14.39805688623114	3.84404356100312
C	5.45659939429735	12.16085457484979	6.66355675039729
H	5.87066854652145	11.51968845925390	7.44255130602996
C	0.04014250170486	9.99562706941760	7.28790416460894
H	-0.66159035601615	10.07059373148479	8.11952288510435
C	6.21916875853627	13.20411304738109	6.13339332969866
H	7.23273911810933	13.38042327424315	6.49533990792197
C	-2.65445438017412	8.43604707578628	1.92526734775750
H	-3.14531184219428	7.76372577541175	1.22051474383314
C	0.55066119412606	8.75133911246160	6.91020313543948
H	0.24642810847756	7.85057376154778	7.44471294651704
Cu	1.33941885944203	12.13441309209087	2.42641532690127
Fe	5.79677776776924	12.50186510398607	0.91283316318541
Fe	0.98903580466271	15.89295732500008	5.26124132398230
Fe	-1.07211818496616	13.57980830304436	-1.36790643973160
N	3.22694646145332	15.28840694219061	2.05429415424821
N	2.55572191450081	14.53233582233087	-0.10600816693263
N	0.98870816358392	15.64224698394011	1.30924491464693
P	-0.87828996973102	12.16910896558121	1.85173002789342
P	1.88636201385258	12.55088226925521	4.61254282219894
P	2.73870445694226	10.78436647851482	1.21479913057683

1Cu (as open form $1Cu_{op}$)

C	7.33587767894384	5.79862934893804	14.86893592144536
H	7.80768115509287	4.87332215919130	15.20392490125981
C	10.32427187955727	4.25643733954691	13.61599831654054
H	10.70427554198726	5.22899951941303	13.29508115791329
C	11.11993838164223	3.42727254338715	14.40681945262920
H	12.11642314296189	3.75535042759147	14.70514669622854
C	4.73874568628261	-0.06574092894123	13.68815059689136
H	4.28757565685522	0.27190853956746	14.61866715155013
C	1.83655922443088	4.68250645441024	13.56923350873452
C	0.60230120920382	-0.91251289455873	10.40303343918231
H	0.16508853787852	-1.78169338463146	10.88300658475507
C	2.17140880407535	-0.46331436293210	16.67346635202703
C	0.19749957736875	-3.92441839958269	14.21878693074238
H	-0.27513359122835	-4.85239421216791	14.54226802455749
N	1.10982675638341	3.56745833640526	13.20273435780178
C	3.90072578005785	-2.08908832151548	17.17662791202687
H	4.95391054340063	-2.32493699785189	17.33328037741792
C	5.89036646743993	-0.96621092914018	11.31411457998214
H	6.33937226238774	-1.32035577416767	10.38547122265168
C	0.33726295461362	1.30237896172447	18.67759010140621
H	1.24939714756181	0.88295719165951	19.10433893510366
C	4.04778900057511	2.69981779816941	15.77370527141116
H	4.10761567132497	2.42648022644898	14.72365195203233
C	-1.94648528156283	2.06112653093306	18.94535347064473
H	-2.80303824676944	2.26325337443305	19.58920099387482

C	1.85620001191349	1.01422149819221	10.03992725181262
H	2.49858920299199	1.87561644893096	10.19995729309099
C	6.11950782265738	-0.23101063884082	13.60635471416136
H	6.74137858968153	-0.00894731309326	14.47415275345795
C	3.03851529040171	2.25440535947130	16.71147873798563
C	0.25852408554201	1.54731485392827	17.29772726176565
C	5.27122529953326	2.44000495752919	10.71998722548376
H	4.90468879916906	1.73069633429029	9.98544594371968
C	0.70040320094044	3.59848959899266	11.89288693673555
C	1.41247741180477	-1.54702556919055	13.36665962902587
N	1.27732171335302	4.35809040076955	10.95669267714422
C	1.93977987735280	5.09180335334899	14.96509350981374
C	3.92212309648849	-0.33896063090274	12.58217178539989
C	4.92436026226020	5.39407991333628	8.81256513415823
H	5.46628632985720	5.07922594233597	7.92586379280387
N	2.43290922415396	5.47806164753560	12.68877864705293
C	-1.01839980683650	1.67669050854752	12.21836533506521
H	-0.77326907567668	1.39942711364758	13.24344123258451
C	1.55737865585285	-0.01615139589950	11.00565999793260
C	2.63420048841247	6.25331618659376	16.84845497735629
H	3.20957192660435	6.88384085376602	17.51947831140285
C	1.05495646348385	4.75632127069479	16.05066085259111
H	0.20818566851040	4.08391205070353	15.99254702757177
C	5.39062687568553	3.84016094564047	12.56561151696455
H	5.12638599159338	4.39496696237965	13.46032816954084
C	2.29210495802260	5.13342777577488	11.38897620913215
C	8.56703735216010	2.59292380356548	13.62989638124771
H	7.57172198029105	2.26157127141410	13.33188676219822
C	0.31589042549626	-0.43162969319514	9.08802589395637
H	-0.38946812262225	-0.87275038304826	8.38992649594135
C	0.03985640473885	-1.57657210032647	13.66307651328776
H	-0.56015274875115	-0.67363758815937	13.55490309564454
C	-0.41295492189433	2.75462052126722	11.47977995820967
C	3.52254634289543	-0.77919874052575	16.87343085611256
H	4.27888471611677	0.00462291712771	16.81847771735449
C	3.30882660889164	5.57883975253910	10.46169879898447
C	2.17695514035928	-2.70856887260607	13.53161124865810
H	3.24618768062549	-2.69027848604985	13.32174794683754
C	-1.98612735218853	1.04730042251806	11.37878304775679
H	-2.59350367639868	0.18628181378233	11.64093139874022
C	-1.03091744609502	2.78640319617151	10.17324308853182
H	-0.77430578427304	3.48344238070269	9.38310261145975
C	-2.04082848967135	2.27951372401208	17.56902002440723
H	-2.97056285909077	2.64763950528684	17.13410663753663
C	1.20171546106649	-1.47014911052019	16.80041612390259
H	0.14699055936029	-1.23209996224552	16.66082193550680
C	6.61582623856077	3.98842933902285	11.82400318004874
C	10.64647747305029	2.17279582598753	14.80335546824492
H	11.27234477032976	1.51931344254495	15.41209309827454
C	4.50560356282076	-0.80534708849248	11.39530341509314
H	3.87785694457690	-1.02834433207353	10.53191548958689
C	-2.00268390647343	1.74106899207555	10.12499699516449
H	-2.62549800955386	1.49311612066767	9.27079303127880
C	4.92912790041323	3.59054984987926	16.45427435774483
H	5.76562675922307	4.11505964816038	16.00549386242800
C	6.75381618975984	6.66393444382759	15.79894220053547
H	6.76962310153998	6.40919285784237	16.85992333048236
C	3.31586422923754	2.89211278702915	17.97402087694499
H	2.72388636540971	2.79701538743431	18.87821919627519
C	1.09521774896037	0.74884907479035	8.86062826675060
H	1.07752534022790	1.36394865568983	7.96590804070328

C	6.70071201728556	-0.66862047636040	12.41322797120612
H	7.78315529461577	-0.77697437370214	12.33973766256492
C	2.92193232330325	6.02129278881813	15.46987303955807
H	3.73677352405702	6.44776829181063	14.89409339051016
C	3.63259013481953	4.93488303927411	9.21050800770182
H	3.00423463124132	4.21766144196989	8.69304909034025
C	1.56930055146868	-3.89021831164139	13.96057885291346
H	2.17258241838248	-4.78934530006617	14.08912078351606
C	4.47537008068152	3.71500841498711	17.80629384786662
H	4.91444709198480	4.35071652737504	18.56971879393090
C	-0.56637356726846	-2.76290318110172	14.07456261956364
H	-1.63566565742249	-2.77935896934072	14.28925539196214
C	4.41790172736999	6.42606842055651	10.82830900437044
H	4.48124899189139	7.00476739226206	11.74238356362643
C	6.77640141790884	7.33812958095811	13.09531088053777
H	6.82282287381656	7.62438890030927	12.04353127541649
C	6.16106842710467	7.85643559847852	15.37694327294729
H	5.71038441217910	8.53331623240053	16.10373231735682
C	6.17637629593692	8.19412200131342	14.02032993602404
H	5.73874675108948	9.13561535471838	13.68558441352108
C	1.58192055767624	-2.77166163915727	17.11855719118690
H	0.82112429587154	-3.54493548695504	17.22325803057190
C	1.48286018299344	5.47709114747331	17.20528186973203
H	1.02800096777323	5.41832162262299	18.18955589806108
C	6.52625541986293	3.12243621402290	10.67428128022678
H	7.28495820396221	3.02267433918831	9.90339071117886
C	7.33823500267408	6.11785134785542	13.50282697221308
C	-0.94097692817650	2.02114729748650	16.74847952337222
H	-1.00754126557409	2.18784553898294	15.67056837514418
C	2.93249557663381	-3.08672354956531	17.29867904215067
H	3.22722530432468	-4.10754118330280	17.54362682343012
C	-0.75884486356058	1.56881141829528	19.49758506470988
H	-0.69152036058635	1.38337556537098	20.57018003814294
C	9.03487267556755	3.85386699676530	13.22879126194381
C	4.57201359326715	2.88381279240416	11.88494955637093
H	3.57272158454879	2.57595258156299	12.18435333187074
C	5.40253236317318	6.31321236921251	9.80202764649690
H	6.37329053373954	6.80023209917383	9.79049217483339
C	9.37274256213529	1.75777540013964	14.40796393695455
H	9.00433553331549	0.77597219406679	14.71115921058734
P	1.66251410724887	1.22031192383320	16.18580658232355
P	8.06242087322491	5.02894330856004	12.19901906477502
P	2.13730370529439	-0.00049918055308	12.71616512016911
Fe	-0.11378643490483	1.00917995877499	10.50164835134026
Fe	2.98091563870855	4.25720420546526	16.50261098688217
Fe	5.00040364549577	4.48514993731495	10.67248295937411
Cu	1.48312350154697	1.74640905703317	14.00847240212776

1Ag

C	2.57915034583885	11.40773479427514	-2.94501018509080
H	2.57493109965741	12.28120398532409	-3.59911409593680
C	-1.64438238784105	10.62008024904905	2.45606597672398
C	2.45889964395541	9.04888067997534	1.75747102778171
C	3.45603093383669	14.63837795296070	0.87596258804424
C	5.30952842281904	13.66918598927435	-0.65684126567533
H	4.69812698521877	13.39563984287703	-1.50851770710938
C	1.97419820376367	15.69958171503610	2.25001995812353
C	-1.47860563912874	12.19841246195247	0.10837627896621
C	-2.71050802866155	12.65511689434177	-0.48070342264062
H	-3.50508029813600	13.17378328682136	0.04644858065861
C	4.57489121889026	10.87966949704277	1.30721994579323
C	-0.68750366569946	11.59809167274379	-0.93668802481512

H	0.30023446007997	11.16810345364413	-0.81964242768360
C	6.62905144289940	11.21117997506091	2.33271342769936
H	7.38637428880318	11.50243683108803	3.05455675787833
C	1.07832139378583	13.86137037586686	5.62549580322880
C	0.65282288260180	17.87353488331245	4.97196320784177
H	-0.10320537714417	18.46853658120238	5.47583065918508
C	1.70571729865769	16.46749972433341	3.45967597324446
C	2.59152458339747	9.16824264124704	-1.27556849357641
H	2.59827082832039	8.29166291201991	-0.62836404042585
C	4.82552994196963	14.23875541120702	0.57730557045091
C	5.96027488187713	14.43794792271509	1.44003354270514
H	5.91623690265984	14.84650330088954	2.44174243973896
C	1.51690988620541	14.58380576118895	6.79148292980485
H	2.50479553973652	14.51885654073548	7.23656348226447
C	1.30931670984430	14.98917969490956	0.18526064899188
C	2.51459583833357	9.00796083685994	-2.66066422586940
H	2.45995732343412	8.00486153656341	-3.08517993462190
C	-0.66843435855103	15.21217014011603	6.33641619701088
H	-1.62484479793657	15.72505950097919	6.37669185872140
C	2.64149046591241	16.77553817756080	4.51398251156031
H	3.66030509094104	16.41250214821366	4.57970370515930
C	1.47942515199939	11.05544473715568	5.59450786349774
C	-0.27889165931120	14.25971133253907	5.34669466678024
H	-0.88912205755971	13.90743176829712	4.52340980940843
C	5.60600247384383	10.49422994786341	0.37897328937769
H	5.43988886092840	10.13597040108380	-0.63211368586056
C	-0.64068389230450	14.74127734213235	-2.97995738615639
H	-0.79929951563613	14.46587761476016	-4.01845069745425
C	-2.90443970503181	15.70673334517591	2.93851361405238
H	-2.69343528150992	16.77520413684528	2.87000684978170
C	-1.43141392146316	11.68177833265728	-2.15236432899074
H	-1.08889573590989	11.34576490911287	-3.12662931386886
C	0.60881528088387	11.06324829563158	6.69329076901426
H	0.21100147481329	12.00669430244092	7.06687069967270
C	3.47547858186708	8.21546011616161	2.24484897076153
H	4.51180058122849	8.55250115666283	2.22856739180183
C	2.64006334699450	11.57708561317421	-1.56149731200213
H	2.64902698279407	12.58070958756343	-1.12796648788163
C	2.65702063566043	10.45498053546354	-0.71941858173779
C	-1.97427244448382	14.78373742615809	2.45968077329060
H	-1.02064431333404	15.12388557569653	2.04734130461456
C	-1.87071314385353	9.07459369602876	4.31253533064911
H	-1.68677512066273	8.83307641313542	5.35873919994440
C	3.69295297179989	12.83870690462904	5.33519721180305
C	-2.67509589217324	12.33586810158356	-1.87286285112671
H	-3.44079738609175	12.58708690807708	-2.60130910182101
C	0.47063710476005	17.14135775333907	3.76292055516735
H	-0.42697746097707	17.09732741124908	3.15908500372161
C	1.98709715156989	9.83493868248377	5.12338062629354
H	2.65949557909915	9.81531680804477	4.26576272044239
C	1.64101164565037	8.63970303265246	5.75013825301422
H	2.04555911729294	7.70111118281723	5.37320807765754
C	6.86834004082461	10.70209763927832	1.01510185605323
H	7.84200524803645	10.54343528688148	0.56028466185186
C	-1.41668833766736	10.28817320343725	3.80040480656691
H	-0.88572323105492	10.98042149895699	4.45378421204835
C	-2.24784061038883	13.40984046636808	2.53813018713428
C	-1.59171149887402	15.39372855087816	-2.12959605453759
H	-2.59462091413348	15.69916778106996	-2.41300555232143
C	2.51400375300727	10.12603671845840	-3.49852037887178
H	2.45949855464120	9.99835728744984	-4.58028865135607

C	1.12863715926602	8.60274639069675	1.78100810842019
H	0.32879399270994	9.24456405780330	1.41174911210552
C	0.31599674256513	14.97154449220054	-0.88122684604068
C	-4.36874213745471	13.90296810520702	3.60647817372494
H	-5.29887498182614	13.55548994164318	4.05760408492029
C	7.12763623606465	14.02073711507033	0.73661763480772
H	8.14119644270392	14.02496917420395	1.12668736910431
C	-3.44979143271382	12.97298182810939	3.11582322136478
H	-3.66757495259615	11.90759929836615	3.18541037190978
C	-4.10113667047819	15.27116907418364	3.51433979547291
H	-4.82248871077580	15.99591424029751	3.89366630751424
C	0.53055060512088	14.47151291760587	-2.21374711390447
H	1.43555044386299	13.98718958845298	-2.55826987448576
C	-1.01354041954854	15.52986153146284	-0.83292537237960
H	-1.47754059541881	15.98147628572304	0.03590937149556
C	1.99020744456092	17.64868597021994	5.43471501080022
H	2.42268176201701	18.04515739523468	6.34860890554729
C	5.21819628191109	11.32760094295889	2.51718725677425
H	4.71781056515323	11.69438381571146	3.40561289274081
C	0.81968256221544	7.33551209003092	2.27093028032808
H	-0.21777141827507	7.00354023337439	2.27977299827422
C	-2.54449407277030	8.17336205011775	3.48254786239082
H	-2.89624624734081	7.22123434310732	3.88170831304368
C	0.43806740926003	15.41483074776600	7.22373593825176
H	0.46859194564933	16.11327625644057	8.05517396452547
C	3.16036461732307	6.94970316320221	2.74692451255156
H	3.95645916413566	6.30592302483889	3.12318016456622
C	6.72703128161853	13.54761367280630	-0.55528236238382
H	7.38493592387006	13.13291102722663	-1.31332308224876
C	4.20540047470416	12.11194925472350	6.42090584468489
H	3.59184536513998	11.35755081953824	6.91263713608429
C	5.78536962770577	14.04763923816212	5.17228429952140
H	6.40425758047337	14.80355359710139	4.68607745251329
C	1.83539242306384	6.50667334683187	2.75701069024652
H	1.59366665992885	5.51634491604593	3.14484707870192
C	-2.32109457508648	9.71506196808680	1.62632564954081
H	-2.50324486476775	9.96473914867377	0.58113893684722
C	4.49371100108817	13.80293942908515	4.70498467031296
H	4.11038888773531	14.34088592530051	3.83378593352739
C	5.50490873062653	12.35186008307224	6.87221423368687
H	5.89753415705607	11.78028380424520	7.71396943387809
C	0.25466137683069	9.86116687858832	7.31234779415712
H	-0.42188459468616	9.87465955533217	8.16785532643301
C	6.29561101905581	13.32332881877334	6.25315094973568
H	7.30862701667762	13.51240479633914	6.61028774568753
C	-2.76481357168082	8.49346130394444	2.14058272192033
H	-3.29190556998887	7.79458396432494	1.48979534309410
C	0.77189883770232	8.65026691057023	6.84540710195127
H	0.49724375287908	7.71453316362656	7.33383417789881
Ag	1.35910236252304	12.20704374515639	2.39585086571400
Fe	5.80305491979414	12.45919451675013	0.92157396924861
Fe	0.97589034941423	15.86786323206750	5.29154831999164
Fe	-1.09531421183657	13.55180219949808	-1.36628791317687
N	3.22409378676307	15.23006444772171	2.06687654398063
N	2.53041561387598	14.48447057688317	-0.09132689002747
N	0.97971978306554	15.59506330688041	1.34307385896148
P	-1.03012007088296	12.23201421804594	1.84707910047938
P	1.98244924082701	12.59014452516064	4.73534980331628
P	2.79996107557414	10.71604436062541	1.08587525335053

1Au

C	2.571527	11.421766	-2.887881
H	2.602232	12.293919	-3.542975
C	-1.617497	10.566229	2.407519
C	2.486166	9.050454	1.817798
C	3.463858	14.670304	0.872043
C	5.325231	13.703815	-0.653217
H	4.719427	13.441797	-1.512569
C	1.977338	15.734721	2.235560
C	-1.444848	12.211035	0.107162
C	-2.687351	12.688995	-0.443968
H	-3.461380	13.211261	0.109270
C	4.563348	10.911779	1.319738
C	-0.692121	11.607445	-0.963207
H	0.290671	11.160774	-0.873166
C	6.645480	11.207743	2.296821
H	7.423931	11.482839	3.002355
C	1.078954	13.879704	5.592117
C	0.633580	17.890214	4.961574
H	-0.129357	18.474037	5.468092
C	1.702050	16.501564	3.444554
C	2.499464	9.185910	-1.216443
H	2.477867	8.309541	-0.569738
C	4.834093	14.266290	0.581294
C	5.962068	14.453568	1.454438
H	5.910765	14.853274	2.459212
C	1.558451	14.590160	6.750289
H	2.557477	14.512245	7.167250
C	1.320927	15.016581	0.172924
C	2.410433	9.027512	-2.601127
H	2.315004	8.026704	-3.023837
C	-0.634001	15.238769	6.364107
H	-1.584493	15.759034	6.436990
C	2.636090	16.820238	4.496919
H	3.660379	16.472117	4.558585
C	1.410463	11.065563	5.590581
C	-0.282345	14.290021	5.356760
H	-0.918395	13.941694	4.552010
C	5.567062	10.523017	0.361863
H	5.372314	10.174464	-0.647359
C	-0.624242	14.740233	-2.992865
H	-0.781851	14.454039	-4.028594
C	-2.851129	15.663886	2.936093
H	-2.656257	16.732716	2.834376
C	-1.468576	11.710907	-2.156897
H	-1.158000	11.377959	-3.142854
C	0.514725	11.110195	6.667961
H	0.119679	12.066262	7.010413
C	3.524217	8.257925	2.327420
H	4.548567	8.629425	2.320960
C	2.640879	11.590761	-1.504867
H	2.684186	12.594928	-1.075096
C	2.615868	10.469702	-0.661462
C	-1.927368	14.741009	2.445250
H	-0.991561	15.085019	1.997159
C	-1.898507	8.981269	4.222355
H	-1.747048	8.717722	5.268526
C	3.659172	12.804748	5.280352
C	-2.695384	12.379129	-1.838389
H	-3.477770	12.645716	-2.543267
C	0.459292	17.158295	3.750974

H	-0.438399	17.102139	3.148522
C	1.915768	9.828352	5.161576
H	2.609019	9.781562	4.322181
C	1.538917	8.652857	5.807276
H	1.942057	7.700944	5.463513
C	6.846678	10.708755	0.968935
H	7.806946	10.543069	0.488840
C	-1.430382	10.205889	3.751031
H	-0.919102	10.884145	4.433584
C	-2.182798	13.366347	2.562709
C	-1.574905	15.405051	-2.151292
H	-2.576215	15.710827	-2.439995
C	2.453011	10.143460	-3.440342
H	2.391635	10.017111	-4.521895
C	1.171615	8.558991	1.828046
H	0.356401	9.168907	1.439785
C	0.329397	14.990637	-0.895447
C	-4.267043	13.860822	3.700903
H	-5.175168	13.513139	4.194693
C	7.133443	14.035850	0.758013
H	8.143684	14.031932	1.156613
C	-3.357983	12.929893	3.193588
H	-3.564369	11.864646	3.290423
C	-4.019770	15.229210	3.567549
H	-4.734884	15.954740	3.957047
C	0.544913	14.475911	-2.221498
H	1.449023	13.984695	-2.558144
C	-0.998257	15.553541	-0.855148
H	-1.461272	16.016992	0.008051
C	1.975163	17.682798	5.420894
H	2.404356	18.083616	6.334455
C	5.241053	11.338970	2.517771
H	4.764169	11.699415	3.421253
C	0.899963	7.288051	2.329936
H	-0.125448	6.920190	2.327906
C	-2.544838	8.097185	3.352947
H	-2.907144	7.136281	3.720462
C	0.499608	15.426002	7.220231
H	0.560338	16.117929	8.055468
C	3.245323	6.988856	2.842526
H	4.057821	6.377796	3.237654
C	6.741456	13.574812	-0.541113
H	7.403768	13.163322	-1.297078
C	4.196174	12.031408	6.321161
H	3.597168	11.251043	6.789076
C	5.737353	14.040634	5.144061
H	6.340356	14.827057	4.687209
C	1.936379	6.500889	2.841215
H	1.723415	5.507646	3.238429
C	-2.269019	9.679780	1.538579
H	-2.421262	9.951518	0.494380
C	4.441093	13.806055	4.684989
H	4.043214	14.379550	3.843790
C	5.502146	12.259301	6.760275
H	5.913585	11.650274	7.566015
C	0.129099	9.927086	7.304631
H	-0.568385	9.969125	8.142129
C	6.273403	13.268097	6.178084
H	7.290394	13.449728	6.527694
C	-2.725094	8.446434	2.012450
H	-3.230729	7.761457	1.330695

C	0.641882	8.699427	6.878955
H	0.342894	7.778801	7.381669
Au	1.335025	12.127313	2.430580
Fe	5.801284	12.479052	0.918588
Fe	0.984470	15.889683	5.277097
Fe	-1.086695	13.570368	-1.367980
N	3.228186	15.267652	2.058565
N	2.543164	14.513965	-0.098463
N	0.986989	15.628202	1.325500
P	-0.985867	12.186460	1.842300
P	1.937955	12.576398	4.706061
P	2.787138	10.722605	1.141710

Triazine+Cu

Cu	1.33926397356944	12.13354784675977	2.42667573577746
C	3.47134742098076	14.69214591315811	0.86901062465192
C	1.97360532597711	15.75200915072574	2.22488042287492
C	1.33008771432087	15.03055238479888	0.15857674513461
N	3.22681476863506	15.28753515638308	2.05454365173999
N	2.55530495208330	14.53163578876876	-0.10572909577999
N	0.98849119806575	15.64147615038767	1.30979275776982
H	0.56330021889960	14.93442419401237	-0.61504441253436
H	1.73945886069856	16.25486139314124	3.16721583673623
H	4.48191556676955	14.31644202186436	0.68650773362939

Triazine+Ag

Ag	1.36007570263307	12.20433669559540	2.39154911493072
C	3.45604256613390	14.63915610785080	0.87590649265235
C	1.97358776315031	15.69739555978449	2.25157487940205
C	1.30924123898827	14.99011668827597	0.18557203555339
N	3.22371145791428	15.22875333694003	2.06778282724192
N	2.53060047153968	14.48642633266197	-0.09173116770645
N	0.97924695031948	15.59392402551400	1.34435208340459
H	0.53482923955519	14.89752320942565	-0.58036716311476
H	1.75213911994202	16.20619629987658	3.19487152556003
H	4.46172548982381	14.25566174407511	0.68489937207617

Triazine+Au

Au	1.33308666173864	12.12596138222222	2.43201686396621
C	3.46304140084042	14.66769914329327	0.87295728007915
C	1.97708227956843	15.73303870787946	2.23637048276124
C	1.32024074603209	15.01486436216286	0.17387982308402
N	3.22770574838291	15.26535530323318	2.05939946638000
N	2.54222321840860	14.51163872281762	-0.09747398102221
N	0.98664019353348	15.62683080075094	1.32637422761878
H	1.75019723523975	16.23719457766255	3.17996119304853
H	4.47288004713670	14.29313201746458	0.68283613555581
H	0.55050246911897	14.92394498251332	-0.59767149147153
